# WFDC12-overexpressing contributes to the development of atopic dermatitis via accelerating ALOX12/15 metabolism and PAF accumulation

**DOI:** 10.1038/s41419-023-05686-3

**Published:** 2023-03-08

**Authors:** Guolin Li, Linna Gu, Fulei Zhao, Yawen Hu, Xiaoyan Wang, Fanlian Zeng, Jiadong Yu, Chengcheng Yue, Pei Zhou, Ya Li, Yuting Feng, Jing Hu, Nongyu Huang, Wenling Wu, Kaijun Cui, Wei Li, Jiong Li

**Affiliations:** 1 State Key Laboratory of Biotherapy and Cancer Center, West China Hospital, West China Medical School, Sichuan University and Collaborative Innovation Center for Biotherapy, Chengdu, China; 2 Department of Cardiology, West China Hospital, Sichuan University, Chengdu, China; 3 Department of Dermatovenereology, West China Hospital, Sichuan University, Chengdu, China

**Keywords:** Atopic dermatitis, Immunogenetics

## Abstract

Atopic dermatitis (AD) is a chronic inflammatory skin disease characterized by eczema-like skin lesions, dry skin, severe itching, and recurrent recurrence. The whey acidic protein four-disulfide core domain gene *WFDC12* is highly expressed in skin tissue and up-regulated in the skin lesions of AD patients, but its role and relevant mechanism in AD pathogenesis have not been studied yet. In this study, we found that the expression of WFDC12 was closely related to clinical symptoms of AD and the severity of AD-like lesions induced by DNFB in transgenic mice. WFDC12-overexpressing in the epidermis might promote the migration of skin-presenting cells to lymph nodes and increase Th cell infiltration. Meanwhile, the number and ratio of immune cells and mRNA levels of cytokines were significantly upregulated in transgenic mice. In addition, we found that *ALOX12/15* gene expression was upregulated in the arachidonic acid metabolism pathway, and the corresponding metabolite accumulation was increased. The activity of epidermal serine hydrolase decreased and the accumulation of platelet-activating factor (PAF) increased in the epidermis of transgenic mice. Collectively, our data demonstrate that WFDC12 may contribute to the exacerbation of AD-like symptoms in DNFB-induced mouse model by enhancing arachidonic acid metabolism and PAF accumulation and that WFDC12 may be a potential therapeutic target for human atopic dermatitis.

## Introduction

Atopic dermatitis (AD) is a common multifactorial chronic inflammatory disease characterized by impaired skin barrier, immune disorders, and dysbiosis of skin flora [1, 2]. Approximately 20% of children and 10% of adults in developed countries are affected by AD [3], which is clinically manifested as chronic lesions such as recurrent erythema eczema, dry skin, and severe itching [4]. The prevalence of changes in mood and sleeping disorders in AD patients is higher than that in the normal population, and normal life is hideously disturbed [5]. Patients’ epidermal barrier is usually blocked by genetic or other incentives, resulting in augmenting the percutaneous filtration rate and the barrier permeability, increasing the invasiveness of the penetrable antigens such as microbes and allergens from the external environment to the inside body [6], enhancing the absorption and presentation of antigens by antigen-presenting cells (APCs) such as epidermal Langerhans-cells (LCs) and dermic reside dendritic cells (rDCs) and the migration of APCs to the lymph nodes. Antigen presentation activates T cells and activates effector T cells migrate to the skin and participate in the regulation of local immune responses. Skin inflammation is the core mechanism of AD. In childhood or the acute phase of AD, the Th2-mediated immune response is the main mechanism in the human body. During this period, the release of the alarmins caused by the destruction of the epidermal barrier activates APCs, which migrate to lymph nodes and activate CD4^+^T cells to become Th2 cells and migrate to the skin lesions. The activated Th2 cells produce IL-4 and IL-13, which promote the production of antigen-specific IgE through signal transduction in B cells [7]. In the lesions of patients, keratinocytes release alarmins such as IL-33 and thymic stromal lymphopoietin (TSLP) to send pro-inflammatory and itchy signals. With the development of AD and the occurrence of chronic inflammation, Th1/Th17 cells become active and dominated via secreting a variety of cytokines to activate other immune cells and cooperate with inflammatory factors. Skin resident immune cells and T cells that migrate to lymph nodes produce large amounts of cytokines that recruit and activate other immune cells to regulate inflammation. With the progression of inflammation in the skin, mast cells, eosinophils, and neutrophils are involved in regulating the skin’s immune microenvironment [8]. The pathogenesis of AD contains many complex factors such as genetics, environment, and psychology, and the existing drug therapy has limited effects and certain side effects. Therefore, it is urgent to further study the mechanism and correlated functional molecules of AD to lay a foundation for the development of new drug targets and clinical treatment.

Arachidonic acid (AA), a polyunsaturated fatty acid with 20 carbon atoms and 4 double bounds, is mainly esterified to phospholipids at the second carbon (sn-2) of the phospholipid glycerol backbone of the cell membrane [9]. When keratinocytes respond to skin irritation and injury, AA is released from phospholipid by the action of phospholipases, and AA metabolism is then activated [10]. Increasing evidence highlights that AA metabolism and relevant enzymatic pathways are widely involved in many inflammatory diseases [11], such as asthma [12], arthritis [13], etc. However, the role of AA metabolism and relevant metabolic enzymes in the pathogenesis of AD remains elusive.

There are so many proteases and endogenous protease inhibitor molecules in human beings’ skin [14, 15]. They catalyze different substrates to form a complex hydrolysis network and play a role as key signal molecules in the cellular pathway and then participate in various physiological functions such as epidermal differentiation and desquamation, lipid synthesis, and cytokine secretion. The dysregulation of biochemical reactions caused by proteases is a key factor in the occurrence and development of various human skin diseases [14–20]. Endogenous protease inhibitor molecules also play a vital role in maintaining normal skin physiological functions and resisting the attack of skin diseases [21, 22]. The whey acidic protein 4-disulfide core domain 12 (WFDC12) is a member of the whey acidic protein (WAP) family, which has a core disulfide domain and has pleiotropic effects on protease inhibition, antibacterial and antiviral activities [23]. For example, the member of this family WFDC14, secretory leukocyte peptidase inhibitor (SLPI), and WFDC5 are highly expressed in the skin and participate in the regulation of skin homeostasis [24–26]. Meanwhile, SLPI has a higher plasma content in AD patients and promotes the migration of eosinophils to the lesions [27]. Both SLPI and WFDC14 have been shown to play an irreplaceable role in the development of psoriasis. These studies indicate that the members of this family have great academic and practical values in the clinical development and application in skin inflammatory diseases. As a molecule highly expressed in human skin specifically, WFDC12 is highly expressed in non-skin lesions and skin lesions in psoriasis and AD [26], but its characteristic role and underlying mechanisms in skin homeostasis and skin diseases remain mysterious.

Our findings revealed the unique role and potential mechanism of WFDC12 and AA metabolism pathway in the occurrence and development of AD, providing an experimental basis and theoretical basis for the clarification of AD pathogenesis and the search for new clinical drug targets and treatment methods.

## Results

### The expression of WFDC12 positively correlated with AD clinical feature

**Fig. 1:**
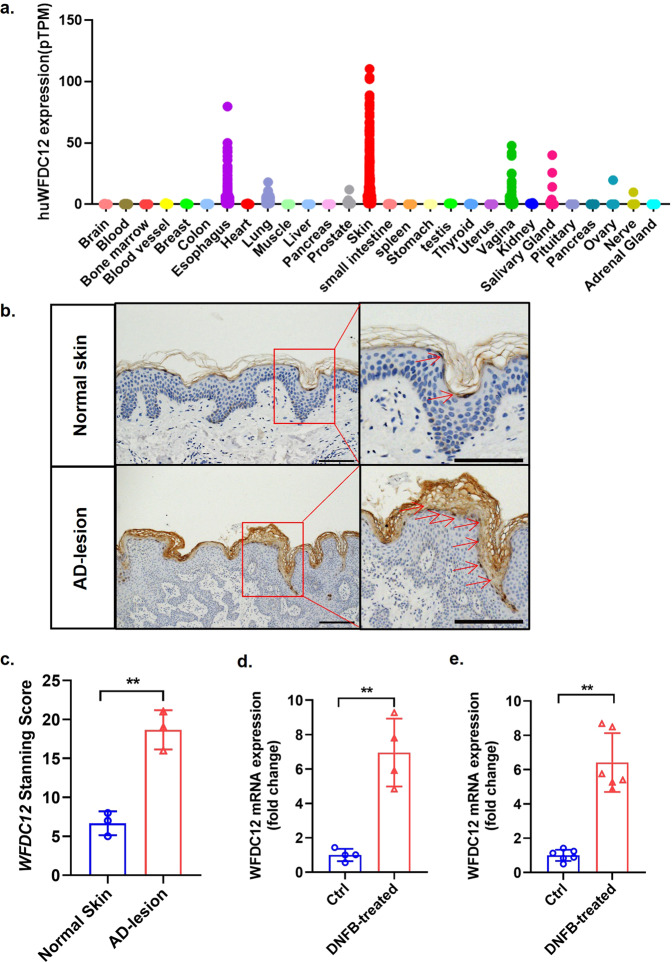
WFDC12 was closely related to the occurrence and progression of AD. **a** The relative expression of *WFDC12* in different tissues in normal people from *GTEx* database (*n* = 6–860) (*huWFDC12*, Human *WFDC12*). **b** Immunohistochemical staining of WFDC12 was performed in human normal skin (*n* = 3) and AD-lesion (*n* = 3). Scale bars: left figure. 200 μm; right figure. 100 μm. **c** The positive expression level of WFDC12 in immunohistochemistry staining. **d** RT-qPCR analysis of *WFDC12* mRNA level in mouse normal skin (*n* = 4) and DNFB-induced skin lesions (*n* = 4). **e** The transcriptional level of *WFDC12* in mouse normal skin (*n* = 6) and DNFB-induced skin lesions (*n* = 6) from GEO Datasets (GDS2820). Data were expressed as mean ± SD. Asterisks indicate statistical significance based on unpaired or paired *T*-test; **p < 0.01.

To explore the association between WFDC12 and AD, we first analyzed the mRNA levels of WFDC12 in different human tissues through the *GTEx* database. The results showed that the expression level of WFDC12 was the highest in the skin (Fig. 1a), indicating that WFDC12 might play an important role in the occurrence and development of skin-related diseases. Then, we examined WFDC12 protein expression level and distribution in the healthy human skin tissue and the skin lesions of AD patients by using immunohistochemical staining. WFDC12 was mainly located in the epidermis of the skin and was mainly expressed in the highly differentiated stratum corneous (Fig. 1b). Compared with normal skin tissue, WFDC12 expression was significantly increased in the skin tissue of AD patients (Fig. 1c), which is consistent with previous reports [26, 28]. 2,4-dinitrofluorobenzene (DNFB) treatment was performed on the dorsal skin of wild-type mice (WT) to construct the AD mouse model and the mRNA level of WFDC12 in the lesions was detected. The results showed that WFDC12 was significantly increased in the skin lesion of the modeled mice (Fig. 1d). In addition, we analyzed the level of WFDC12 in the *GDS2820* with normal mouse skin and DNFB-induced AD-like skin tissue. And we found that the mRNA level of WFDC12 in the skin lesion area of the modeled mice was higher (Fig. 1e), which was consistent with the results obtained in our experiments. Thus, WFDC12 is bound up with AD clinical features and may participate in the development of AD.

### The construction and characterization of skin homeostasis of specific epidermal K14-WFDC12-overexpressing mice

**Fig. 2:**
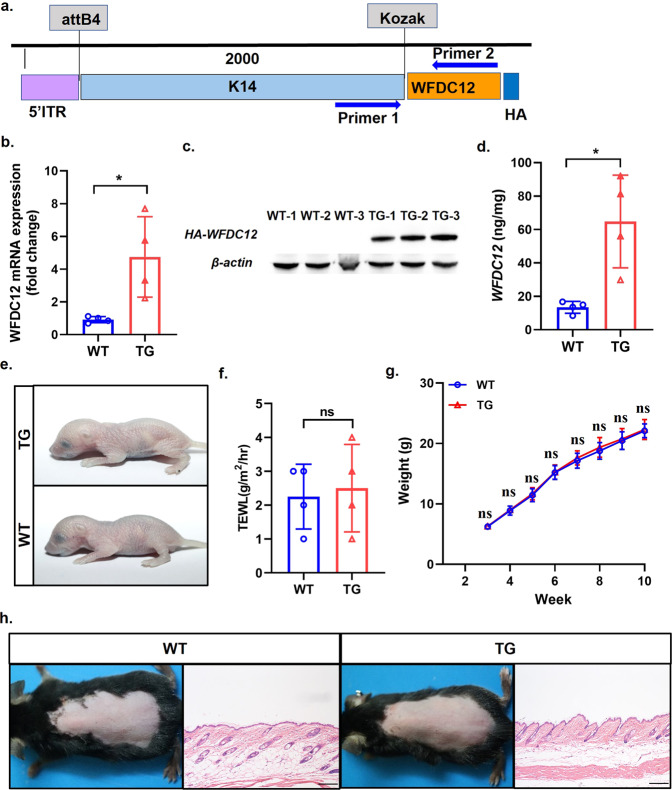
The transgenic mice with keratinocyte-specific overexpression of WFDC12 (K14-WFDC12) were successfully constructed and the overexpression of WFDC12 did not affect the skin homeostasis and survival of mice. **a** The construction schematic diagram of K14-WFDC12 plasmid. **b** RT-PCR analysis was used to detect WFDC12 mRNA levels in WT and TG mice. **c** The western blot results of dorsal skins of WT and TG by using anti-HA. **d** ELISA was used to detect the protein expression of WFDC12 in the skin of WT and TG mice. **e** The toluidine blue staining of fetus mice. **f** TWEL determination in fetus rats. **g** Statistical chart of weight change during mouse growth. **h** Direct view of 8-week-old mice dorsal skin after shaving and H&E staining of paraffin sections in skin stable state. Scale bar: 200 μm. Data were expressed as mean ± SD. Asterisks indicate statistical significance based on unpaired or paired *T*-tests. ‘ns’ indicates no statistical difference.

To further explore the exact role and underlying mechanism of WFDC12 in the development and progression of AD, we constructed K14-WFDC12-overexpressing transgenic mice (TG) with high specific WFDC12 expression in epidermis using K14 promoter (Fig. 2a). The identification of TG was proceeded by using PCR with K14-WFDC12 primers and gel electrophoresis. The length of PCR products is 551 bp in TG, while no electrophoretic band represents WT (Fig, S1a). To verify the stable expression of K14-WFDC12, we detected the mRNA level of WFDC12 by qRT-PCR, and the results showed that WFDC12 was highly expressed in TG (Figs. 2b and S1b). Meanwhile, the constructed TG carried the HA tag (Fig. 2a). Western blot was used to detect the HA-Tag, and HA expression was found in the TG but not in WT (Fig. 2c, Supplemental Material 2). In addition, we measured the expression of WFDC12 protein in mice by using ELISA and found that the WFDC12 content was higher in TG (Fig. 2d).

It has been reported that deletion or overexpression of epidermal protease or protease inhibitors might affect skin barrier function in the homeostasis in mice, causing spontaneous skin diseases and impairing the survival of mice [29]. To confirm whether the overexpression of WFDC12 caused the above changes, toluidine blue (TB) staining (Fig. 2e) and trans epidermal water loss (TEWL) (Fig. 2f) were taken from fetal mice about 3 days old to detect skin barrier function, and the results showed no difference between TG and WT. Secondly, we recorded the changes in body weight of mice during growth and found no significant difference in body weight between TG and WT mice (Fig. 2g). Furthermore, we observed the surface appearance and volume of mice aged about 8 weeks and took the back skin for histopathological Hematoxylin and Eosin (H&E) staining. The results showed that the two groups share no difference in the surface appearance (Fig. 2h) and thickness of the epidermis (Fig. S1c). Consistent with the above results, we found that overexpression of WFDC12 in HaCaT cells did not have significant effects on cell migration (Fig. S2a) and proliferation (Fig. S2b).

These results suggest that TG mice were successfully constructed, and the overexpression of WFDC12 in keratinocytes does not affect the natural physiological status of the skin barrier, body weight, appearance, and skin structure in mice in homeostasis.

### WFDC12-overexpressing exacerbated AD-like symptoms in DNFB-induced K14-WFDC12 transgenic mice

**Fig. 3:**
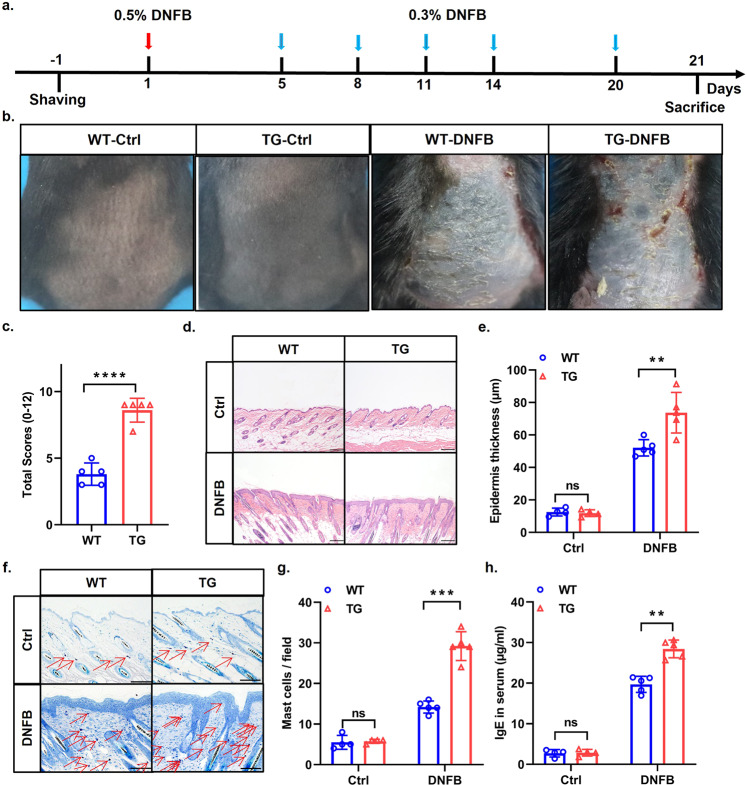
WFDC12-overexpressing exacerbated DNFB-induced AD-like symptoms in mice. **a** Established scheme of DNFB-induced AD mouse model. **b** Dorsal skin lesions after 21 days of modeling. **c** Total scores of dorsal skin lesions after 21 days of modeling (*n* = 5). **d** The H&E staining representative figure in skin lesions. Scale bar. 200 μm. **e** Quantitative analysis of epidermal thickness. Five visual field measurements were randomly selected for each sample (*n* = 4). **f** Toluidine blue staining representative images. scale bar 100 μm; Arrows indicate mast cells. **g** Statistical results of mast cells. Five visual field measurements were randomly selected for each sample (*n* = 4 or 5). **h** ELISA was used to detect serum IgE (*n* = 4 or 5). Data were expressed as mean ± SD. Asterisks indicate statistical significance based on unpaired or paired *T*-test; *p < 0.05, **p < 0.01, ***p < 0.001, ****p < 0.001, ‘ns’ indicates no statistical difference.

To study the function of WFDC12 in AD pathogenesis, an AD animal model was established by applying 0.5% DNFB on the back skin after being stimulated by 0.3% DNFB. The DNFB-induced scheme is shown in Fig. 3a. From the second day to the end of modeling, mice began to show varying degrees of erythema, edema, scab, ulceration, dryness, scales, and pigmentation in dorsal skin (Fig. 3b). We carried out a phenotypic analysis of the skin back tissues based on four AD-like symptoms including desquamation, erythema hemorrhage, epidermal damage or shedding and edema to evaluate the severity of illness in mice modeled for 21 days. The results showed that AD-like inflamed symptoms on the skin back of TG mice were more serious and the scores of skin back lesions of TG were obviously higher than that of WT mice (Figs. 3c and S2). Additionally, these epidermis cytokines such as IL-4 and IL-13 stimulate B lymphocytes to produce serum-specific IgE, so the increase of serum IgE is one of the markers of AD [18]. Therefore, both apparent symptoms and histopathological and hematological indicators are equally important for the assessment of the illness’s severity. Tissue and serum samples were obtained from modeled mice after 21 days modeling procedure. Paraffin-embedded skin tissue was sectioned for H&E and TB staining. Meanwhile, the skin thickness and mast cell infiltration in the skin were measured and the serum IgE was detected by ELISA. Compared to WT, the enhancive epidermal thickness (Fig. 3d, e), the accessorial number of dermal infiltration mast cells (Fig. 3f, g), and the increased contents of serum IgE were more remarkable (Fig. 3h). These results imply that AD-like inflamed lesions in TG are more serious than those in WT after induced by DNFB.

### WFDC12-overexpressing promoted the migration of antigen-presenting cells to lymph nodes and increased disease-related T cell differentiation in DNFB-induced mice

**Fig. 4:**
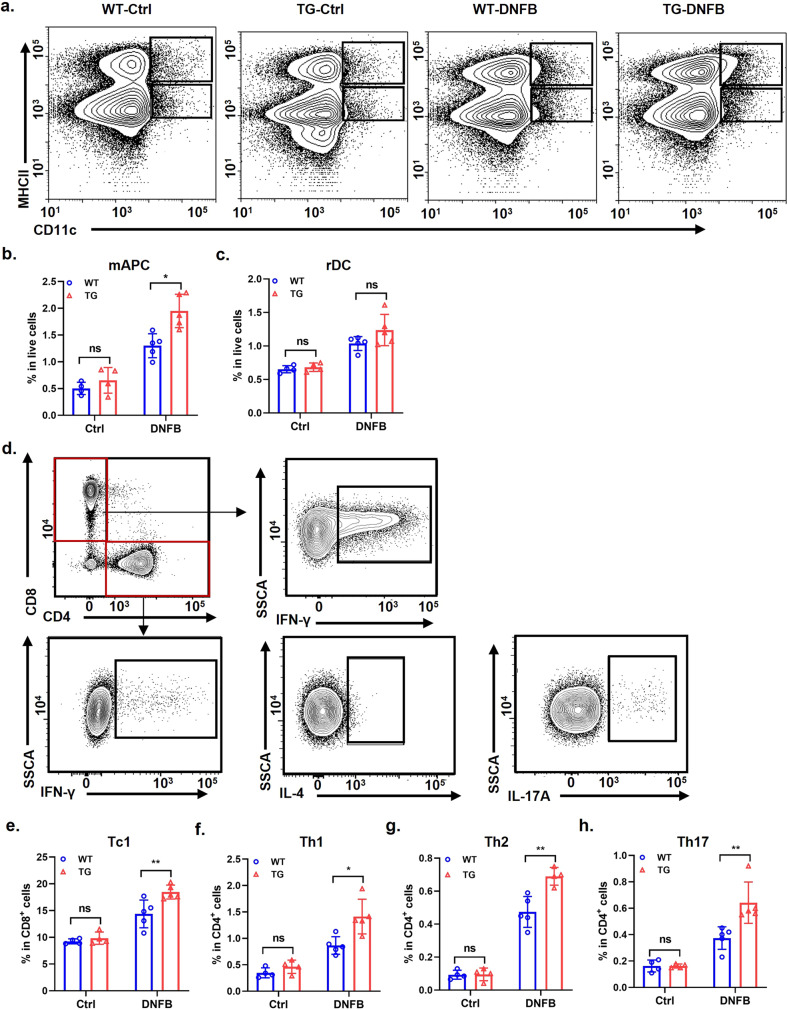
WFDC12-overexpressing facilitates the migration of mAPCs to lymph nodes and T cells differentiation in lymph nodes. **a** Analysis of mAPCs and rDCs in lymph nodes of WT and TG mice in control and after DNFB-induced. **b** and **c** The proportion of mAPC (**b**) and rDC (**c**) in the total number of live cells in mice (*n* = 4 or 5). **d** Flow cytometric analysis of T cell differentiation in mouse lymph nodes. **e**–**h** The ratio of Tc1 (**e**), Th1 (**f**), Th2 (**g**), and Th17 (**h**) in mouse lymph nodes. Data were expressed as mean ± SD. Asterisks indicate statistical significance based on unpaired or paired *T*-test; *p < 0.05, **p < 0.01, ***p < 0.001, ‘ns’ indicates no statistical difference.

The activation of T cells by DNFB mainly depends on the uptake of hapten complexes in the skin by migrant antigen-presenting cells (mAPCs) and their migration to lymph nodes to activate T cells [30]. The flow capability of mAPCs which flow from the epidermis to lymph nodes keeps in a steady state during normal homeostatic conditions, while it is enhanced in infected or inflamed skin [31]. Therefore, we first detected the infiltration of mAPCs migrating from the epidermis in mice lymph nodes (Fig. 4a). The results showed that there was no significant difference in mAPC and rDC infiltration in lymph nodes between solvent control WT mice (WT-Ctrl) and solvent control K14-WFDC12 mice (TG-Ctrl) (Fig. 4b, c). Compared with the control group (WT-Ctrl and TG-Ctrl), the infiltration of mAPC in DNFB-induced mice (WT-DNFB and TG-DNFB) lymph nodes increased significantly (Fig. 4b). Moreover, compared with WT-DNFB group mice, K14-WFDC12 mice treated with DNFB showed more mAPC infiltration, while no significant difference was found in rDC infiltration (Fig. 4b, c).

Next, we investigated the differentiation of T cells in the inguinal lymph nodes of control and DNFB-induced mice (Fig. 4d). The results showed that there was no difference in the infiltration proportion of various cells between the solvent control group WT-DNFB and TG-DNFB mice (Fig. 4e–h). After DNFB induction, the Th1 (Fig. 4e) and Tc1 (Fig. 4f) cells of lymph node endocrine IFN-γ in TG-DNFB mice were significantly higher than those in WT-DNFB mice. The infiltration of Th2 (Fig. 4g) and Th17 (Fig. 4h) cells secreting IL-4/IL17 in TG-DNFB mice was also higher than those of WT-DNFB mice.

These results indicate that specific WFDC12-overexpressing in the epidermis may accelerate the differentiation of Th cells in lymph nodes by promoting the migration of mAPCs from the skin to lymph nodes.

### WFDC12-overexpressing enhanced immune response in epidermis in DNFB-induced mice

**Fig. 5:**
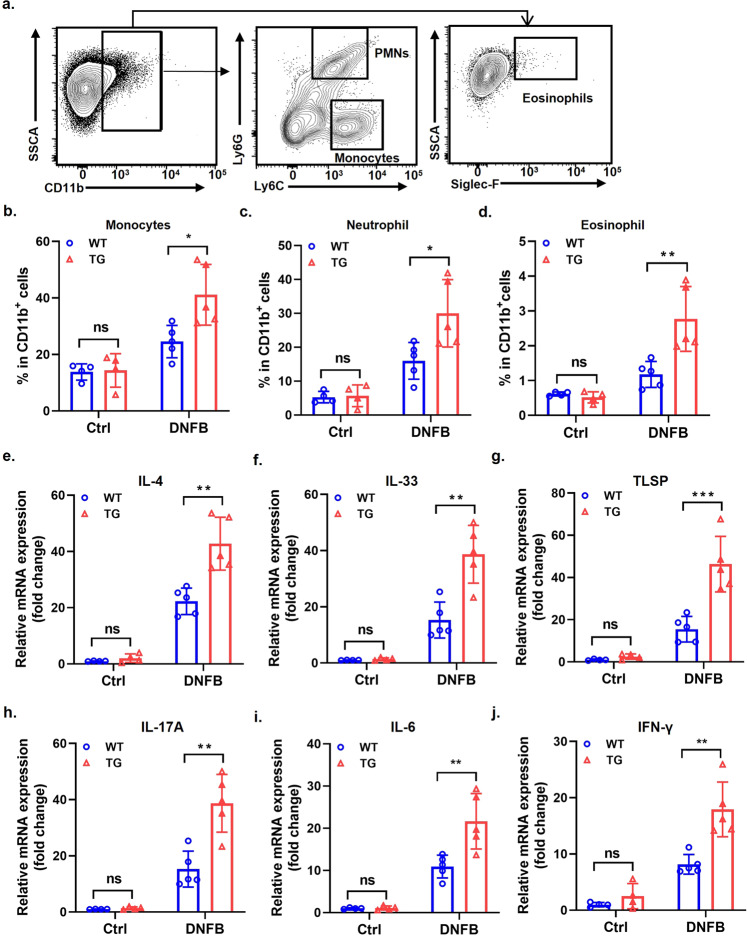
WFDC12-overexpressing enhanced immune responses in mice epidermis. **a** Flow cytometric analysis of bone marrow cells (BMCs) in mice epidermis. **b**–**d** The ratio of monocytes (**b**). neutrophil (**c**) and eosinophil (**d**) in CD11b^+^ cells in mice epidermis (*n* = 4 or 5). **e**–**j** RT-qPCR analysis of IL-4 (**e**), IL-33 (**f**), TLSP (**g**), IL-17A (**h**), IL-6 (**i**). and IFN-γ (**j**) (*n* = 4 or 5). Data were expressed as mean ± SD. Asterisks indicate statistical significance based on unpaired or paired *T*-test; *p < 0.05, **p < 0.01, ***p < 0.001, ‘ns’ indicates no statistical difference.

In addition to T cells, many bones marrow-derived immune cells are also involved in the occurrence and development of AD. And they can be recruited by chemokines and secrete reciprocal cytokines, proteases, and other bioactive media to regulate skin immune microcirculation [32, 33]. Moreover, the immune cells infiltrating the skin lesions can regulate each other in a cytokine–cytokine receptor manner [34]. On the one hand, we examined the infiltration of bone marrow cells in the epidermis by flow cytometry (Fig. 5a). It was found that in the control groups, the infiltration of monocytes (Fig. 5b), neutrophils (Fig. 5c) and eosinophils (Fig. 5d) in the skin lesions of WT and TG were not significantly different. After DNFB induction, the infiltration of monocytes (Fig. 5b), neutrophils (Fig. 5c), and eosinophils (Fig. 5d) in the skin lesion area of TG-DNFB were significantly higher than that of WT-DNFB. On the other hand, the expression of related cytokines was detected by qRT-PCR. The results showed that the expression of cytokines such as IL-33, TSLP, and IFN-γ in TG-DNFB were higher than those of in WT-DNFB (Fig. 5e). Interestingly, the transcriptome sequencing data showed that the expression of various cytokines (such as CXCL9/10 related to Th1), chemokines (CCL19/21 related to DC cell migration) and their receptors in TG mice were higher than WT mice (Fig. S4). These results suggest that epidermal-specific overexpression of WFDC12 promotes the infiltration of immune cells and the expression of related cytokines and chemokines, strengthening the epidermal immune inflammatory response.

### WFDC12 regulated arachidonic acid lipoxygenase metabolism pathway in modeled K14-WFDC12 mice

**Fig. 6:**
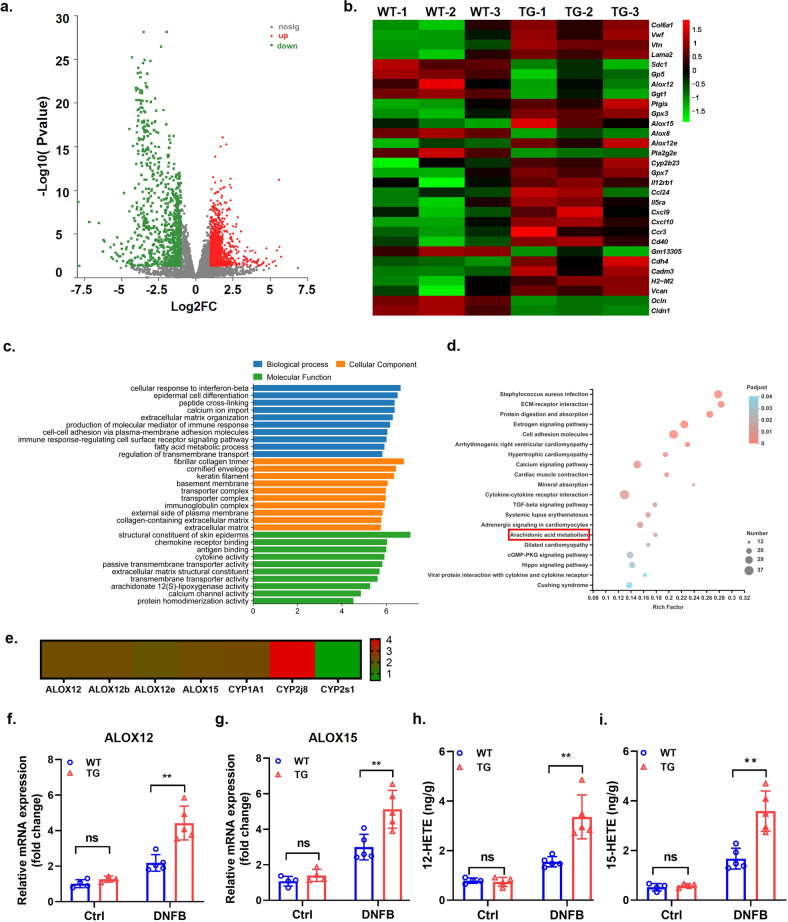
WFDC12-overexpressing regulates the metabolic pathways of arachidonic acid lipoxygenase in the epidermis of modeled mice. **a** Volcano map of DEGs in WT-DNFB/ TG-DNFB. DEGs were more than 2 times and FDR ≤ 0.05. The abscissa represents the fold change of gene expression in each group. The ordinate represents the statistical difference in gene expression. Red dots and blue dots represent upregulated genes and downregulated genes in WT-DNFB/ TG-DNFB, respectively. Gray dots indicate the indifference genes. **b** Heatmaps showing the representative 30 DEGs in WT-DNFB/ TG-DNFB. **c** DEGs in TG-DNFB and WT-DNFB were clustered in the term of biological process. subcellular localization and molecular function. **d** KEGG Pathway enrichment analysis results. The results were arranged according to the *p* value from the smallest to the largest. and 10 items were selected for drawing. **e** The fold changes of DEGs were gathered in the AA pathway in DNFB-induced mice. **f** and **g** RT-PCR analysis of the relative expression of ALOX12 (**f**) and ALOX15 (**g**) in the epidermis of mice (*n* = 4 or 5). **h** and **i** ELISA analysis of 12-HETE (**h**) and 15-HETE (**i**) in the epidermis of mice (*n* = 4 or 5). Data were expressed as mean ± SD. Asterisks indicate statistical significance based on unpaired or paired *T*-test; **p < 0.01. ‘ns’ indicates no statistical difference.

The above studies showed that WFDC12-overexpressing promoted the migration of mouse epidermal APCs to lymph nodes, promoted the differentiation of T cells in lymph nodes related to disease progression, and affected the infiltration of immune cells in the immune microenvironment of mouse skin lesions. Next, we evaluated the gene expression profile in mouse epidermis after DNFB induction by RNA-seq analysis to investigate the mechanism of WFDC12 accelerating AD-like skin inflammation in mice. Among 2004 differentially expressed genes (DEGs) in WT-DNFB and TG-DNFB, 1117 and 887 genes were significantly upregulated and downregulated, respectively (Fig. 6a, Supplementary Table S2). The heatmap displayed 30 representative DEGs (Fig. 6b). GO enrichment analysis found that DEGs were mainly enriched in pathways which contained responding to interferon-β (IFN-β), epidermal cell differentiation, peptide cross-linking, fatty acid metabolism and antigen receptor mediated pathways (Fig. 6c). At the molecular function level, DEGs were primarily enriched in terms of chemokine activity, cytokine activity and extracellular matrix (ECM) mechanism composition (Fig. 6c). In addition, we found that the signaling pathways with significant statistical differences chiefly included *Staphylococcus aureus* infection, extracellular matrix–receptor interaction, arachidonic acid (AA) signaling pathway, and cytokine–cytokine receptor interaction receptor signaling pathway (Fig. 6d). Combined with the analysis of DEGs in WT mice before and after DNFB induction (Fig. S5) and previous reports [11, 35], we hypothesized that the mechanism of WFDC12 regulating skin immunity may be closely related to the AA metabolism pathway. Subsequently, we found significant changes in the expression of genes involved in the lipoxygenase pathway and cytochrome P450 pathway in TG-DNFB (Fig. 6e). RT-qPCR was used to verify the transcriptome results, and it was found that the expression of arachidonate 12-lipoxygenase (*ALOX12*) and arachidonate 15-lipoxygenase (*ALOX15*) in the dorsal skin of TG-DNFB was up-regulated (Fig. 6f, g). In addition, we found that *ALOX12/15* silencing has no significant effect on the migration (Fig. S6a) and proliferation (Fig. S6b) of mouse keratinocyte PAM212. It has been reported that ALOX12 and ALOX15 are crucial lipid-metabolizing enzymes that enhance AA metabolism in AD lesions [33, 36, 37]. Their metabolites, 12-hydroxyeicosatetraenoic acid (12-HETE) and 15-hydroxyeicosatetraenoic acid (15-HETE) exert proinflammatory effects by promoting chemokine expression [38–40] and immune cell infiltration [41, 42]. We found that the contents of 12-HETE and 15-HETE in the skin lesion area of TG-DNFB were higher than those in WT-DNFB by using ELISA (Fig. 6h, i).

These results suggest that keratinocyte-specific high expression of WFDC12 may up-regulate the expression of ALOX12 and ALOX15 and promote the accumulation of inflammatory mediators 12-HETE and 15-HETE of AA metabolites by activating the lipoxygenase AlOX12/15 pathway in the epidermal AA metabolic pathway, thus aggravating those AD symptoms induced by DNFB in mice.

### WFDC12 affected the activity of epidermal serine hydrolase

**Fig. 7:**
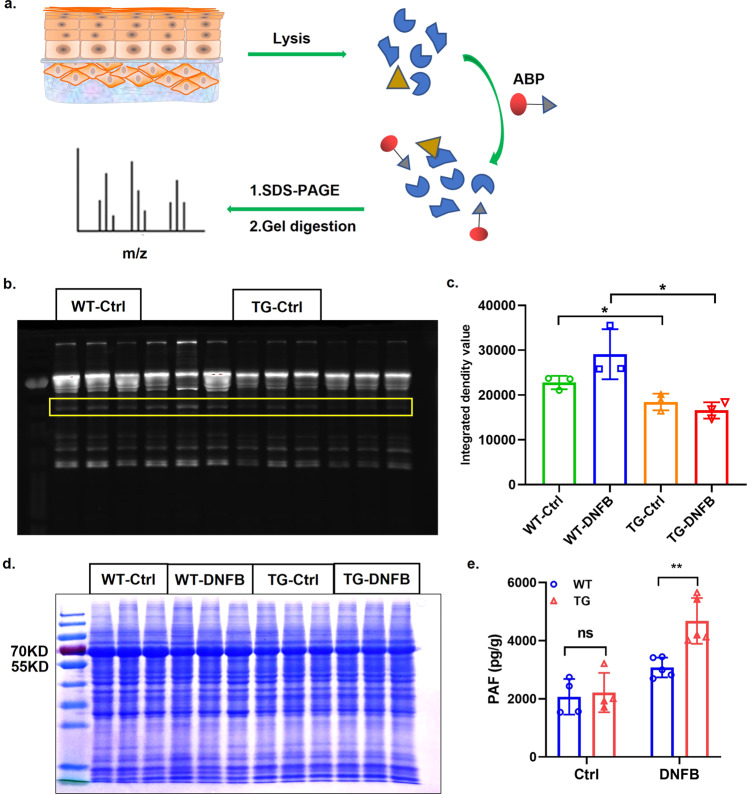
WFDC12-expressing accelerates the activity of serine hydrolase strengthen and the accumulation of PAF. **a** Schematic diagram of enzyme activity detection with the probe with serine hydrolase binding activity. ABP activity-based probe. **b** The gel fluorescence imaging of serine protease activity in mice epidermis. **c** Quantitative analysis of serine protease activity. **d** Coomassie brilliant blue staining analysis of proteins in the epidermis. **e** The content of PAF in mice epidermis.

In the previous sections, we found that WFDC12 may regulate AD immune inflammation by affecting the metabolism of lipoxygenase ALOX12/15 in the AA metabolic pathway and promoting the production and accumulation of inflammatory mediators 12/15-HETE. Next, a specific fluorescence probe and mass spectrometry (MS) were used to explore the functional substrate of WFDC12 as an enzyme inhibitor. The experimental technical process was shown in Fig. 7a. Mice back skin tissue protein incubated with the activity-based probe (ABP) with specific serine hydrolase activity sites and fluorescent report groups. Electrophoresis was performed after the reaction, then imaging with a fluorescent chemiluminescence imager and evaluating the enzyme activity by fluorescence intensity (Fig. 7b). The results showed that the serine protease activity in TG mice was significantly lower than that in WT mice, whether DNFB-induced or not (Fig. 7c). Moreover, total proteins were stained using Coomassie brilliant blue after SDS–PAGE electrophoresis (Fig. 7d).

To further identify the enzyme whose activity was inhibited by WFDC12, we cut the differential bands for mass spectrometry identification by comparing the fluorescence imaging and the Coomassie brilliant blue staining. The results showed that the differential bands contained 9331 peptides belonging to 988 proteins, of which 13 had serine protease activity. Further analysis revealed that 3 serine hydrolases of the 13 were related to the regulation of the AA metabolic pathway mentioned above, namely Cytochrome P450 protein 4F14 (CYP 4F14), and the subtype of platelet-activating factor acetylhydrolase (PAF-AH) PAF-AHIBI and PAF-AH2 (Supplementary Table S3). CYP4F14, also known as leukotriene-B4 ω-hydroxylase 3, inactivates the inflammatory medium leukotriene-B4 (LTB4) which is the metabolons of AA in lipoxygenase 5 pathway by hydroxylation or carboxylation [43]. PAF-AH is a hydrolase of PAF and inactivates PAF by catalyzing the hydrolysis of acetyl esters at the SN-2 site of PAF [44]. PAF is a pro-inflammatory lipid medium of glycerophosphoric choline expressed in a variety of immune cells, which activates immune cells by binding to receptors and is widely involved in the regulation of various allergic diseases such as asthma and AD [45–47]. Therefore, we used ELISA to detect the content of PAF in the skin of the modeled mice. The results showed that the PAF content in K14-WFDC12 mice was significantly higher than that in WT mice (Fig. 7e).

In conclusion, WFDC12 may promote the accumulation of PAF by regulating enzyme activity and activating the lipoxygenase pathway of AA metabolism to promote the production of inflammatory lipid mediators, thus participating in the immune inflammatory response in AD pathogenesis and regulating AD progression.

## Discussion

AD is a chronic inflammatory skin disease with complex etiology [48]. Existing clinical treatment methods or programs have obvious side effects and AD cannot be cured, seriously affecting patients’ physical and mental health and quality of life [3]. Proteases and protease inhibitors inside the human body skin widely participate in controlling the expression of growth factor, chemokines, cytokines and lipid active molecules, the activation of signal receptors and adhesion molecules, the regulation of ion channels, and the remodeling of extracellular matrix and so on all sorts of biological process, gradually become important regulatory factors in physiological and pathological skin environment.

*WFDC12* is located on human chromosome 20 (20q13) and encodes the epidermal endogenous serine protease inhibitor WFDC12 which belongs to the WFDC family involved in both innate and adaptive immunity extensively [49]. WFDC12 has been reported to be highly expressed in the skin and was upregulated in the lesions of AD patients, but its precise role and concerned mechanism in AD have not been studied yet. In this study, we systematically explored the role and possible mechanism of WFDC12 in AD progression with the DNFB-induced mouse model.

In our study, we found that WFDC12 was up-regulated in AD patients and DNFB-induced AD mice models through public databases and related confirmatory experiments, and preliminarily clarified the clinical relevance of its expression with AD. In the inflammatory state, those cytokines such as IL-17A and TNF-α in epidermal keratinocytes promote the transcription and expression of transcription factor AP-1 and enhance its binding to the AP-1 binding site in the upstream region of WFDC12, leading to the up-regulation of WFDC12 expressions [50]. This may be one of the mechanisms contributing to the high expression of WFDC12 in AD lesions. It has been reported that deletion or overexpression of proteases and/or their inhibitors in the epidermis may affect epidermal development and skin barrier in mice, resulting in impaired survival status in mice [16–19]. However, no skin barrier damage and spontaneous skin inflammation were observed in K14-initiated transgenic mice with epidermal-specific high expression of WFDC12, and overexpression of WFDC12 did not affect the proliferation and migration of HaCaT cells, suggesting that overexpression of WFDC12 had no significant effect on the development of mice skin homeostasis and the biological behavior of keratinocytes. Therefore, we surmised that there might be a corresponding alternative mechanism for the protease and protease inhibitor interaction network to maintain skin homeostasis and ensure the normal function of the skin barrier. When human skin is exposed to external stimulus in the inflammatory state, keratinocytes are activated, immune cells are infiltrated in large quantities, and protease hydrolysis activity is dysregulated. We supposed that WFDC12 plays a momentous role in these above processes.

We used DNFB to smear dorsal skin to establish an AD mice model and found that the modeled K14-WFDC12 transgenic mice showed more serious desquamation, deeper epidermal thickening, increased mast cell dermal infiltration in the skin lesions and increased serum IgE content, and other pathological features of AD. In addition to being absorbed by epidermal resident APCs and delivered to lymph nodes, DNFB also activates keratinocytes in the epidermis, resulting in the production of cytokines and chemokines [51]. Therefore, flow cytometry showed that the proportion of specific CD11c + MHCII^high^ that migrated from skin to lymph node was significantly increased in TG, while there was no significant difference in the proportion of rDCs. APCs migrate to lymph nodes and activate the differentiation of undifferentiated T cells into effector T cells to secrete cytokines. The differentiation of Tc1, Th1, Th2, and Th17 cells in the lymph nodes of K14-WFDC12 transgenic mice was significantly increased (the proportion was significantly higher than WT). T cells migrating to the epidermis produce many cytokines, while keratinocytes, DC cells, and macrophages inherent in the skin also secrete chemokines and cytokines, such as CXCL9 and CXCL19, which further recruit monocytes, neutrophils, and eosinophils and other immune cells to infiltrate the inflammatory site. In K14-WFDC12 mice, the infiltration of monocytes, neutrophils, and eosinophils was increased in the skin, and the expressions of inflammatory factors such as IL-4, IL-33, CXCL9, and TSLP were significantly up-regulated. The foregoing data show that skin-specific overexpression of WFDC12 may facilitate the migration of APCs with the ability to migrate to lymph nodes and enhance the differentiation of T cells, taking part in the regulation of the skin immune microenvironment.

Transcriptome sequencing is an important research method to study pathological mechanisms. In this paper, we took mouse back skin tissue for transcriptome sequencing. Consistent with the above experimental results, the Th1/Th2 differentiation-related signaling pathway and IL-17 signaling pathway were activated in the mice after modeling. Meanwhile, the PPAR signaling pathway related to lipid metabolism was also activated in the skin after DNFB induction, indicating that lipid metabolism may play an indispensable role in the DNFB-induced AD model. What’s more, these DEGs were mainly concentrated in pathways of cytokines, ECM, and lipid metabolism after WFDC12-overexpressing. Transcriptomic and lipidomic analysis of lesions and non-lesions in AD patients revealed that AA metabolism was abnormal in the lesions of AD patients [39]. Under normal physiological conditions, AA binds to cell membranes to form supporting complex membrane phospholipids. AA was hydrolyzed off the membrane with the help of phospholipases when cells received external stimulators and AA metabolism is activated. There are three main pathways of AA metabolism: epoxidase pathway (COXs pathway), lipoxygenases pathway (LOXs pathway), and cytochromes pathway (CYPs pathway). AA metabolism pathways contain a wide variety of enzymes, and their metabolites play important physiological regulatory functions in inflammation, fever, blood pressure, reproductive system, respiratory system, etc. Therefore, the AA metabolic pathway in this KEGG enrichment result attracted our attention. We then conducted a validation test. In DNFB-induced K14-WFDC12 transgenic mice, the expression of lipoxygenases ALOX12 and ALOX15 in the AA metabolic pathway was upregulated significantly. Some studies have shown that 12/15-LOX and their derivatives function in epithelial inflammatory diseases and epithelial injury repair [52]. ALOX15-deficient mice showed a deficiency of ALOX15-derived lipoxin A4 (LXA4) and neuroprotectin D1 (NPD1), as well as defects in corneal re-epithelialization and neutrophil recruitment. But in our study, ALOX12/15 silencing in mouse PAM212 keratinocytes did not affect the ability of proliferation and migration (Fig. S6). In studies of lipid composition in keratinocyte-derived human epidermal equivalents from AD patients, 12/15-LOX-derived products 12/15-HETE were found to be the major metabolite of the lipoxygenase pathway in AD pathogenesis [39]. In our study, the expression of ALOX15/12 was up-regulated in the skin of K14-WFDC12 mice, and the content of major metabolite 12/15-HETE was also clearly increased. 12/15-HETE has been shown to be an active mediator of lipids exerting proinflammatory effects. In conclusion, we hypothesized that WFDC12 may regulate the process of the skin immune microenvironment by up-regulating the metabolic pathway of AA lipoxygenase.

WFDC12 is a member of the serine protease suppressor family, and the recombinant WFDC12 protein has inhibitory effects on kallikrein 7 (KLK7) and human neutrophil elastase (HNE) in vitro [26]. KLK7 is specifically highly expressed in the epidermis and is mainly associated with the process of cuticle desquamate and some studies have shown that the absence of WFDC12 has no effect on AD-like inflammation [53]. HNE acts a proinflammatory role in most inflammatory diseases, so existing reports on WFDC12 substrate do not well explain our findings. Based on the above study, we hypothesized that WFDC12 has other functional substrates to activate the lipoxygenase ALOX12/15 pathway of AA to function in a pro-inflammatory role in the more complex physiological environment in vivo and then tested it. CYP4F14 and PAF-AH were found to regulate AA metabolism. The former regulates the downstream pathway of AA metabolism and metabolizes and inactivates the proinflammatory mediator LTB4 which is the product of AA metabolism by lipoxygenase 5 (ALOX5) [54]. PAF-AH is the only enzyme that can regulate the hydrolysis of PAF. Once PAF-AH is disabled or inhibited, PAF accumulates in the body. PAF is a proinflammatory lipid mediator and functions in many inflammatory diseases by binding to the receptor PAF-R. In the skin, PAF regulates the expression of cytokines such as IL-8, IL-6, and IL-1β in keratinocytes by binding to PAF-R in the keratinocyte epidermis. Based on the above, we demonstrate a potential molecular mechanism by which WFDC12 promotes DNFB-induced AD inflammation by regulating AA metabolic pathways and related enzyme activities (Fig. S7). When the skin is exposed to external irritation, membrane phospholipids are broken down into LYSO-PAF (precursor of PAF) and free AA under the action of phospholipase A2 [55]. On the one hand, LYSO-PAF is metabolized into PAF and enters the cell via a receptor (PAF-R). WFDC12 inactivates PAF-AH to make the accumulation of PAF in cells rapidly and to stimulate the augment of chemokines and cytokines, thus causing enhanced inflammatory response. On the other hand, free AA increased and AA metabolism in cells was strengthened. In the LOXs pathway, AA can be metabolized by ALOX5 to LTB4 (a potent chemotactic lipid mediator that can be inactivated by CYP 4F14) or directly metabolized by ALOX12/15 to 12/15-HETE [11]. WFDC12 inhibits the activity of CYP 4F14, which can lead to the increase of LTB4 and enhance the secretion of various factors. Meanwhile, CYP 4F14 was inactivated in the AA CYPs pathway, which enhanced the metabolism of the ALOX12/15 pathway, resulting in the increase of 12/15-HETE and further aggravation of AD symptoms. Taken together, WFDC12 may activate the ALOX12/15 pathway in AA metabolism by affecting the activity of epidermal PAF-AH and CYP 4F14, thus promoting the accumulation of relevant inflammatory lipid mediators and participating in the regulation of AD pathogenesis and progression.

It is worth mentioning that we did not construct WFDC12-knockout mice and perform corresponding verification and explore whether WFDC12 function loss has a protective effect on DNFB induction, which may be an important aspect of our future experiments. In addition, in our current experimental designs, direct interactions between WFDC12 and 3 potential interacting substrates CYP 4F14, PAF-AHIBI (encoding PAF-AHIβ subunit), and PAF-AH2 (single subunit polypeptide) were not explored. This will be the focus of our subsequent work to identify the molecules that play an exact or major role in the overall mechanism.

In summary, our study found that the specific high expression of WFDC12 in epidermal keratinocytes plays an irreplaceable role in promoting the development of AD by regulating AA metabolism. Meanwhile, we combined these research means in cell biology, transcriptomics, and protease activity studies to investigate the related mechanism of WFDC12 in regulating the pathogenesis of AD, which provides new insights and theoretical basis for the elucidation of the pathogenesis, new diagnosis, and treatment methods and the search for potent drug targets of AD.

## Materials and methods

### Animals

The K14-WFDC12 transgenic mice were commissioned to construct by *Saiye Biotechnology Co., Ltd*. C57BL/6 mice (Wild-type, WT) used for breeding were purchased according to the policies and agreements approved by Sichuan University. Sterile house condition, 12 h light/12 h dark cycle, the temperature of 25 ± 1 °C, and free access to water and food. The experiments were carried out following the National Institutes of Health’s ethical guidelines for the care and use of laboratory animals and the International Association for the Study of Pain (IASP). Tail biopsies of mice were collected and used to identify genotypes by PCR with the Mouse Direct PCR Kit (*Bimake*, B40015). The primers employed in PCR are shown in Supplementary Table S1. All experimental procedures were performed following the guidelines of experimental animals from Sichuan University.

### Human subjects

Biopsies of the lesional skin of 3 AD patients and the healthy skin of 3 donors were taken from the West China Hospital of Sichuan University. This study was conducted by the principles of the Helsinki Declaration and was approved by the ethics committee of West China Hospital of Sichuan University (Chengdu, Sichuan, China).

### Cell lines

The human keratinocyte cell line HaCaT was obtained from the China Center for Type Culture Collection (0106) and the mouse keratinocyte cell line PAM212 was purchased from the *Mingzhoubio* (MZ-2610). HaCaT and PAM212 cells were cultured in Dulbecco’s modified Eagle’s medium (DMEM; Thermo Fisher Scientific, C11995500BT) and Roswell Park Memorial Institute 1640 medium (RPMI-1640; Gibco, 11875119), respectively. And both mediums were supplemented with 10% (v:v) fetal bovine serum (FBS; Thermo Fisher Scientific, 10099141), 100 U/mL penicillin G, and 0.1 mg/mL streptomycin sulfate (Thermo Fisher Scientific, 15140122). The cell culture conditions were 37 °C and 5% CO_2_. All cells were found to be free from mycoplasma contamination.

### DNFB-induced AD-like skin inflammation model

The treatment of each group was as follows: The female mice (aged 8 weeks, 17–18 g) were sorted out to construct a DNFB-induced AD model. Before induction of DNFB, the back skin of mice was shaved and exposed with an approximate area of 2 × 3 cm. The solvent was prepared with acetone (*Sinopharm Chemical Reagent Co., Ltd*,10000418) and olive oil (*Sigma-Aldrich*, O1514) at a volume ratio of 3:1, and a certain amount of DNFB (*Sigma-Aldrich*, D1529) was added to prepare 0.5% and 0.3% DNFB solutions. For DNFB induction treatment, 150 μL 0.5% DNFB solution was applied on the first day for sensitization, and 120 μL 0.3% DNFB solution was applied on the fifth, eighth, 11th, 14th, 17th, and 20th days to induce AD-like inflammation. In the control group, the solvent was applied at the corresponding time points. The control group applied the same amount of solvent at the same time point. During the modeling process, the following four symptoms were evaluated: desquamation, erythema bleeding, epidermal damage or shedding, and edema [28]. Here we refer to the scoring standards for AD scores in mice, as follows: the degree of the scale, the degree of erythema, and the degree of thickening of the dorsal skin; each factor is independent in the range of 0–3. Score (0—none, 1—slight, 2—moderate, 3—maximum, and recorded every 24 h). The total score ranges from 0 to 12.

### RNA isolation, cDNA synthesis, and reverse transcription-quantitative polymerase chain reaction (RT-qPCR)

Mice skin tissues were ground in liquid nitrogen and used for RNA extraction with TRIzol (Invitrogen; Thermo Fisher Scientific, Inc.) according to the manufacturer’s protocol. Total RNA (2 µg) was reverse transcribed into cDNA using the PrimeScript RT reagent kit with gDNA Eraser (Takara Bio, Inc., Otsu, Japan) at 42 °C for 50 min and 85 °C for 5 min according to the instructions. cDNA (20 ng) was subjected to qPCR analysis with TB Green™ Premix Ex Taq™ II (Tli RNaseH Plus; Takara Bio, Inc.). *β-actin* was used as the internal gene and quantification were performed using the 2^−ΔΔCt^ method. The primers employed in RT-qPCR are shown in Supplementary Table S2.

### Western blotting

The samples derived from skin tissue were lysed, separated by electrophoresis on SDS–PAGE gels (*Beyotime Institute of Biotechnology*, P0012AC), and transferred to polyvinylidene fluoride (PVDF) membranes (*MerckMinipore*, IPVH00010). For western blotting detection, the proteins were incubated overnight with the following primary antibody: HA (*Cell Signaling Technology*, 1:1000 dilution), incubated overnight. Labeling of the primary antibodies was detected using goat anti-rabbit antibody conjugated to horseradish peroxidase (HRP) (*Invitrogen*, 2215587, 1:10,000 dilution), and further detected using ECL reagents (*MerckMinipore*, WBULS0500). ImageJ was used for further quantification of the band intensities in the images, and only the band intensities in the linear range were included.

### Hematoxylin and eosin (H&E) staining, microscopy, and image analysis

Human skin and mouse dorsal skin were fixed in 4% paraformaldehyde in PBS, embedded in paraffin, sectioned, and stained with H&E for histopathologic examination. Images were captured using an Olympus BX600 microscope (Olympus Corporation, Tokyo, Japan) and SPOT Flex camera (Olympus Corporation, Tokyo, Japan) and were analyzed with ImagePro Plus (version 6.0, Media Cybernetics) software. The epithelial thickness and infiltrating cells were evaluated in independent regions. For the measurement of skin thickness, 5 visual fields, and 5–10 measuring points were selected for each back film, and the average value was taken.

### Immunohistochemistry, microscopy, and image analysis

Human skin were tissues fixed in 4% paraformaldehyde in PBS, and the fixed sections were incubated in 3% H_2_O_2_ solution in PBS at room temperature for 10 min. Antigen retrieval was performed in sodium citrate buffer (0.01 M, pH 6.0) in a microwave oven at 1000 W for 3 min. Nonspecific antibody binding was blocked by incubation with 5% normal goat serum in PBS for 1 h at room temperature. Slides were stained overnight at 4 °C with the following primary antibodies: WFDC12 (*Proteintech*, 25101-1-AP; 1:500 dilution). The slides were subsequently washed and incubated with biotin-conjugated secondary antibodies for 30 min, and then with Horseradish Peroxidase Streptavidin (HRP Streptavidin) for 30 min (SPlink Detection Kits; ZSGB-BIO, SP-9001 or SP-9002). The sections were developed using the 3,3ʹ-diaminobenzidine (DAB) substrate kit (ZSGB-BIO, ZLI-9017) and counterstained with hematoxylin. Images were captured using an Olympus BX600 microscope and SPOT Flex camera. ImagePro Plus was used for further quantification of the DAB intensity.

### Toluidine blue staining, microscopy, and image analysis

Newborn mice were sacrificed and dehydrated by sequential incubation in 25%, 50%, 75%, and 100% methanol. After rehydration in PBS, they were incubated for 10 min in 0.01% toluidine blue and detained with PBS. Mice dorsal skin was fixed in 4% paraformaldehyde in PBS, embedded in paraffin, sectioned, and stained with 0.5% toluidine blue for 25 min. 0.5% acetic acid was dropped for cytoplasmic separation, and sections were sealed with neutral gum. Each step should be cleaned with distilled water. Images were captured using an Olympus BX600 microscope (Olympus Corporation, Tokyo, Japan) and SPOT Flex camera (Olympus Corporation, Tokyo, Japan) and were analyzed with ImagePro Plus (version 6.0, Media Cybernetics) software. Infiltrating cells were evaluated in independent regions, 4-5 visual fields were selected for each back film, and the average value was taken.

### Enzyme-linked immunosorbent assay (ELISA)

QuantiCyto® Mouse IgE ELISA Kit (*NeoBioscience*, EMC117.96) was used to detect the levels of serum IgE in mice. Mouse Wfdc12 (Single WAP motif protein 2) ELISA Kit (*Wuhan Fine Biotech Co., Ltd*., EM2019) was used to detect the levels of WFDC12 in mice epidermis. And 12-HETE (12-Hydroxyeicosatetraenoic Acid) ELISA Kit (*Wuhan Fine Biotech Co., Ltd*., EU3131), and 15-HETE (15-hydroxyeicosatetraenoic acid) ELISA Kit (*Wuhan Fine Biotech Co., Ltd*., EU2612) were used to detect the level of 12-HETE and 15-HETE, respectively. All assays were performed according to the manufacturer’s instructions.

### Flow cytometry

To obtain single-cell suspension from dorsal skin, samples were removed from the subcutaneous fat and mucosal tissue with the scalpel and spread into a Petri dish. The samples were then incubated in 2.4 U/mL dispase II overnight at 4 °C and then immersed in DMEM containing 50% (v:v) FBS to inactivate the dispase II. Gently scrape off the epidermal layer and add 5 mL of 0.25% EDTA-free trypsin (*Thermo Fisher*, 15050057) to the tube to obtain a single cell suspension, after digestion at 37 °C for 20 min. Finally, neutralized with 5 mL DMEM medium (*GbicoTM*, LS11995065). Single-cell suspension of epidermis cells was made followed by mechanical dissociation with a gentle MACS dissociator (Miltenyi Biotech, Bergisch Gladbach, Germany), and filtered sequentially through 70 μm cell strainers (*BD Bioscience*, 352350), and cells were washed once with PBS.

To obtain single-cell suspension from lymph nodes, samples were ground in 40 μm cell strainers (*BD Bioscience*, 352340) with 5 mL PBS solution, and then filtered with 70 μm cell strainers (*BD Bioscience*, 352350). Cells were washed once with PBS.

For surface staining, the cells were stained with appropriate antibodies against surface antigens in PBS on ice for 30 min. The cellular viability was assessed by staining with 7-amino actinomycin D (7-AAD) (*BioLegend*, 420404; 0.5 μg/mL) to exclude dead cells. For the analysis of IL-4、IFN-γ and IL-17A production, in vitro re-stimulation, and intracellular staining, single-cell suspensions were incubated for 4 h at 37 °C with PMA (*Sigma-Aldrich*, p1585; 200 ng/mL), brefeldin A (*BioLegend*, 420601; 5 μg/mL), and ionomycin (*Abcam*, ab120116; 1 μg/mL). The cells were then washed and stained with the fixable viability stain 620 (FVS 620; *BD-Biosciences*, 564996) for 10 min. After performing surface staining as described above, cells were fixed with 4% paraformaldehyde and permeabilized with PBS supplemented with 0.1% Triton X-100. Intracellular staining with fluorescent-labeled antibodies was per- formed for 30 min in PBS. For flow cytometric analysis, the cells were washed and resuspended in PBS. Flow cytometry was per- formed using the NovoCyte flow cytometer and ACEA NovoExpressTM software (*ACEA Biosciences*, San Diego, CA, USA) and BD LSRFortessaTM and Flow Jo™ software (*BD Biosciences*, USA). The single-cell suspensions were stained with the following antibodies: CD3-APC-CY7 (100222), CD4-PerCP/Cy5.5 (100434), CD8-PE-CY7 (100722), IFN-γ-FITC (505806), IL-4-APC (562045), IL-17A- PE (506903), CD11b -FITC (11-0112-82), Ly6G-PE-cy7 (108416), Ly6C-APC/Cy7 (1208026), Siglec-F-Bv421 (E50-2440), CD11c-APC/Cy7 (117324), MHCII-FITC(I-A-I-E, 107606). Antibodies were purchased from *eBioscience* and *BioLegend* and used at 1:100 dilution.

### Genome-wide transcriptome profiling by RNA-Seq

Total RNA of mice’s back skin was isolated with Trizol (Invitrogen) and subjected to RNA-seq analysis. RNA sequencing was performed by *Majorbio Bio-pharm Technology Co., Ltd* (Shanghai, China). The raw reads (SRA Data Accession number: *PRJNA917152*) were aligned to the mm10 reference genome (build mm10) by using HISAT2 software. The mapping rate was more than 90% overall across all the samples. StringTie was used to quantify the gene expression counts. Differential expression analysis was performed on the count data using the R package DESeq2. Benjamini–Hochberg step-up method to control false discovery rate. Significant genes are defined by a Benjamini and Hochberg corrected *p* value of cut-off of 0.05 and fold-change of at least 2.

Through GO analysis, the functions of genes at different levels were analyzed, and the clustering items with significant statistical differences were drawn into a functional clustering relationship diagram, relationship weight.

KEGG pathway analysis was performed on the differential genes with FC > 2 in the two groups of transcriptomics, and *p* value < 0.01 was selected as a statistical enrichment difference, and the signal pathway with a higher enrichment index was analyzed by the enrichment index, to analyze the KEGG pathway layer (KEGG pathway), so as to understand the transcriptome data more intuitively and comprehensively.

### Enzyme activity was detected by the serine protease activity probe

The tissue was ground in liquid nitrogen, resuspended in cold lysis buffer (20 mM HEPES pH 7.2, 2 mM DTT, 250 mM sucrose, 1 mM MgCl_2_, 2.5 U/mL benzonase), and incubated on ice (15–30 min). Protein concentrations were determined by a Quick StartTM Bradford Protein Assay and diluted samples were flash-frozen in liquid nitrogen and stored at −80 °C until further use.

The sample was diluted to 2 mg/mL, and 50 μL was absorbed. The probe was balanced in desiccant to room temperature, and 100 μL DMSO was added to prepare a 0.1 mm storage solution. 1 μL of ActivX^®^ Serine Hydrolase Probes (*Thermo*, 88318) was added to each sample at a final concentration of 2 μm/μL. After mixing, the samples were incubated for 1 h at room temperature under the light. The reaction was terminated by boiling 10 μL 6X SDS–PAGE protein loading buffer for 5 min. The reaction was electrophoresed with 12% SDS–PAGE. Gels were scanned using Cy3 and Cy5 multichannel settings (605/50 and 695/55, filters respectively) and stained with Coomassie after scanning. Fluorescence intensity was analyzed by Image J. The SDS–PAGE with fluorescence coloring was placed in Coomassie brilliant blue glue dye solution and stained on a shaker for 2 h. Then, the molecules with fluorescence coloring and Coomassie brilliant blue staining were decolorized and scanned, and the different bands were cut out for identification.

### Lentiviral gene overexpression in vitro

Lentivirus gene overexpression was performed by using pCDH-CMV vector to overexpress homo WFDC12 (Genewiz). Lentivirus was prepared by transient transfection of HEK293T cells with transfer vectors along with second-generation packaging constructs (pMD2.G and psPAX2). The viral titers were determined with qPCR. HaCaT cells were transfected with concentrated virus supernatant overnight in the presence of polybrene (5 mg/mL) and selected in puromycin (0.5 mg/mL).

### siRNA-mediated gene silencing in vitro

RNAi transfection was performed in cells at 30–50% confluency, mixing 20 pmol siRNA with RNAFit transfection reagent according to the manufacturer’s conditions. siRNAs and RNAFit transfection reagent were obtained from Shanghai HANBIO *Co., Ltd*. 72 h post-transfection, cells were harvested and processed as required. All siRNA sequences corresponding to all genes were shown in Supplementary Table S4.

### In vitro keratinocyte wound healing assay and proliferation assay

Cell migration ability was calculated using a wound healing assay. Keratinocytes were plated in 6-well plates at a concentration of about 1.5 × 10^6^ cells/well and allowed to form a confluent monolayer for 12–24 h. The monolayer was scratched with a sterile 200 µL (yellow) pipette tip both horizontally and vertically across the plate, washed with serum-free medium to remove floating and detached cells, and photographed (0 and 12 h) using an inverted fluorescence microscope (Olympus, Tokyo, Japan). Cell proliferation assay was measured by Cell Counting Kit-8 (Bimake, China) according to the manufacturer’s instructions. Keratinocytes were plated in 96-well plates at a density of 2 × 10^3^ cells per well. After seeding, cell proliferation was assessed. The cells were incubated for 2 h in 100 µL of CCK-8 reagent at 37 °C. The optical density (OD) at 450 nm was determined using a microplate reader.

### Statistics

The statistical software GraphPad Prism 8.0.8 was used for data analysis in the experiment. The difference between the two groups was compared by unpaired or paired *T*-test, and the experimental data were expressed as mean ± standard error. * Indicates *p* < 0.05, the difference is statistically significant, ** indicates *p* < 0.01, the difference is statistically very significant, *** indicates *p* < 0.001, the difference is statistically extremely significant, and ns indicates no statistical significance.

## Supplementary information

Original Data File

Supplemental Material

Supplementary Table S2

Supplementary Figure S7

## Data Availability

All data of the present study can be available with the approval of the corresponding authors.

## Material availability

All materials of the present study can be available with the approval of the corresponding authors.
