# Supplementary material for: WFDC12-overexpressing contributes to the development of atopic dermatitis via accelerating ALOX12/15 metabolism and PAF accumulation
Source: Cell Death Dis. 2023 Mar 8;14(3):185. doi: 10.1038/s41419-023-05686-3 (PMC9992393; doi:10.1038/s41419-023-05686-3)
Supplement: Supplementary file 1 — Original Data File [file 41419_2023_5686_MOESM1_ESM.docx]

**Supplementary Material 2**

**WFDC12-overexpressing contributes to the development of atopic dermatitis via accelerating ALOX12/15 metabolism and PAF accumulation**

Guolin Li^1*^, Linna Gu^1*^, Fulei Zhao^1*^, Yawen Hu^1^, Xiaoyan Wang^1^, Fanlian Zeng^1^, Jiadong Yu^1^, Chengcheng Yue ^1^, Pei Zhou^1^, Ya Li^1^, Yuting Feng^1^, Jing Hu^1^, Nongyu Huang^1^, Wenling Wu^1^, Kaijun Cui^2^, Wei Li^3^, Jiong Li^1^^#^.

^1^ State Key Laboratory of Biotherapy and Cancer Center, West China Hospital, West China Medical School, Sichuan University and Collaborative Innovation Center for Biotherapy, Chengdu, China

^2^ Department of Cardiology, West China Hospital, Sichuan University, Chengdu, China

^3^ Department of Dermatovenereology, West China Hospital, Sichuan University, Chengdu, China.

^*^ These authors contributed equally to this work.

^#^ Corresponding author.

Jiong Li, State Key Laboratory of Biotherapy and Cancer Center, West China Hospital, West China Medical School, Sichuan University and Collaborative Innovation Center for Biotherapy, Chengdu, China.

Email: lijionghh@scu.edu.cn


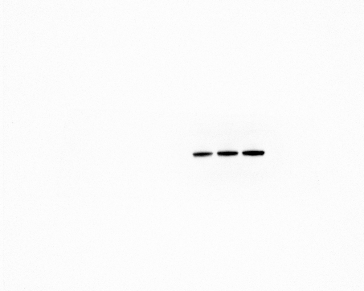

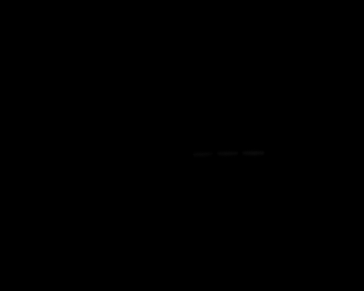

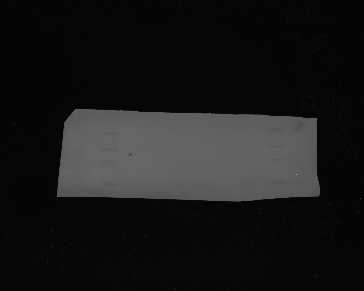


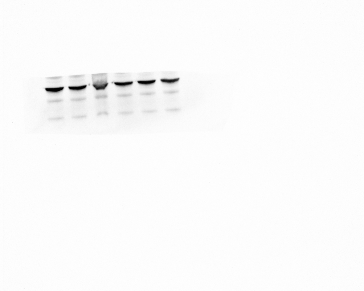

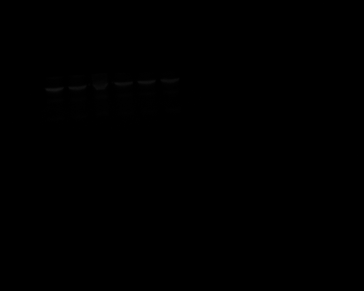

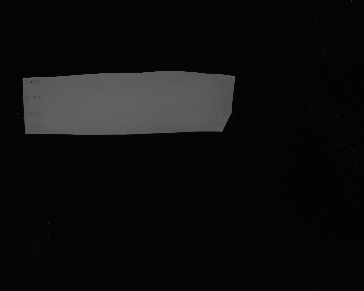


**The original images of western blotting results of WFDC12 (up) and β-Actin (down) in the dorsal skins of WT and TG.**
