# Supplementary material for: WFDC12-overexpressing contributes to the development of atopic dermatitis via accelerating ALOX12/15 metabolism and PAF accumulation
Source: Cell Death Dis. 2023 Mar 8;14(3):185. doi: 10.1038/s41419-023-05686-3 (PMC9992393; doi:10.1038/s41419-023-05686-3)
Supplement: Supplementary file 2 — Supplemental Material [file 41419_2023_5686_MOESM2_ESM.docx]

**Supplementary Material**

**WFDC12-overexpressing contributes to the development of atopic dermatitis via accelerating ALOX12/15 metabolism and PAF accumulation**

Guolin Li^1*^, Linna Gu^1*^, Fulei Zhao^1*^, Yawen Hu^1^, Xiaoyan Wang^1^, Fanlian Zeng^1^, Jiadong Yu^1^, Chengcheng Yue ^1^, Pei Zhou^1^, Ya Li^1^, Yuting Feng^1^, Jing Hu^1^, Nongyu Huang^1^, Wenling Wu^1^, Kaijun Cui^2^, Wei Li^3^, Jiong Li^1^^#^.

^1^ State Key Laboratory of Biotherapy and Cancer Center, West China Hospital, West China Medical School, Sichuan University and Collaborative Innovation Center for Biotherapy, Chengdu, China

^2^ Department of Cardiology, West China Hospital, Sichuan University, Chengdu, China

^3^ Department of Dermatovenereology, West China Hospital, Sichuan University, Chengdu, China.

^*^ These authors contributed equally to this work.

^#^ Corresponding author.

Jiong Li, State Key Laboratory of Biotherapy and Cancer Center, West China Hospital, West China Medical School, Sichuan University and Collaborative Innovation Center for Biotherapy, Chengdu, China.

Email: lijionghh@scu.edu.cn

**Supplementary Table S1. Partial primers used for RT-qPCR and PCR analysis.**

| Gene | Forward (F) / Reverse (R) | Sequence (5’ to 3‘’) |
| --- | --- | --- |
| *β-actin* | F | CCTCTATGCCAACACAGTGC |
|  | R | ACATCTGCTGGAAGGTGGAC |
| *WFDC12* | F | AATAATCAGCCAGGACTTC |
|  | R | CACACTCAATGTAGTTCTTATG |
| *IL-6* | F | CTGCAAGAGACTTCCATCCAG |
|  | R | AGTGGTATAGACAGGTCTGTTGG |
| *IL-37* | F | TGCAAGTACTAGTACGGATGGGGC |
|  | R | GGACTTCTTGTGCCATTTCCTGAG |
| *IL-17A* | F | CTCAGACTACCTCAACCGTTCC |
|  | R | CATGTGGTGGTCCAGCTTTCC |
| *TNF-α* | F | GCCACCACGCTCTTCTGTCT |
|  | R | ACTCCAGCTGCTCCTCCACTT |
| *ALOX12* | F | TTATGAAGCTCCGCTCTGGC |
|  | R | CCAAAGGCAAAGCACTCCAC |
| *ALOX15* | F | TCGCAAGGATGCTGAAGAGTGG |
|  | R | GGACTGTAGTTCAATCTCCAGAC |

**Supplementary Table S3. The protein analysis result of MS.**

| **NCBI accession NO.** | **Protein name** | **Coverage / %** | **Molecular weight （KD）** |
| --- | --- | --- | --- |
| NP_038653 | Platelet-activating factor acetylhydrolase IB subunit beta (PAF-AHIB) | 72.68 | 46.64 |
| XP_044093423 | Platelet-activating factor acetylhydrolase 2 (PAF-AH2) | 18.21 | 43.53 |
| NP_001191262 | Cytochrome P450, family 4, subfamily f, polypeptide 14 (CYP450 4F14) | 1.34 | 59.76 |

**Supplementary Table S4. siRNAs used for gene silencing.**

| Name | Target mRNA | Sense sequence (5’ to 3’) |
| --- | --- | --- |
| siRNA NC | / | UUCUCCGAACGUGUCACGUTT |
| siALOX12-1 | ALOX12 | GCAGGAGACAAUGCCUUAGAUTT |
| siALOX12-2 | ALOX12 | CCUGCCUUAUGAAUACCUCAATT |
| siALOX12-3 | ALOX12 | CUCAGCCAAUUUCAAGCAGAUTT |
| siALOX15-1 | ALOX15 | GGCAAGUCAUGAAUCGGUATT |
| siALOX15-2 | ALOX15 | GGACAAAAGACUUGAAUUUTT |
| siALOX15-3 | ALOX15 | CACUCUGUUUGAAGCGGAUUUTT |


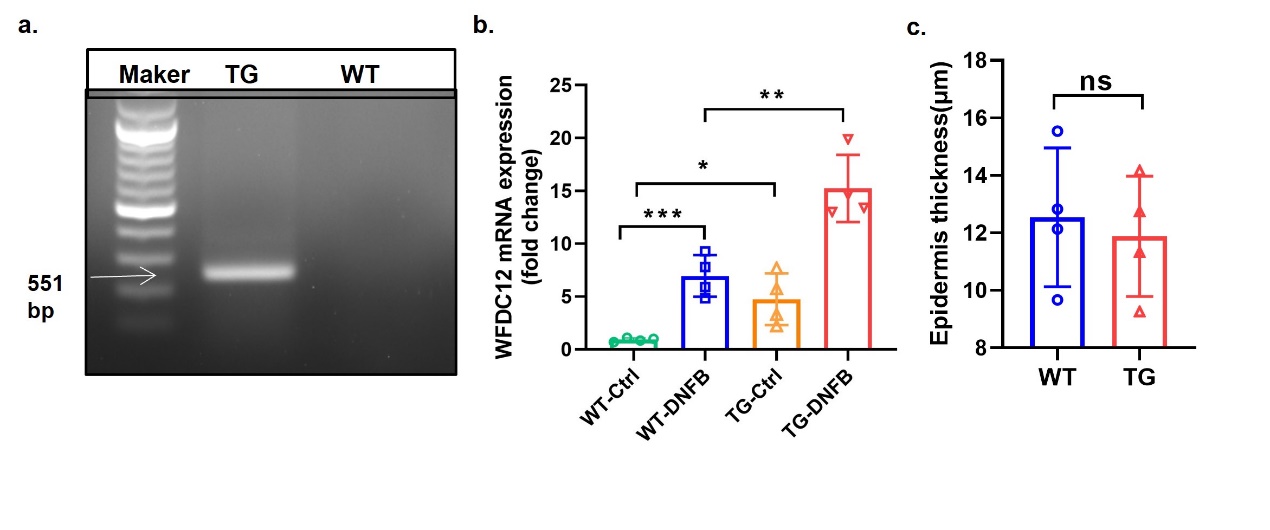
**Supplementary Figure S1.**

**Mice identification, detection of WFDC12 and epidermis thickness in mice.**

**(a)** PCR analysis was used to detect K14-WFDC12 gene in WT and TG mice. **(b)** RT-PCR analysis was used to detect WFDC12 mRNA levels in WT and TG mice. **(c)** The epidermis thickness detection in mice**.**


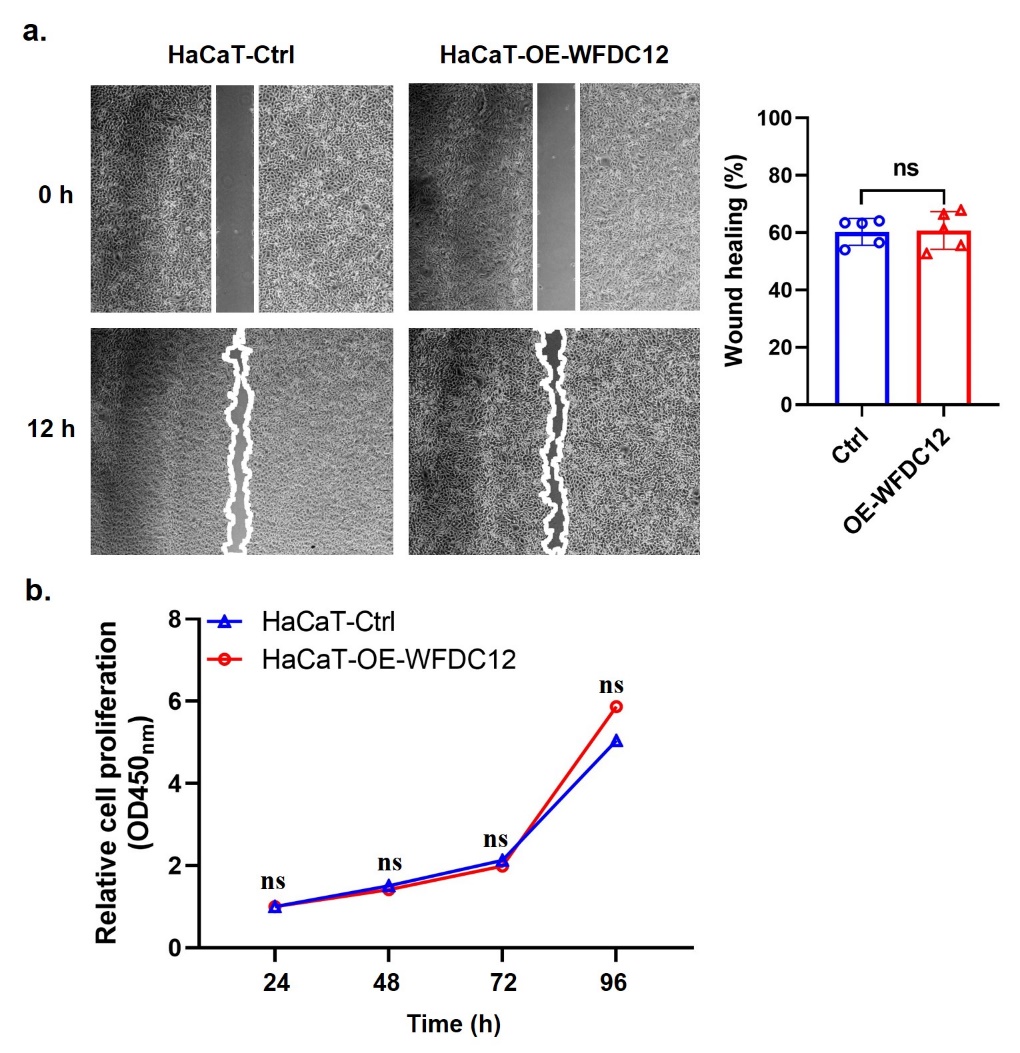


**Supplementary Figure S2.**

**WFDC12-overexpressing has no effect on the migration and proliferation of HaCaT cells.**

**(a)** Representative images of the scratch assay (left) and wound closure rate (right) of HaCaT cells (n = 5) after overexpression of WFDC12. **(b)** Proliferation of HaCaT cells (n = 3) after overexpression of WFDC12 was detected using cell proliferation detection reagent.


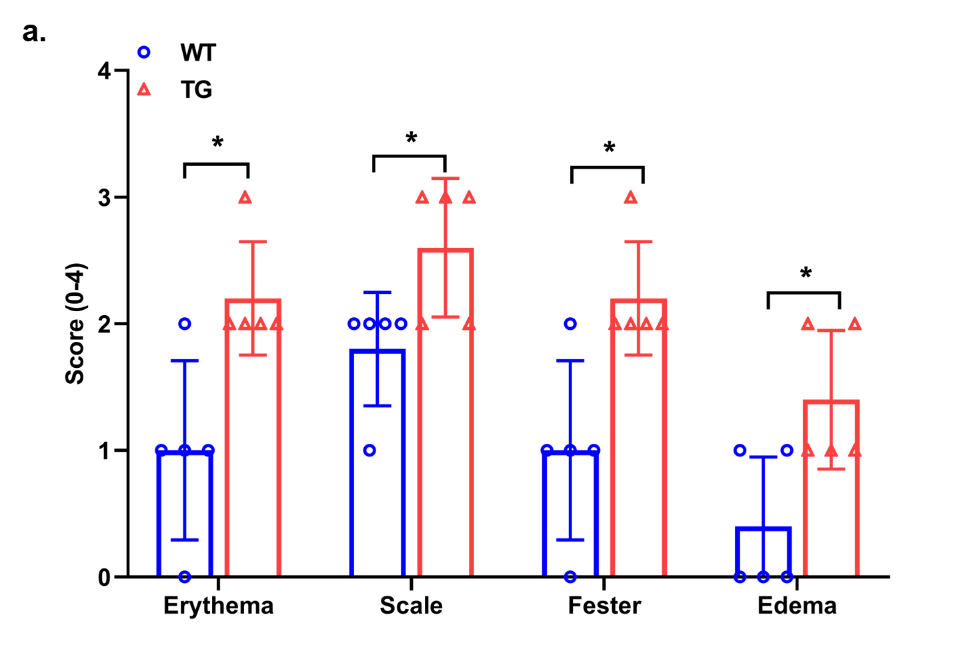


**Supplementary Figure S3.**

**Score of dorsal skin lesions of after 21 days of modeling (n = 5).**


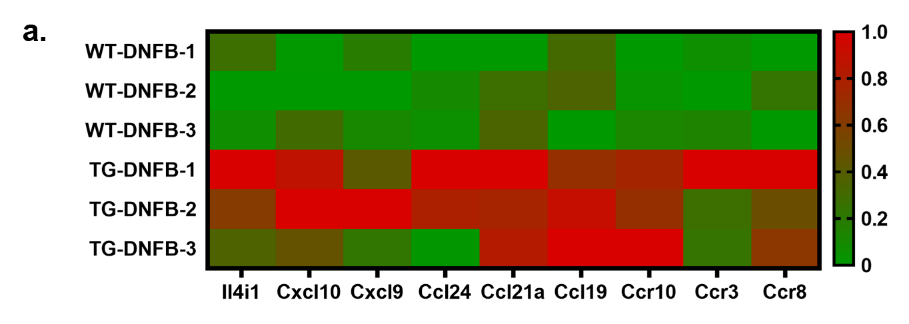


**Supplementary Figure S4.**

**The expression heat map of connected EDGs (including cytokine, chemokine and receptor genes) (n=3).**


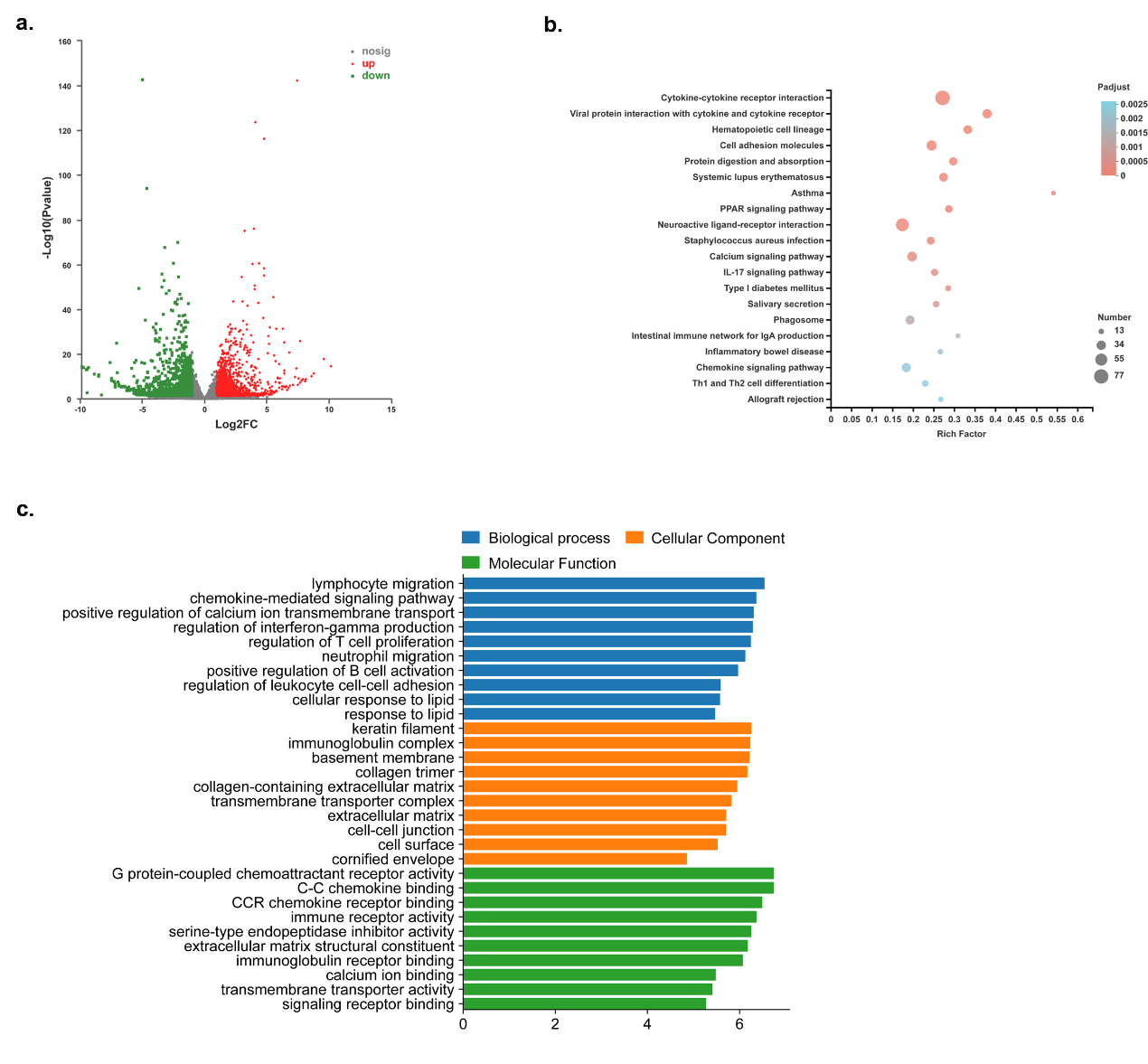


**Supplementary Figure S5.**

**The EDGs analysis in WT-DNFB and WT-Ctrl mice.**

**(a)** Volcano map of DEGs in WT-DNFB/ WT-CTRL. DEGs were more than 2 times and FDR ≤ 0.05. The abscissa represents the fold change of gene expression in each group. The ordinate represents the statistical difference of gene expression. Red dots and blue dots represent upregulated genes and downregulated gene in WT-DNFB/ WT-CTRL, respectively. Gray dots indicate the indifference genes. **(b)** DEGs were clustered in the term of biological process, subcellular localization and molecular function. **(c)** KEGG Pathway enrichment analysis results. The results were arranged according to P value from the smallest to the largest and 10 items were selected for drawing.


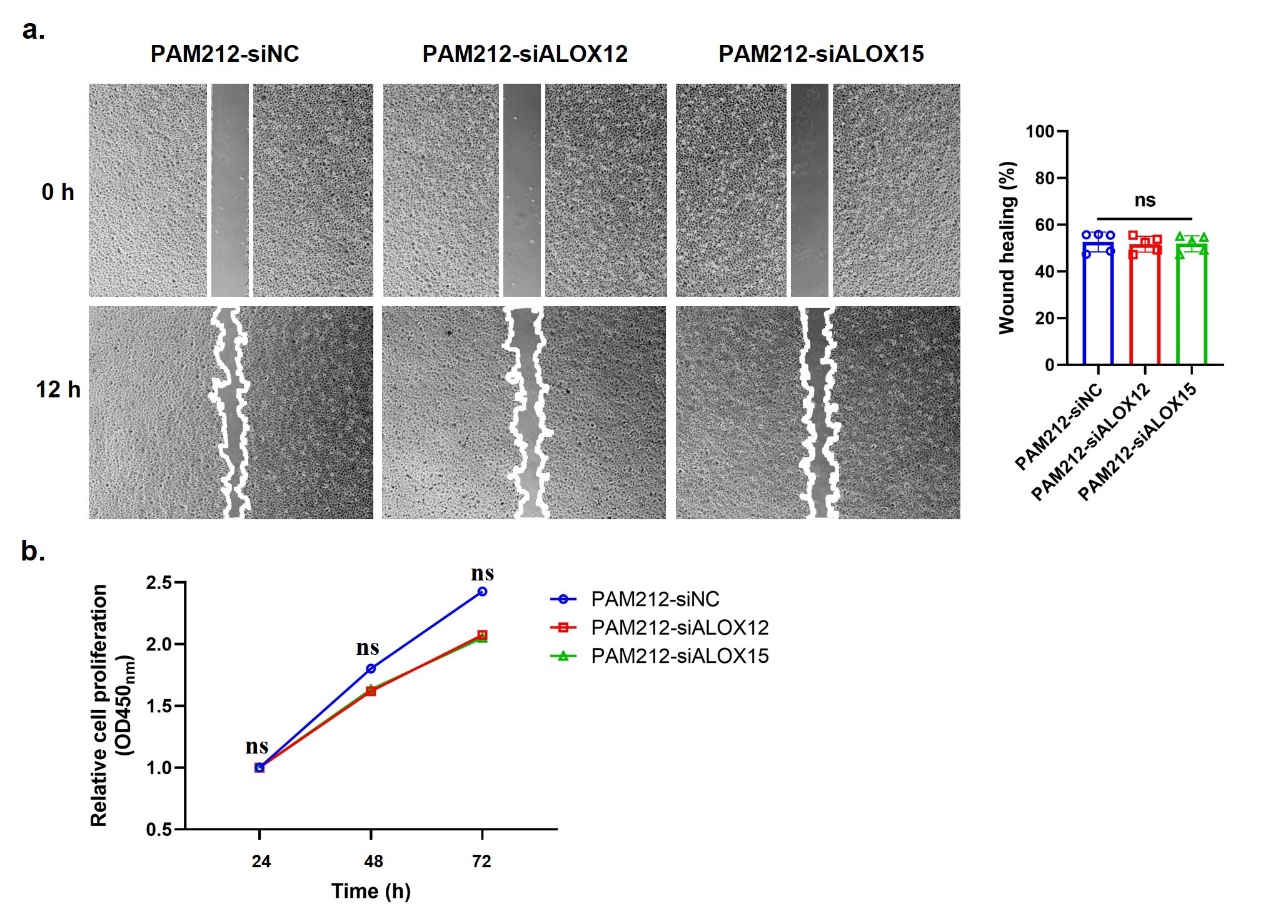
**S****upplementary Figure S6.**

**ALOX12/15 silencing have no effect on the migration and proliferation of HaCaT cells.**

**(a)** Representative images of the scratch assay (left) and wound closure rate (right) of PAM212 cells (n = 5) after overexpression of WFDC12. **(b)** Proliferation of PAM212 cells (n = 3) after overexpression of WFDC12 was detected using cell proliferation detection reagent.
