# Supplementary material for: WFDC12-overexpressing contributes to the development of atopic dermatitis via accelerating ALOX12/15 metabolism and PAF accumulation
Source: Cell Death Dis. 2023 Mar 8;14(3):185. doi: 10.1038/s41419-023-05686-3 (PMC9992393; doi:10.1038/s41419-023-05686-3)
Supplement: Supplementary file 3 — Supplementary Table S2 [file 41419_2023_5686_MOESM3_ESM.pdf]

Supplementary Table S2. The differentially expressed genes (DEGs) in WT-DNFB and TG-DNFB								
Gene_id	Gene name	Gene description	FC(TG-DNFB/WT-DNFB)	Log2FC(TG-DNFB/WT-DNFB)	Pvalue	Padjust	Significant	Regulate
ENSMUSG00000049548	Krt82	keratin 82 [Source:MGI Symbol;Acc:MGI:2149248]	0.086652496	-3.528614885	7.76E-29	6.81E-25	yes	down
ENSMUSG00000030739	Myh14	myosin, heavy polypeptide 14 [Source:MGI Symbol;Acc:MGI:1919210]	0.260252393	-1.942016665	7.88E-29	6.81E-25	yes	down
ENSMUSG00000024600	Slc27a6	solute carrier family 27 (fatty acid transporter), member 6 [Source:MGI Symbol;Acc:MGI:3036230]	0.197579691	-2.339493435	3.84E-27	2.21E-23	yes	down
ENSMUSG00000112600	Gm49918	predicted gene, 49918 [Source:MGI Symbol;Acc:MGI:6270621]	0.050598971	-4.304748147	5.81E-26	2.51E-22	yes	down
ENSMUSG00000069805	Fbp1	fructose bisphosphatase 1 [Source:MGI	0.091847602	-3.444614129	1.27E-25	4.32E-22	yes	down
ENSMUSG00000032726	Bmp8a	bone morphogenetic protein 8a [Source:MGI Symbol;Acc:MGI:104515]	0.119728788	-3.062158019	1.50E-25	4.32E-22	yes	down
ENSMUSG00000049598	Vsig8	V-set and immunoglobulin domain containing 8 [Source:MGI Symbol;Acc:MGI:3642995]	0.085892838	-3.541318354	2.63E-25	6.48E-22	yes	down
ENSMUSG00000112380	Gm9736	predicted gene 9736 [Source:MGI Symbol;Acc:MGI:3842085]	0.068812836	-3.861178492	9.38E-25	2.03E-21	yes	down
ENSMUSG00000069582	Krtap10-4	keratin associated protein 10-4 [Source:MGI Symbol;Acc:MGI:1925013]	0.075168488	-3.733728209	3.36E-24	6.40E-21	yes	down
ENSMUSG00000112170	Gm9508	predicted gene 9508 [Source:MGI Symbol;Acc:MGI:3779918]	0.066457538	-3.91142334	3.70E-24	6.40E-21	yes	down
ENSMUSG00000025328	Padi3	peptidyl arginine deiminase, type III [Source:MGI Symbol;Acc:MGI:1338891]	0.105003731	-3.251487502	4.41E-24	6.94E-21	yes	down
ENSMUSG00000111915	Gm3285	predicted gene 3285 [Source:MGI Symbol;Acc:MGI:3781463]	0.075591807	-3.72562632	1.21E-23	1.74E-20	yes	down
ENSMUSG00000063661	Krt73	keratin 73 [Source:MGI Symbol;Acc:MGI:3607712]	0.096091066	-3.379453892	5.49E-23	7.30E-20	yes	down
ENSMUSG00000035831	Krt25	keratin 25 [Source:MGI Symbol;Acc:MGI:1918060]	0.100070857	-3.320906205	1.27E-22	1.56E-19	yes	down
ENSMUSG00000051879	Krt71	keratin 71 [Source:MGI Symbol;Acc:MGI:1861586]	0.103783296	-3.268353835	1.68E-22	1.93E-19	yes	down
ENSMUSG00000094913	Gm9507	predicted gene 9507 [Source:MGI Symbol;Acc:MGI:3779917]	0.062778126	-3.993594226	1.85E-22	1.94E-19	yes	down
ENSMUSG00000110324	Gm40460	predicted gene, 40460 [Source:MGI Symbol;Acc:MGI:5623345]	0.060485372	-4.04726992	1.90E-22	1.94E-19	yes	down
ENSMUSG00000073785	Krtap5-5	keratin associated protein 5-5 [Source:MGI Symbol;Acc:MGI:2149673]	0.067031441	-3.89901825	2.43E-22	2.33E-19	yes	down
ENSMUSG00000061584	Lyg2	lysozyme G-like 2 [Source:MGI Symbol;Acc:MGI:2685622]	0.075685346	-3.723842188	3.48E-22	3.17E-19	yes	down
ENSMUSG00000090471	Gm4553	predicted gene 4553 [Source:MGI Symbol;Acc:MGI:3782737]	0.06223775	-4.006066286	3.72E-22	3.21E-19	yes	down
ENSMUSG00000078253	Krtap16-1	keratin associated protein 16-1 [Source:MGI Symbol;Acc:MGI:3650326]	0.081689955	-3.613697504	4.26E-22	3.50E-19	yes	down
ENSMUSG00000075567	Krtap1-4	keratin associated protein 1-4 [Source:MGI Symbol;Acc:MGI:3651229]	0.076101132	-3.715938273	6.51E-22	5.11E-19	yes	down
ENSMUSG00000023387	Kcnk16	potassium channel, subfamily K, member 16 [Source:MGI Symbol;Acc:MGI:1921821]	0.081629907	-3.614758373	7.42E-22	5.57E-19	yes	down
ENSMUSG00000038560	Sp6	trans-acting transcription factor 6 [Source:MGI Symbol;Acc:MGI:1932575]	0.150269616	-2.734374767	3.00E-21	2.16E-18	yes	down
ENSMUSG00000112223	Krtap10-10	keratin associated protein 10-10 [Source:MGI Symbol;Acc:MGI:3645300]	0.074358703	-3.749354575	4.47E-21	3.09E-18	yes	down
ENSMUSG00000114011	Gm47955	predicted gene, 47955 [Source:MGI Symbol;Acc:MGI:6097226]	0.067784694	-3.882896635	4.77E-21	3.14E-18	yes	down
ENSMUSG00000110091	Gm39115	predicted gene, 39115 [Source:MGI Symbol;Acc:MGI:5622000]	0.061652854	-4.019688512	4.90E-21	3.14E-18	yes	down
ENSMUSG00000110061	Gm45337	predicted gene 45337 [Source:MGI Symbol;Acc:MGI:5791173]	0.082263011	-3.603612318	5.39E-21	3.33E-18	yes	down
ENSMUSG00000073413	Ly6g6d	lymphocyte antigen 6 complex, locus G6D [Source:MGI Symbol;Acc:MGI:2148931]	0.102501752	-3.286279519	6.01E-21	3.58E-18	yes	down
ENSMUSG00000116336	Gm49425	predicted gene, 49425 [Source:MGI Symbol;Acc:MGI:6155060]	0.079054782	-3.661003465	6.74E-21	3.88E-18	yes	down
ENSMUSG00000022986	Krt75	keratin 75 [Source:MGI Symbol;Acc:MGI:1923500]	0.122559171	-3.028449652	7.74E-21	4.20E-18	yes	down
ENSMUSG00000027313	Chac1	ChaC, cation transport regulator 1 [Source:MGI Symbol;Acc:MGI:1916315]	0.122049031	-3.034467255	7.78E-21	4.20E-18	yes	down
ENSMUSG00000071195	Gm10318	predicted gene 10318 [Source:MGI Symbol;Acc:MGI:3704118]	0.077830397	-3.683522465	1.33E-20	6.99E-18	yes	down

ENSMUSG00000096131	Gm9639	predicted gene 9639 [Source:MGI Symbol;Acc:MGI:3780046]	0.077697859	-3.685981342	3.53E-20	1.79E-17	yes	down
ENSMUSG00000078668	Gm11595	predicted gene 11595 [Source:MGI Symbol;Acc:MGI:3652308]	0.083813758	-3.576669104	3.80E-20	1.88E-17	yes	down
ENSMUSG00000094120	Gm3233	predicted gene 3233 [Source:MGI Symbol;Acc:MGI:3781411]	0.069958633	-3.837354094	4.76E-20	2.29E-17	yes	down
ENSMUSG00000109859	Gm45618	predicted gene 45618 [Source:MGI Symbol;Acc:MGI:5791454]	0.066923404	-3.901345355	6.32E-20	2.95E-17	yes	down
ENSMUSG00000051396	Gm45902	predicted gene 45902 [Source:MGI Symbol;Acc:MGI:5805017]	0.221984194	-2.171471136	6.92E-20	3.15E-17	yes	down
ENSMUSG00000070990	Foxe1	forkhead box E1 [Source:MGI Symbol;Acc:MGI:1353500]	0.157349706	-2.667953611	7.72E-20	3.42E-17	yes	down
ENSMUSG00000046474	Krtap4-16	keratin associated protein 4-16 [Source:MGI Symbol;Acc:MGI:3651030]	0.089747226	-3.477988836	9.26E-20	4.00E-17	yes	down
ENSMUSG00000059169	Krt40	keratin 40 [Source:MGI Symbol;Acc:MGI:3629968]	0.09461732	-3.401751887	1.11E-19	4.65E-17	yes	down
ENSMUSG00000114861	Gm47991	predicted gene, 47991 [Source:MGI Symbol;Acc:MGI:6097286]	0.078265662	-3.675476713	1.13E-19	4.65E-17	yes	down
ENSMUSG00000095817	Gm3238	predicted gene 3238 [Source:MGI Symbol;Acc:MGI:3781416]	0.067571616	-3.88743884	3.23E-19	1.30E-16	yes	down
ENSMUSG00000113084	A030003K21Rik	RIKEN cDNA A030003K21 gene [Source:MGI Symbol;Acc:MGI:1925163]	0.067604312	-3.886740925	9.83E-19	3.86E-16	yes	down
ENSMUSG00000048981	Krt31	keratin 31 [Source:MGI Symbol;Acc:MGI:1309993]	0.089383835	-3.483842244	1.05E-18	4.02E-16	yes	down
ENSMUSG00000067596	Krt74	keratin 74 [Source:MGI Symbol;Acc:MGI:3629975]	0.107169359	-3.222035611	1.14E-18	4.28E-16	yes	down
ENSMUSG00000001510	Dlx3	distal-less homeobox 3 [Source:MGI Symbol;Acc:MGI:94903]	0.151031631	-2.727077369	1.25E-18	4.60E-16	yes	down
ENSMUSG00000078262	Krtap4-9	keratin associated protein 4-9 [Source:MGI Symbol;Acc:MGI:3652060]	0.088211402	-3.502891037	1.32E-18	4.70E-16	yes	down
ENSMUSG00000109655	Gm29735	predicted gene, 29735 [Source:MGI Symbol;Acc:MGI:5588894]	0.068026134	-3.877767096	1.33E-18	4.70E-16	yes	down
ENSMUSG00000048013	Krt35	keratin 35 [Source:MGI Symbol;Acc:MGI:1858899]	0.080763765	-3.630148016	1.50E-18	5.19E-16	yes	down
ENSMUSG00000042109	Csdc2	cold shock domain containing C2, RNA binding [Source:MGI Symbol;Acc:MGI:2146027]	0.146819167	-2.767887776	1.72E-18	5.84E-16	yes	down
ENSMUSG00000113097	Gm47959	predicted gene, 47959 [Source:MGI Symbol;Acc:MGI:6097234]	0.080636394	-3.632425065	1.82E-18	6.04E-16	yes	down
ENSMUSG00000001021	S100a3	S100 calcium binding protein A3 [Source:MGI Symbol;Acc:MGI:1338849]	0.090276882	-3.4694996	2.02E-18	6.57E-16	yes	down
ENSMUSG00000058368	Krtap21-1	keratin associated protein 21-1 [Source:MGI Symbol;Acc:MGI:2157767]	0.130785844	-2.9347217	2.15E-18	6.87E-16	yes	down
ENSMUSG00000046095	Krt32	keratin 32 [Source:MGI Symbol;Acc:MGI:1309995]	0.103031315	-3.278845204	2.92E-18	9.19E-16	yes	down
ENSMUSG00000001655	Hoxc13	homeobox C13 [Source:MGI Symbol;Acc:MGI:99560]	0.251227815	-1.99293189	3.08E-18	9.51E-16	yes	down
ENSMUSG00000020891	Alox8	arachidonate 8-lipoxygenase [Source:MGI Symbol;Acc:MGI:1098228]	0.106587893	-3.229884526	3.96E-18	1.20E-15	yes	down
ENSMUSG00000017588	Krt27	keratin 27 [Source:MGI Symbol;Acc:MGI:1339999]	0.11152692	-3.16453611	4.18E-18	1.24E-15	yes	down
ENSMUSG00000104423	A030005K14Rik	RIKEN cDNA A030005K14 gene [Source:MGI Symbol;Acc:MGI:1925171]	0.064543389	-3.953586863	4.47E-18	1.31E-15	yes	down
ENSMUSG00000027403	Tgm6	transglutaminase 6 [Source:MGI Symbol;Acc:MGI:3044321]	0.090079304	-3.472660502	4.82E-18	1.39E-15	yes	down
ENSMUSG00000070334	Krtap31-1	keratin associated protein 31-1 [Source:MGI Symbol;Acc:MGI:1918081]	0.095174717	-3.393277812	5.21E-18	1.48E-15	yes	down
ENSMUSG00000038599	Capn8	calpain 8 [Source:MGI Symbol;Acc:MGI:2181366]	0.085594819	-3.54633271	7.18E-18	2.00E-15	yes	down
ENSMUSG00000090455	Gm2431	predicted gene 2431 [Source:MGI Symbol;Acc:MGI:3780598]	0.045200439	-4.467519412	9.47E-18	2.60E-15	yes	down
ENSMUSG00000089724	Krtap4-8	keratin associated protein 4-8 [Source:MGI Symbol;Acc:MGI:3652306]	0.090076712	-3.472702025	1.09E-17	2.94E-15	yes	down
ENSMUSG00000070473	Cldn3	claudin 3 [Source:MGI Symbol;Acc:MGI:1329044]	0.142715572	-2.808785334	1.39E-17	3.66E-15	yes	down
ENSMUSG00000045109	Krtap4-7	keratin associated protein 4-7 [Source:MGI Symbol;Acc:MGI:1923694]	0.08867033	-3.495404742	1.41E-17	3.66E-15	yes	down
ENSMUSG00000063251	Krtap4-1	keratin associated protein 4-1 [Source:MGI Symbol;Acc:MGI:3622079]	0.092261662	-3.438124901	1.42E-17	3.66E-15	yes	down
ENSMUSG00000078252	Krtap17-1	keratin associated protein 17-1 [Source:MGI Symbol;Acc:MGI:1925164]	0.102071565	-3.292347082	1.61E-17	4.09E-15	yes	down
ENSMUSG00000069583	Krtap12-1	keratin associated protein 12-1 [Source:MGI Symbol;Acc:MGI:1328315]	0.092625401	-3.432448305	1.76E-17	4.41E-15	yes	down

ENSMUSG00000047641	Krt87	keratin 87 [Source:MGI Symbol;Acc:MGI:3665486]	0.106183202	-3.235372536	1.91E-17	4.71E-15	yes	down
ENSMUSG00000002057	Foxn1	forkhead box N1 [Source:MGI Symbol;Acc:MGI:102949]	0.15936235	-2.649617268	1.97E-17	4.81E-15	yes	down
ENSMUSG00000062278	Gm11562	predicted gene 11562 [Source:MGI Symbol;Acc:MGI:3652059]	0.092275615	-3.437906749	2.07E-17	4.96E-15	yes	down
ENSMUSG00000113267	Gm47969	predicted gene, 47969 [Source:MGI Symbol;Acc:MGI:6097249]	0.063402089	-3.979325812	2.40E-17	5.68E-15	yes	down
ENSMUSG00000057674	Gm11938	predicted gene 11938 [Source:MGI Symbol;Acc:MGI:3651233]	0.09305125	-3.425830661	2.45E-17	5.73E-15	yes	down
ENSMUSG00000047963	Stbd1	starch binding domain 1 [Source:MGI Symbol;Acc:MGI:1261768]	0.173271426	-2.528894336	2.57E-17	5.92E-15	yes	down
ENSMUSG00000052415	Tchh	trichohyalin [Source:MGI Symbol;Acc:MGI:2177944]	0.120852273	-3.048683486	3.49E-17	7.93E-15	yes	down
ENSMUSG00000030110	Ret	ret proto-oncogene [Source:MGI Symbol;Acc:MGI:97902]	0.356083569	-1.489712227	5.89E-17	1.31E-14	yes	down
ENSMUSG00000067615	Krt81	keratin 81 [Source:MGI Symbol;Acc:MGI:1928858]	0.107138368	-3.222452875	5.90E-17	1.31E-14	yes	down
ENSMUSG00000045005	Fzd5	frizzled class receptor 5 [Source:MGI Symbol;Acc:MGI:108571]	0.289331029	-1.789207045	6.28E-17	1.37E-14	yes	down
ENSMUSG00000078261	Gm11596	predicted gene 11596 [Source:MGI Symbol;Acc:MGI:3652177]	0.08979419	-3.477234092	6.78E-17	1.47E-14	yes	down
ENSMUSG00000110631	Gm42047	predicted gene, 42047 [Source:MGI Symbol;Acc:MGI:5624932]	3.455995828	1.789101476	9.86E-17	2.10E-14	yes	up
ENSMUSG00000101315	Krtap28-13	keratin associated protein 28-13 [Source:MGI Symbol;Acc:MGI:1918636]	0.070721731	-3.821702611	1.03E-16	2.17E-14	yes	down
ENSMUSG00000112864	Gm18596	predicted gene, 18596 [Source:MGI Symbol;Acc:MGI:5010781]	0.089003404	-3.489995673	1.13E-16	2.35E-14	yes	down
ENSMUSG00000078256	Gm11565	predicted gene 11565 [Source:MGI Symbol;Acc:MGI:3650327]	0.078601421	-3.669300794	1.51E-16	3.11E-14	yes	down
ENSMUSG00000048294	Krtap4-13	keratin associated protein 4-13 [Source:MGI Symbol;Acc:MGI:1916714]	0.084944176	-3.557341156	1.65E-16	3.36E-14	yes	down
ENSMUSG00000024664	Fads3	fatty acid desaturase 3 [Source:MGI Symbol;Acc:MGI:1928740]	0.2923631	-1.774166859	1.73E-16	3.47E-14	yes	down
ENSMUSG00000078260	Gm11569	predicted gene 11569 [Source:MGI Symbol;Acc:MGI:3709346]	0.071050996	-3.815001308	1.80E-16	3.57E-14	yes	down
ENSMUSG00000049556	Lingo1	leucine rich repeat and Ig domain containing 1 [Source:MGI Symbol;Acc:MGI:1915522]	0.308106526	-1.698498856	3.79E-16	7.46E-14	yes	down
ENSMUSG00000001804	Dsg4	desmoglein 4 [Source:MGI Symbol;Acc:MGI:2661061]	0.126718939	-2.980295933	4.46E-16	8.66E-14	yes	down
ENSMUSG00000096481	Gm3250	predicted gene 3250 [Source:MGI Symbol;Acc:MGI:3781428]	0.065060464	-3.942075081	5.21E-16	1.00E-13	yes	down
ENSMUSG00000076934	Iglv1	immunoglobulin lambda variable 1 [Source:MGI	4.126227478	2.044823359	5.70E-16	1.08E-13	yes	up
ENSMUSG00000113925	Gm7544	predicted gene 7544 [Source:MGI Symbol;Acc:MGI:3779750]	0.082732564	-3.595400901	7.34E-16	1.38E-13	yes	down
ENSMUSG00000110254	Gm47762	predicted gene, 47762 [Source:MGI Symbol;Acc:MGI:6096914]	0.053305866	-4.229561892	8.14E-16	1.51E-13	yes	down
ENSMUSG00000044139	Prss53	protease, serine 53 [Source:MGI Symbol;Acc:MGI:2652890]	0.150386588	-2.733252182	1.01E-15	1.85E-13	yes	down
ENSMUSG00000021130	Galnt16	polypeptide N-acetylgalactosaminyltransferase 16 [Source:MGI Symbol;Acc:MGI:1917754]	3.178375301	1.668289488	1.07E-15	1.94E-13	yes	up
ENSMUSG00000070803	Cited4	Cbp/p300-interacting transactivator, with Glu/Asp-rich carboxy-terminal domain, 4 [Source:MGI Symbol;Acc:MGI:1861694]	0.177727767	-2.492258999	1.16E-15	2.10E-13	yes	down
ENSMUSG00000031995	St14	suppression of tumorigenicity 14 (colon carcinoma) [Source:MGI Symbol;Acc:MGI:1338881]	0.291464251	-1.778609154	1.32E-15	2.35E-13	yes	down
ENSMUSG00000056270	Prr9	proline rich 9 [Source:MGI Symbol;Acc:MGI:1925680]	0.106230613	-3.234728516	1.33E-15	2.35E-13	yes	down
ENSMUSG00000034382	AI661453	expressed sequence AI661453 [Source:MGI Symbol;Acc:MGI:2146908]	0.286131696	-1.805248773	1.43E-15	2.50E-13	yes	down
ENSMUSG00000060756	Krtap2-4	keratin associated protein 2-4 [Source:MGI Symbol;Acc:MGI:1918703]	0.106290969	-3.233909073	1.88E-15	3.25E-13	yes	down
ENSMUSG00000055194	Actbl2	actin, beta-like 2 [Source:MGI Symbol;Acc:MGI:2444552]	0.085059679	-3.555380783	2.04E-15	3.49E-13	yes	down
ENSMUSG00000047109	Cldn14	claudin 14 [Source:MGI Symbol;Acc:MGI:1860425]	0.108818815	-3.200000073	2.26E-15	3.83E-13	yes	down
ENSMUSG00000024409	Psors1c2	psoriasis susceptibility 1 candidate 2 (human) [Source:MGI Symbol;Acc:MGI:1930025]	0.097741299	-3.354887913	2.31E-15	3.87E-13	yes	down
ENSMUSG00000050239	Krtap24-1	keratin associated protein 24-1 [Source:MGI Symbol;Acc:MGI:2685158]	0.084110326	-3.571573269	3.22E-15	5.31E-13	yes	down
ENSMUSG00000045236	Krtap5-4	keratin associated protein 5-4 [Source:MGI Symbol;Acc:MGI:1354758]	0.068716631	-3.86319688	3.23E-15	5.31E-13	yes	down

ENSMUSG00000039508	Calhm4	calcium homeostasis modulator family member 4 [Source:MGI Symbol;Acc:MGI:2685489]	0.094190133	-3.408280252	3.28E-15	5.35E-13	yes	down
ENSMUSG00000094012	Gm10024	predicted gene 10024 [Source:MGI Symbol;Acc:MGI:3641784]	0.086770199	-3.526656548	3.47E-15	5.61E-13	yes	down
ENSMUSG00000028751	Pla2g2e	phospholipase A2, group IIE [Source:MGI Symbol;Acc:MGI:1349660]	0.124340398	-3.007632997	3.77E-15	6.04E-13	yes	down
ENSMUSG00000021469	Msx2	msh homeobox 2 [Source:MGI Symbol;Acc:MGI:97169]	0.152688254	-2.71133901	6.16E-15	9.77E-13	yes	down
ENSMUSG00000056350	Krtap13-1	keratin associated protein 13-1 [Source:MGI Symbol;Acc:MGI:2146359]	0.102376729	-3.288040272	7.30E-15	1.15E-12	yes	down
ENSMUSG00000073786	Gm7579	predicted gene 7579 [Source:MGI Symbol;Acc:MGI:3647476]	0.067992923	-3.878471603	7.49E-15	1.17E-12	yes	down
ENSMUSG00000064165	Krt39	keratin 39 [Source:MGI Symbol;Acc:MGI:3588208]	0.086067466	-3.538388197	9.02E-15	1.39E-12	yes	down
ENSMUSG00000027068	Dhrs9	dehydrogenase/reductase (SDR family) member 9 [Source:MGI Symbol;Acc:MGI:2442798]	0.270979293	-1.883745481	9.10E-15	1.39E-12	yes	down
ENSMUSG00000022180	Slc7a8	solute carrier family 7 (cationic amino acid transporter, y+ system), member 8 [Source:MGI Symbol;Acc:MGI:1355323]	0.282300153	-1.824698184	1.09E-14	1.65E-12	yes	down
ENSMUSG00000044164	Rnf182	ring finger protein 182 [Source:MGI Symbol;Acc:MGI:3045355]	0.136137316	-2.876865526	1.28E-14	1.92E-12	yes	down
ENSMUSG00000067149	Jchain	immunoglobulin joining chain [Source:MGI	2.459520013	1.298376795	1.33E-14	1.98E-12	yes	up
ENSMUSG00000032537	Ephb1	Eph receptor B1 [Source:MGI Symbol;Acc:MGI:1096337]	0.269070512	-1.893943802	1.73E-14	2.56E-12	yes	down
ENSMUSG00000020916	Krt36	keratin 36 [Source:MGI Symbol;Acc:MGI:109364]	0.114740542	-3.123552854	2.01E-14	2.94E-12	yes	down
ENSMUSG00000026062	Slc9a2	solute carrier family 9 (sodium/hydrogen exchanger), member 2 [Source:MGI Symbol;Acc:MGI:105075]	0.190588473	-2.391467228	2.23E-14	3.22E-12	yes	down
ENSMUSG00000072849	Serpina1e	serine (or cysteine) peptidase inhibitor, clade A, member 1E [Source:MGI	0.141552612	-2.820589726	2.24E-14	3.22E-12	yes	down
ENSMUSG00000078735	Il11ra2	interleukin 11 receptor, alpha chain 2 [Source:MGI Symbol;Acc:MGI:109123]	0.175912485	-2.50707022	2.43E-14	3.47E-12	yes	down
ENSMUSG00000044649	Krtap4-2	keratin associated protein 4-2 [Source:MGI Symbol;Acc:MGI:1915923]	0.105687266	-3.242126536	3.30E-14	4.67E-12	yes	down
ENSMUSG00000020183	Cpm	carboxypeptidase M [Source:MGI Symbol;Acc:MGI:1917824]	0.180527715	-2.469707758	6.41E-14	9.00E-12	yes	down
ENSMUSG00000058172	Krtap6-1	keratin associated protein 6-1 [Source:MGI Symbol;Acc:MGI:1330228]	0.120062962	-3.058136928	7.47E-14	1.03E-11	yes	down
ENSMUSG00000095970	Gm19402	predicted gene, 19402 [Source:MGI Symbol;Acc:MGI:5011587]	0.078941346	-3.663075067	7.48E-14	1.03E-11	yes	down
ENSMUSG00000045319	Proser2	proline and serine rich 2 [Source:MGI Symbol;Acc:MGI:2442238]	0.285798623	-1.806929131	7.98E-14	1.09E-11	yes	down
ENSMUSG00000078258	Gm11564	predicted gene 11564 [Source:MGI Symbol;Acc:MGI:3650329]	0.100214106	-3.318842497	8.70E-14	1.18E-11	yes	down
ENSMUSG00000034295	Fhod3	formin homology 2 domain containing 3 [Source:MGI Symbol;Acc:MGI:1925847]	0.367635473	-1.443652118	1.01E-13	1.36E-11	yes	down
ENSMUSG00000096421	Gm10100	predicted gene 10100 [Source:MGI Symbol;Acc:MGI:3642388]	0.062111076	-4.009005639	1.03E-13	1.38E-11	yes	down
ENSMUSG00000054083	Capn12	calpain 12 [Source:MGI Symbol;Acc:MGI:1891369]	0.145655022	-2.779372652	1.07E-13	1.42E-11	yes	down
ENSMUSG00000051481	Krtap31-2	keratin associated protein 31-2 [Source:MGI Symbol;Acc:MGI:3650789]	0.102951362	-3.279965176	1.43E-13	1.89E-11	yes	down
ENSMUSG00000096380	Gm19668	predicted gene, 19668 [Source:MGI Symbol;Acc:MGI:5011853]	0.080701601	-3.631258887	1.73E-13	2.27E-11	yes	down
ENSMUSG00000036492	Rnf39	ring finger protein 39 [Source:MGI Symbol;Acc:MGI:2156378]	0.278169015	-1.845966365	1.87E-13	2.43E-11	yes	down
ENSMUSG00000030494	Rhpn2	rhophilin, Rho GTPase binding protein 2 [Source:MGI Symbol;Acc:MGI:1289234]	0.17927507	-2.479753211	1.95E-13	2.51E-11	yes	down
ENSMUSG00000025329	Padi1	peptidyl arginine deiminase, type I [Source:MGI Symbol;Acc:MGI:1338893]	0.118267465	-3.079874846	1.98E-13	2.53E-11	yes	down
ENSMUSG00000051379	Flrt3	fibronectin leucine rich transmembrane protein 3 [Source:MGI	0.359056131	-1.477718696	3.34E-13	4.24E-11	yes	down
ENSMUSG00000057723	Krt33b	keratin 33B [Source:MGI Symbol;Acc:MGI:1309991]	0.119198381	-3.06856345	3.48E-13	4.39E-11	yes	down
ENSMUSG00000027985	Lef1	lymphoid enhancer binding factor 1 [Source:MGI	0.173725655	-2.525117271	3.62E-13	4.54E-11	yes	down
ENSMUSG00000028940	Hes2	hes family bHLH transcription factor 2 [Source:MGI Symbol;Acc:MGI:1098624]	0.132576228	-2.915105983	6.35E-13	7.90E-11	yes	down

ENSMUSG00000053897	Slc39a8	solute carrier family 39 (metal ion transporter), member 8 [Source:MGI Symbol;Acc:MGI:1914797]	0.202725096	-2.302403398	7.89E-13	9.74E-11	yes	down
ENSMUSG00000047501	Cldn4	claudin 4 [Source:MGI Symbol;Acc:MGI:1313314]	0.272103248	-1.877773919	1.04E-12	1.28E-10	yes	down
ENSMUSG00000012123	Crybg2	crystallin beta-gamma domain containing 2 [Source:MGI Symbol;Acc:MGI:1334463]	0.463057677	-1.110736192	1.10E-12	1.34E-10	yes	down
ENSMUSG00000054753	AU018091	expressed sequence AU018091 [Source:MGI Symbol;Acc:MGI:2142124]	0.171658851	-2.54238385	1.13E-12	1.36E-10	yes	down
ENSMUSG00000019960	Dusp6	dual specificity phosphatase 6 [Source:MGI Symbol;Acc:MGI:1914853]	0.461390855	-1.115938687	1.20E-12	1.44E-10	yes	down
ENSMUSG00000068073	1110025L11Rik	RIKEN cDNA 1110025L11 gene [Source:MGI Symbol;Acc:MGI:1915887]	0.136254726	-2.87562182	1.31E-12	1.56E-10	yes	down
ENSMUSG00000027579	Srms	src-related kinase lacking C-terminal regulatory tyrosine and N-terminal myristylation sites [Source:MGI Symbol;Acc:MGI:101865]	0.206665062	-2.274633581	1.42E-12	1.68E-10	yes	down
ENSMUSG00000055717	Slain1	SLAIN motif family, member 1 [Source:MGI Symbol;Acc:MGI:2145578]	0.205037448	-2.286040667	1.42E-12	1.68E-10	yes	down
ENSMUSG00000006345	Ggt1	gamma-glutamyltransferase 1 [Source:MGI	0.139817694	-2.838381147	1.53E-12	1.79E-10	yes	down
ENSMUSG00000034226	Rhov	ras homolog family member V [Source:MGI Symbol;Acc:MGI:2444227]	0.431277215	-1.213312596	1.67E-12	1.93E-10	yes	down
ENSMUSG00000054252	Fgfr3	fibroblast growth factor receptor 3 [Source:MGI	0.402177075	-1.314097247	1.90E-12	2.18E-10	yes	down
ENSMUSG00000089731	Gm10013	predicted gene 10013 [Source:MGI Symbol;Acc:MGI:3704303]	0.079346678	-3.655686365	1.91E-12	2.18E-10	yes	down
ENSMUSG00000011632	Pinlyp	phospholipase A2 inhibitor and LY6/PLAUR domain containing [Source:MGI Symbol;Acc:MGI:3615324]	0.19128838	-2.386178854	2.27E-12	2.56E-10	yes	down
ENSMUSG00000020099	Unc5b	unc-5 netrin receptor B [Source:MGI Symbol;Acc:MGI:894703]	0.477280789	-1.067089828	2.35E-12	2.64E-10	yes	down
ENSMUSG00000028862	Map3k6	mitogen-activated protein kinase kinase kinase 6 [Source:MGI Symbol;Acc:MGI:1855691]	0.399515804	-1.323675522	2.46E-12	2.74E-10	yes	down
ENSMUSG00000063767	S100a7a	S100 calcium binding protein A7A [Source:MGI Symbol;Acc:MGI:2687194]	0.131676798	-2.924926936	2.58E-12	2.86E-10	yes	down
ENSMUSG00000016028	Celsr1	cadherin, EGF LAG seven-pass G-type receptor 1 [Source:MGI Symbol;Acc:MGI:1100883]	0.46458648	-1.105980924	2.68E-12	2.95E-10	yes	down
ENSMUSG00000035557	Krt17	keratin 17 [Source:MGI Symbol;Acc:MGI:96691]	0.333788206	-1.582995113	2.90E-12	3.17E-10	yes	down
ENSMUSG00000093954	Gm16867	predicted gene, 16867 [Source:MGI Symbol;Acc:MGI:4439791]	0.251846417	-1.989383887	3.06E-12	3.32E-10	yes	down
ENSMUSG00000068876	Cgn	cingulin [Source:MGI Symbol;Acc:MGI:1927237]	0.393141899	-1.346877969	3.07E-12	3.32E-10	yes	down
ENSMUSG00000023274	Cd4	CD4 antigen [Source:MGI Symbol;Acc:MGI:88335]	2.261777512	1.17745702	3.13E-12	3.36E-10	yes	up
ENSMUSG00000055926	Gm14137	predicted gene 14137 [Source:MGI Symbol;Acc:MGI:3651144]	0.297487151	-1.749100736	4.63E-12	4.94E-10	yes	down
ENSMUSG00000070385	Ampd1	adenosine monophosphate deaminase 1 [Source:MGI	5.080993387	2.345110586	4.68E-12	4.97E-10	yes	up
ENSMUSG00000024812	Tjp2	tight junction protein 2 [Source:MGI Symbol;Acc:MGI:1341872]	0.435804358	-1.198247472	4.84E-12	5.10E-10	yes	down
ENSMUSG00000037185	Krt80	keratin 80 [Source:MGI Symbol;Acc:MGI:1921377]	0.464236678	-1.107067585	5.08E-12	5.32E-10	yes	down
ENSMUSG00000062433	Krtap6-2	keratin associated protein 6-2 [Source:MGI Symbol;Acc:MGI:1330280]	0.139119388	-2.845604603	5.89E-12	6.13E-10	yes	down
ENSMUSG00000066100	Krtap5-1	keratin associated protein 5-1 [Source:MGI Symbol;Acc:MGI:1354732]	0.092754256	-3.430442707	6.28E-12	6.47E-10	yes	down
ENSMUSG00000046490	Rnf222	ring finger protein 222 [Source:MGI Symbol;Acc:MGI:2443227]	0.219282336	-2.189138492	6.29E-12	6.47E-10	yes	down
ENSMUSG00000071361	Mcpt9	mast cell protease 9 [Source:MGI Symbol;Acc:MGI:1194491]	48.97369202	5.613935057	6.45E-12	6.59E-10	yes	up
ENSMUSG00000078259	Gm11554	predicted gene 11554 [Source:MGI Symbol;Acc:MGI:3705237]	0.098994534	-3.336507325	6.57E-12	6.69E-10	yes	down
ENSMUSG00000050953	Gja1	gap junction protein, alpha 1 [Source:MGI	0.355346031	-1.492703509	7.50E-12	7.56E-10	yes	down
ENSMUSG00000006574	Slc4a1	solute carrier family 4 (anion exchanger), member 1 [Source:MGI Symbol;Acc:MGI:109393]	0.160657164	-2.637942779	7.52E-12	7.56E-10	yes	down
ENSMUSG00000031543	Ank1	ankyrin 1, erythroid [Source:MGI Symbol;Acc:MGI:88024]	3.221610741	1.687782187	8.66E-12	8.65E-10	yes	up
ENSMUSG00000037846	Rtkn2	rhotekin 2 [Source:MGI Symbol;Acc:MGI:2158417]	0.201956382	-2.307884361	1.22E-11	1.22E-09	yes	down

ENSMUSG00000095079	Igha	immunoglobulin heavy constant alpha [Source:MGI	2.497017697	1.320206046	1.37E-11	1.35E-09	yes	up
ENSMUSG00000021508	Cxcl14	chemokine (C-X-C motif) ligand 14 [Source:MGI Symbol;Acc:MGI:1888514]	0.43944461	-1.186246764	1.46E-11	1.43E-09	yes	down
ENSMUSG00000006445	Epha2	Eph receptor A2 [Source:MGI Symbol;Acc:MGI:95278]	0.496265505	-1.010815916	1.49E-11	1.46E-09	yes	down
ENSMUSG00000024232	Bambi	BMP and activin membrane-bound inhibitor [Source:MGI Symbol;Acc:MGI:1915260]	0.197559879	-2.339638102	1.66E-11	1.61E-09	yes	down
ENSMUSG00000037868	Egr2	early growth response 2 [Source:MGI Symbol;Acc:MGI:95296]	0.474717623	-1.074858487	1.80E-11	1.73E-09	yes	down
ENSMUSG00000028633	Ctps	cytidine 5'-triphosphate synthase [Source:MGI Symbol;Acc:MGI:1858304]	0.280422326	-1.834326879	1.85E-11	1.77E-09	yes	down
ENSMUSG00000056885	Gm4559	predicted gene 4559 [Source:MGI Symbol;Acc:MGI:3782743]	0.069515748	-3.846516354	2.02E-11	1.92E-09	yes	down
ENSMUSG00000027993	Trim2	tripartite motif-containing 2 [Source:MGI Symbol;Acc:MGI:1933163]	0.440268869	-1.183543259	2.08E-11	1.97E-09	yes	down
ENSMUSG00000027015	Cybrd1	cytochrome b reductase 1 [Source:MGI	0.246313793	-2.021430677	2.27E-11	2.14E-09	yes	down
ENSMUSG00000079278	Tmem233	transmembrane protein 233 [Source:MGI Symbol;Acc:MGI:3651514]	4.941173481	2.304853709	2.63E-11	2.45E-09	yes	up
ENSMUSG00000034159	Mab21l4	mab-21-like 4 [Source:MGI Symbol;Acc:MGI:1919124]	0.175925328	-2.506964896	2.69E-11	2.50E-09	yes	down
ENSMUSG00000026638	Irf6	interferon regulatory factor 6 [Source:MGI Symbol;Acc:MGI:1859211]	0.466152668	-1.101125569	2.99E-11	2.76E-09	yes	down
ENSMUSG00000075707	Dio3	deiodinase, iodothyronine type III [Source:MGI Symbol;Acc:MGI:1306782]	0.16617731	-2.589204685	3.31E-11	3.05E-09	yes	down
ENSMUSG00000042045	Sln	sarcolipin [Source:MGI Symbol;Acc:MGI:1913652]	3.894163276	1.961313375	4.73E-11	4.33E-09	yes	up
ENSMUSG00000020327	Fgf22	fibroblast growth factor 22 [Source:MGI Symbol;Acc:MGI:1914362]	0.247228448	-2.016083336	4.82E-11	4.38E-09	yes	down
ENSMUSG00000018849	Wwc1	WW, C2 and coiled-coil domain containing 1 [Source:MGI Symbol;Acc:MGI:2388637]	0.42376182	-1.238674485	4.88E-11	4.42E-09	yes	down
ENSMUSG00000024011	Pi16	peptidase inhibitor 16 [Source:MGI Symbol;Acc:MGI:1921366]	3.598789114	1.847511564	5.06E-11	4.56E-09	yes	up
ENSMUSG00000035606	Ky	kyphoscoliosis peptidase [Source:MGI	5.233587059	2.387800097	5.22E-11	4.68E-09	yes	up
ENSMUSG00000021567	Nkd2	naked cuticle 2 [Source:MGI Symbol;Acc:MGI:1919543]	0.445467592	-1.166607615	5.51E-11	4.91E-09	yes	down
ENSMUSG00000069584	Gm10272	predicted gene 10272 [Source:MGI Symbol;Acc:MGI:3642183]	0.059684765	-4.066493474	6.33E-11	5.61E-09	yes	down
ENSMUSG00000037362	Ccn3	cellular communication network factor 3 [Source:MGI Symbol;Acc:MGI:109185]	3.189607557	1.673378929	6.44E-11	5.68E-09	yes	up
ENSMUSG00000003469	Phyhip	phytanoyl-CoA hydroxylase interacting protein [Source:MGI Symbol;Acc:MGI:1860417]	0.40037618	-1.320571951	6.83E-11	5.99E-09	yes	down
ENSMUSG00000058725	Gm11937	predicted gene 11937 [Source:MGI Symbol;Acc:MGI:3651231]	0.113142151	-3.143791591	6.86E-11	5.99E-09	yes	down
ENSMUSG00000049436	Upk1b	uroplakin 1B [Source:MGI Symbol;Acc:MGI:98912]	0.098599484	-3.342276088	7.30E-11	6.34E-09	yes	down
ENSMUSG00000043782	Bicdl2	BICD family like cargo adaptor 2 [Source:MGI Symbol;Acc:MGI:2388267]	0.285801792	-1.806913135	7.33E-11	6.34E-09	yes	down
ENSMUSG00000079470	Utp14b	UTP14B small subunit processome component [Source:MGI Symbol;Acc:MGI:2445092]	0.327106493	-1.612167696	7.85E-11	6.75E-09	yes	down
ENSMUSG00000075602	Ly6a	lymphocyte antigen 6 complex, locus A [Source:MGI Symbol;Acc:MGI:107527]	2.094081357	1.066317493	8.13E-11	6.96E-09	yes	up
ENSMUSG00000030825	Hsd17b14	hydroxysteroid (17-beta) dehydrogenase 14 [Source:MGI Symbol;Acc:MGI:1913315]	0.130086825	-2.942453235	8.20E-11	6.98E-09	yes	down
ENSMUSG00000095794	Igkv6-17	immunoglobulin kappa variable 6-17 [Source:MGI Symbol;Acc:MGI:1330833]	3.148592409	1.65470701	8.29E-11	7.03E-09	yes	up
ENSMUSG00000046694	Tent5b	terminal nucleotidyltransferase 5B [Source:MGI Symbol;Acc:MGI:2140500]	0.318523043	-1.650530352	9.64E-11	8.13E-09	yes	down
ENSMUSG00000033644	Piwil2	piwi-like RNA-mediated gene silencing 2 [Source:MGI Symbol;Acc:MGI:1930036]	0.475592884	-1.072200965	1.04E-10	8.72E-09	yes	down
ENSMUSG00000036098	Myrf	myelin regulatory factor [Source:MGI Symbol;Acc:MGI:2684944]	0.12721802	-2.974625059	1.15E-10	9.58E-09	yes	down
ENSMUSG00000029999	Tgfa	transforming growth factor alpha [Source:MGI	0.350433083	-1.512789114	1.28E-10	1.06E-08	yes	down
ENSMUSG00000039385	Cdh6	cadherin 6 [Source:MGI Symbol;Acc:MGI:107435]	0.217212582	-2.202820422	1.52E-10	1.26E-08	yes	down

ENSMUSG00000026582	Sele	selectin, endothelial cell [Source:MGI Symbol;Acc:MGI:98278]	2.744199049	1.45638513	1.56E-10	1.28E-08	yes	up
ENSMUSG00000038292	Kash5	KASH domain containing 5 [Source:MGI Symbol;Acc:MGI:2687329]	0.083258182	-3.586264139	1.59E-10	1.30E-08	yes	down
ENSMUSG00000055027	Smyd1	SET and MYND domain containing 1 [Source:MGI Symbol;Acc:MGI:104790]	2.860525165	1.516280036	1.68E-10	1.37E-08	yes	up
ENSMUSG00000044813	Shb	src homology 2 domain-containing transforming protein B [Source:MGI Symbol;Acc:MGI:98294]	0.48855266	-1.033414018	1.70E-10	1.38E-08	yes	down
ENSMUSG00000021798	Ldb3	LIM domain binding 3 [Source:MGI Symbol;Acc:MGI:1344412]	2.564464388	1.358657537	1.99E-10	1.60E-08	yes	up
ENSMUSG00000050359	Sprr1a	small proline-rich protein 1A [Source:MGI Symbol;Acc:MGI:106660]	0.33702097	-1.569089733	1.99E-10	1.60E-08	yes	down
ENSMUSG00000095593	Gm7138	predicted gene 7138 [Source:MGI Symbol;Acc:MGI:3779678]	0.035388878	-4.820560184	2.12E-10	1.69E-08	yes	down
ENSMUSG00000027186	Elf5	E74-like factor 5 [Source:MGI Symbol;Acc:MGI:1335079]	0.146164835	-2.774331836	2.13E-10	1.69E-08	yes	down
ENSMUSG00000078254	Krtap29-1	keratin associated protein 29-1 [Source:MGI Symbol;Acc:MGI:3652056]	0.112283732	-3.154779171	2.25E-10	1.78E-08	yes	down
ENSMUSG00000001247	Lsr	lipolysis stimulated lipoprotein receptor [Source:MGI Symbol;Acc:MGI:1927471]	0.397379559	-1.331410431	2.29E-10	1.80E-08	yes	down
ENSMUSG00000060586	H2-Eb1	histocompatibility 2, class II antigen E beta [Source:MGI Symbol;Acc:MGI:95901]	2.430924736	1.281505227	2.30E-10	1.81E-08	yes	up
ENSMUSG00000046352	Gjb2	gap junction protein, beta 2 [Source:MGI	0.269958982	-1.889187876	2.34E-10	1.83E-08	yes	down
ENSMUSG00000062380	Tubb3	tubulin, beta 3 class III [Source:MGI Symbol;Acc:MGI:107813]	0.368774564	-1.439188945	3.22E-10	2.51E-08	yes	down
ENSMUSG00000054537	Tmprss11e	transmembrane protease, serine 11e [Source:MGI Symbol;Acc:MGI:3513175]	0.344204033	-1.538664093	3.28E-10	2.54E-08	yes	down
ENSMUSG00000049872	Calhm5	calcium homeostasis modulator family member 5 [Source:MGI Symbol;Acc:MGI:2143897]	0.372093841	-1.426261583	3.50E-10	2.70E-08	yes	down
ENSMUSG00000042745	Id1	inhibitor of DNA binding 1, HLH protein [Source:MGI Symbol;Acc:MGI:96396]	0.484566534	-1.045233326	3.60E-10	2.77E-08	yes	down
ENSMUSG00000018648	Dusp14	dual specificity phosphatase 14 [Source:MGI Symbol;Acc:MGI:1927168]	0.26242379	-1.930029582	3.87E-10	2.96E-08	yes	down
ENSMUSG00000007122	Casq1	calsequestrin 1 [Source:MGI Symbol;Acc:MGI:1309468]	3.448473646	1.785957941	4.18E-10	3.18E-08	yes	up
ENSMUSG00000050201	Otop2	otopetrin 2 [Source:MGI Symbol;Acc:MGI:2388365]	0.122400132	-3.030322975	4.79E-10	3.62E-08	yes	down
ENSMUSG00000040389	Wdr47	WD repeat domain 47 [Source:MGI Symbol;Acc:MGI:2139593]	0.408014612	-1.293307275	4.80E-10	3.62E-08	yes	down
ENSMUSG00000028444	Cntfr	ciliary neurotrophic factor receptor [Source:MGI	0.410162607	-1.285732124	4.89E-10	3.68E-08	yes	down
ENSMUSG00000032724	Abtb2	ankyrin repeat and BTB (POZ) domain containing 2 [Source:MGI Symbol;Acc:MGI:2139365]	0.435405298	-1.199569133	5.11E-10	3.82E-08	yes	down
ENSMUSG00000025986	Slc39a10	solute carrier family 39 (zinc transporter), member 10 [Source:MGI Symbol;Acc:MGI:1914515]	0.368294309	-1.441068991	5.80E-10	4.32E-08	yes	down
ENSMUSG00000031785	Adgrg1	adhesion G protein-coupled receptor G1 [Source:MGI Symbol;Acc:MGI:1340051]	0.427398356	-1.226346736	6.03E-10	4.47E-08	yes	down
ENSMUSG00000090066	1110002E22Rik	RIKEN cDNA 1110002E22 gene [Source:MGI Symbol;Acc:MGI:1915066]	3.320913178	1.731580005	7.13E-10	5.23E-08	yes	up
ENSMUSG00000026205	Slc23a3	solute carrier family 23 (nucleobase transporters), member 3 [Source:MGI Symbol;Acc:MGI:104516]	0.178123415	-2.489050917	7.16E-10	5.23E-08	yes	down
ENSMUSG00000015957	Wnt11	wingless-type MMTV integration site family, member 11 [Source:MGI Symbol;Acc:MGI:101948]	0.379484683	-1.397886439	7.17E-10	5.23E-08	yes	down
ENSMUSG00000041313	Slc7a1	solute carrier family 7 (cationic amino acid transporter, y+ system), member 1 [Source:MGI	0.496415248	-1.010380665	7.17E-10	5.23E-08	yes	down
ENSMUSG00000029859	Epha1	Eph receptor A1 [Source:MGI Symbol;Acc:MGI:107381]	0.416417661	-1.263896836	7.75E-10	5.63E-08	yes	down
ENSMUSG00000047281	Sfn	stratifin [Source:MGI Symbol;Acc:MGI:1891831]	0.438102311	-1.190660269	7.94E-10	5.74E-08	yes	down
ENSMUSG00000034112	Atp2c2	ATPase, Ca++ transporting, type 2C, member 2 [Source:MGI Symbol;Acc:MGI:1916297]	0.312251589	-1.67921918	8.11E-10	5.84E-08	yes	down
ENSMUSG00000022658	Tagln3	transgelin 3 [Source:MGI Symbol;Acc:MGI:1926784]	0.112664045	-3.149900915	8.22E-10	5.90E-08	yes	down
ENSMUSG00000006462	A530013C23Rik	RIKEN cDNA A530013C23 gene [Source:MGI Symbol;Acc:MGI:3041178]	0.315994743	-1.662027539	8.73E-10	6.24E-08	yes	down

ENSMUSG00000087141	Plcxd2	phosphatidylinositol-specific phospholipase C, X domain containing 2 [Source:MGI Symbol;Acc:MGI:3647874]	0.316428532	-1.660048402	8.88E-10	6.32E-08	yes	down
ENSMUSG00000016756	Cmah	cytidine monophospho-N-acetylneuraminic acid hydroxylase [Source:MGI Symbol;Acc:MGI:103227]	0.256218446	-1.964553752	8.96E-10	6.35E-08	yes	down
ENSMUSG00000090515	Krtap27-1	keratin associated protein 27-1 [Source:MGI Symbol;Acc:MGI:3646229]	0.088550543	-3.49735503	9.64E-10	6.80E-08	yes	down
ENSMUSG00000059970	Hspa2	heat shock protein 2 [Source:MGI Symbol;Acc:MGI:96243]	0.396914046	-1.333101479	9.94E-10	6.99E-08	yes	down
ENSMUSG00000021565	Slc6a19	solute carrier family 6 (neurotransmitter transporter), member 19 [Source:MGI	0.22956395	-2.12303199	1.08E-09	7.53E-08	yes	down
ENSMUSG00000027368	Dusp2	dual specificity phosphatase 2 [Source:MGI Symbol;Acc:MGI:101911]	0.248073892	-2.011158187	1.13E-09	7.88E-08	yes	down
ENSMUSG00000044243	Bhlha9	basic helix-loop-helix family, member a9 [Source:MGI Symbol;Acc:MGI:2444198]	0.107781262	-3.213821712	1.15E-09	7.95E-08	yes	down
ENSMUSG00000027315	Spint1	serine protease inhibitor, Kunitz type 1 [Source:MGI Symbol;Acc:MGI:1338033]	0.418035354	-1.258303138	1.22E-09	8.39E-08	yes	down
ENSMUSG00000025931	Paqr8	progestin and adipoQ receptor family member VIII [Source:MGI Symbol;Acc:MGI:1921479]	0.317011164	-1.657394448	1.25E-09	8.59E-08	yes	down
ENSMUSG00000006777	Krt23	keratin 23 [Source:MGI Symbol;Acc:MGI:2148866]	0.332385794	-1.589069372	1.28E-09	8.76E-08	yes	down
ENSMUSG00000062694	Cav3	caveolin 3 [Source:MGI Symbol;Acc:MGI:107570]	5.008887482	2.324490204	1.38E-09	9.39E-08	yes	up
ENSMUSG00000021678	F2rl1	coagulation factor II (thrombin) receptor-like 1 [Source:MGI Symbol;Acc:MGI:101910]	0.414044398	-1.272142619	1.45E-09	9.80E-08	yes	down
ENSMUSG00000018830	Myh11	myosin, heavy polypeptide 11, smooth muscle [Source:MGI Symbol;Acc:MGI:102643]	2.037963605	1.027128287	1.50E-09	1.01E-07	yes	up
ENSMUSG00002074934			0.201334252	-2.312335462	1.57E-09	1.06E-07	yes	down
ENSMUSG00000030672	Mylpf	myosin light chain, phosphorylatable, fast skeletal muscle [Source:MGI Symbol;Acc:MGI:97273]	2.727945251	1.44781469	1.61E-09	1.08E-07	yes	up
ENSMUSG00000024975	Pdcd4	programmed cell death 4 [Source:MGI	0.448772002	-1.155945424	1.69E-09	1.13E-07	yes	down
ENSMUSG00000055725	Paqr3	progestin and adipoQ receptor family member III [Source:MGI Symbol;Acc:MGI:2679683]	0.338224421	-1.563947265	1.71E-09	1.13E-07	yes	down
ENSMUSG00000046845	Il1f10	interleukin 1 family, member 10 [Source:MGI Symbol;Acc:MGI:2652548]	0.106228808	-3.234753029	2.04E-09	1.35E-07	yes	down
ENSMUSG00000046761	Fam83h	family with sequence similarity 83, member H [Source:MGI Symbol;Acc:MGI:2145900]	0.496469862	-1.010221952	2.05E-09	1.35E-07	yes	down
ENSMUSG00000100626	H2al1a	H2A histone family member L1A [Source:MGI Symbol;Acc:MGI:3714114]	0.004112027	-7.925934496	2.12E-09	1.39E-07	yes	down
ENSMUSG00000031351	Zfp185	zinc finger protein 185 [Source:MGI Symbol;Acc:MGI:108095]	0.419655613	-1.252722219	2.13E-09	1.40E-07	yes	down
ENSMUSG00000048450	Msx1	msh homeobox 1 [Source:MGI Symbol;Acc:MGI:97168]	0.297411657	-1.749466898	2.34E-09	1.53E-07	yes	down
ENSMUSG00000041782	Lad1	ladinin [Source:MGI Symbol;Acc:MGI:109343]	0.473119141	-1.079724566	2.40E-09	1.56E-07	yes	down
ENSMUSG00000038403	Hjv	hemojuvelin BMP co-receptor [Source:MGI Symbol;Acc:MGI:1916835]	3.861190829	1.949045857	2.46E-09	1.59E-07	yes	up
ENSMUSG00000095721	Gm7137	predicted gene 7137 [Source:MGI Symbol;Acc:MGI:3779677]	0.063127914	-3.985578113	2.49E-09	1.60E-07	yes	down
ENSMUSG00000022562	Oplah	5-oxoprolinase (ATP-hydrolysing) [Source:MGI Symbol;Acc:MGI:1922725]	0.497856044	-1.00619945	2.65E-09	1.70E-07	yes	down
ENSMUSG00000020062	Slc5a8	solute carrier family 5 (iodide transporter), member 8 [Source:MGI Symbol;Acc:MGI:2384916]	0.410513087	-1.284499879	2.78E-09	1.78E-07	yes	down
ENSMUSG00000112236	Gm48180	predicted gene, 48180 [Source:MGI Symbol;Acc:MGI:6097556]	0.063672762	-3.973179838	2.81E-09	1.79E-07	yes	down
ENSMUSG00000024270	Slc39a6	solute carrier family 39 (metal ion transporter), member 6 [Source:MGI Symbol;Acc:MGI:2147279]	0.468014485	-1.095374913	2.93E-09	1.86E-07	yes	down
ENSMUSG00000020871	Dlx4	distal-less homeobox 4 [Source:MGI Symbol;Acc:MGI:94904]	0.132585242	-2.915007894	2.93E-09	1.86E-07	yes	down
ENSMUSG00000040118	Cacna2d1	calcium channel, voltage-dependent, alpha2/delta subunit 1 [Source:MGI Symbol;Acc:MGI:88295]	2.307836643	1.206541109	2.95E-09	1.86E-07	yes	up

ENSMUSG00000040694	Apobec2	apolipoprotein B mRNA editing enzyme, catalytic polypeptide 2 [Source:MGI Symbol;Acc:MGI:1343178]	4.173088484	2.061115511	3.09E-09	1.94E-07	yes	up
ENSMUSG00000026407	Cacna1s	calcium channel, voltage-dependent, L type, alpha 1S subunit [Source:MGI Symbol;Acc:MGI:88294]	3.067045938	1.616849775	3.17E-09	1.98E-07	yes	up
ENSMUSG00000000938	Hoxa10	homeobox A10 [Source:MGI Symbol;Acc:MGI:96171]	0.243201643	-2.039775118	3.20E-09	1.99E-07	yes	down
ENSMUSG00000031936	Hephl1	hephaestin-like 1 [Source:MGI Symbol;Acc:MGI:2685355]	0.26948307	-1.891733454	3.42E-09	2.12E-07	yes	down
ENSMUSG00000034282	Evpl	envoplakin [Source:MGI Symbol;Acc:MGI:107507]	0.468875032	-1.092724638	4.07E-09	2.51E-07	yes	down
ENSMUSG00000041476	Smpx	small muscle protein, X-linked [Source:MGI Symbol;Acc:MGI:1913356]	3.246952464	1.699086263	4.54E-09	2.78E-07	yes	up
ENSMUSG00000017300	Tnnc2	troponin C2, fast [Source:MGI Symbol;Acc:MGI:98780]	2.794407557	1.482542449	4.54E-09	2.78E-07	yes	up
ENSMUSG00000021062	Rab15	RAB15, member RAS oncogene family [Source:MGI Symbol;Acc:MGI:1916865]	0.389162805	-1.361554265	4.88E-09	2.98E-07	yes	down
ENSMUSG00000051910	Sox6	SRY (sex determining region Y)-box 6 [Source:MGI	2.465863664	1.302093036	5.12E-09	3.12E-07	yes	up
ENSMUSG00000021638	Ocln	occludin [Source:MGI Symbol;Acc:MGI:106183]	0.430221854	-1.216847282	5.44E-09	3.30E-07	yes	down
ENSMUSG00000039682	Lap3	leucine aminopeptidase 3 [Source:MGI	0.386692575	-1.370741033	5.75E-09	3.47E-07	yes	down
ENSMUSG00000036594	H2-Aa	histocompatibility 2, class II antigen A, alpha [Source:MGI Symbol;Acc:MGI:95895]	2.311538636	1.208853477	5.84E-09	3.51E-07	yes	up
ENSMUSG00000039578	Ccser1	coiled-coil serine rich 1 [Source:MGI Symbol;Acc:MGI:3045354]	0.369392818	-1.436772279	5.84E-09	3.51E-07	yes	down
ENSMUSG00000038580	Sct	secretin [Source:MGI Symbol;Acc:MGI:99466]	0.087289157	-3.518053744	6.25E-09	3.74E-07	yes	down
ENSMUSG00000006411	Nectin4	nectin cell adhesion molecule 4 [Source:MGI Symbol;Acc:MGI:1918990]	0.428642441	-1.222153394	6.28E-09	3.74E-07	yes	down
ENSMUSG00000040703	Cyp2s1	cytochrome P450, family 2, subfamily s, polypeptide 1 [Source:MGI Symbol;Acc:MGI:1921384]	0.398358283	-1.327861523	6.61E-09	3.92E-07	yes	down
ENSMUSG00000057829	Gm5278	predicted pseudogene 5278 [Source:MGI Symbol;Acc:MGI:3643701]	0.124188993	-3.009390788	6.79E-09	4.02E-07	yes	down
ENSMUSG00000109592	Gm45417	predicted gene 45417 [Source:MGI Symbol;Acc:MGI:5791253]	0.05212487	-4.261884299	7.01E-09	4.13E-07	yes	down
ENSMUSG00000046733	Gprc5a	G protein-coupled receptor, family C, group 5, member A [Source:MGI Symbol;Acc:MGI:1891250]	0.198727955	-2.331133267	7.13E-09	4.19E-07	yes	down
ENSMUSG00000019787	Trdn	triadin [Source:MGI Symbol;Acc:MGI:1924007]	2.114427964	1.080267411	7.56E-09	4.41E-07	yes	up
ENSMUSG00000022449	Adamts20	a disintegrin-like and metallopeptidase (reprolysin type) with thrombospondin type 1 motif, 20 [Source:MGI	0.269993397	-1.88900397	7.74E-09	4.50E-07	yes	down
ENSMUSG00000069873	4930438A08Rik	RIKEN cDNA 4930438A08 gene [Source:MGI Symbol;Acc:MGI:1921238]	0.261602173	-1.934553568	8.57E-09	4.97E-07	yes	down
ENSMUSG00000025993	Slc40a1	solute carrier family 40 (iron-regulated transporter), member 1 [Source:MGI	0.414659827	-1.269999811	8.98E-09	5.19E-07	yes	down
ENSMUSG00000094146	Gm10142	predicted gene 10142 [Source:MGI Symbol;Acc:MGI:3641725]	0.065749918	-3.926867086	9.55E-09	5.50E-07	yes	down
ENSMUSG00000031075	Ano1	anoctamin 1, calcium activated chloride channel [Source:MGI Symbol;Acc:MGI:2142149]	2.279658864	1.18881795	9.58E-09	5.50E-07	yes	up
ENSMUSG00000028024	Enpep	glutamyl aminopeptidase [Source:MGI	2.472449159	1.305940855	1.00E-08	5.75E-07	yes	up
ENSMUSG00000073421	H2-Ab1	histocompatibility 2, class II antigen A, beta 1 [Source:MGI Symbol;Acc:MGI:103070]	2.266098716	1.180210709	1.11E-08	6.32E-07	yes	up
ENSMUSG00000036867	Smad6	SMAD family member 6 [Source:MGI	0.434564919	-1.202356379	1.20E-08	6.79E-07	yes	down
ENSMUSG00000069717	Gm11568	predicted gene 11568 [Source:MGI Symbol;Acc:MGI:3650331]	0.079658535	-3.650027248	1.21E-08	6.82E-07	yes	down
ENSMUSG00000073877	Gm13306	predicted gene 13306 [Source:MGI Symbol;Acc:MGI:3713752]	4.684112994	2.227775878	1.25E-08	7.07E-07	yes	up
ENSMUSG00000024164	C3	complement component 3 [Source:MGI	2.641556707	1.401388381	1.28E-08	7.18E-07	yes	up
ENSMUSG00000018339	Gpx3	glutathione peroxidase 3 [Source:MGI Symbol;Acc:MGI:105102]	2.512846339	1.329322453	1.28E-08	7.18E-07	yes	up
ENSMUSG00000024617	Camk2a	calcium/calmodulin-dependent protein kinase II alpha [Source:MGI Symbol;Acc:MGI:88256]	2.59473979	1.375589867	1.52E-08	8.46E-07	yes	up
ENSMUSG00000005716	Pvalb	parvalbumin [Source:MGI Symbol;Acc:MGI:97821]	3.454616098	1.788525397	1.53E-08	8.52E-07	yes	up

ENSMUSG00000031451	Gas6	growth arrest specific 6 [Source:MGI Symbol;Acc:MGI:95660]	2.067798575	1.048095659	1.62E-08	8.90E-07	yes	up
ENSMUSG00000022519	Srl	sarcalumenin [Source:MGI Symbol;Acc:MGI:2146620]	2.361148296	1.239488655	1.62E-08	8.90E-07	yes	up
ENSMUSG00000024411	Aqp4	aquaporin 4 [Source:MGI Symbol;Acc:MGI:107387]	4.443009453	2.151537211	1.64E-08	8.98E-07	yes	up
ENSMUSG00000038521	C1s1	complement component 1, s subcomponent 1 [Source:MGI Symbol;Acc:MGI:1355312]	2.349884548	1.232589877	1.66E-08	9.06E-07	yes	up
ENSMUSG00000100190	Krtap28-10	keratin associated protein 28-10 [Source:MGI Symbol;Acc:MGI:3779575]	0.06357149	-3.975476294	1.67E-08	9.06E-07	yes	down
ENSMUSG00000076552	Igkv4-61	immunoglobulin kappa chain variable 4-61 [Source:MGI Symbol;Acc:MGI:4439819]	4.926002001	2.300417214	1.71E-08	9.26E-07	yes	up
ENSMUSG00000024039	Cbs	cystathionine beta-synthase [Source:MGI	0.25203234	-1.98831923	1.80E-08	9.74E-07	yes	down
ENSMUSG00000071471	Krtap26-1	keratin associated protein 26-1 [Source:MGI Symbol;Acc:MGI:1916783]	0.077604984	-3.687706882	1.83E-08	9.83E-07	yes	down
ENSMUSG00000032380	Dapk2	death-associated protein kinase 2 [Source:MGI Symbol;Acc:MGI:1341297]	0.401992913	-1.314758027	1.87E-08	9.98E-07	yes	down
ENSMUSG00000032068	Plet1	placenta expressed transcript 1 [Source:MGI Symbol;Acc:MGI:1923759]	0.384645519	-1.378398594	1.94E-08	1.04E-06	yes	down
ENSMUSG00000053007	Creb5	cAMP responsive element binding protein 5 [Source:MGI Symbol;Acc:MGI:2443973]	0.449316632	-1.154195629	1.99E-08	1.06E-06	yes	down
ENSMUSG00000040998	Npnt	nephronectin [Source:MGI Symbol;Acc:MGI:2148811]	2.680685267	1.422601846	2.01E-08	1.07E-06	yes	up
ENSMUSG00000028972	Car6	carbonic anhydrase 6 [Source:MGI Symbol;Acc:MGI:1333786]	0.237024478	-2.07689204	2.04E-08	1.08E-06	yes	down
ENSMUSG00000001901	Kcnh6	potassium voltage-gated channel, subfamily H (eag-related), member 6 [Source:MGI Symbol;Acc:MGI:2684139]	0.440540913	-1.182652085	2.06E-08	1.08E-06	yes	down
ENSMUSG00000056900	Usp13	ubiquitin specific peptidase 13 (isopeptidase T-3) [Source:MGI Symbol;Acc:MGI:1919857]	2.761399776	1.465399767	2.08E-08	1.09E-06	yes	up
ENSMUSG00000026574	Dpt	dermatopontin [Source:MGI Symbol;Acc:MGI:1928392]	2.972340511	1.571599401	2.10E-08	1.10E-06	yes	up
ENSMUSG00000036306	Lzts1	leucine zipper, putative tumor suppressor 1 [Source:MGI Symbol;Acc:MGI:2684762]	0.412439294	-1.277746307	2.11E-08	1.10E-06	yes	down
ENSMUSG00000032523	Hhatl	hedgehog acyltransferase-like [Source:MGI Symbol;Acc:MGI:1922020]	4.205810356	2.072383799	2.13E-08	1.11E-06	yes	up
ENSMUSG00000022512	Cldn1	claudin 1 [Source:MGI Symbol;Acc:MGI:1276109]	0.487055533	-1.037841821	2.16E-08	1.12E-06	yes	down
ENSMUSG00000024610	Cd74	CD74 antigen (invariant polypeptide of major histocompatibility complex, class II antigen-associated) [Source:MGI	2.22060945	1.150955681	2.33E-08	1.20E-06	yes	up
ENSMUSG00000040147	Maob	monoamine oxidase B [Source:MGI Symbol;Acc:MGI:96916]	2.298673012	1.200801256	2.43E-08	1.25E-06	yes	up
ENSMUSG00000041842	Fhdc1	FH2 domain containing 1 [Source:MGI	0.474376907	-1.075894313	2.46E-08	1.26E-06	yes	down
ENSMUSG00000029337	Fgf5	fibroblast growth factor 5 [Source:MGI	0.127595649	-2.970348963	2.47E-08	1.27E-06	yes	down
ENSMUSG00000028403	Zdhhc21	zinc finger, DHHC domain containing 21 [Source:MGI Symbol;Acc:MGI:1915518]	0.437392752	-1.19299878	2.49E-08	1.27E-06	yes	down
ENSMUSG00000048582	Gja3	gap junction protein, alpha 3 [Source:MGI	0.129230382	-2.951982804	2.65E-08	1.35E-06	yes	down
ENSMUSG00000027412	Lpin3	lipin 3 [Source:MGI Symbol;Acc:MGI:1891342]	0.411804768	-1.279967561	2.71E-08	1.38E-06	yes	down
ENSMUSG00000048776	Pthlh	parathyroid hormone-like peptide [Source:MGI	0.23715244	-2.076113385	2.77E-08	1.40E-06	yes	down
ENSMUSG00000083548	Gm11514	predicted gene 11514 [Source:MGI Symbol;Acc:MGI:3649789]	0.045053422	-4.472219487	2.89E-08	1.46E-06	yes	down
ENSMUSG00000059336	Slc14a1	solute carrier family 14 (urea transporter), member 1 [Source:MGI Symbol;Acc:MGI:1351654]	2.989114393	1.57971811	2.94E-08	1.48E-06	yes	up
ENSMUSG00000036923	Stox1	storkhead box 1 [Source:MGI Symbol;Acc:MGI:2684909]	0.162850216	-2.61838246	3.05E-08	1.53E-06	yes	down
ENSMUSG00000034055	Phka1	phosphorylase kinase alpha 1 [Source:MGI	2.462660143	1.300217544	3.45E-08	1.72E-06	yes	up
ENSMUSG00000026185	Igfbp5	insulin-like growth factor binding protein 5 [Source:MGI Symbol;Acc:MGI:96440]	3.37234022	1.753750091	3.56E-08	1.77E-06	yes	up
ENSMUSG00000020592	Sdc1	syndecan 1 [Source:MGI Symbol;Acc:MGI:1349162]	0.46826803	-1.09459355	3.64E-08	1.80E-06	yes	down
ENSMUSG00000075566	Krtap4-6	keratin associated protein 4-6 [Source:MGI Symbol;Acc:MGI:1916018]	0.087866631	-3.508540816	3.67E-08	1.81E-06	yes	down

ENSMUSG000 00035020	Epgn	epithelial mitogen [Source:MGI Symbol;Acc:MGI:1919170]	0.305203229	-1.712157867	3.83E-08	1.89E-06	yes	down
ENSMUSG000 00061462	Obscn	obscurin, cytoskeletal calmodulin and titin-interacting RhoGEF [Source:MGI	2.924591477	1.548235115	4.12E-08	2.02E-06	yes	up
ENSMUSG000 00005338	Cadm3	cell adhesion molecule 3 [Source:MGI	2.879776281	1.525956739	4.32E-08	2.11E-06	yes	up
ENSMUSG000 00054065	Pkp3	plakophilin 3 [Source:MGI Symbol;Acc:MGI:1891830]	0.468970386	-1.09243127	4.33E-08	2.11E-06	yes	down
ENSMUSG000 00020589	Cyria	CYFIP related Rac1 interactor A [Source:MGI Symbol;Acc:MGI:1261783]	0.360566338	-1.471663381	4.35E-08	2.11E-06	yes	down
ENSMUSG000 00032500	Dclk3	doublecortin-like kinase 3 [Source:MGI	0.244697444	-2.030929064	4.39E-08	2.13E-06	yes	down
ENSMUSG000 00101026	Ly6g6g	lymphocyte antigen 6 complex, locus G6G [Source:MGI Symbol;Acc:MGI:1925975]	0.078426931	-3.672507051	4.68E-08	2.26E-06	yes	down
ENSMUSG000 00025537	Phkg1	phosphorylase kinase gamma 1 [Source:MGI	2.809167045	1.490142416	4.72E-08	2.27E-06	yes	up
ENSMUSG000 00021835	Bmp4	bone morphogenetic protein 4 [Source:MGI	0.427697077	-1.225338747	5.14E-08	2.46E-06	yes	down
ENSMUSG000 00039639	Kcne1	potassium voltage-gated channel, Isk-related subfamily, member 1 [Source:MGI	0.103024515	-3.278940419	5.49E-08	2.62E-06	yes	down
ENSMUSG000 00060716	Plekhh1	pleckstrin homology domain containing, family H (with MyTH4 domain) member 1 [Source:MGI Symbol;Acc:MGI:2144989]	0.307067851	-1.70337062	5.69E-08	2.71E-06	yes	down
ENSMUSG000 00017724	Etv4	ets variant 4 [Source:MGI Symbol;Acc:MGI:99423]	0.380484001	-1.394092302	5.76E-08	2.73E-06	yes	down
ENSMUSG000 00043719	Col6a6	collagen, type VI, alpha 6 [Source:MGI	3.07021387	1.618339157	6.11E-08	2.89E-06	yes	up
ENSMUSG000 00031097	Tnni2	troponin I, skeletal, fast 2 [Source:MGI	2.553398054	1.35241846	6.21E-08	2.93E-06	yes	up
ENSMUSG000 00026989	Dapl1	death associated protein-like 1 [Source:MGI Symbol;Acc:MGI:1923997]	0.318991868	-1.64840845	6.40E-08	3.02E-06	yes	down
ENSMUSG000 00004552	Ctse	cathepsin E [Source:MGI Symbol;Acc:MGI:107361]	0.282077012	-1.825838999	6.81E-08	3.20E-06	yes	down
ENSMUSG000 00075012	Fjx1	four jointed box 1 [Source:MGI Symbol;Acc:MGI:1341907]	0.446127207	-1.164472962	6.94E-08	3.25E-06	yes	down
ENSMUSG000 00113973	A030014E15 Rik	RIKEN cDNA A030014E15 gene [Source:MGI Symbol;Acc:MGI:1925167]	0.069079595	-3.855596569	7.60E-08	3.53E-06	yes	down
ENSMUSG000 00113880	A030005L19 Rik	RIKEN cDNA A030005L19 gene [Source:MGI Symbol;Acc:MGI:1925172]	0.069565178	-3.845490862	7.67E-08	3.55E-06	yes	down
ENSMUSG000 00022371	Col14a1	collagen, type XIV, alpha 1 [Source:MGI Symbol;Acc:MGI:1341272]	2.75161299	1.460277572	7.75E-08	3.58E-06	yes	up
ENSMUSG000 00078131	Krtap1-3	keratin associated protein 1-3 [Source:MGI Symbol;Acc:MGI:3650443]	0.090446698	-3.46678835	7.82E-08	3.61E-06	yes	down
ENSMUSG000 00049173	Myoz3	myozenin 3 [Source:MGI Symbol;Acc:MGI:2179296]	4.282351601	2.098403253	8.39E-08	3.85E-06	yes	up
ENSMUSG000 00046167	Gldn	gliomedin [Source:MGI Symbol;Acc:MGI:2388361]	2.971124181	1.571008905	8.73E-08	3.99E-06	yes	up
ENSMUSG000 00021203	Otub2	OTU domain, ubiquitin aldehyde binding 2 [Source:MGI Symbol;Acc:MGI:1915399]	0.414051791	-1.272116859	8.82E-08	4.02E-06	yes	down
ENSMUSG000 00030994	D7Ertd443e	DNA segment, Chr 7, ERATO Doi 443, expressed [Source:MGI Symbol;Acc:MGI:1196431]	0.210922311	-2.245216383	9.10E-08	4.14E-06	yes	down
ENSMUSG000 00032058	Ppp2r1b	protein phosphatase 2, regulatory subunit A, beta [Source:MGI Symbol;Acc:MGI:1920949]	0.485845324	-1.041431011	9.25E-08	4.19E-06	yes	down
ENSMUSG000 00046807	Lrrc75b	leucine rich repeat containing 75B [Source:MGI Symbol;Acc:MGI:2143657]	0.365217079	-1.453173861	9.29E-08	4.19E-06	yes	down
ENSMUSG000 00035592	Krt33a	keratin 33A [Source:MGI Symbol;Acc:MGI:1919138]	0.090587586	-3.464542825	9.50E-08	4.28E-06	yes	down
ENSMUSG000 00024665	Fads2	fatty acid desaturase 2 [Source:MGI Symbol;Acc:MGI:1930079]	0.488735966	-1.032872817	9.93E-08	4.46E-06	yes	down
ENSMUSG000 00025938	Slco5a1	solute carrier organic anion transporter family, member 5A1 [Source:MGI	0.35879156	-1.478782139	1.09E-07	4.86E-06	yes	down
ENSMUSG000 00020102	Slc16a7	solute carrier family 16 (monocarboxylic acid transporters), member 7 [Source:MGI Symbol;Acc:MGI:1330284]	0.442891816	-1.174973757	1.17E-07	5.21E-06	yes	down
ENSMUSG000 00061048	Cdh3	cadherin 3 [Source:MGI Symbol;Acc:MGI:88356]	0.498264201	-1.005017172	1.23E-07	5.45E-06	yes	down
ENSMUSG000 00034248	Slc25a37	solute carrier family 25, member 37 [Source:MGI Symbol;Acc:MGI:1914962]	0.384984501	-1.377127728	1.26E-07	5.57E-06	yes	down

ENSMUSG00000021047	Nova1	NOVA alternative splicing regulator 1 [Source:MGI Symbol;Acc:MGI:104297]	2.964185832	1.567635897	1.26E-07	5.58E-06	yes	up
ENSMUSG00000047889	Serpinb6d	serine (or cysteine) peptidase inhibitor, clade B, member 6d [Source:MGI	7.080971965	2.823947404	1.27E-07	5.58E-06	yes	up
ENSMUSG00000032350	Gclc	glutamate-cysteine ligase, catalytic subunit [Source:MGI Symbol;Acc:MGI:104990]	0.4080927	-1.29303119	1.32E-07	5.82E-06	yes	down
ENSMUSG00000026335	Pam	peptidylglycine alpha-amidating monooxygenase [Source:MGI Symbol;Acc:MGI:97475]	2.157495432	1.109357505	1.34E-07	5.85E-06	yes	up
ENSMUSG00000015405	Ace2	angiotensin I converting enzyme (peptidyl-dipeptidase A) 2 [Source:MGI	0.383470824	-1.38281128	1.34E-07	5.85E-06	yes	down
ENSMUSG00000015852	Fcrls	Fc receptor-like S, scavenger receptor [Source:MGI Symbol;Acc:MGI:1933397]	2.016174446	1.011620471	1.35E-07	5.88E-06	yes	up
ENSMUSG00000043029	Trpv3	transient receptor potential cation channel, subfamily V, member 3 [Source:MGI Symbol;Acc:MGI:2181407]	0.397758022	-1.330037069	1.37E-07	5.96E-06	yes	down
ENSMUSG00000000296	Tpd52l1	tumor protein D52-like 1 [Source:MGI	0.418331321	-1.257282075	1.37E-07	5.97E-06	yes	down
ENSMUSG00000038239	Hrc	histidine rich calcium binding protein [Source:MGI	2.834791516	1.503242636	1.41E-07	6.09E-06	yes	up
ENSMUSG00000091212	Krtap11-1	keratin associated protein 11-1 [Source:MGI	0.083921769	-3.574811103	1.48E-07	6.38E-06	yes	down
ENSMUSG00000022218	Tgm1	transglutaminase 1, K polypeptide [Source:MGI	0.446432502	-1.163486031	1.48E-07	6.40E-06	yes	down
ENSMUSG00000028278	Rragd	Ras-related GTP binding D [Source:MGI Symbol;Acc:MGI:1098604]	2.952159867	1.561770849	1.57E-07	6.74E-06	yes	up
ENSMUSG00000025330	Padi4	peptidyl arginine deiminase, type IV [Source:MGI Symbol;Acc:MGI:1338898]	0.302156492	-1.726632155	1.58E-07	6.79E-06	yes	down
ENSMUSG00000105703	Gm43305	predicted gene 43305 [Source:MGI Symbol;Acc:MGI:5663442]	2.980291464	1.575453429	1.65E-07	7.06E-06	yes	up
ENSMUSG00000029171	Pgm2	phosphoglucomutase 2 [Source:MGI Symbol;Acc:MGI:97564]	0.451247251	-1.148009951	1.66E-07	7.06E-06	yes	down
ENSMUSG00000032883	Acsl3	acyl-CoA synthetase long-chain family member 3 [Source:MGI Symbol;Acc:MGI:1921455]	0.472231339	-1.082434308	1.66E-07	7.07E-06	yes	down
ENSMUSG00000085014	Gm13490	predicted gene 13490 [Source:MGI Symbol;Acc:MGI:3650821]	0.056623699	-4.142450185	1.69E-07	7.18E-06	yes	down
ENSMUSG00000020656	Grhl1	grainyhead like transcription factor 1 [Source:MGI Symbol;Acc:MGI:2182540]	0.457786151	-1.127254276	1.70E-07	7.21E-06	yes	down
ENSMUSG00000025608	Podxl	podocalyxin-like [Source:MGI Symbol;Acc:MGI:1351317]	2.01720041	1.012354424	1.71E-07	7.21E-06	yes	up
ENSMUSG00000051262	Nat8f3	N-acetyltransferase 8 (GCN5-related) family member 3 [Source:MGI Symbol;Acc:MGI:2136449]	3.521670318	1.816259857	1.73E-07	7.31E-06	yes	up
ENSMUSG00000059845	Gm11567	predicted gene 11567 [Source:MGI Symbol;Acc:MGI:3649436]	0.094809662	-3.398822104	1.80E-07	7.57E-06	yes	down
ENSMUSG00000070335	Krtap9-1	keratin associated protein 9-1 [Source:MGI Symbol;Acc:MGI:1309997]	0.099115651	-3.334743308	1.81E-07	7.59E-06	yes	down
ENSMUSG00000069718	Gm11563	predicted gene 11563 [Source:MGI Symbol;Acc:MGI:3650330]	0.094728716	-3.400054355	1.88E-07	7.85E-06	yes	down
ENSMUSG00000029832	Nfe2l3	nuclear factor, erythroid derived 2, like 3 [Source:MGI Symbol;Acc:MGI:1339958]	0.398683737	-1.326683338	1.91E-07	7.97E-06	yes	down
ENSMUSG00000038670	Mybpc2	myosin binding protein C, fast-type [Source:MGI Symbol;Acc:MGI:1336170]	3.854128255	1.946404581	1.98E-07	8.24E-06	yes	up
ENSMUSG00000024922	Ovol1	ovo like zinc finger 1 [Source:MGI Symbol;Acc:MGI:1330290]	0.376369346	-1.409778964	2.03E-07	8.42E-06	yes	down
ENSMUSG00000064181	Rab3ip	RAB3A interacting protein [Source:MGI Symbol;Acc:MGI:105933]	0.39837942	-1.327784972	2.12E-07	8.76E-06	yes	down
ENSMUSG00000051041	Olfml1	olfactomedin-like 1 [Source:MGI Symbol;Acc:MGI:2679264]	2.356097194	1.236399055	2.14E-07	8.84E-06	yes	up
ENSMUSG00000060487	Samd5	sterile alpha motif domain containing 5 [Source:MGI Symbol;Acc:MGI:2444815]	0.362565228	-1.463687524	2.15E-07	8.85E-06	yes	down
ENSMUSG00000061723	Tnnt3	troponin T3, skeletal, fast [Source:MGI	2.521731522	1.334414686	2.20E-07	8.97E-06	yes	up
ENSMUSG00000109445	4930560O18Rik	RIKEN cDNA 4930560O18 gene [Source:MGI Symbol;Acc:MGI:1923088]	0.160628472	-2.638200452	2.20E-07	8.97E-06	yes	down
ENSMUSG00000052302	Tbc1d30	TBC1 domain family, member 30 [Source:MGI Symbol;Acc:MGI:1921944]	0.453266702	-1.141567915	2.22E-07	9.04E-06	yes	down

ENSMUSG00000015652	Steap1	six transmembrane epithelial antigen of the prostate 1 [Source:MGI Symbol;Acc:MGI:1917608]	0.298144798	-1.74591493	2.26E-07	9.18E-06	yes	down
ENSMUSG00000040287	Stac3	SH3 and cysteine rich domain 3 [Source:MGI Symbol;Acc:MGI:3606571]	3.283611126	1.715283281	2.40E-07	9.72E-06	yes	up
ENSMUSG00000024743	Syt7	synaptotagmin VII [Source:MGI Symbol;Acc:MGI:1859545]	0.234580154	-2.09184713	2.43E-07	9.83E-06	yes	down
ENSMUSG00000069722	Krtap3-3	keratin associated protein 3-3 [Source:MGI Symbol;Acc:MGI:1913630]	0.097566642	-3.357468222	2.49E-07	1.00E-05	yes	down
ENSMUSG00000019929	Dcn	decorin [Source:MGI Symbol;Acc:MGI:94872]	2.11940093	1.08365653	2.51E-07	1.01E-05	yes	up
ENSMUSG00000030399	Ckm	creatine kinase, muscle [Source:MGI Symbol;Acc:MGI:88413]	2.988393586	1.579370171	2.69E-07	1.08E-05	yes	up
ENSMUSG00000074971	Fibin	fin bud initiation factor homolog (zebrafish) [Source:MGI Symbol;Acc:MGI:1914856]	2.268714211	1.181874885	2.70E-07	1.08E-05	yes	up
ENSMUSG00000025927	Tfap2b	transcription factor AP-2 beta [Source:MGI Symbol;Acc:MGI:104672]	0.4894717	-1.030702644	2.80E-07	1.11E-05	yes	down
ENSMUSG00000108967	Gm45181	predicted gene 45181 [Source:MGI Symbol;Acc:MGI:5753757]	0.135939723	-2.878961002	2.91E-07	1.16E-05	yes	down
ENSMUSG00000078130	Gm11555	predicted gene 11555 [Source:MGI Symbol;Acc:MGI:3651823]	0.088849473	-3.492492972	2.97E-07	1.18E-05	yes	down
ENSMUSG00000072720	Myo18b	myosin XVIIIb [Source:MGI Symbol;Acc:MGI:1921626]	2.468296729	1.30351584	3.05E-07	1.20E-05	yes	up
ENSMUSG00000041012	Cmtm8	CKLF-like MARVEL transmembrane domain containing 8 [Source:MGI Symbol;Acc:MGI:2447167]	0.461044217	-1.117022975	3.08E-07	1.21E-05	yes	down
ENSMUSG00000078255	Krtap9-5	keratin associated protein 9-5 [Source:MGI Symbol;Acc:MGI:3650333]	0.099208376	-3.333394256	3.13E-07	1.23E-05	yes	down
ENSMUSG00000016349	Eef1a2	eukaryotic translation elongation factor 1 alpha 2 [Source:MGI Symbol;Acc:MGI:1096317]	2.625174253	1.392413189	3.19E-07	1.25E-05	yes	up
ENSMUSG00000060600	Eno3	enolase 3, beta muscle [Source:MGI Symbol;Acc:MGI:95395]	2.541844581	1.34587582	3.20E-07	1.25E-05	yes	up
ENSMUSG00000067614	Krt86	keratin 86 [Source:MGI Symbol;Acc:MGI:109362]	0.098466644	-3.344221105	3.23E-07	1.26E-05	yes	down
ENSMUSG00000017204	Gsdma	gasdermin A [Source:MGI Symbol;Acc:MGI:1889509]	0.47194333	-1.08331446	3.25E-07	1.26E-05	yes	down
ENSMUSG00000038170	Pde4dip	phosphodiesterase 4D interacting protein (myomegalin) [Source:MGI Symbol;Acc:MGI:1891434]	2.382864318	1.252696806	3.29E-07	1.28E-05	yes	up
ENSMUSG00000068245	Phf11d	PHD finger protein 11D [Source:MGI Symbol;Acc:MGI:1277133]	2.13021595	1.090999691	3.40E-07	1.31E-05	yes	up
ENSMUSG00000067613	Krt83	keratin 83 [Source:MGI Symbol;Acc:MGI:3690448]	0.099207204	-3.333411303	3.44E-07	1.33E-05	yes	down
ENSMUSG00000021094	Dhrs7	dehydrogenase/reductase (SDR family) member 7 [Source:MGI Symbol;Acc:MGI:1913625]	2.29671374	1.199571052	3.45E-07	1.33E-05	yes	up
ENSMUSG00000051802	Krtap19-5	keratin associated protein 19-5 [Source:MGI Symbol;Acc:MGI:1330295]	0.059342839	-4.07478224	3.58E-07	1.37E-05	yes	down
ENSMUSG00000046794	Ppp1r3b	protein phosphatase 1, regulatory subunit 3B [Source:MGI Symbol;Acc:MGI:2177268]	0.393857876	-1.344252969	3.72E-07	1.42E-05	yes	down
ENSMUSG00000021319	Sfrp4	secreted frizzled-related protein 4 [Source:MGI Symbol;Acc:MGI:892010]	3.615354653	1.854137177	4.09E-07	1.55E-05	yes	up
ENSMUSG00000019564	Arid3a	AT rich interactive domain 3A (BRIGHT-like) [Source:MGI Symbol;Acc:MGI:1328360]	0.47443547	-1.075716222	4.24E-07	1.60E-05	yes	down
ENSMUSG00000051076	Vtcn1	V-set domain containing T cell activation inhibitor 1 [Source:MGI Symbol;Acc:MGI:3039619]	0.253496493	-1.979962305	4.24E-07	1.60E-05	yes	down
ENSMUSG00000037855	Zfp365	zinc finger protein 365 [Source:MGI Symbol;Acc:MGI:2143676]	0.402544661	-1.31277924	4.46E-07	1.67E-05	yes	down
ENSMUSG00000044938	Klhl31	kelch-like 31 [Source:MGI Symbol;Acc:MGI:3045305]	3.179639464	1.668863189	4.47E-07	1.67E-05	yes	up
ENSMUSG00000095662	H2al1g	H2A histone family member L1G [Source:MGI Symbol;Acc:MGI:3710577]	0.007005546	-7.157286843	4.53E-07	1.69E-05	yes	down
ENSMUSG00000059632	Krtap8-1	keratin associated protein 8-1 [Source:MGI Symbol;Acc:MGI:1330293]	0.089828704	-3.476679673	4.56E-07	1.69E-05	yes	down
ENSMUSG00000026950	Neb	nebulin [Source:MGI Symbol;Acc:MGI:97292]	2.271733775	1.183793775	4.62E-07	1.71E-05	yes	up
ENSMUSG00000020061	Mybpc1	myosin binding protein C, slow-type [Source:MGI Symbol;Acc:MGI:1336213]	2.041911903	1.029920623	4.63E-07	1.71E-05	yes	up
ENSMUSG00000112478	Gm47761	predicted gene, 47761 [Source:MGI Symbol;Acc:MGI:6096912]	3.509987007	1.81146569	4.63E-07	1.71E-05	yes	up

ENSMUSG00000057174	Krtap19-9b	keratin associated protein 19-9B [Source:MGI Symbol;Acc:MGI:2181750]	0.093784627	-3.414504735	4.72E-07	1.74E-05	yes	down
ENSMUSG00000036446	Lum	lumican [Source:MGI Symbol;Acc:MGI:109347]	2.645921001	1.403769988	4.76E-07	1.75E-05	yes	up
ENSMUSG00000078657	Crnn	cornulin [Source:MGI Symbol;Acc:MGI:2685861]	0.105251751	-3.248083856	4.77E-07	1.75E-05	yes	down
ENSMUSG00000028005	Gucy1b1	guanylate cyclase 1, soluble, beta 1 [Source:MGI Symbol;Acc:MGI:1860604]	2.254902064	1.173064775	4.83E-07	1.76E-05	yes	up
ENSMUSG00000021831	Ero1a	endoplasmic reticulum oxidoreductase 1 alpha [Source:MGI Symbol;Acc:MGI:1354385]	0.446658548	-1.162755724	4.85E-07	1.76E-05	yes	down
ENSMUSG00000043252	Tmem64	transmembrane protein 64 [Source:MGI	0.49940206	-1.001726322	4.96E-07	1.80E-05	yes	down
ENSMUSG00000055639	Dach1	dachshund family transcription factor 1 [Source:MGI Symbol;Acc:MGI:1277991]	0.430041282	-1.217452936	4.97E-07	1.80E-05	yes	down
ENSMUSG00000041886	Macc1	metastasis associated in colon cancer 1 [Source:MGI Symbol;Acc:MGI:2685113]	0.434301143	-1.203232346	5.07E-07	1.83E-05	yes	down
ENSMUSG00000044626	Liph	lipase, member H [Source:MGI Symbol;Acc:MGI:2388029]	0.436906786	-1.19460258	5.37E-07	1.94E-05	yes	down
ENSMUSG00000074647	Fam83c	family with sequence similarity 83, member C [Source:MGI Symbol;Acc:MGI:1918655]	0.363528615	-1.459859165	5.50E-07	1.98E-05	yes	down
ENSMUSG00000042436	Mfap4	microfibrillar-associated protein 4 [Source:MGI Symbol;Acc:MGI:1342276]	2.972143324	1.571503688	5.56E-07	2.00E-05	yes	up
ENSMUSG00000018983	E2f2	E2F transcription factor 2 [Source:MGI	0.394234157	-1.342875317	5.69E-07	2.03E-05	yes	down
ENSMUSG00000026576	Atp1b1	ATPase, Na+/K+ transporting, beta 1 polypeptide [Source:MGI Symbol;Acc:MGI:88108]	0.470693932	-1.087138842	5.96E-07	2.13E-05	yes	down
ENSMUSG00000030909	Anks4b	ankyrin repeat and sterile alpha motif domain containing 4B [Source:MGI Symbol;Acc:MGI:1919324]	0.010805955	-6.532029623	6.05E-07	2.15E-05	yes	down
ENSMUSG00000024136	Dnase1l2	deoxyribonuclease 1-like 2 [Source:MGI Symbol;Acc:MGI:1913955]	0.417581029	-1.259871926	6.05E-07	2.15E-05	yes	down
ENSMUSG00000033730	Egr3	early growth response 3 [Source:MGI Symbol;Acc:MGI:1306780]	0.428829002	-1.221525617	6.09E-07	2.16E-05	yes	down
ENSMUSG00000107193	Gm43700	predicted gene 43700 [Source:MGI Symbol;Acc:MGI:5663837]	3.411000759	1.770195076	6.09E-07	2.16E-05	yes	up
ENSMUSG00000043485	Krt34	keratin 34 [Source:MGI Symbol;Acc:MGI:1309994]	0.092917003	-3.427913573	6.14E-07	2.17E-05	yes	down
ENSMUSG00000069721	Krtap3-2	keratin associated protein 3-2 [Source:MGI Symbol;Acc:MGI:1913958]	0.102142244	-3.291348442	6.21E-07	2.19E-05	yes	down
ENSMUSG00000025557	Slc15a1	solute carrier family 15 (oligopeptide transporter), member 1 [Source:MGI Symbol;Acc:MGI:1861376]	0.489252171	-1.031349842	6.37E-07	2.24E-05	yes	down
ENSMUSG00000037434	Slc30a1	solute carrier family 30 (zinc transporter), member 1 [Source:MGI Symbol;Acc:MGI:1345281]	0.41920287	-1.2542795	6.46E-07	2.26E-05	yes	down
ENSMUSG00000090225	Gm11559	predicted gene 11559 [Source:MGI Symbol;Acc:MGI:3652067]	0.087130272	-3.520682139	6.67E-07	2.33E-05	yes	down
ENSMUSG00000030205	Gprc5d	G protein-coupled receptor, family C, group 5, member D [Source:MGI Symbol;Acc:MGI:1935037]	0.090266396	-3.469667176	6.91E-07	2.41E-05	yes	down
ENSMUSG00000039357	Fut11	fucosyltransferase 11 [Source:MGI Symbol;Acc:MGI:1920318]	6.592882567	2.720909384	7.31E-07	2.53E-05	yes	up
ENSMUSG00000026640	Plxna2	plexin A2 [Source:MGI Symbol;Acc:MGI:107684]	0.480517485	-1.057339167	7.38E-07	2.55E-05	yes	down
ENSMUSG00000020407	Upp1	uridine phosphorylase 1 [Source:MGI Symbol;Acc:MGI:1097668]	0.353465034	-1.500360588	7.39E-07	2.55E-05	yes	down
ENSMUSG00000033502	Cdc14a	CDC14 cell division cycle 14A [Source:MGI Symbol;Acc:MGI:2442676]	0.477834893	-1.065415887	7.40E-07	2.55E-05	yes	down
ENSMUSG00000052180	Serpinb6c	serine (or cysteine) peptidase inhibitor, clade B, member 6c [Source:MGI	2.420671835	1.27540751	7.44E-07	2.56E-05	yes	up
ENSMUSG00000097767	Miat	myocardial infarction associated transcript (non-protein coding) [Source:MGI Symbol;Acc:MGI:2444886]	4.34433241	2.119134496	7.56E-07	2.59E-05	yes	up
ENSMUSG00000041120	Nbl1	NBL1, DAN family BMP antagonist [Source:MGI Symbol;Acc:MGI:104591]	2.508555527	1.326856873	7.57E-07	2.59E-05	yes	up
ENSMUSG00000039904	Gpr37	G protein-coupled receptor 37 [Source:MGI Symbol;Acc:MGI:1313297]	0.285951818	-1.806156016	7.59E-07	2.59E-05	yes	down
ENSMUSG00000039364	Sectm1b	secreted and transmembrane 1B [Source:MGI Symbol;Acc:MGI:1929083]	2.834729088	1.503210865	8.08E-07	2.74E-05	yes	up

ENSMUSG00000044086	Lmod3	leiomodin 3 (fetal) [Source:MGI Symbol;Acc:MGI:2444169]	2.515968409	1.331113807	8.09E-07	2.74E-05	yes	up
ENSMUSG00000061816	Myl1	myosin, light polypeptide 1 [Source:MGI	2.332279911	1.221740946	8.11E-07	2.74E-05	yes	up
ENSMUSG00000038463	Olfml2b	olfactomedin-like 2B [Source:MGI Symbol;Acc:MGI:2443310]	2.734645146	1.451353638	8.53E-07	2.87E-05	yes	up
ENSMUSG00000000567	Sox9	SRY (sex determining region Y)-box 9 [Source:MGI	0.47143864	-1.084858085	8.61E-07	2.89E-05	yes	down
ENSMUSG00000035357	Pdzrn3	PDZ domain containing RING finger 3 [Source:MGI Symbol;Acc:MGI:1933157]	0.440441605	-1.18297734	8.83E-07	2.95E-05	yes	down
ENSMUSG00000074928	Krtap14	keratin associated protein 14 [Source:MGI Symbol;Acc:MGI:1346079]	0.0678625	-3.881241605	8.84E-07	2.95E-05	yes	down
ENSMUSG00000030283	St8sia1	ST8 alpha-N-acetyl-neuraminide alpha-2,8-sialyltransferase 1 [Source:MGI	0.239750268	-2.060395667	8.88E-07	2.95E-05	yes	down
ENSMUSG00000085683	Tmem238l	transmembrane protein 238 like [Source:MGI Symbol;Acc:MGI:1918826]	0.206057055	-2.278884237	8.89E-07	2.95E-05	yes	down
ENSMUSG00000078815	Cacng6	calcium channel, voltage-dependent, gamma subunit 6 [Source:MGI Symbol;Acc:MGI:1859168]	3.523444485	1.816986483	8.89E-07	2.95E-05	yes	up
ENSMUSG00000055937	Krt28	keratin 28 [Source:MGI Symbol;Acc:MGI:1918093]	0.088909789	-3.491513921	9.03E-07	2.99E-05	yes	down
ENSMUSG00000022537	Tmem44	transmembrane protein 44 [Source:MGI	2.007799225	1.00561501	9.35E-07	3.08E-05	yes	up
ENSMUSG00000015396	Cd83	CD83 antigen [Source:MGI Symbol;Acc:MGI:1328316]	2.919393069	1.545668469	9.60E-07	3.16E-05	yes	up
ENSMUSG00000042532	Golga7b	golgi autoantigen, golgin subfamily a, 7B [Source:MGI Symbol;Acc:MGI:1918396]	0.260151048	-1.942578573	9.69E-07	3.19E-05	yes	down
ENSMUSG00000079017	Ifi27l2a	interferon, alpha-inducible protein 27 like 2A [Source:MGI Symbol;Acc:MGI:1924183]	2.557288045	1.35461467	9.90E-07	3.25E-05	yes	up
ENSMUSG00000045566	Sprr4	small proline-rich protein 4 [Source:MGI Symbol;Acc:MGI:2654508]	0.36204917	-1.465742453	1.03E-06	3.36E-05	yes	down
ENSMUSG00000002500	Rpl3l	ribosomal protein L3-like [Source:MGI	2.460182441	1.298765306	1.08E-06	3.51E-05	yes	up
ENSMUSG00000022629	Kif21a	kinesin family member 21A [Source:MGI Symbol;Acc:MGI:109188]	0.460164114	-1.119779617	1.11E-06	3.62E-05	yes	down
ENSMUSG00000118106		TEC	0.336510354	-1.5712772	1.14E-06	3.69E-05	yes	down
ENSMUSG00000028392	Bspry	B-box and SPRY domain containing [Source:MGI Symbol;Acc:MGI:2177191]	0.391896152	-1.351456687	1.19E-06	3.84E-05	yes	down
ENSMUSG00000028047	Thbs3	thrombospondin 3 [Source:MGI Symbol;Acc:MGI:98739]	3.156930619	1.658522554	1.23E-06	3.98E-05	yes	up
ENSMUSG00000056605	Krt72	keratin 72 [Source:MGI Symbol;Acc:MGI:2146034]	0.086507608	-3.531029172	1.25E-06	4.01E-05	yes	down
ENSMUSG00000056706	Krtap7-1	keratin associated protein 7-1 [Source:MGI Symbol;Acc:MGI:1918613]	0.095971134	-3.381255653	1.27E-06	4.07E-05	yes	down
ENSMUSG00000031740	Mmp2	matrix metallopeptidase 2 [Source:MGI	2.802480523	1.486704347	1.31E-06	4.21E-05	yes	up
ENSMUSG00000043461	Sptssb	serine palmitoyltransferase, small subunit B [Source:MGI Symbol;Acc:MGI:1913433]	0.141971395	-2.816327816	1.32E-06	4.22E-05	yes	down
ENSMUSG00000068075	Gm10229	predicted gene 10229 [Source:MGI Symbol;Acc:MGI:3711943]	0.111231253	-3.168365897	1.33E-06	4.24E-05	yes	down
ENSMUSG00000022525	Plaat1	phospholipase A and acyltransferase 1 [Source:MGI Symbol;Acc:MGI:1351473]	5.720182283	2.516061122	1.33E-06	4.24E-05	yes	up
ENSMUSG00000051906	Cd209f	CD209f antigen [Source:MGI Symbol;Acc:MGI:1916392]	2.37518228	1.248038235	1.35E-06	4.29E-05	yes	up
ENSMUSG00000040666	Sh3bgr	SH3-binding domain glutamic acid-rich protein [Source:MGI Symbol;Acc:MGI:1354740]	2.104080028	1.073189578	1.38E-06	4.38E-05	yes	up
ENSMUSG00000078349	AW011738	expressed sequence AW011738 [Source:MGI Symbol;Acc:MGI:2140540]	0.467718123	-1.096288765	1.41E-06	4.46E-05	yes	down
ENSMUSG00000030108	Slc6a13	solute carrier family 6 (neurotransmitter transporter, GABA), member 13 [Source:MGI	0.24076697	-2.054290607	1.42E-06	4.49E-05	yes	down
ENSMUSG00000032181	Scg3	secretogranin III [Source:MGI Symbol;Acc:MGI:103032]	3.898727599	1.963003358	1.42E-06	4.49E-05	yes	up
ENSMUSG00000044641	Pard6b	par-6 family cell polarity regulator beta [Source:MGI Symbol;Acc:MGI:2135605]	0.320142571	-1.643213561	1.45E-06	4.57E-05	yes	down
ENSMUSG00000040950	Mgl2	macrophage galactose N-acetyl-galactosamine specific lectin 2 [Source:MGI Symbol;Acc:MGI:2385729]	3.414533816	1.771688622	1.46E-06	4.57E-05	yes	up

ENSMUSG00000048706	Lurap1l	leucine rich adaptor protein 1-like [Source:MGI Symbol;Acc:MGI:106510]	0.486635887	-1.03908538	1.46E-06	4.57E-05	yes	down
ENSMUSG00000096534	Krtap16-3	keratin associated protein 16-3 [Source:MGI Symbol;Acc:MGI:1918619]	0.072273891	-3.790381617	1.46E-06	4.57E-05	yes	down
ENSMUSG00000079588	Tmem182	transmembrane protein 182 [Source:MGI Symbol;Acc:MGI:1923725]	2.128120441	1.089579803	1.51E-06	4.70E-05	yes	up
ENSMUSG00000076583	Igkv8-24	immunoglobulin kappa chain variable 8-24 [Source:MGI Symbol;Acc:MGI:4947958]	6.449793605	2.689252995	1.54E-06	4.77E-05	yes	up
ENSMUSG00000005628	Tmod4	tropomodulin 4 [Source:MGI Symbol;Acc:MGI:1355285]	2.440066184	1.28692028	1.56E-06	4.84E-05	yes	up
ENSMUSG00000037977	6430571L13Rik	RIKEN cDNA 6430571L13 gene [Source:MGI Symbol;Acc:MGI:2445137]	0.311528253	-1.682565085	1.64E-06	5.02E-05	yes	down
ENSMUSG00000029359	Tesc	tescalcin [Source:MGI Symbol;Acc:MGI:1930803]	0.324437237	-1.623988681	1.65E-06	5.06E-05	yes	down
ENSMUSG00000075570	Krt26	keratin 26 [Source:MGI Symbol;Acc:MGI:2444913]	0.101638576	-3.29848003	1.67E-06	5.12E-05	yes	down
ENSMUSG00000015619	Gata3	GATA binding protein 3 [Source:MGI Symbol;Acc:MGI:95663]	0.474648975	-1.075067127	1.74E-06	5.31E-05	yes	down
ENSMUSG00000061751	Kalrn	kalirin, RhoGEF kinase [Source:MGI Symbol;Acc:MGI:2685385]	0.426747751	-1.228544546	1.78E-06	5.42E-05	yes	down
ENSMUSG00000001027	Scn4a	sodium channel, voltage-gated, type IV, alpha [Source:MGI Symbol;Acc:MGI:98250]	3.731056988	1.899584396	1.90E-06	5.74E-05	yes	up
ENSMUSG00000027358	Bmp2	bone morphogenetic protein 2 [Source:MGI	0.412609957	-1.27714946	1.91E-06	5.76E-05	yes	down
ENSMUSG00000047361	Gm973	predicted gene 973 [Source:MGI Symbol;Acc:MGI:2685819]	2.485633934	1.313613842	1.92E-06	5.76E-05	yes	up
ENSMUSG00000041737	Tmem45b	transmembrane protein 45b [Source:MGI Symbol;Acc:MGI:2384574]	3.375213005	1.754978551	2.00E-06	6.00E-05	yes	up
ENSMUSG00000027908	Tchhl1	trichohyalin-like 1 [Source:MGI Symbol;Acc:MGI:1918575]	0.116695199	-3.099182893	2.02E-06	6.04E-05	yes	down
ENSMUSG00000070469	Adamtsl3	ADAMTS-like 3 [Source:MGI Symbol;Acc:MGI:3028499]	3.123255146	1.643050431	2.03E-06	6.04E-05	yes	up
ENSMUSG00000041984	Rptn	repetin [Source:MGI Symbol;Acc:MGI:1099055]	2.693396603	1.429426682	2.03E-06	6.04E-05	yes	up
ENSMUSG00000034990	Otoa	otoancorin [Source:MGI Symbol;Acc:MGI:2149209]	0.137878918	-2.858526211	2.11E-06	6.24E-05	yes	down
ENSMUSG00000047953	Gp5	glycoprotein 5 (platelet) [Source:MGI Symbol;Acc:MGI:1096363]	0.159874695	-2.644986486	2.17E-06	6.39E-05	yes	down
ENSMUSG00000029718	Pcolce	procollagen C-endopeptidase enhancer protein [Source:MGI Symbol;Acc:MGI:105099]	2.165346703	1.11459804	2.20E-06	6.46E-05	yes	up
ENSMUSG00000047564	Krtap3-1	keratin associated protein 3-1 [Source:MGI Symbol;Acc:MGI:1916723]	0.097837373	-3.353470529	2.20E-06	6.46E-05	yes	down
ENSMUSG00000050765	Gm5084	predicted gene 5084 [Source:MGI Symbol;Acc:MGI:3647835]	0.480492727	-1.057413502	2.25E-06	6.60E-05	yes	down
ENSMUSG00000051431	Gpr87	G protein-coupled receptor 87 [Source:MGI Symbol;Acc:MGI:1934133]	0.43823776	-1.190214297	2.26E-06	6.60E-05	yes	down
ENSMUSG00000036437	Npy1r	neuropeptide Y receptor Y1 [Source:MGI Symbol;Acc:MGI:104963]	2.144208435	1.100445155	2.27E-06	6.64E-05	yes	up
ENSMUSG00000062400	Krtap6-5	keratin associated protein 6-5 [Source:MGI Symbol;Acc:MGI:1915734]	0.126585891	-2.981811481	2.33E-06	6.80E-05	yes	down
ENSMUSG00000049809	Krtap9-3	keratin associated protein 9-3 [Source:MGI Symbol;Acc:MGI:1922836]	0.105387718	-3.246221354	2.36E-06	6.87E-05	yes	down
ENSMUSG00000071540	3425401B19Rik	RIKEN cDNA 3425401B19 gene [Source:MGI Symbol;Acc:MGI:3588196]	2.47027059	1.304669081	2.36E-06	6.87E-05	yes	up
ENSMUSG00000074001	Klhl40	kelch-like 40 [Source:MGI Symbol;Acc:MGI:1919580]	2.912449627	1.542233098	2.38E-06	6.88E-05	yes	up
ENSMUSG00000004633	Chn2	chimerin 2 [Source:MGI Symbol;Acc:MGI:1917243]	0.456520124	-1.131249637	2.51E-06	7.22E-05	yes	down
ENSMUSG00000023232	Serinc2	serine incorporator 2 [Source:MGI Symbol;Acc:MGI:1919132]	0.490331106	-1.028171806	2.56E-06	7.35E-05	yes	down
ENSMUSG00000074206	Adh6b	alcohol dehydrogenase 6B (class V) [Source:MGI Symbol;Acc:MGI:2446626]	0.065741623	-3.927049119	2.64E-06	7.56E-05	yes	down
ENSMUSG00000009614	Sardh	sarcosine dehydrogenase [Source:MGI	0.454215555	-1.138550981	2.67E-06	7.64E-05	yes	down
ENSMUSG00000046879	Irgm1	immunity-related GTPase family M member 1 [Source:MGI Symbol;Acc:MGI:107567]	2.202117565	1.138891492	2.72E-06	7.75E-05	yes	up
ENSMUSG00000033327	Tnxb	tenascin XB [Source:MGI Symbol;Acc:MGI:1932137]	3.207487825	1.681443789	2.74E-06	7.80E-05	yes	up
ENSMUSG00000086514	Gm11747	predicted gene 11747 [Source:MGI Symbol;Acc:MGI:3702093]	2.459443061	1.298331655	2.76E-06	7.85E-05	yes	up

ENSMUSG00000048583	Igf2	insulin-like growth factor 2 [Source:MGI	2.24816471	1.168747737	2.84E-06	8.03E-05	yes	up
ENSMUSG00000018634	Crhr1	corticotropin releasing hormone receptor 1 [Source:MGI Symbol;Acc:MGI:88498]	0.112211347	-3.155709528	2.92E-06	8.20E-05	yes	down
ENSMUSG00000025991	Cps1	carbamoyl-phosphate synthetase 1 [Source:MGI Symbol;Acc:MGI:891996]	0.237766366	-2.072383448	3.02E-06	8.48E-05	yes	down
ENSMUSG00000030249	Abcc9	ATP-binding cassette, sub-family C (CFTR/MRP), member 9 [Source:MGI	2.238576401	1.162581557	3.05E-06	8.54E-05	yes	up
ENSMUSG00000097451	Rian	RNA imprinted and accumulated in nucleus [Source:MGI Symbol;Acc:MGI:1922995]	2.444325127	1.289436195	3.06E-06	8.54E-05	yes	up
ENSMUSG00000038415	Foxq1	forkhead box Q1 [Source:MGI Symbol;Acc:MGI:1298228]	0.396862471	-1.333288954	3.10E-06	8.63E-05	yes	down
ENSMUSG00000035095	Fam167a	family with sequence similarity 167, member A [Source:MGI Symbol;Acc:MGI:3606565]	0.469375962	-1.091184137	3.10E-06	8.63E-05	yes	down
ENSMUSG00000059325	Hopx	HOP homeobox [Source:MGI Symbol;Acc:MGI:1916782]	0.481742212	-1.053666751	3.16E-06	8.77E-05	yes	down
ENSMUSG00000030905	Crym	crystallin, mu [Source:MGI Symbol;Acc:MGI:102675]	0.088238688	-3.502444852	3.17E-06	8.78E-05	yes	down
ENSMUSG00000030730	Atp2a1	ATPase, Ca++ transporting, cardiac muscle, fast twitch 1 [Source:MGI Symbol;Acc:MGI:105058]	2.88809157	1.530116485	3.20E-06	8.85E-05	yes	up
ENSMUSG00000020475	Pgam2	phosphoglycerate mutase 2 [Source:MGI Symbol;Acc:MGI:1933118]	2.744972944	1.456791929	3.28E-06	9.02E-05	yes	up
ENSMUSG00000079363	Gbp4	guanylate binding protein 4 [Source:MGI	2.432301247	1.282321922	3.32E-06	9.11E-05	yes	up
ENSMUSG00000047253	Krtap1-5	keratin associated protein 1-5 [Source:MGI Symbol;Acc:MGI:1916914]	0.110789678	-3.174104616	3.40E-06	9.30E-05	yes	down
ENSMUSG00000097604	Gm17322	predicted gene, 17322 [Source:MGI Symbol;Acc:MGI:4936956]	0.169474521	-2.560859706	3.42E-06	9.36E-05	yes	down
ENSMUSG00000029154	Cwh43	cell wall biogenesis 43 C-terminal homolog [Source:MGI Symbol;Acc:MGI:2444131]	0.446868734	-1.162076987	3.50E-06	9.56E-05	yes	down
ENSMUSG00000036687	Tmem184a	transmembrane protein 184a [Source:MGI Symbol;Acc:MGI:2385897]	0.484632117	-1.045038077	3.52E-06	9.59E-05	yes	down
ENSMUSG00000024064	Galnt14	polypeptide N-acetylgalactosaminyltransferase 14 [Source:MGI Symbol;Acc:MGI:1918935]	0.313177984	-1.674945297	3.61E-06	9.82E-05	yes	down
ENSMUSG00000040693	Slco4c1	solute carrier organic anion transporter family, member 4C1 [Source:MGI	0.284597913	-1.813003013	3.67E-06	9.95E-05	yes	down
ENSMUSG00000020159	Gabrp	gamma-aminobutyric acid (GABA) A receptor, pi [Source:MGI Symbol;Acc:MGI:2387597]	0.090610439	-3.464178928	3.70E-06	1.00E-04	yes	down
ENSMUSG00000021303	Gng4	guanine nucleotide binding protein (G protein), gamma 4 [Source:MGI Symbol;Acc:MGI:102703]	0.342110886	-1.547464083	3.70E-06	1.00E-04	yes	down
ENSMUSG00000027611	Procr	protein C receptor, endothelial [Source:MGI Symbol;Acc:MGI:104596]	0.443722482	-1.172270443	3.70E-06	1.00E-04	yes	down
ENSMUSG00000086552	Dlx4os	distal-less homeobox 4, opposite strand [Source:MGI Symbol;Acc:MGI:2444057]	0.1708553	-2.549153095	3.79E-06	0.00010204	yes	down
ENSMUSG00000015702	Anxa9	annexin A9 [Source:MGI Symbol;Acc:MGI:1923711]	0.407177226	-1.296271223	3.88E-06	0.00010423	yes	down
ENSMUSG00000017344	Vtn	vitronectin [Source:MGI Symbol;Acc:MGI:98940]	2.411371338	1.269853835	3.90E-06	0.00010465	yes	up
ENSMUSG00000069372	Ctxn3	cortexin 3 [Source:MGI Symbol;Acc:MGI:3642816]	4.198530976	2.069884631	3.94E-06	0.00010563	yes	up
ENSMUSG00000071656	Lrrn4cl	LRRN4 C-terminal like [Source:MGI Symbol;Acc:MGI:1916102]	2.507481135	1.326238847	4.14E-06	0.00011038	yes	up
ENSMUSG00000048234	Rnf149	ring finger protein 149 [Source:MGI Symbol;Acc:MGI:2677438]	0.448423143	-1.157067357	4.18E-06	0.00011112	yes	down
ENSMUSG00000079164	Tlr5	toll-like receptor 5 [Source:MGI Symbol;Acc:MGI:1858171]	2.34399859	1.228971702	4.25E-06	0.00011279	yes	up
ENSMUSG00000024049	Myom1	myomesin 1 [Source:MGI Symbol;Acc:MGI:1341430]	2.466187165	1.302282294	4.30E-06	0.00011373	yes	up
ENSMUSG00000028683	Eif2b3	eukaryotic translation initiation factor 2B, subunit 3 [Source:MGI Symbol;Acc:MGI:1313286]	0.48100383	-1.055879714	4.33E-06	0.00011421	yes	down
ENSMUSG00000029819	Npy	neuropeptide Y [Source:MGI Symbol;Acc:MGI:97374]	0.271211439	-1.882510068	4.36E-06	0.00011471	yes	down
ENSMUSG00000046157	Tmem229b	transmembrane protein 229B [Source:MGI Symbol;Acc:MGI:2444389]	0.343124362	-1.543196534	4.38E-06	0.00011515	yes	down
ENSMUSG00000032648	Pygm	muscle glycogen phosphorylase [Source:MGI	2.922787537	1.547344961	4.39E-06	0.00011522	yes	up

ENSMUSG00000030592	Ryr1	ryanodine receptor 1, skeletal muscle [Source:MGI	2.861648433	1.516846441	4.40E-06	0.00011536	yes	up
ENSMUSG00000034855	Cxcl10	chemokine (C-X-C motif) ligand 10 [Source:MGI Symbol;Acc:MGI:1352450]	2.480355031	1.310546639	4.57E-06	0.00011964	yes	up
ENSMUSG00000047730	Fcgbp	Fc fragment of IgG binding protein [Source:MGI Symbol;Acc:MGI:2444336]	3.760723634	1.91101029	4.69E-06	0.00012223	yes	up
ENSMUSG00000026676	Ccdc3	coiled-coil domain containing 3 [Source:MGI Symbol;Acc:MGI:1921436]	2.035780434	1.02558197	4.83E-06	0.00012584	yes	up
ENSMUSG00000021379	Id4	inhibitor of DNA binding 4 [Source:MGI	0.451491908	-1.147227965	4.89E-06	0.00012704	yes	down
ENSMUSG00000000957	Mmp14	matrix metallopeptidase 14 (membrane-inserted) [Source:MGI Symbol;Acc:MGI:101900]	2.522643	1.334936053	4.99E-06	0.00012922	yes	up
ENSMUSG00000028270	Gbp2	guanylate binding protein 2 [Source:MGI Symbol;Acc:MGI:102772]	3.037925968	1.603086713	5.06E-06	0.00013073	yes	up
ENSMUSG00000033470	Cysltr2	cysteinyl leukotriene receptor 2 [Source:MGI Symbol;Acc:MGI:1917336]	2.613613688	1.386045915	5.23E-06	0.00013435	yes	up
ENSMUSG00000029862	Clcn1	chloride channel, voltage-sensitive 1 [Source:MGI	2.773807616	1.47186773	5.25E-06	0.00013462	yes	up
ENSMUSG00000000385	Tmprss2	transmembrane protease, serine 2 [Source:MGI Symbol;Acc:MGI:1354381]	0.358874225	-1.478449785	5.31E-06	0.00013579	yes	down
ENSMUSG00000059493	Nhs	NHS actin remodeling regulator [Source:MGI Symbol;Acc:MGI:2684894]	0.497926186	-1.005996207	5.39E-06	0.00013735	yes	down
ENSMUSG00000089865	Gm44503	predicted readthrough transcript (NMD candidate), 44503 [Source:MGI	3.869073751	1.951988229	5.39E-06	0.00013735	yes	up
ENSMUSG00000041323	Ak7	adenylate kinase 7 [Source:MGI Symbol;Acc:MGI:1926051]	0.227719549	-2.13466995	5.47E-06	0.0001392	yes	down
ENSMUSG00000044645	Gm7334	predicted gene 7334 [Source:MGI Symbol;Acc:MGI:3647393]	0.44604713	-1.16473194	5.48E-06	0.00013933	yes	down
ENSMUSG00000105096	Gbp10	guanylate-binding protein 10 [Source:MGI Symbol;Acc:MGI:4359647]	8.346436871	3.061160436	5.73E-06	0.00014535	yes	up
ENSMUSG00000020019	Ntn4	netrin 4 [Source:MGI Symbol;Acc:MGI:1888978]	0.454202871	-1.13859127	5.77E-06	0.00014584	yes	down
ENSMUSG00000044550	Tceal3	transcription elongation factor A (SII)-like 3 [Source:MGI Symbol;Acc:MGI:1913354]	0.399368416	-1.324207852	5.77E-06	0.00014584	yes	down
ENSMUSG00000053846	Lipg	lipase, endothelial [Source:MGI Symbol;Acc:MGI:1341803]	0.423903408	-1.23819253	5.80E-06	0.0001464	yes	down
ENSMUSG00000021768	Dusp13	dual specificity phosphatase 13 [Source:MGI Symbol;Acc:MGI:1351599]	4.309975191	2.107679565	5.88E-06	0.0001479	yes	up
ENSMUSG00000091345	Col6a5	collagen, type VI, alpha 5 [Source:MGI	3.035895566	1.602122163	5.88E-06	0.0001479	yes	up
ENSMUSG00000026090	Cracdl	capping protein inhibiting regulator of actin like [Source:MGI Symbol;Acc:MGI:1919347]	0.396059931	-1.336209341	5.89E-06	0.0001479	yes	down
ENSMUSG00000093955	Ighv1-34	immunoglobulin heavy variable 1-34 [Source:MGI Symbol;Acc:MGI:4439659]	2.53682729	1.343025303	5.91E-06	0.00014825	yes	up
ENSMUSG00000104713	Gbp6	guanylate binding protein 6 [Source:MGI Symbol;Acc:MGI:2140937]	3.278709368	1.713128024	5.92E-06	0.00014825	yes	up
ENSMUSG00000028268	Gbp3	guanylate binding protein 3 [Source:MGI Symbol;Acc:MGI:1926263]	2.434417123	1.283576386	5.96E-06	0.00014901	yes	up
ENSMUSG00000031303	Map3k15	mitogen-activated protein kinase kinase kinase 15 [Source:MGI Symbol;Acc:MGI:2448588]	0.395795663	-1.33717229	6.02E-06	0.00015032	yes	down
ENSMUSG00000041986	Elmod1	ELMO/CED-12 domain containing 1 [Source:MGI Symbol;Acc:MGI:3583900]	0.410101095	-1.285948498	6.04E-06	0.00015057	yes	down
ENSMUSG00000022797	Tfrc	transferrin receptor [Source:MGI Symbol;Acc:MGI:98822]	0.414254215	-1.271411719	6.08E-06	0.00015123	yes	down
ENSMUSG00000002769	Gnmt	glycine N-methyltransferase [Source:MGI Symbol;Acc:MGI:1202304]	0.185391668	-2.431351687	6.09E-06	0.00015139	yes	down
ENSMUSG00000021356	Irf4	interferon regulatory factor 4 [Source:MGI Symbol;Acc:MGI:1096873]	2.101616337	1.071499321	6.15E-06	0.00015259	yes	up
ENSMUSG00000027401	Tgm3	transglutaminase 3, E polypeptide [Source:MGI	0.252037292	-1.98829088	6.18E-06	0.00015312	yes	down
ENSMUSG00000027800	Tm4sf1	transmembrane 4 superfamily member 1 [Source:MGI Symbol;Acc:MGI:104678]	0.38898776	-1.362203334	6.25E-06	0.00015434	yes	down
ENSMUSG00000023043	Krt18	keratin 18 [Source:MGI Symbol;Acc:MGI:96692]	0.150016865	-2.736803398	6.40E-06	0.00015779	yes	down

ENSMUSG00000017652	Cd40	CD40 antigen [Source:MGI Symbol;Acc:MGI:88336]	2.352492744	1.234190273	6.48E-06	0.00015947	yes	up
ENSMUSG00000000093	Tbx2	T-box 2 [Source:MGI Symbol;Acc:MGI:98494]	2.038830657	1.027741952	6.52E-06	0.00016026	yes	up
ENSMUSG00000054905	Stfa3	stefin A3 [Source:MGI Symbol;Acc:MGI:106196]	2.33413753	1.222889569	6.81E-06	0.00016685	yes	up
ENSMUSG00000026109	Tmeff2	transmembrane protein with EGF-like and two follistatin-like domains 2 [Source:MGI Symbol;Acc:MGI:1861735]	2.323725051	1.216439375	6.84E-06	0.00016723	yes	up
ENSMUSG00000030693	Klk10	kallikrein related-peptidase 10 [Source:MGI Symbol;Acc:MGI:1916790]	0.385650793	-1.374633016	6.99E-06	0.00016984	yes	down
ENSMUSG00000051747	Ttn	titin [Source:MGI Symbol;Acc:MGI:98864]	2.768156439	1.468925477	7.03E-06	0.0001707	yes	up
ENSMUSG00000079018	Ly6c1	lymphocyte antigen 6 complex, locus C1 [Source:MGI Symbol;Acc:MGI:96882]	2.04543629	1.032408602	7.36E-06	0.00017788	yes	up
ENSMUSG00000038155	Gstp2	glutathione S-transferase, pi 2 [Source:MGI	2.286273513	1.192998007	7.51E-06	0.00018102	yes	up
ENSMUSG00000015354	Pcolce2	procollagen C-endopeptidase enhancer 2 [Source:MGI Symbol;Acc:MGI:1923727]	2.669188305	1.416401088	7.51E-06	0.00018102	yes	up
ENSMUSG00000024896	Minpp1	multiple inositol polyphosphate histidine phosphatase 1 [Source:MGI Symbol;Acc:MGI:1336159]	0.491871032	-1.023648003	7.52E-06	0.00018102	yes	down
ENSMUSG00000032334	Loxl1	lysyl oxidase-like 1 [Source:MGI Symbol;Acc:MGI:106096]	2.241747728	1.164623936	7.65E-06	0.00018343	yes	up
ENSMUSG00000038763	Alpk3	alpha-kinase 3 [Source:MGI Symbol;Acc:MGI:2151224]	2.518287921	1.332443239	7.66E-06	0.00018343	yes	up
ENSMUSG00000060469	Krtap19-3	keratin associated protein 19-3 [Source:MGI Symbol;Acc:MGI:1925168]	0.058757447	-4.089084474	7.69E-06	0.000184	yes	down
ENSMUSG00000035112	Wnk4	WNK lysine deficient protein kinase 4 [Source:MGI Symbol;Acc:MGI:1917097]	0.458759773	-1.124189203	7.75E-06	0.00018479	yes	down
ENSMUSG00000095992	Krtap22-2	keratin associated protein 22-2 [Source:MGI Symbol;Acc:MGI:1915990]	0.120741441	-3.050007172	7.86E-06	0.00018723	yes	down
ENSMUSG00000039395	Mreg	melanoregulin [Source:MGI Symbol;Acc:MGI:2151839]	0.441563739	-1.179306392	8.05E-06	0.00019093	yes	down
ENSMUSG00000032278	Paqr5	progestin and adipoQ receptor family member V [Source:MGI Symbol;Acc:MGI:1921340]	0.32061646	-1.641079603	8.08E-06	0.00019139	yes	down
ENSMUSG00000095930	Nim1k	NIM1 serine/threonine protein kinase [Source:MGI Symbol;Acc:MGI:2442399]	0.365802533	-1.450863031	8.12E-06	0.00019194	yes	down
ENSMUSG00000055629	B4galnt4	beta-1,4-N-acetyl-galactosaminyl transferase 4 [Source:MGI Symbol;Acc:MGI:2652891]	0.423936215	-1.23808088	8.17E-06	0.00019297	yes	down
ENSMUSG00000019368	Sec14l4	SEC14-like lipid binding 4 [Source:MGI	2.60762388	1.382735792	8.28E-06	0.00019519	yes	up
ENSMUSG00000050108	Bpifc	BPI fold containing family C [Source:MGI Symbol;Acc:MGI:3026884]	0.450155235	-1.151505497	8.54E-06	0.00020107	yes	down
ENSMUSG00000028358	Zfp618	zinc finger protein 618 [Source:MGI Symbol;Acc:MGI:1919950]	0.490389043	-1.028001351	8.61E-06	0.00020198	yes	down
ENSMUSG00000046826	Fam187b	family with sequence similarity 187, member B [Source:MGI Symbol;Acc:MGI:1923665]	0.338495465	-1.562791591	8.63E-06	0.00020223	yes	down
ENSMUSG00000113581	Dio3os	deiodinase, iodothyronine type III, opposite strand [Source:MGI Symbol;Acc:MGI:2664395]	0.121792593	-3.037501694	8.88E-06	0.00020742	yes	down
ENSMUSG00000074457	S100a16	S100 calcium binding protein A16 [Source:MGI Symbol;Acc:MGI:1915110]	0.499483358	-1.001491485	8.90E-06	0.00020763	yes	down
ENSMUSG00000078921	Tgtp2	T cell specific GTPase 2 [Source:MGI Symbol;Acc:MGI:3710083]	2.93834076	1.555001715	9.05E-06	0.0002108	yes	up
ENSMUSG00000042251	Pm20d1	peptidase M20 domain containing 1 [Source:MGI Symbol;Acc:MGI:2442939]	0.316865347	-1.658058202	9.08E-06	0.00021121	yes	down
ENSMUSG00000022440	C1qtnf6	C1q and tumor necrosis factor related protein 6 [Source:MGI Symbol;Acc:MGI:1919959]	2.310226951	1.208034585	9.23E-06	0.00021336	yes	up
ENSMUSG00000034457	Eda2r	ectodysplasin A2 receptor [Source:MGI Symbol;Acc:MGI:2442860]	2.101747858	1.071589603	9.54E-06	0.00021969	yes	up
ENSMUSG00000049551	Fzd9	frizzled class receptor 9 [Source:MGI Symbol;Acc:MGI:1313278]	5.676578139	2.50502153	9.54E-06	0.00021969	yes	up
ENSMUSG00000052949	Rnf157	ring finger protein 157 [Source:MGI Symbol;Acc:MGI:2442484]	0.485020524	-1.043882299	9.59E-06	0.0002205	yes	down
ENSMUSG00000098488	Pla2g4b	phospholipase A2, group IVB (cytosolic) [Source:MGI Symbol;Acc:MGI:2384819]	0.40945082	-1.288237916	9.75E-06	0.0002232	yes	down

ENSMUSG00000028528	Dnajc6	DnaJ heat shock protein family (Hsp40) member C6 [Source:MGI Symbol;Acc:MGI:1919935]	0.342109149	-1.547471407	9.79E-06	0.00022383	yes	down
ENSMUSG00000025997	Ikzf2	IKAROS family zinc finger 2 [Source:MGI Symbol;Acc:MGI:1342541]	2.070609973	1.050055828	1.01E-05	0.00023008	yes	up
ENSMUSG00000068074	Gm10228	predicted gene 10228 [Source:MGI Symbol;Acc:MGI:3704467]	0.127468477	-2.971787588	1.03E-05	0.00023361	yes	down
ENSMUSG00000030785	Cox6a2	cytochrome c oxidase subunit 6A2 [Source:MGI Symbol;Acc:MGI:104649]	2.089099278	1.062881054	1.03E-05	0.00023373	yes	up
ENSMUSG00000105557	Mir3966	microRNA 3966 [Source:MGI Symbol;Acc:MGI:4950405]	4.891713962	2.290340046	1.07E-05	0.00024286	yes	up
ENSMUSG00000020681	Ace	angiotensin I converting enzyme (peptidyl-dipeptidase A) 1 [Source:MGI	2.321165693	1.214849511	1.08E-05	0.00024434	yes	up
ENSMUSG00000055489	Ano5	anoctamin 5 [Source:MGI Symbol;Acc:MGI:3576659]	3.504081815	1.80903646	1.09E-05	0.00024589	yes	up
ENSMUSG00000033420	Antxr1	anthrax toxin receptor 1 [Source:MGI Symbol;Acc:MGI:1916788]	2.698034519	1.431908807	1.11E-05	0.00024861	yes	up
ENSMUSG00000022598	Psca	prostate stem cell antigen [Source:MGI	0.178662259	-2.484693189	1.13E-05	0.00025305	yes	down
ENSMUSG00000027134	Lpcat4	lysophosphatidylcholine acyltransferase 4 [Source:MGI Symbol;Acc:MGI:2138993]	0.466816831	-1.099071516	1.14E-05	0.00025401	yes	down
ENSMUSG00000023391	Dlx2	distal-less homeobox 2 [Source:MGI Symbol;Acc:MGI:94902]	0.358735938	-1.479005816	1.20E-05	0.00026834	yes	down
ENSMUSG00000047419	Cmya5	cardiomyopathy associated 5 [Source:MGI Symbol;Acc:MGI:1923719]	2.613299161	1.385872288	1.21E-05	0.00026953	yes	up
ENSMUSG00000037071	Scd1	stearoyl-Coenzyme A desaturase 1 [Source:MGI	2.45240602	1.294197851	1.23E-05	0.00027189	yes	up
ENSMUSG00000031972	Acta1	actin, alpha 1, skeletal muscle [Source:MGI	2.172684398	1.119478625	1.23E-05	0.00027272	yes	up
ENSMUSG00000004105	Angptl2	angiopoietin-like 2 [Source:MGI Symbol;Acc:MGI:1347002]	2.175114493	1.121091343	1.25E-05	0.00027711	yes	up
ENSMUSG00000045629	Sh3tc2	SH3 domain and tetratricopeptide repeats 2 [Source:MGI Symbol;Acc:MGI:2444417]	0.46113468	-1.116739925	1.26E-05	0.00027902	yes	down
ENSMUSG00000026825	Dnm1	dynamin 1 [Source:MGI Symbol;Acc:MGI:107384]	2.487069927	1.314447071	1.27E-05	0.00027924	yes	up
ENSMUSG00000031480	Thsd1	thrombospondin, type I, domain 1 [Source:MGI Symbol;Acc:MGI:1929096]	0.476901857	-1.068235695	1.32E-05	0.00028987	yes	down
ENSMUSG00000029370	Rassf6	Ras association (RalGDS/AF-6) domain family member 6 [Source:MGI	0.348889562	-1.519157659	1.34E-05	0.00029267	yes	down
ENSMUSG00000026241	Nppc	natriuretic peptide type C [Source:MGI	0.270197039	-1.88791623	1.34E-05	0.00029301	yes	down
ENSMUSG00000053414	Hunk	hormonally upregulated Neu-associated kinase [Source:MGI Symbol;Acc:MGI:1347352]	0.362431505	-1.464219725	1.34E-05	0.00029315	yes	down
ENSMUSG00000113846	Gm6217	predicted gene 6217 [Source:MGI Symbol;Acc:MGI:3644079]	0.072520805	-3.785461246	1.35E-05	0.00029331	yes	down
ENSMUSG00000031112	Stk26	serine/threonine kinase 26 [Source:MGI	0.414908637	-1.269134406	1.36E-05	0.00029656	yes	down
ENSMUSG00000073295	Nudt11	nudix (nucleoside diphosphate linked moiety X)-type motif 11 [Source:MGI Symbol;Acc:MGI:1930957]	0.39971173	-1.322968182	1.40E-05	0.00030302	yes	down
ENSMUSG00000044294	Krt84	keratin 84 [Source:MGI Symbol;Acc:MGI:96700]	0.264542964	-1.918426046	1.41E-05	0.00030418	yes	down
ENSMUSG00000024304	Cdh2	cadherin 2 [Source:MGI Symbol;Acc:MGI:88355]	2.14705656	1.102360197	1.41E-05	0.00030418	yes	up
ENSMUSG00000010830	Kdelr3	KDEL (Lys-Asp-Glu-Leu) endoplasmic reticulum protein retention receptor 3 [Source:MGI Symbol;Acc:MGI:2145953]	2.139690064	1.097401836	1.41E-05	0.00030474	yes	up
ENSMUSG00000039774	Galnt12	polypeptide N-acetylgalactosaminyltransferase 12 [Source:MGI Symbol;Acc:MGI:2444664]	2.28334636	1.191149718	1.49E-05	0.00031951	yes	up
ENSMUSG00000020473	Aebp1	AE binding protein 1 [Source:MGI Symbol;Acc:MGI:1197012]	2.707393692	1.43690469	1.51E-05	0.00032203	yes	up
ENSMUSG00000026208	Des	desmin [Source:MGI Symbol;Acc:MGI:94885]	2.163619684	1.113446928	1.56E-05	0.00033248	yes	up
ENSMUSG00000038248	Sobp	sine oculis binding protein [Source:MGI	2.86315506	1.517605805	1.57E-05	0.00033523	yes	up
ENSMUSG00000031070	Mrgprf	MAS-related GPR, member F [Source:MGI Symbol;Acc:MGI:2384823]	2.721583085	1.444446079	1.58E-05	0.00033547	yes	up
ENSMUSG00000030401	Rtn2	reticulon 2 (Z-band associated protein) [Source:MGI Symbol;Acc:MGI:107612]	2.036314549	1.025960431	1.58E-05	0.00033582	yes	up
ENSMUSG00000010760	Phlda2	pleckstrin homology like domain, family A, member 2 [Source:MGI Symbol;Acc:MGI:1202307]	0.291276719	-1.7795377	1.60E-05	0.00033836	yes	down

ENSMUSG00000031312	Itgb1bp2	integrin beta 1 binding protein 2 [Source:MGI Symbol;Acc:MGI:1353420]	2.709289745	1.437914691	1.60E-05	0.00033934	yes	up
ENSMUSG00000027318	Adam33	a disintegrin and metallopeptidase domain 33 [Source:MGI Symbol;Acc:MGI:1341813]	2.319179803	1.213614675	1.61E-05	0.00034001	yes	up
ENSMUSG00000026389	Steap3	STEAP family member 3 [Source:MGI	2.014375597	1.010332711	1.62E-05	0.00034117	yes	up
ENSMUSG00000022931	Krtap15	keratin associated protein 15 [Source:MGI Symbol;Acc:MGI:1347350]	0.06653101	-3.909829247	1.62E-05	0.00034117	yes	down
ENSMUSG00000103523	2210017I01Rik	RIKEN cDNA 2210017I01 gene [Source:MGI Symbol;Acc:MGI:3588251]	0.308216904	-1.697982109	1.62E-05	0.00034117	yes	down
ENSMUSG00000051316	Taf7	TATA-box binding protein associated factor 7 [Source:MGI Symbol;Acc:MGI:1346348]	0.474966769	-1.074101516	1.64E-05	0.00034357	yes	down
ENSMUSG00000036564	Ndrg4	N-myc downstream regulated gene 4 [Source:MGI Symbol;Acc:MGI:2384590]	2.069490602	1.049275697	1.67E-05	0.00034958	yes	up
ENSMUSG00000058914	C1qtnf3	C1q and tumor necrosis factor related protein 3 [Source:MGI Symbol;Acc:MGI:1932136]	2.921557598	1.546737732	1.73E-05	0.00035998	yes	up
ENSMUSG00000067377	Tspan6	tetraspanin 6 [Source:MGI Symbol;Acc:MGI:1926264]	0.473278844	-1.079237663	1.75E-05	0.00036489	yes	down
ENSMUSG00000033715	Akr1c14	aldo-keto reductase family 1, member C14 [Source:MGI Symbol;Acc:MGI:2145458]	2.528407051	1.338228743	1.76E-05	0.00036505	yes	up
ENSMUSG00000030471	Zdhhc13	zinc finger, DHHC domain containing 13 [Source:MGI Symbol;Acc:MGI:1919227]	0.471274628	-1.085360083	1.76E-05	0.00036505	yes	down
ENSMUSG00000030935	Acsm3	acyl-CoA synthetase medium-chain family member 3 [Source:MGI Symbol;Acc:MGI:99538]	0.344084389	-1.539165657	1.88E-05	0.00038773	yes	down
ENSMUSG00000081194	Gm8424	predicted gene 8424 [Source:MGI Symbol;Acc:MGI:3646584]	3.467164896	1.793756452	1.92E-05	0.00039506	yes	up
ENSMUSG00000027887	Sypl2	synaptophysin-like 2 [Source:MGI Symbol;Acc:MGI:1328311]	2.649103798	1.405504373	1.95E-05	0.00039904	yes	up
ENSMUSG00000022546	Gpt	glutamic pyruvic transaminase, soluble [Source:MGI	2.219222877	1.150054565	1.98E-05	0.00040372	yes	up
ENSMUSG00000045027	Prss22	protease, serine 22 [Source:MGI Symbol;Acc:MGI:1918085]	0.498033358	-1.005685717	1.98E-05	0.00040372	yes	down
ENSMUSG00000105039	Gm32585	predicted gene, 32585 [Source:MGI Symbol;Acc:MGI:5591744]	0.079854307	-3.646485969	2.00E-05	0.00040721	yes	down
ENSMUSG00000081205	Gm5940	predicted gene 5940 [Source:MGI Symbol;Acc:MGI:3648847]	0.246116278	-2.022588014	2.01E-05	0.00040879	yes	down
ENSMUSG00000094546	Ighv1-26	immunoglobulin heavy variable 1-26 [Source:MGI Symbol;Acc:MGI:4439641]	3.179224585	1.668674934	2.02E-05	0.0004099	yes	up
ENSMUSG00000027204	Fbn1	fibrillin 1 [Source:MGI Symbol;Acc:MGI:95489]	2.459761755	1.298518588	2.03E-05	0.00041068	yes	up
ENSMUSG00000035258	Abi3bp	ABI family member 3 binding protein [Source:MGI Symbol;Acc:MGI:2444583]	2.128847713	1.09007275	2.04E-05	0.00041307	yes	up
ENSMUSG00000038119	Cdon	cell adhesion molecule-related/down-regulated by oncogenes [Source:MGI Symbol;Acc:MGI:1926387]	2.499643	1.321722063	2.06E-05	0.00041477	yes	up
ENSMUSG00000020334	Slc22a4	solute carrier family 22 (organic cation transporter), member 4 [Source:MGI	2.421116002	1.275672204	2.06E-05	0.00041478	yes	up
ENSMUSG00000076674	Ighv3-8	immunoglobulin heavy variable V3-8 [Source:MGI Symbol;Acc:MGI:3645298]	5.17814593	2.372435624	2.06E-05	0.00041478	yes	up
ENSMUSG00000026043	Col3a1	collagen, type III, alpha 1 [Source:MGI	2.635654006	1.398160994	2.09E-05	0.00041893	yes	up
ENSMUSG00000038112	AW551984	expressed sequence AW551984 [Source:MGI Symbol;Acc:MGI:2143322]	2.490392728	1.316373269	2.11E-05	0.00042243	yes	up
ENSMUSG00000030616	Sytl2	synaptotagmin-like 2 [Source:MGI Symbol;Acc:MGI:1933366]	0.455717321	-1.13378889	2.12E-05	0.0004227	yes	down
ENSMUSG00000007805	Twist2	twist basic helix-loop-helix transcription factor 2 [Source:MGI Symbol;Acc:MGI:104685]	3.1823121	1.670075333	2.17E-05	0.00043243	yes	up
ENSMUSG00000024526	Cidea	cell death-inducing DNA fragmentation factor, alpha subunit-like effector A [Source:MGI Symbol;Acc:MGI:1270845]	2.092324036	1.065106297	2.21E-05	0.0004391	yes	up
ENSMUSG00000021636	Marveld2	MARVEL (membrane-associating) domain containing 2 [Source:MGI Symbol;Acc:MGI:2446166]	0.482069198	-1.052687845	2.26E-05	0.00044806	yes	down
ENSMUSG00000027848	Olfml3	olfactomedin-like 3 [Source:MGI Symbol;Acc:MGI:1914877]	2.238858985	1.162763663	2.28E-05	0.00045056	yes	up
ENSMUSG00000055675	Kbtbd11	kelch repeat and BTB (POZ) domain containing 11 [Source:MGI Symbol;Acc:MGI:1922151]	2.034311989	1.024540953	2.29E-05	0.00045197	yes	up

ENSMUSG00000020216	Jsrp1	junctional sarcoplasmic reticulum protein 1 [Source:MGI Symbol;Acc:MGI:1916700]	2.412184943	1.270340523	2.36E-05	0.00046397	yes	up
ENSMUSG00000039131	Gipc2	GIPC PDZ domain containing family, member 2 [Source:MGI Symbol;Acc:MGI:1889209]	0.4338677	-1.20467291	2.40E-05	0.00046933	yes	down
ENSMUSG00000036960	Clca2	chloride channel accessory 2 [Source:MGI Symbol;Acc:MGI:2139758]	2.007773613	1.005596607	2.41E-05	0.00046987	yes	up
ENSMUSG00000023046	Igfbp6	insulin-like growth factor binding protein 6 [Source:MGI Symbol;Acc:MGI:96441]	2.204835106	1.140670765	2.45E-05	0.00047739	yes	up
ENSMUSG00000076598	Igkv3-7	immunoglobulin kappa variable 3-7 [Source:MGI Symbol;Acc:MGI:1330852]	2.314670495	1.210806833	2.46E-05	0.00048015	yes	up
ENSMUSG00000000253	Gmpr	guanosine monophosphate reductase [Source:MGI Symbol;Acc:MGI:1913605]	2.259629547	1.176086271	2.48E-05	0.00048195	yes	up
ENSMUSG00000054196	Cthrc1	collagen triple helix repeat containing 1 [Source:MGI Symbol;Acc:MGI:1915838]	3.172260259	1.665511138	2.49E-05	0.00048291	yes	up
ENSMUSG00000027077	Smtnl1	smoothelin-like 1 [Source:MGI Symbol;Acc:MGI:1915928]	2.949731997	1.560583882	2.53E-05	0.00048846	yes	up
ENSMUSG00000025213	Kazald1	Kazal-type serine peptidase inhibitor domain 1 [Source:MGI Symbol;Acc:MGI:2147606]	2.379295502	1.250534462	2.55E-05	0.00049127	yes	up
ENSMUSG00000022357	Klhl38	kelch-like 38 [Source:MGI Symbol;Acc:MGI:3045310]	2.756056934	1.462605691	2.56E-05	0.00049175	yes	up
ENSMUSG00000037379	Spon2	spondin 2, extracellular matrix protein [Source:MGI Symbol;Acc:MGI:1923724]	2.439243236	1.286433627	2.56E-05	0.00049175	yes	up
ENSMUSG00000078853	Igtp	interferon gamma induced GTPase [Source:MGI Symbol;Acc:MGI:107729]	2.583474154	1.369312451	2.56E-05	0.00049175	yes	up
ENSMUSG00000001622	Csn3	casein kappa [Source:MGI Symbol;Acc:MGI:107461]	0.100749757	-3.311151743	2.60E-05	0.00049866	yes	down
ENSMUSG00000054759	Krtap5-2	keratin associated protein 5-2 [Source:MGI Symbol;Acc:MGI:1918873]	0.025833986	-5.27458595	2.61E-05	0.00049871	yes	down
ENSMUSG00000037348	Paqr7	progestin and adipoQ receptor family member VII [Source:MGI Symbol;Acc:MGI:1919154]	2.590162482	1.373042601	2.61E-05	0.00049871	yes	up
ENSMUSG00000038179	Slamf7	SLAM family member 7 [Source:MGI Symbol;Acc:MGI:1922595]	2.760409291	1.464882194	2.69E-05	0.00051197	yes	up
ENSMUSG00000013584	Aldh1a2	aldehyde dehydrogenase family 1, subfamily A2 [Source:MGI Symbol;Acc:MGI:107928]	0.184231182	-2.440410827	2.69E-05	0.00051197	yes	down
ENSMUSG00000003555	Cyp17a1	cytochrome P450, family 17, subfamily a, polypeptide 1 [Source:MGI	3.451315108	1.787146199	2.75E-05	0.00052248	yes	up
ENSMUSG00000003418	St8sia6	ST8 alpha-N-acetyl-neuraminide alpha-2,8-sialyltransferase 6 [Source:MGI	0.347885934	-1.523313746	2.79E-05	0.00052934	yes	down
ENSMUSG00000000983	Wfdc18	WAP four-disulfide core domain 18 [Source:MGI Symbol;Acc:MGI:107506]	0.378478071	-1.401718383	2.84E-05	0.00053684	yes	down
ENSMUSG00000025461	Scart1	scavenger receptor family member expressed on T cells 1 [Source:MGI Symbol;Acc:MGI:2443796]	3.3263819	1.733953813	2.92E-05	0.0005484	yes	up
ENSMUSG00000001604	Tcea3	transcription elongation factor A (SII), 3 [Source:MGI Symbol;Acc:MGI:1196908]	2.282621531	1.190691674	2.97E-05	0.00055888	yes	up
ENSMUSG00000031595	Pdgfrl	platelet-derived growth factor receptor-like [Source:MGI Symbol;Acc:MGI:1916047]	2.348995694	1.232044069	2.98E-05	0.00055948	yes	up
ENSMUSG00000031489	Adrb3	adrenergic receptor, beta 3 [Source:MGI	2.155038322	1.107713524	3.03E-05	0.00056743	yes	up
ENSMUSG00000000753	Serpinf1	serine (or cysteine) peptidase inhibitor, clade F, member 1 [Source:MGI	2.04977772	1.035467471	3.07E-05	0.00057362	yes	up
ENSMUSG00000055409	Nell1	NEL-like 1 [Source:MGI Symbol;Acc:MGI:2443902]	5.197743771	2.377885516	3.13E-05	0.00058448	yes	up
ENSMUSG00000078132	Gm11939	predicted gene 11939 [Source:MGI Symbol;Acc:MGI:3651443]	0.110086943	-3.183284733	3.16E-05	0.00058943	yes	down
ENSMUSG00000059049	Frem1	Fras1 related extracellular matrix protein 1 [Source:MGI Symbol;Acc:MGI:2670972]	2.701165254	1.433581905	3.17E-05	0.00059008	yes	up
ENSMUSG00000029685	Asb15	ankyrin repeat and SOCS box-containing 15 [Source:MGI Symbol;Acc:MGI:1926160]	4.893442876	2.290849858	3.31E-05	0.00061423	yes	up
ENSMUSG00000063142	Kcnma1	potassium large conductance calcium-activated channel, subfamily M, alpha member 1 [Source:MGI Symbol;Acc:MGI:99923]	3.365613671	1.750869584	3.36E-05	0.00062398	yes	up
ENSMUSG00000010476	Ebf3	early B cell factor 3 [Source:MGI Symbol;Acc:MGI:894289]	2.049149776	1.035025438	3.39E-05	0.00062774	yes	up

ENSMUSG00000038370	Pcp4l1	Purkinje cell protein 4-like 1 [Source:MGI Symbol;Acc:MGI:1913675]	2.626099578	1.392921622	3.39E-05	0.00062823	yes	up
ENSMUSG00000022871	Fetub	fetuin beta [Source:MGI Symbol;Acc:MGI:1890221]	2.265856217	1.180056316	3.40E-05	0.00062898	yes	up
ENSMUSG00000096361	Gm5814	predicted pseudogene 5814 [Source:MGI Symbol;Acc:MGI:3648910]	0.25025668	-1.998519517	3.41E-05	0.00062898	yes	down
ENSMUSG00000033278	Ptprm	protein tyrosine phosphatase, receptor type, M [Source:MGI Symbol;Acc:MGI:102694]	2.004187429	1.003017434	3.42E-05	0.00063154	yes	up
ENSMUSG00000049404	Rarres1	retinoic acid receptor responder (tazarotene induced) 1 [Source:MGI Symbol;Acc:MGI:1924461]	2.611727374	1.385004309	3.46E-05	0.00063623	yes	up
ENSMUSG00000078680	Mup10	major urinary protein 10 [Source:MGI Symbol;Acc:MGI:1924164]	2.593983065	1.375169061	3.48E-05	0.00063919	yes	up
ENSMUSG00000046248	Krtap5-3	keratin associated protein 5-3 [Source:MGI Symbol;Acc:MGI:1924465]	0.049316176	-4.341795244	3.53E-05	0.00064581	yes	down
ENSMUSG00000053318	Slamf8	SLAM family member 8 [Source:MGI Symbol;Acc:MGI:1921998]	2.593361892	1.374823542	3.55E-05	0.00064781	yes	up
ENSMUSG00000021200	Asb2	ankyrin repeat and SOCS box-containing 2 [Source:MGI Symbol;Acc:MGI:1929743]	2.379832253	1.250859886	3.56E-05	0.00065004	yes	up
ENSMUSG00000048852	Gm12185	predicted gene 12185 [Source:MGI Symbol;Acc:MGI:3652173]	7.04032723	2.815642486	3.62E-05	0.00065932	yes	up
ENSMUSG00000038777	Sema6c	sema domain, transmembrane domain (TM), and cytoplasmic domain, (semaphorin) 6C [Source:MGI Symbol;Acc:MGI:1338032]	2.063589548	1.045156044	3.67E-05	0.00066585	yes	up
ENSMUSG00000005054	Cstb	cystatin B [Source:MGI Symbol;Acc:MGI:109514]	0.454245792	-1.138454944	3.73E-05	0.00067447	yes	down
ENSMUSG00000017417	Plxdc1	plexin domain containing 1 [Source:MGI Symbol;Acc:MGI:1919574]	2.858190632	1.515102143	3.73E-05	0.00067447	yes	up
ENSMUSG00000017817	Jph2	junctophilin 2 [Source:MGI Symbol;Acc:MGI:1891496]	2.312812325	1.209648202	3.80E-05	0.00068661	yes	up
ENSMUSG00000069920	B3gnt9	UDP-GlcNAc:betaGal beta-1,3-N-acetylglucosaminyltransferase 9 [Source:MGI Symbol;Acc:MGI:2142841]	2.275925435	1.186453292	3.86E-05	0.00069561	yes	up
ENSMUSG00000078504	Aadacl4fm5	AADACL4 family member 5 [Source:MGI Symbol;Acc:MGI:2685284]	3.104262707	1.634250655	3.99E-05	0.0007152	yes	up
ENSMUSG00000024727	Trpm6	transient receptor potential cation channel, subfamily M, member 6 [Source:MGI Symbol;Acc:MGI:2675603]	0.281032594	-1.83119063	4.02E-05	0.00072087	yes	down
ENSMUSG00000074063	Osgin1	oxidative stress induced growth inhibitor 1 [Source:MGI Symbol;Acc:MGI:1919089]	0.458432127	-1.125219944	4.08E-05	0.00072868	yes	down
ENSMUSG00000001520	Nrip2	nuclear receptor interacting protein 2 [Source:MGI Symbol;Acc:MGI:1891884]	2.333185425	1.222300967	4.10E-05	0.00073054	yes	up
ENSMUSG00000057969	Sema3b	sema domain, immunoglobulin domain (Ig), short basic domain, secreted, (semaphorin) 3B [Source:MGI	2.487917858	1.314938854	4.13E-05	0.00073498	yes	up
ENSMUSG00000046491	C1qtnf2	C1q and tumor necrosis factor related protein 2 [Source:MGI Symbol;Acc:MGI:1916433]	2.432891158	1.282671779	4.13E-05	0.00073537	yes	up
ENSMUSG00000018727	Cpsf4l	cleavage and polyadenylation specific factor 4-like [Source:MGI Symbol;Acc:MGI:1277182]	0.442493902	-1.17627052	4.14E-05	0.00073618	yes	down
ENSMUSG00000036278	Macrod1	mono-ADP ribosylhydrolase 1 [Source:MGI Symbol;Acc:MGI:2147583]	2.065618454	1.046573795	4.18E-05	0.00074135	yes	up
ENSMUSG00000028518	Prkaa2	protein kinase, AMP-activated, alpha 2 catalytic subunit [Source:MGI Symbol;Acc:MGI:1336173]	2.045579594	1.032509674	4.20E-05	0.00074412	yes	up
ENSMUSG00000057650	Krtap19-2	keratin associated protein 19-2 [Source:MGI Symbol;Acc:MGI:2157572]	0.064454118	-3.955583661	4.25E-05	0.0007513	yes	down
ENSMUSG00000027016	Zfp385b	zinc finger protein 385B [Source:MGI Symbol;Acc:MGI:2444734]	3.472479422	1.795966145	4.26E-05	0.00075281	yes	up
ENSMUSG00000028680	Plk3	polo like kinase 3 [Source:MGI Symbol;Acc:MGI:109604]	0.49363198	-1.018492232	4.27E-05	0.00075486	yes	down
ENSMUSG00000020722	Cacng1	calcium channel, voltage-dependent, gamma subunit 1 [Source:MGI Symbol;Acc:MGI:1206582]	2.290771534	1.195833581	4.34E-05	0.000765	yes	up
ENSMUSG00000060923	Acyp2	acylphosphatase 2, muscle type [Source:MGI Symbol;Acc:MGI:1922822]	2.03320453	1.02375535	4.35E-05	0.00076747	yes	up
ENSMUSG00000056481	Cd248	CD248 antigen, endosialin [Source:MGI	2.11067331	1.077703296	4.36E-05	0.00076772	yes	up

ENSMUSG00000024935	Slc1a1	solute carrier family 1 (neuronal/epithelial high affinity glutamate transporter, system Xag), member 1 [Source:MGI Symbol;Acc:MGI:105083]	0.421672628	-1.24580472	4.46E-05	0.00078367	yes	down
ENSMUSG00000019989	Enpp3	ectonucleotide pyrophosphatase/phosphodiesterase 3 [Source:MGI Symbol;Acc:MGI:2143702]	2.037637008	1.026897067	4.47E-05	0.00078367	yes	up
ENSMUSG00000025780	Itih5	inter-alpha (globulin) inhibitor H5 [Source:MGI Symbol;Acc:MGI:1925751]	2.303867676	1.204057857	4.50E-05	0.0007889	yes	up
ENSMUSG00000048699	Krt90	keratin 90 [Source:MGI Symbol;Acc:MGI:3045312]	0.298463756	-1.744372344	4.53E-05	0.00079253	yes	down
ENSMUSG00000024846	Cst6	cystatin E/M [Source:MGI Symbol;Acc:MGI:1920970]	0.436684898	-1.195335456	4.53E-05	0.00079253	yes	down
ENSMUSG00000028179	Cth	cystathionase (cystathionine gamma-lyase) [Source:MGI Symbol;Acc:MGI:1339968]	0.322629134	-1.632051371	4.54E-05	0.00079253	yes	down
ENSMUSG00000079110	Capn3	calpain 3 [Source:MGI Symbol;Acc:MGI:107437]	2.629923442	1.395020803	4.54E-05	0.00079253	yes	up
ENSMUSG00000040612	Ildr2	immunoglobulin-like domain containing receptor 2 [Source:MGI Symbol;Acc:MGI:1196370]	2.340484803	1.226807398	4.56E-05	0.00079417	yes	up
ENSMUSG00000062077	Trim54	tripartite motif-containing 54 [Source:MGI Symbol;Acc:MGI:1889623]	2.65912074	1.410949286	4.58E-05	0.00079653	yes	up
ENSMUSG00000024302	Dtna	dystrobrevin alpha [Source:MGI Symbol;Acc:MGI:106039]	2.510636244	1.328053018	4.59E-05	0.00079803	yes	up
ENSMUSG00000103144	Pcdhga1	protocadherin gamma subfamily A, 1 [Source:MGI Symbol;Acc:MGI:1935212]	2.619651392	1.389374839	4.61E-05	0.00080111	yes	up
ENSMUSG00000074006	Omp	olfactory marker protein [Source:MGI Symbol;Acc:MGI:97436]	0.158981919	-2.653065398	4.62E-05	0.00080204	yes	down
ENSMUSG00000021622	Ckmt2	creatine kinase, mitochondrial 2 [Source:MGI Symbol;Acc:MGI:1923972]	2.459577335	1.298410418	4.70E-05	0.00081377	yes	up
ENSMUSG00000085918	Gm13032	predicted gene 13032 [Source:MGI Symbol;Acc:MGI:3702685]	0.201979364	-2.307720192	4.72E-05	0.00081657	yes	down
ENSMUSG00000079362	Gm43302	predicted gene 43302 [Source:MGI Symbol;Acc:MGI:5663439]	5.358451187	2.421816063	4.73E-05	0.00081657	yes	up
ENSMUSG00000050808	Muc15	mucin 15 [Source:MGI Symbol;Acc:MGI:2442110]	0.48232741	-1.051915297	4.77E-05	0.00082249	yes	down
ENSMUSG00000061451	Tmem151a	transmembrane protein 151A [Source:MGI Symbol;Acc:MGI:2147713]	0.336235338	-1.572456738	4.80E-05	0.00082725	yes	down
ENSMUSG00000094935	Gm9726	predicted gene 9726 [Source:MGI Symbol;Acc:MGI:3648149]	0.013461589	-6.215007452	4.83E-05	1	yes	down
ENSMUSG00000037738	Nek5	NIMA (never in mitosis gene a)-related expressed kinase 5 [Source:MGI	0.23337005	-2.099308673	4.86E-05	0.00083624	yes	down
ENSMUSG00000057855	Krtap6-3	keratin associated protein 6-3 [Source:MGI Symbol;Acc:MGI:1330279]	0.363667169	-1.459309406	4.91E-05	0.00084307	yes	down
ENSMUSG00000081683	Fzd10	frizzled class receptor 10 [Source:MGI	0.458306261	-1.125616099	4.99E-05	0.00085366	yes	down
ENSMUSG00000058665	En1	engrailed 1 [Source:MGI Symbol;Acc:MGI:95389]	2.510561056	1.328009811	5.04E-05	0.00086035	yes	up
ENSMUSG00000069874	Irgm2	immunity-related GTPase family M member 2 [Source:MGI Symbol;Acc:MGI:1926262]	2.48494283	1.313212661	5.05E-05	0.00086144	yes	up
ENSMUSG00000022816	Fstl1	follistatin-like 1 [Source:MGI Symbol;Acc:MGI:102793]	2.089194716	1.06294696	5.05E-05	0.00086144	yes	up
ENSMUSG00000079168	Cd209g	CD209g antigen [Source:MGI Symbol;Acc:MGI:1917442]	2.110617477	1.077665132	5.08E-05	0.00086498	yes	up
ENSMUSG00000033765	Calm4	calmodulin 4 [Source:MGI Symbol;Acc:MGI:1931464]	2.054296999	1.038644774	5.08E-05	0.00086498	yes	up
ENSMUSG00000105681	Gm43428	predicted gene 43428 [Source:MGI Symbol;Acc:MGI:5663565]	0.151168848	-2.725767225	5.09E-05	0.00086498	yes	down
ENSMUSG00000050761	Gp1bb	glycoprotein Ib, beta polypeptide [Source:MGI Symbol;Acc:MGI:107852]	0.321952781	-1.635078983	5.14E-05	0.00087249	yes	down
ENSMUSG00000004698	Hdac9	histone deacetylase 9 [Source:MGI Symbol;Acc:MGI:1931221]	2.227248794	1.155262723	5.19E-05	0.00087662	yes	up
ENSMUSG00000034227	Foxj1	forkhead box J1 [Source:MGI Symbol;Acc:MGI:1347474]	0.164891469	-2.600411334	5.29E-05	0.00089169	yes	down
ENSMUSG00000021720	Rnf180	ring finger protein 180 [Source:MGI Symbol;Acc:MGI:1919066]	0.456244663	-1.132120411	5.31E-05	0.00089485	yes	down
ENSMUSG00000028834	Trim63	tripartite motif-containing 63 [Source:MGI Symbol;Acc:MGI:2447992]	3.163666308	1.661597438	5.32E-05	0.00089485	yes	up
ENSMUSG00000068697	Myoz1	myozenin 1 [Source:MGI Symbol;Acc:MGI:1929471]	2.359686838	1.238595407	5.53E-05	0.00092321	yes	up
ENSMUSG00000106086	Lef1os1	LEF1 opposite strand RNA 1 [Source:MGI Symbol;Acc:MGI:5663489]	0.252991515	-1.982839098	5.62E-05	0.0009367	yes	down

ENSMUSG00000037206	Islr	immunoglobulin superfamily containing leucine-rich repeat [Source:MGI	2.09284757	1.065467238	5.70E-05	0.00094549	yes	up
ENSMUSG00000079105	C7	complement component 7 [Source:MGI	3.236383481	1.694382564	5.73E-05	0.00094867	yes	up
ENSMUSG00000029151	Slc30a3	solute carrier family 30 (zinc transporter), member 3 [Source:MGI Symbol;Acc:MGI:1345280]	0.17772704	-2.492264899	5.88E-05	0.00096982	yes	down
ENSMUSG00000105504	Gbp5	guanylate binding protein 5 [Source:MGI Symbol;Acc:MGI:2429943]	2.516028973	1.331148536	5.92E-05	0.00097499	yes	up
ENSMUSG00000046532	Ar	androgen receptor [Source:MGI Symbol;Acc:MGI:88064]	2.026458492	1.018960625	5.98E-05	0.00098401	yes	up
ENSMUSG00000087581	Gm11571	predicted gene 11571 [Source:MGI Symbol;Acc:MGI:3650087]	0.152137125	-2.716555848	5.98E-05	0.00098401	yes	down
ENSMUSG00000043631	Ecm2	extracellular matrix protein 2, female organ and adipocyte specific [Source:MGI Symbol;Acc:MGI:3039578]	2.516994397	1.331702005	5.99E-05	0.00098438	yes	up
ENSMUSG00000047746	Fbxo40	F-box protein 40 [Source:MGI Symbol;Acc:MGI:2443753]	2.261889715	1.177528588	6.15E-05	0.00100626	yes	up
ENSMUSG00000035279	Ssc5d	scavenger receptor cysteine rich family, 5 domains [Source:MGI Symbol;Acc:MGI:3606211]	2.783496731	1.476898389	6.15E-05	0.00100626	yes	up
ENSMUSG00000020889	Nr1d1	nuclear receptor subfamily 1, group D, member 1 [Source:MGI Symbol;Acc:MGI:2444210]	2.644741623	1.403126786	6.18E-05	0.00101019	yes	up
ENSMUSG00000020866	Cacna1g	calcium channel, voltage-dependent, T type, alpha 1G subunit [Source:MGI	3.006481838	1.588076243	6.21E-05	0.0010152	yes	up
ENSMUSG00000059602	Syn3	synapsin III [Source:MGI Symbol;Acc:MGI:1351334]	0.354278126	-1.497045701	6.34E-05	0.00103225	yes	down
ENSMUSG00000039384	Dusp10	dual specificity phosphatase 10 [Source:MGI Symbol;Acc:MGI:1927070]	0.452044344	-1.145463793	6.46E-05	0.00105081	yes	down
ENSMUSG00000029561	Oasl2	2'-5' oligoadenylate synthetase-like 2 [Source:MGI Symbol;Acc:MGI:1344390]	2.075766754	1.053644343	6.51E-05	0.00105698	yes	up
ENSMUSG00000021090	Lrrc9	leucine rich repeat containing 9 [Source:MGI Symbol;Acc:MGI:1925507]	5.486921775	2.455997008	6.60E-05	0.00106615	yes	up
ENSMUSG00000048764	Tmprss11f	transmembrane protease, serine 11f [Source:MGI Symbol;Acc:MGI:2442348]	0.439096911	-1.187388711	6.66E-05	0.00107331	yes	down
ENSMUSG00000036545	Adamts2	a disintegrin-like and metallopeptidase (reprolysin type) with thrombospondin type 1 motif, 2 [Source:MGI	2.166224499	1.115182766	6.70E-05	0.00107924	yes	up
ENSMUSG00000031028	Tub	tubby bipartite transcription factor [Source:MGI Symbol;Acc:MGI:2651573]	2.857976268	1.514993936	6.82E-05	0.00109726	yes	up
ENSMUSG00000025165	Sectm1a	secreted and transmembrane 1A [Source:MGI Symbol;Acc:MGI:2384805]	2.23587979	1.160842625	6.85E-05	0.00109969	yes	up
ENSMUSG00000092572	Serpinb10	serine (or cysteine) peptidase inhibitor, clade B (ovalbumin), member 10 [Source:MGI Symbol;Acc:MGI:2138648]	2.384549967	1.253717014	6.86E-05	0.00109969	yes	up
ENSMUSG00000014453	Blk	B lymphoid kinase [Source:MGI Symbol;Acc:MGI:88169]	3.186500338	1.671972814	6.92E-05	0.00110838	yes	up
ENSMUSG00000038201	Kcna7	potassium voltage-gated channel, shaker-related subfamily, member 7 [Source:MGI	2.361800547	1.239887135	6.93E-05	0.00110838	yes	up
ENSMUSG00000034765	Dusp5	dual specificity phosphatase 5 [Source:MGI Symbol;Acc:MGI:2685183]	0.441709222	-1.178831141	7.07E-05	0.00112673	yes	down
ENSMUSG00000049134	Nrap	nebulin-related anchoring protein [Source:MGI Symbol;Acc:MGI:1098765]	2.069817533	1.049503591	7.09E-05	0.0011274	yes	up
ENSMUSG00000036381	P2ry14	purinergic receptor P2Y, G-protein coupled, 14 [Source:MGI Symbol;Acc:MGI:2155705]	2.083411026	1.05894749	7.25E-05	0.00114891	yes	up
ENSMUSG00000085933	Tmem61	transmembrane protein 61 [Source:MGI	0.201334584	-2.312333084	7.27E-05	0.00115028	yes	down
ENSMUSG00000022686	B3gnt5	UDP-GlcNAc:betaGal beta-1,3-N-acetylglucosaminyltransferase 5 [Source:MGI Symbol;Acc:MGI:2137302]	0.283095434	-1.820639614	7.29E-05	0.00115157	yes	down
ENSMUSG00000022044	Stmn4	stathmin-like 4 [Source:MGI Symbol;Acc:MGI:1931224]	7.73306979	2.951041233	7.31E-05	0.00115354	yes	up
ENSMUSG00000027656	Ccn5	cellular communication network factor 5 [Source:MGI Symbol;Acc:MGI:1328326]	2.379186184	1.250468175	7.38E-05	0.0011636	yes	up
ENSMUSG00000086801	Gm15943	predicted gene 15943 [Source:MGI Symbol;Acc:MGI:3802102]	0.289767062	-1.787034484	7.39E-05	0.00116535	yes	down

ENSMUSG00000032024	Clmp	CXADR-like membrane protein [Source:MGI Symbol;Acc:MGI:1918816]	2.101275109	1.071265059	7.40E-05	0.00116579	yes	up
ENSMUSG00000111987	Gm48809	predicted gene, 48809 [Source:MGI Symbol;Acc:MGI:6098521]	0.172271981	-2.537240023	7.48E-05	0.00117623	yes	down
ENSMUSG00000041220	Elovl6	ELOVL family member 6, elongation of long chain fatty acids (yeast) [Source:MGI Symbol;Acc:MGI:2156528]	2.878578989	1.525356801	7.64E-05	0.00119566	yes	up
ENSMUSG00000069303	H2bc24	H2B clustered histone 24 [Source:MGI	0.043272545	-4.53040423	7.72E-05	0.00120726	yes	down
ENSMUSG00000026220	Slc16a14	solute carrier family 16 (monocarboxylic acid transporters), member 14 [Source:MGI Symbol;Acc:MGI:1919031]	2.103938786	1.07309273	7.75E-05	0.001211	yes	up
ENSMUSG00000025784	Clec3b	C-type lectin domain family 3, member b [Source:MGI Symbol;Acc:MGI:104540]	2.728661013	1.448193177	7.88E-05	0.00122777	yes	up
ENSMUSG00000006457	Actn3	actinin alpha 3 [Source:MGI Symbol;Acc:MGI:99678]	3.380583605	1.757272327	7.92E-05	0.0012322	yes	up
ENSMUSG00000038135	Crygn	crystallin, gamma N [Source:MGI Symbol;Acc:MGI:2449167]	5.58596876	2.481807505	7.94E-05	0.00123343	yes	up
ENSMUSG00000025978	Rftn2	raftlin family member 2 [Source:MGI Symbol;Acc:MGI:1921263]	2.359736026	1.23862548	7.95E-05	0.00123452	yes	up
ENSMUSG00000029868	Trpv6	transient receptor potential cation channel, subfamily V, member 6 [Source:MGI Symbol;Acc:MGI:1927259]	0.478777453	-1.062572883	8.02E-05	0.00124168	yes	down
ENSMUSG00000062591	Tubb4a	tubulin, beta 4A class IVA [Source:MGI Symbol;Acc:MGI:107848]	2.246785656	1.167862498	8.02E-05	0.00124168	yes	up
ENSMUSG00000085549	1700047F07Rik	RIKEN cDNA 1700047F07 gene [Source:MGI Symbol;Acc:MGI:1920570]	0.014313724	-6.126457075	8.10E-05	1	yes	down
ENSMUSG00000066975	Cryba4	crystallin, beta A4 [Source:MGI Symbol;Acc:MGI:102716]	0.090108739	-3.472189168	8.10E-05	0.001251	yes	down
ENSMUSG00000032355	Mlip	muscular LMNA-interacting protein [Source:MGI Symbol;Acc:MGI:1916892]	2.330938448	1.220910908	8.17E-05	0.0012598	yes	up
ENSMUSG00000074896	Ifit3	interferon-induced protein with tetratricopeptide repeats 3 [Source:MGI	2.551846073	1.351541308	8.17E-05	0.0012598	yes	up
ENSMUSG00000020312	Shc2	SHC (Src homology 2 domain containing) transforming protein 2 [Source:MGI Symbol;Acc:MGI:106180]	2.142409814	1.099234474	8.37E-05	0.00128573	yes	up
ENSMUSG00000036596	Cpz	carboxypeptidase Z [Source:MGI Symbol;Acc:MGI:88487]	2.420802749	1.275485531	8.51E-05	0.0013004	yes	up
ENSMUSG00000041741	Pde3a	phosphodiesterase 3A, cGMP inhibited [Source:MGI Symbol;Acc:MGI:1860764]	2.059250621	1.042119424	8.53E-05	0.00130279	yes	up
ENSMUSG00000020810	Cygb	cytoglobin [Source:MGI Symbol;Acc:MGI:2149481]	2.158174819	1.109811732	8.65E-05	0.00131809	yes	up
ENSMUSG00000038903	Ccdc68	coiled-coil domain containing 68 [Source:MGI Symbol;Acc:MGI:3612676]	0.29372892	-1.767442777	8.70E-05	0.0013215	yes	down
ENSMUSG00000022229	Atp12a	ATPase, H+/K+ transporting, nongastric, alpha polypeptide [Source:MGI Symbol;Acc:MGI:1926943]	0.494176789	-1.016900844	8.72E-05	0.00132413	yes	down
ENSMUSG00000117406	Ntn3	netrin 3 [Source:MGI Symbol;Acc:MGI:1341188]	2.45213897	1.294040743	8.79E-05	0.00133073	yes	up
ENSMUSG00000027661	Slc2a10	solute carrier family 2 (facilitated glucose transporter), member 10 [Source:MGI Symbol;Acc:MGI:2156687]	2.141131142	1.098373162	8.79E-05	0.00133073	yes	up
ENSMUSG00000028111	Ctsk	cathepsin K [Source:MGI Symbol;Acc:MGI:107823]	2.507893321	1.326475981	8.79E-05	0.00133073	yes	up
ENSMUSG00000016024	Lbp	lipopolysaccharide binding protein [Source:MGI Symbol;Acc:MGI:1098776]	2.28939908	1.19496897	8.90E-05	0.00134334	yes	up
ENSMUSG00000040536	Necab1	N-terminal EF-hand calcium binding protein 1 [Source:MGI Symbol;Acc:MGI:1916602]	0.18227876	-2.455781634	8.92E-05	0.00134559	yes	down
ENSMUSG00000062257	Opcml	opioid binding protein/cell adhesion molecule-like [Source:MGI Symbol;Acc:MGI:97397]	3.046609326	1.607204512	8.96E-05	0.00134939	yes	up
ENSMUSG00000022758	P2rx6	purinergic receptor P2X, ligand-gated ion channel, 6 [Source:MGI Symbol;Acc:MGI:1337113]	3.420623122	1.77425916	9.04E-05	0.00135957	yes	up
ENSMUSG00000002732	Fkbp7	FK506 binding protein 7 [Source:MGI Symbol;Acc:MGI:1336879]	2.007515369	1.005411032	9.06E-05	0.00136055	yes	up
ENSMUSG00000005947	Itgae	integrin alpha E, epithelial-associated [Source:MGI Symbol;Acc:MGI:1298377]	2.177692477	1.122800238	9.09E-05	0.00136304	yes	up

ENSMUSG00000042918	Mamstr	MEF2 activating motif and SAP domain containing transcriptional regulator [Source:MGI Symbol;Acc:MGI:1921740]	2.404749945	1.265886885	9.18E-05	0.00137454	yes	up
ENSMUSG00000019982	Myb	myeloblastosis oncogene [Source:MGI	0.418281205	-1.257454922	9.33E-05	0.00139366	yes	down
ENSMUSG00000032033	Barx2	BarH-like homeobox 2 [Source:MGI Symbol;Acc:MGI:109617]	0.369781538	-1.435254899	9.44E-05	0.00140791	yes	down
ENSMUSG00000111933	Gm36660	predicted gene, 36660 [Source:MGI Symbol;Acc:MGI:5595819]	0.210797664	-2.246069213	9.48E-05	0.00141072	yes	down
ENSMUSG00000036782	Klhl13	kelch-like 13 [Source:MGI Symbol;Acc:MGI:1914705]	2.067634654	1.047981287	9.79E-05	0.00145039	yes	up
ENSMUSG00000028037	Ifi44	interferon-induced protein 44 [Source:MGI Symbol;Acc:MGI:2443016]	2.221043756	1.151237815	9.85E-05	0.00145783	yes	up
ENSMUSG00000116656	Gm49708	predicted gene, 49708 [Source:MGI Symbol;Acc:MGI:6215173]	0.336333125	-1.572037219	9.86E-05	0.00145819	yes	down
ENSMUSG00000096715	Igkv3-4	immunoglobulin kappa variable 3-4 [Source:MGI Symbol;Acc:MGI:1330855]	2.179450851	1.12396467	9.95E-05	0.00147	yes	up
ENSMUSG00000070419	Cyp3a57	cytochrome P450, family 3, subfamily a, polypeptide 57 [Source:MGI Symbol;Acc:MGI:3646373]	4.796730814	2.26205148	0.00010016	0.0014774	yes	up
ENSMUSG00000068263	Efcc1	EF hand and coiled-coil domain containing 1 [Source:MGI Symbol;Acc:MGI:3611451]	2.911265264	1.541646299	0.00010042	0.00147871	yes	up
ENSMUSG00000024897	Apba1	amyloid beta (A4) precursor protein binding, family A, member 1 [Source:MGI Symbol;Acc:MGI:1860297]	2.157966893	1.109672731	0.00010061	0.00148021	yes	up
ENSMUSG00000091649	Phf11b	PHD finger protein 11B [Source:MGI Symbol;Acc:MGI:3645789]	2.125557523	1.087841302	0.00010072	0.00148058	yes	up
ENSMUSG00000017861	Mybl2	myeloblastosis oncogene-like 2 [Source:MGI Symbol;Acc:MGI:101785]	0.495622892	-1.012685271	0.00010085	0.00148128	yes	down
ENSMUSG00000054484	Tmem62	transmembrane protein 62 [Source:MGI	0.492471336	-1.021888338	0.00010138	0.00148649	yes	down
ENSMUSG00000071019	Sdr16c6	short chain dehydrogenase/reductase family 16C, member 6 [Source:MGI Symbol;Acc:MGI:2685269]	2.01686115	1.012111766	0.00010269	0.00150453	yes	up
ENSMUSG00000028865	Cd164l2	CD164 sialomucin-like 2 [Source:MGI	0.470318691	-1.088289426	0.00010315	0.00150999	yes	down
ENSMUSG00000007877	Tcap	titin-cap [Source:MGI Symbol;Acc:MGI:1330233]	2.338163876	1.225376049	0.00010495	0.00153239	yes	up
ENSMUSG00000000627	Sema4f	sema domain, immunoglobulin domain (Ig), TM domain, and short cytoplasmic domain [Source:MGI Symbol;Acc:MGI:1340055]	2.756151758	1.462655327	0.00010942	0.00159227	yes	up
ENSMUSG00000069806	Cacng7	calcium channel, voltage-dependent, gamma subunit 7 [Source:MGI Symbol;Acc:MGI:1932374]	2.533504017	1.341134116	0.0001101	0.00160077	yes	up
ENSMUSG00000002847	Pla1a	phospholipase A1 member A [Source:MGI Symbol;Acc:MGI:1934677]	2.119407246	1.08366083	0.00011121	0.00161558	yes	up
ENSMUSG00000034205	Loxl2	lysyl oxidase-like 2 [Source:MGI Symbol;Acc:MGI:2137913]	2.518015038	1.332286899	0.00011299	0.00163697	yes	up
ENSMUSG00000025909	Sntg1	syntrophin, gamma 1 [Source:MGI Symbol;Acc:MGI:1918346]	0.308383629	-1.697201917	0.00011541	0.00166681	yes	down
ENSMUSG00000025880	Smad7	SMAD family member 7 [Source:MGI	0.491229415	-1.025531144	0.0001158	0.00166969	yes	down
ENSMUSG00000045932	Ifit2	interferon-induced protein with tetratricopeptide repeats 2 [Source:MGI	2.381594243	1.251927639	0.00011691	0.00168435	yes	up
ENSMUSG00000039137	Whrn	whirlin [Source:MGI Symbol;Acc:MGI:2682003]	0.482059336	-1.052717359	0.00011735	0.00168777	yes	down
ENSMUSG00000030996	Art1	ADP-ribosyltransferase 1 [Source:MGI	2.254989441	1.173120678	0.00011958	0.00171582	yes	up
ENSMUSG00000104445	Rhbg	Rhesus blood group-associated B glycoprotein [Source:MGI Symbol;Acc:MGI:1927379]	0.499402747	-1.001724337	0.00011996	0.00171961	yes	down
ENSMUSG00000089789	Rdh1	retinol dehydrogenase 1 (all trans) [Source:MGI Symbol;Acc:MGI:1195275]	2.01543461	1.011090976	0.00012301	0.00175576	yes	up
ENSMUSG00000027514	Zbp1	Z-DNA binding protein 1 [Source:MGI	2.259885342	1.176249577	0.00012309	0.00175576	yes	up
ENSMUSG00000033044	Dhrs7c	dehydrogenase/reductase (SDR family) member 7C [Source:MGI Symbol;Acc:MGI:1915710]	4.514199792	2.174470272	0.00012349	0.00175995	yes	up
ENSMUSG00000001930	Vwf	Von Willebrand factor [Source:MGI Symbol;Acc:MGI:98941]	2.047327449	1.033741865	0.00012498	0.00177976	yes	up
ENSMUSG00000038963	Slco4a1	solute carrier organic anion transporter family, member 4a1 [Source:MGI	0.412253832	-1.27839519	0.00012528	0.00178053	yes	down

ENSMUSG00000032262	Elovl4	elongation of very long chain fatty acids (FEN1/Elo2, SUR4/Elo3, yeast)-like 4 [Source:MGI Symbol;Acc:MGI:1933331]	2.369962668	1.244864334	0.00012649	0.0017939	yes	up
ENSMUSG00000078675	Mup16	major urinary protein 16 [Source:MGI Symbol;Acc:MGI:3780250]	3.405603663	1.767910547	0.00012811	0.00181533	yes	up
ENSMUSG00000042249	Grk3	G protein-coupled receptor kinase 3 [Source:MGI	2.191854954	1.132152331	0.00012881	0.00182377	yes	up
ENSMUSG00000032649	Colgalt2	collagen beta(1-O)galactosyltransferase 2 [Source:MGI	2.570331629	1.361954511	0.00012915	0.00182711	yes	up
ENSMUSG00000025185	Loxl4	lysyl oxidase-like 4 [Source:MGI Symbol;Acc:MGI:1914823]	2.687965953	1.426514864	0.00013026	0.00183835	yes	up
ENSMUSG00000108413	BC026762	cDNA sequence BC026762 [Source:MGI Symbol;Acc:MGI:2652842]	0.390034368	-1.35832684	0.00013265	0.00186742	yes	down
ENSMUSG00000090314	2310050C09Rik	RIKEN cDNA 2310050C09 gene [Source:MGI Symbol;Acc:MGI:1913783]	2.501692553	1.322904499	0.00013351	0.00187686	yes	up
ENSMUSG00000029228	Lnx1	ligand of numb-protein X 1 [Source:MGI Symbol;Acc:MGI:1278335]	0.44329066	-1.173675131	0.00013387	0.00188008	yes	down
ENSMUSG00000047787	Flrt1	fibronectin leucine rich transmembrane protein 1 [Source:MGI	0.408755875	-1.290688628	0.00013478	0.00188674	yes	down
ENSMUSG00000078506	Aadacl4fm2	AADACL4 family member 2 [Source:MGI Symbol;Acc:MGI:3652194]	3.148114762	1.654488134	0.00013602	0.00190099	yes	up
ENSMUSG00000037016	Frem2	Fras1 related extracellular matrix protein 2 [Source:MGI Symbol;Acc:MGI:2444465]	2.572692048	1.363278777	0.00013726	0.00191445	yes	up
ENSMUSG00000051373	Plpp7	phospholipid phosphatase 7 (inactive) [Source:MGI Symbol;Acc:MGI:2445183]	2.075341821	1.053348976	0.00013782	0.00191987	yes	up
ENSMUSG00000018593	Sparc	secreted acidic cysteine rich glycoprotein [Source:MGI Symbol;Acc:MGI:98373]	2.146956631	1.102293049	0.00013909	0.00193134	yes	up
ENSMUSG00000052920	Prkg1	protein kinase, cGMP-dependent, type I [Source:MGI Symbol;Acc:MGI:108174]	2.301147486	1.202353452	0.00014037	0.00194452	yes	up
ENSMUSG00000015843	Rxrg	retinoid X receptor gamma [Source:MGI	2.2942504	1.198022859	0.00014149	0.00195534	yes	up
ENSMUSG00000033174	Mgll	monoglyceride lipase [Source:MGI Symbol;Acc:MGI:1346042]	2.496548971	1.319935206	0.0001418	0.00195802	yes	up
ENSMUSG00000022603	Mroh4	maestro heat-like repeat family member 4 [Source:MGI Symbol;Acc:MGI:1916689]	0.031005208	-5.011345618	0.00014409	0.00198325	yes	down
ENSMUSG00000042717	Ppp1r3a	protein phosphatase 1, regulatory subunit 3A [Source:MGI Symbol;Acc:MGI:2153588]	2.134041528	1.093588251	0.00014548	0.00200081	yes	up
ENSMUSG00000042751	Nmnat2	nicotinamide nucleotide adenylyltransferase 2 [Source:MGI Symbol;Acc:MGI:2444155]	0.397401648	-1.331330241	0.00014886	0.002036	yes	down
ENSMUSG00000021268	Meg3	maternally expressed 3 [Source:MGI Symbol;Acc:MGI:1202886]	2.319180232	1.213614942	0.00015454	0.00210042	yes	up
ENSMUSG00000056569	Mpz	myelin protein zero [Source:MGI Symbol;Acc:MGI:103177]	2.091003239	1.064195297	0.00015593	0.00211428	yes	up
ENSMUSG00000003476	Crhr2	corticotropin releasing hormone receptor 2 [Source:MGI Symbol;Acc:MGI:894312]	2.638205684	1.399557047	0.00015707	0.00212807	yes	up
ENSMUSG00000029120	Ppp2r2c	protein phosphatase 2, regulatory subunit B, gamma [Source:MGI Symbol;Acc:MGI:2442660]	2.188443058	1.129904847	0.00015888	0.00214927	yes	up
ENSMUSG00000074121	Ntf5	neurotrophin 5 [Source:MGI Symbol;Acc:MGI:97381]	0.44898769	-1.155252205	0.0001593	0.00215316	yes	down
ENSMUSG00000023191	P3h3	prolyl 3-hydroxylase 3 [Source:MGI Symbol;Acc:MGI:1315208]	2.019015197	1.01365177	0.00016016	0.00216309	yes	up
ENSMUSG00000046676	Lce1l	late cornified envelope 1L [Source:MGI	2.894964363	1.533545589	0.00016314	0.0021982	yes	up
ENSMUSG00000017446	C1qtnf1	C1q and tumor necrosis factor related protein 1 [Source:MGI Symbol;Acc:MGI:1919254]	2.39107191	1.257657519	0.00016449	0.00221477	yes	up
ENSMUSG00000087617	Gm14268	predicted gene 14268 [Source:MGI Symbol;Acc:MGI:3649848]	0.14250761	-2.810889132	0.00016465	0.00221521	yes	down
ENSMUSG00000037548	H2-DMb2	histocompatibility 2, class II, locus Mb2 [Source:MGI Symbol;Acc:MGI:95923]	2.190954802	1.131559723	0.00016518	0.002218	yes	up
ENSMUSG00000014602	Kif1a	kinesin family member 1A [Source:MGI	2.53298314	1.340837474	0.00016525	0.002218	yes	up
ENSMUSG00000031618	Nr3c2	nuclear receptor subfamily 3, group C, member 2 [Source:MGI Symbol;Acc:MGI:99459]	2.138801602	1.096802663	0.00016671	0.00223414	yes	up
ENSMUSG00000068606	Gm4841	predicted gene 4841 [Source:MGI Symbol;Acc:MGI:3643814]	4.974827917	2.314646623	0.00016723	0.00223855	yes	up

ENSMUSG00000026829	Gbgt1	globoside alpha-1,3-N-acetylgalactosaminyltransferase 1 [Source:MGI Symbol;Acc:MGI:2449143]	2.46559984	1.301938673	0.0001673	0.00223855	yes	up
ENSMUSG00000106139	Gm30648	predicted gene, 30648 [Source:MGI Symbol;Acc:MGI:5589807]	0.021943394	-5.510069501	0.00016762	1	yes	down
ENSMUSG00000056632	Dsg3	desmoglein 3 [Source:MGI Symbol;Acc:MGI:99499]	0.460914353	-1.117429401	0.0001717	0.00229043	yes	down
ENSMUSG00000006014	Prg4	proteoglycan 4 (megakaryocyte stimulating factor, articular superficial zone protein) [Source:MGI Symbol;Acc:MGI:1891344]	2.75671804	1.462951715	0.00017224	0.00229582	yes	up
ENSMUSG00000020435	Osbp2	oxysterol binding protein 2 [Source:MGI Symbol;Acc:MGI:1921559]	0.36773359	-1.443267132	0.00017397	0.00231353	yes	down
ENSMUSG00000078673	Mup19	major urinary protein 19 [Source:MGI Symbol;Acc:MGI:3705235]	2.766835457	1.46823685	0.00017411	0.00231359	yes	up
ENSMUSG00000034317	Trim59	tripartite motif-containing 59 [Source:MGI Symbol;Acc:MGI:1914199]	0.485627459	-1.042078098	0.00017486	0.00232177	yes	down
ENSMUSG00000044043	Pcdhb14	protocadherin beta 14 [Source:MGI Symbol;Acc:MGI:2136749]	2.198899865	1.136781907	0.00017595	0.00233085	yes	up
ENSMUSG00000041911	Dlx1	distal-less homeobox 1 [Source:MGI Symbol;Acc:MGI:94901]	0.368195224	-1.441457184	0.00017723	0.00234607	yes	down
ENSMUSG00000029158	Yipf7	Yip1 domain family, member 7 [Source:MGI Symbol;Acc:MGI:1922831]	2.991448136	1.58084405	0.00017827	0.00235805	yes	up
ENSMUSG00000024008	Cpne5	copine V [Source:MGI Symbol;Acc:MGI:2385908]	0.4049284	-1.304261264	0.00018183	0.0024014	yes	down
ENSMUSG00000040260	Daam2	dishevelled associated activator of morphogenesis 2 [Source:MGI Symbol;Acc:MGI:1923691]	2.369034095	1.244298962	0.00018226	0.00240527	yes	up
ENSMUSG00000039891	Txlnb	taxilin beta [Source:MGI Symbol;Acc:MGI:2671945]	2.193767299	1.133410502	0.00018249	0.00240641	yes	up
ENSMUSG00000031486	Adgra2	adhesion G protein-coupled receptor A2 [Source:MGI Symbol;Acc:MGI:1925810]	2.14096924	1.098264068	0.00018366	0.00241817	yes	up
ENSMUSG00000019539	Rcn3	reticulocalbin 3, EF-hand calcium binding domain [Source:MGI Symbol;Acc:MGI:1277122]	2.061968431	1.044022245	0.00018651	0.00245019	yes	up
ENSMUSG00000020553	Pctp	phosphatidylcholine transfer protein [Source:MGI Symbol;Acc:MGI:107375]	2.909631776	1.540836587	0.00018705	0.00245542	yes	up
ENSMUSG00000041329	Atp1b2	ATPase, Na+/K+ transporting, beta 2 polypeptide [Source:MGI Symbol;Acc:MGI:88109]	2.139095916	1.097001174	0.00018721	0.00245561	yes	up
ENSMUSG00000073876	Gm13305	predicted gene 13305 [Source:MGI Symbol;Acc:MGI:3801997]	0.397371803	-1.331438591	0.00018841	0.00246943	yes	down
ENSMUSG00000036334	Igsf10	immunoglobulin superfamily, member 10 [Source:MGI Symbol;Acc:MGI:1923481]	2.621531219	1.390409727	0.00018903	0.00247449	yes	up
ENSMUSG00000067780	Pi15	peptidase inhibitor 15 [Source:MGI Symbol;Acc:MGI:1934659]	0.417311272	-1.260804204	0.00018908	0.00247449	yes	down
ENSMUSG00000110980	Gm47204	predicted gene, 47204 [Source:MGI Symbol;Acc:MGI:6096003]	2.00803337	1.005783245	0.00018976	0.00248148	yes	up
ENSMUSG00000056133	Unc93a2	unc-93 homolog A2 [Source:MGI Symbol;Acc:MGI:3712668]	0.476053942	-1.070803038	0.00019047	0.00248707	yes	down
ENSMUSG00000000125	Wnt3	wingless-type MMTV integration site family, member 3 [Source:MGI Symbol;Acc:MGI:98955]	0.362926462	-1.462250842	0.00019123	0.00249501	yes	down
ENSMUSG00000068105	Tnfrsf13c	tumor necrosis factor receptor superfamily, member 13c [Source:MGI	0.20868204	-2.260621656	0.00019163	0.00249835	yes	down
ENSMUSG00000031253	Srpx2	sushi-repeat-containing protein, X-linked 2 [Source:MGI Symbol;Acc:MGI:1916042]	2.209834269	1.143938176	0.00019374	0.00252025	yes	up
ENSMUSG00000035296	Sgcg	sarcoglycan, gamma (dystrophin-associated glycoprotein) [Source:MGI Symbol;Acc:MGI:1346524]	2.119397868	1.083654446	0.00019668	0.00255077	yes	up
ENSMUSG00000038641	Akr1d1	aldo-keto reductase family 1, member D1 [Source:MGI Symbol;Acc:MGI:2384785]	2.944689243	1.558115393	0.00019765	0.0025615	yes	up
ENSMUSG00000008206	Cers4	ceramide synthase 4 [Source:MGI Symbol;Acc:MGI:1914510]	2.429473363	1.280643615	0.00019921	0.00257403	yes	up
ENSMUSG00000023968	Crip3	cysteine-rich protein 3 [Source:MGI Symbol;Acc:MGI:2152434]	0.201975697	-2.307746386	0.00019922	0.00257403	yes	down
ENSMUSG00000039542	Ncam1	neural cell adhesion molecule 1 [Source:MGI	2.250120336	1.170002158	0.00020005	0.00258287	yes	up
ENSMUSG00000042828	Trim72	tripartite motif-containing 72 [Source:MGI Symbol;Acc:MGI:3612190]	2.394438082	1.259687129	0.0002009	0.0025881	yes	up
ENSMUSG00000025739	Gng13	guanine nucleotide binding protein (G protein), gamma 13 [Source:MGI Symbol;Acc:MGI:1925616]	0.080621943	-3.632683637	0.00020335	0.00261569	yes	down
ENSMUSG00000022053	Ebf2	early B cell factor 2 [Source:MGI Symbol;Acc:MGI:894332]	2.203824422	1.14000929	0.00020553	0.00263586	yes	up

ENSMUSG00000022032	Scara5	scavenger receptor class A, member 5 [Source:MGI Symbol;Acc:MGI:1918395]	2.444649637	1.289627715	0.00020591	0.00263884	yes	up
ENSMUSG00000056367	Actr3b	ARP3 actin-related protein 3B [Source:MGI Symbol;Acc:MGI:2661120]	0.415479458	-1.267150946	0.00020639	0.00264297	yes	down
ENSMUSG00000051331	Cacna1c	calcium channel, voltage-dependent, L type, alpha 1C subunit [Source:MGI Symbol;Acc:MGI:103013]	2.137558937	1.095964198	0.00020785	0.00265918	yes	up
ENSMUSG00000024784	Gpha2	glycoprotein hormone alpha 2 [Source:MGI Symbol;Acc:MGI:2156541]	0.137325961	-2.864323706	0.00021048	0.0026854	yes	down
ENSMUSG00000085004	5430427M07Rik	RIKEN cDNA 5430427M07 gene [Source:MGI Symbol;Acc:MGI:1918599]	0.191286628	-2.386192068	0.00021241	0.00270404	yes	down
ENSMUSG00000094686	Ccl21a	chemokine (C-C motif) ligand 21A (serine) [Source:MGI Symbol;Acc:MGI:1349183]	2.477692589	1.308997201	0.00021279	0.00270563	yes	up
ENSMUSG00000020017	Hal	histidine ammonia lyase [Source:MGI Symbol;Acc:MGI:96010]	2.083029685	1.058683399	0.00021369	0.00271437	yes	up
ENSMUSG00000044033	Ccdc141	coiled-coil domain containing 141 [Source:MGI Symbol;Acc:MGI:1919735]	3.073901611	1.620070988	0.00021492	0.002728	yes	up
ENSMUSG00000089694	Nat8f7	N-acetyltransferase 8 (GCN5-related) family member 7 [Source:MGI Symbol;Acc:MGI:3782661]	3.860470144	1.948776555	0.00021552	0.00273357	yes	up
ENSMUSG00000104876	Trdc	T cell receptor delta, constant region [Source:MGI	2.545389194	1.347886264	0.00021631	0.00273785	yes	up
ENSMUSG00000024371	C2	complement component 2 (within H-2S) [Source:MGI	2.21385474	1.146560564	0.00021852	0.00276358	yes	up
ENSMUSG00000037736	Limch1	LIM and calponin homology domains 1 [Source:MGI Symbol;Acc:MGI:1924819]	2.019948074	1.014318206	0.00021963	0.00277551	yes	up
ENSMUSG00000010064	Slc38a3	solute carrier family 38, member 3 [Source:MGI Symbol;Acc:MGI:1923507]	0.398643011	-1.326830718	0.00022144	0.0027903	yes	down
ENSMUSG00000064080	Fbln2	fibulin 2 [Source:MGI Symbol;Acc:MGI:95488]	2.445142787	1.289918715	0.00022187	0.00279161	yes	up
ENSMUSG00000078920	Ifi47	interferon gamma inducible protein 47 [Source:MGI	2.028915567	1.020708829	0.00022368	0.00280838	yes	up
ENSMUSG00000043982	Krtap19-4	keratin associated protein 19-4 [Source:MGI Symbol;Acc:MGI:2157757]	0.054595166	-4.195082983	0.00022502	0.0028198	yes	down
ENSMUSG00000067276	Capn6	calpain 6 [Source:MGI Symbol;Acc:MGI:1100850]	2.048089272	1.034278601	0.00022782	0.00284993	yes	up
ENSMUSG00000022844	Pdia5	protein disulfide isomerase associated 5 [Source:MGI Symbol;Acc:MGI:1919849]	2.02403106	1.017231429	0.00023056	0.00287386	yes	up
ENSMUSG00000031448	Adprhl1	ADP-ribosylhydrolase like 1 [Source:MGI Symbol;Acc:MGI:2442168]	2.314434	1.210659422	0.00023057	0.00287386	yes	up
ENSMUSG00000029417	Cxcl9	chemokine (C-X-C motif) ligand 9 [Source:MGI Symbol;Acc:MGI:1352449]	2.832834028	1.502246079	0.00023179	0.0028829	yes	up
ENSMUSG00000022900	Ildr1	immunoglobulin-like domain containing receptor 1 [Source:MGI Symbol;Acc:MGI:2146574]	0.435674507	-1.198677397	0.00023378	0.00290548	yes	down
ENSMUSG00000087383	Gm12446	predicted gene 12446 [Source:MGI Symbol;Acc:MGI:3651433]	0.219544287	-2.187416104	0.00023441	0.00290996	yes	down
ENSMUSG00000027470	Mylk2	myosin, light polypeptide kinase 2, skeletal muscle [Source:MGI Symbol;Acc:MGI:2139434]	2.284405989	1.191819072	0.00023505	0.00291432	yes	up
ENSMUSG00000005763	Cd247	CD247 antigen [Source:MGI Symbol;Acc:MGI:88334]	2.03855915	1.027549818	0.00023533	0.00291432	yes	up
ENSMUSG00000081169	Gm12551	predicted gene 12551 [Source:MGI Symbol;Acc:MGI:3651664]	2.67872291	1.421545355	0.0002369	0.00293164	yes	up
ENSMUSG00000096464	Ighv2-2	immunoglobulin heavy variable 2-2 [Source:MGI Symbol;Acc:MGI:4439894]	5.568898477	2.477391992	0.00024268	0.00299252	yes	up
ENSMUSG00000028226	Mmp16	matrix metallopeptidase 16 [Source:MGI Symbol;Acc:MGI:1276107]	3.159926098	1.659890818	0.00024707	0.00303906	yes	up
ENSMUSG00000029675	Eln	elastin [Source:MGI Symbol;Acc:MGI:95317]	2.325281398	1.217405317	0.00024716	0.00303906	yes	up
ENSMUSG00000026193	Fn1	fibronectin 1 [Source:MGI Symbol;Acc:MGI:95566]	2.135102978	1.094305654	0.00024752	0.00304133	yes	up
ENSMUSG00000029661	Col1a2	collagen, type I, alpha 2 [Source:MGI Symbol;Acc:MGI:88468]	2.609648231	1.383855351	0.0002514	0.00308053	yes	up
ENSMUSG00000020256	Aldh1l2	aldehyde dehydrogenase 1 family, member L2 [Source:MGI Symbol;Acc:MGI:2444680]	2.466232363	1.302308734	0.00025303	0.00309582	yes	up
ENSMUSG00000078922	Tgtp1	T cell specific GTPase 1 [Source:MGI Symbol;Acc:MGI:98734]	2.22103507	1.151232173	0.00025402	0.00310572	yes	up
ENSMUSG00000081225	Cyp2j12	cytochrome P450, family 2, subfamily j, polypeptide 12 [Source:MGI Symbol;Acc:MGI:3717097]	4.963541627	2.311369892	0.00025641	0.00312683	yes	up

ENSMUSG00000018907	Alox12e	arachidonate lipoxygenase, epidermal [Source:MGI Symbol;Acc:MGI:1274790]	2.079053807	1.055927096	0.00025647	0.00312683	yes	up
ENSMUSG00000024678	Ms4a4d	membrane-spanning 4-domains, subfamily A, member 4D [Source:MGI	2.086154952	1.06084632	0.00025704	0.00312891	yes	up
ENSMUSG00000040483	Xaf1	XIAP associated factor 1 [Source:MGI	2.071952658	1.050991039	0.00025718	0.00312891	yes	up
ENSMUSG00000115186	Gm49417	predicted gene, 49417 [Source:MGI Symbol;Acc:MGI:6155048]	2.099807622	1.070257159	0.00026165	0.00317649	yes	up
ENSMUSG00000028036	Ptgfr	prostaglandin F receptor [Source:MGI Symbol;Acc:MGI:97796]	2.139115658	1.097014489	0.00026239	0.00318326	yes	up
ENSMUSG00000047686	Rtl3	retrotransposon Gag like 3 [Source:MGI Symbol;Acc:MGI:2685221]	2.800442956	1.485655041	0.0002644	0.00320057	yes	up
ENSMUSG00000018906	P4ha2	procollagen-proline, 2-oxoglutarate 4-dioxygenase (proline 4-hydroxylase), alpha II polypeptide [Source:MGI Symbol;Acc:MGI:894286]	2.190022433	1.130945648	0.00026561	0.00320449	yes	up
ENSMUSG00000064224	Gsdma3	gasdermin A3 [Source:MGI Symbol;Acc:MGI:3044668]	0.354966491	-1.494245256	0.00026739	0.00321907	yes	down
ENSMUSG00000036951	Aadacl2fm1	AADACL2 family member 1 [Source:MGI Symbol;Acc:MGI:3028051]	3.153873808	1.657124937	0.00026851	0.00322813	yes	up
ENSMUSG00000071342	Lsmem1	leucine-rich single-pass membrane protein 1 [Source:MGI Symbol;Acc:MGI:2685735]	6.376898953	2.672855021	0.00026939	0.00323461	yes	up
ENSMUSG00000036412	Arsi	arylsulfatase i [Source:MGI Symbol;Acc:MGI:2670959]	2.119416778	1.083667318	0.00026943	0.00323461	yes	up
ENSMUSG00000074071	Fam169b	family with sequence similarity 169, member B [Source:MGI Symbol;Acc:MGI:3644026]	0.400831523	-1.318932122	0.00027188	0.00325955	yes	down
ENSMUSG00000106290	Gm43429	predicted gene 43429 [Source:MGI Symbol;Acc:MGI:5663566]	0.216277984	-2.209041283	0.0002741	0.00328165	yes	down
ENSMUSG00000025082	Vwa2	von Willebrand factor A domain containing 2 [Source:MGI Symbol;Acc:MGI:2684334]	3.133182138	1.647628639	0.00027572	0.00329869	yes	up
ENSMUSG00000041420	Meis3	Meis homeobox 3 [Source:MGI Symbol;Acc:MGI:108519]	2.076843803	1.054392717	0.00028106	0.00335427	yes	up
ENSMUSG00000032816	Igdcc4	immunoglobulin superfamily, DCC subclass, member 4 [Source:MGI Symbol;Acc:MGI:1858497]	2.200873305	1.138076098	0.00028114	0.00335427	yes	up
ENSMUSG00000028597	Gpx7	glutathione peroxidase 7 [Source:MGI Symbol;Acc:MGI:1914555]	2.078339177	1.055431116	0.00028295	0.00337116	yes	up
ENSMUSG00000027107	Chrna1	cholinergic receptor, nicotinic, alpha polypeptide 1 (muscle) [Source:MGI Symbol;Acc:MGI:87885]	2.80265353	1.486793406	0.00028845	0.00341323	yes	up
ENSMUSG00000021763	Cspg4b	chondroitin sulfate proteoglycan 4B [Source:MGI Symbol;Acc:MGI:3040697]	2.396183019	1.260738105	0.00029041	0.00342709	yes	up
ENSMUSG00000044933	Sstr3	somatostatin receptor 3 [Source:MGI Symbol;Acc:MGI:98329]	0.220642142	-2.180219728	0.00029306	0.00345358	yes	down
ENSMUSG00000045314	Sowahb	sosondowah ankyrin repeat domain family member B [Source:MGI Symbol;Acc:MGI:1925338]	0.397786014	-1.329935542	0.00029789	0.00349492	yes	down
ENSMUSG00000032243	Itga11	integrin alpha 11 [Source:MGI Symbol;Acc:MGI:2442114]	2.261887149	1.177526951	0.00029818	0.00349492	yes	up
ENSMUSG00000049804	Armcx4	armadillo repeat containing, X-linked 4 [Source:MGI Symbol;Acc:MGI:2147887]	2.050141931	1.035723791	0.00029935	0.00350623	yes	up
ENSMUSG00000087644	Gm14703	predicted gene 14703 [Source:MGI Symbol;Acc:MGI:3705165]	0.280425794	-1.834309036	0.00029997	0.00351107	yes	down
ENSMUSG00000006205	Htra1	HtrA serine peptidase 1 [Source:MGI Symbol;Acc:MGI:1929076]	2.212359201	1.145585642	0.00030148	0.00352632	yes	up
ENSMUSG00000110439	Mup22	major urinary protein 22 [Source:MGI Symbol;Acc:MGI:5434675]	2.435258361	1.284074838	0.00030527	0.00356106	yes	up
ENSMUSG00000078937	Cpt1b	carnitine palmitoyltransferase 1b, muscle [Source:MGI Symbol;Acc:MGI:1098297]	2.003675503	1.002648882	0.00030986	0.00359469	yes	up
ENSMUSG00000112120	Gm32255	predicted gene, 32255 [Source:MGI Symbol;Acc:MGI:5591414]	0.354941506	-1.494346806	0.00030992	0.00359469	yes	down
ENSMUSG00000020908	Myh3	myosin, heavy polypeptide 3, skeletal muscle, embryonic [Source:MGI Symbol;Acc:MGI:1339709]	2.021591876	1.015491772	0.00031055	0.00359833	yes	up
ENSMUSG00000065952	Rps23rg1	ribosomal protein S23, retrogene 1 [Source:MGI Symbol;Acc:MGI:3612471]	0.200506347	-2.31828019	0.00031274	0.00361895	yes	down
ENSMUSG00000041731	Pgm5	phosphoglucomutase 5 [Source:MGI Symbol;Acc:MGI:1925668]	2.229011278	1.156403916	0.00031316	0.00362129	yes	up
ENSMUSG00000094910	D430019H16Rik	RIKEN cDNA D430019H16 gene [Source:MGI Symbol;Acc:MGI:2443127]	3.076202075	1.621150277	0.00031337	0.00362129	yes	up
ENSMUSG00000022416	Cacna1i	calcium channel, voltage-dependent, alpha 1I subunit [Source:MGI Symbol;Acc:MGI:2178051]	0.162992542	-2.617122142	0.00031608	0.00364538	yes	down

ENSMUSG00000036816	Atoh7	atonal bHLH transcription factor 7 [Source:MGI Symbol;Acc:MGI:1355553]	0.199760751	-2.323654941	0.00031784	0.00366074	yes	down
ENSMUSG00000055874	Foxi3	forkhead box I3 [Source:MGI Symbol;Acc:MGI:3511278]	7.025546047	2.81261036	0.0003203	0.00368419	yes	up
ENSMUSG00000074213	Gm10642	predicted gene 10642 [Source:MGI Symbol;Acc:MGI:3704338]	5.233303963	2.387722057	0.00032269	0.00370923	yes	up
ENSMUSG00000113010	Gm34084	predicted gene, 34084 [Source:MGI Symbol;Acc:MGI:5593243]	0.347164045	-1.526310558	0.00032484	0.00372894	yes	down
ENSMUSG00000022215	Fitm1	fat storage-inducing transmembrane protein 1 [Source:MGI Symbol;Acc:MGI:1915930]	2.533336772	1.341038876	0.000328	0.0037514	yes	up
ENSMUSG00000035934	Pknox2	Pbx/knotted 1 homeobox 2 [Source:MGI Symbol;Acc:MGI:2445415]	2.257400464	1.174662376	0.0003281	0.0037514	yes	up
ENSMUSG00000019027	Dnah1	dynein, axonemal, heavy chain 1 [Source:MGI Symbol;Acc:MGI:107721]	2.079873932	1.056496085	0.00033053	0.00377427	yes	up
ENSMUSG00000041670	Rims1	regulating synaptic membrane exocytosis 1 [Source:MGI Symbol;Acc:MGI:2152971]	0.266494563	-1.907821998	0.00033084	0.00377526	yes	down
ENSMUSG00000103092	Pcdha5	protocadherin alpha 5 [Source:MGI Symbol;Acc:MGI:1298371]	49.52754908	5.630159324	0.00033259	1	yes	up
ENSMUSG00000059412	Fxyd2	FXYD domain-containing ion transport regulator 2 [Source:MGI Symbol;Acc:MGI:1195260]	2.194982251	1.134209274	0.00033419	0.00380593	yes	up
ENSMUSG00000021388	Aspn	asporin [Source:MGI Symbol;Acc:MGI:1913945]	2.586739617	1.371134839	0.00033562	0.00381978	yes	up
ENSMUSG00000060044	Tmem26	transmembrane protein 26 [Source:MGI	2.56266677	1.357645894	0.00033606	0.00382191	yes	up
ENSMUSG00000112653	Gm36176	predicted gene, 36176 [Source:MGI Symbol;Acc:MGI:5595335]	0.076044931	-3.717004104	0.00033625	0.00382191	yes	down
ENSMUSG00000051980	Casr	calcium-sensing receptor [Source:MGI	11.0296923	3.463320639	0.00033873	0.00383999	yes	up
ENSMUSG00000026765	Lypd6b	LY6/PLAUR domain containing 6B [Source:MGI Symbol;Acc:MGI:1919147]	0.482976246	-1.049975859	0.00033914	0.00384128	yes	down
ENSMUSG00000101819	H2al1b	H2A histone family member L1B [Source:MGI Symbol;Acc:MGI:3650131]	0.01875885	-5.736284836	0.00033964	1	yes	down
ENSMUSG00000008153	Clstn3	calsyntenin 3 [Source:MGI Symbol;Acc:MGI:2178323]	3.430053026	1.77823088	0.0003404	0.00385128	yes	up
ENSMUSG00000031461	Myom2	myomesin 2 [Source:MGI Symbol;Acc:MGI:1328358]	2.071848695	1.050918648	0.00034195	0.00386076	yes	up
ENSMUSG00000063522	Ly6m	lymphocyte antigen 6 complex, locus M [Source:MGI Symbol;Acc:MGI:1914288]	2.151995512	1.105675069	0.00034213	0.00386076	yes	up
ENSMUSG00000019768	Esr1	estrogen receptor 1 (alpha) [Source:MGI Symbol;Acc:MGI:1352467]	2.068002999	1.048238278	0.00034354	0.00386917	yes	up
ENSMUSG00000076613	Ighg2b	immunoglobulin heavy constant gamma 2B [Source:MGI Symbol;Acc:MGI:96445]	2.424085906	1.277440827	0.00034424	0.00387447	yes	up
ENSMUSG00000096632	Igkv9-124	immunoglobulin kappa chain variable 9-124 [Source:MGI Symbol;Acc:MGI:3646892]	5.869763673	2.553302419	0.00034498	0.00387777	yes	up
ENSMUSG00000021898	Asb14	ankyrin repeat and SOCS box-containing 14 [Source:MGI Symbol;Acc:MGI:2655107]	2.6808912	1.422712671	0.00034764	0.00389245	yes	up
ENSMUSG00000076617	Ighm	immunoglobulin heavy constant mu [Source:MGI	2.439366482	1.28650652	0.00034852	0.00389982	yes	up
ENSMUSG00000032528	Vipr1	vasoactive intestinal peptide receptor 1 [Source:MGI Symbol;Acc:MGI:109272]	0.495672559	-1.012540704	0.00035334	0.00394353	yes	down
ENSMUSG00000023328	Ache	acetylcholinesterase [Source:MGI Symbol;Acc:MGI:87876]	2.764233609	1.466879545	0.000354	0.00394581	yes	up
ENSMUSG00000027316	Gfra4	glial cell line derived neurotrophic factor family receptor alpha 4 [Source:MGI	2.094381121	1.066523998	0.00035702	0.00396824	yes	up
ENSMUSG00000022330	Osr2	odd-skipped related 2 [Source:MGI Symbol;Acc:MGI:1930813]	2.459317459	1.298257976	0.00035716	0.00396824	yes	up
ENSMUSG00000029765	Plxna4	plexin A4 [Source:MGI Symbol;Acc:MGI:2179061]	2.825362484	1.498435972	0.00035914	0.00398164	yes	up
ENSMUSG00000059901	Adamts14	a disintegrin-like and metallopeptidase (reprolysin type) with thrombospondin type 1 motif, 14 [Source:MGI	2.575004281	1.364574831	0.00035998	0.00398675	yes	up
ENSMUSG00000020902	Ntn1	netrin 1 [Source:MGI Symbol;Acc:MGI:105088]	2.110834544	1.077813499	0.00036157	0.00400176	yes	up
ENSMUSG00000058145	Adamts17	a disintegrin-like and metallopeptidase (reprolysin type) with thrombospondin type 1 motif, 17 [Source:MGI	2.8964074	1.534264542	0.00036415	0.00402524	yes	up
ENSMUSG00000049670	Morn4	MORN repeat containing 4 [Source:MGI Symbol;Acc:MGI:2449568]	0.377205083	-1.406578979	0.00036457	0.00402722	yes	down

ENSMUSG00000024867	Pip5k1b	phosphatidylinositol-4-phosphate 5-kinase, type 1 beta [Source:MGI Symbol;Acc:MGI:107930]	0.437508137	-1.192618246	0.0003687	0.00406247	yes	down
ENSMUSG00000020928	Higd1b	HIG1 domain family, member 1B [Source:MGI Symbol;Acc:MGI:1922939]	2.319264149	1.213667143	0.00036962	0.00407004	yes	up
ENSMUSG00000047898	Ccr4	chemokine (C-C motif) receptor 4 [Source:MGI Symbol;Acc:MGI:107824]	0.416548266	-1.263444421	0.00037005	0.00407216	yes	down
ENSMUSG00000027801	Tm4sf4	transmembrane 4 superfamily member 4 [Source:MGI Symbol;Acc:MGI:2385173]	0.039538096	-4.660612803	0.00037209	0.00409205	yes	down
ENSMUSG00000054672	Scart2	scavenger receptor family member expressed on T cells 2 [Source:MGI Symbol;Acc:MGI:2443685]	2.388687345	1.256218032	0.00037244	0.00409326	yes	up
ENSMUSG00000082292	Gm12250	predicted gene 12250 [Source:MGI Symbol;Acc:MGI:3649299]	2.498950496	1.321322322	0.00037621	0.00412687	yes	up
ENSMUSG00000027254	Map1a	microtubule-associated protein 1 A [Source:MGI Symbol;Acc:MGI:1306776]	2.07823534	1.055359034	0.00038176	0.00417449	yes	up
ENSMUSG00000042115	Klhdc8a	kelch domain containing 8A [Source:MGI Symbol;Acc:MGI:2442630]	0.472892976	-1.080414382	0.00038367	0.00418583	yes	down
ENSMUSG00000026042	Col5a2	collagen, type V, alpha 2 [Source:MGI	2.24127298	1.164318375	0.0003871	0.00421957	yes	up
ENSMUSG00000013921	Clip3	CAP-GLY domain containing linker protein 3 [Source:MGI Symbol;Acc:MGI:1923936]	2.150641628	1.104767142	0.00038838	0.00422763	yes	up
ENSMUSG00000050704	2310061N02Rik	RIKEN cDNA 2310061N02 gene [Source:MGI Symbol;Acc:MGI:1916911]	0.0733085	-3.769875703	0.00039187	0.0042554	yes	down
ENSMUSG00000089960	Ugt1a1	UDP glucuronosyltransferase 1 family, polypeptide A1 [Source:MGI Symbol;Acc:MGI:98898]	2.203534181	1.139819276	0.00039321	0.0042673	yes	up
ENSMUSG00000117442	1810073O08Rik	RIKEN cDNA 1810073O08 gene [Source:MGI Symbol;Acc:MGI:1919535]	0.223785974	-2.159808479	0.00039729	0.00429791	yes	down
ENSMUSG00000025431	Crisp1	cysteine-rich secretory protein 1 [Source:MGI Symbol;Acc:MGI:102553]	0.053924099	-4.212926013	0.00039944	0.00431321	yes	down
ENSMUSG00000078674	Mup18	major urinary protein 18 [Source:MGI Symbol;Acc:MGI:3705220]	2.452848827	1.294458321	0.00040136	0.00433132	yes	up
ENSMUSG00000001103	Sebox	SEBOX homeobox [Source:MGI Symbol;Acc:MGI:108012]	0.226351492	-2.143363278	0.00041467	0.0044499	yes	down
ENSMUSG00000032492	Pth1r	parathyroid hormone 1 receptor [Source:MGI	2.215673833	1.147745519	0.00041551	0.00445344	yes	up
ENSMUSG00000054072	Iigp1	interferon inducible GTPase 1 [Source:MGI Symbol;Acc:MGI:1926259]	2.779775943	1.474968603	0.00041646	0.00445696	yes	up
ENSMUSG00000042976	9930038B18Rik	RIKEN cDNA 9930038B18 gene [Source:MGI Symbol;Acc:MGI:2442156]	0.308603027	-1.696175884	0.00041925	0.00447403	yes	down
ENSMUSG00000094420	Igkv10-96	immunoglobulin kappa variable 10-96 [Source:MGI Symbol;Acc:MGI:4439561]	2.193510449	1.133241579	0.00041984	0.00447755	yes	up
ENSMUSG00000010461	Eya4	EYA transcriptional coactivator and phosphatase 4 [Source:MGI Symbol;Acc:MGI:1337104]	2.631108967	1.395670999	0.000421	0.00448276	yes	up
ENSMUSG00000047793	Sned1	sushi, nidogen and EGF-like domains 1 [Source:MGI Symbol;Acc:MGI:3045960]	2.447222015	1.29114499	0.0004211	0.00448276	yes	up
ENSMUSG00000031250	Tnmd	tenomodulin [Source:MGI Symbol;Acc:MGI:1929885]	2.532490067	1.340556611	0.0004225	0.00448947	yes	up
ENSMUSG00000020638	Cmpk2	cytidine monophosphate (UMP-CMP) kinase 2, mitochondrial [Source:MGI Symbol;Acc:MGI:99830]	2.092152807	1.064988227	0.00042251	0.00448947	yes	up
ENSMUSG00000047712	Ust	uronyl-2-sulfotransferase [Source:MGI	2.004678075	1.003370577	0.00042724	0.00452858	yes	up
ENSMUSG00000068889	Lce1e	late cornified envelope 1E [Source:MGI	2.320979251	1.214733625	0.00042882	0.00453981	yes	up
ENSMUSG00000000792	Slc5a5	solute carrier family 5 (sodium iodide symporter), member 5 [Source:MGI Symbol;Acc:MGI:2149330]	0.312221127	-1.67935993	0.00042952	0.00454159	yes	down
ENSMUSG00000044676	Zfp612	zinc finger protein 612 [Source:MGI Symbol;Acc:MGI:2443465]	0.484514687	-1.045387696	0.00043097	0.00455411	yes	down
ENSMUSG00000026255	Efhd1	EF hand domain containing 1 [Source:MGI Symbol;Acc:MGI:1921607]	0.44261487	-1.175876176	0.00043623	0.00459854	yes	down
ENSMUSG00000068011	Mkrn2os	makorin, ring finger protein 2, opposite strand [Source:MGI Symbol;Acc:MGI:1917541]	0.460569716	-1.118508542	0.00043982	0.00462226	yes	down
ENSMUSG00000012017	Scarf2	scavenger receptor class F, member 2 [Source:MGI Symbol;Acc:MGI:1858430]	2.27544913	1.186151334	0.00044323	0.00465247	yes	up
ENSMUSG00000042638	Gucy2c	guanylate cyclase 2c [Source:MGI Symbol;Acc:MGI:106903]	0.175048257	-2.514175397	0.00044646	0.00467647	yes	down

ENSMUSG00000027919	Lce1g	late cornified envelope 1G [Source:MGI	2.191329878	1.131806681	0.00044751	0.00468139	yes	up
ENSMUSG00000075318	Scn2a	sodium channel, voltage-gated, type II, alpha [Source:MGI Symbol;Acc:MGI:98248]	2.416372185	1.272842685	0.00045875	0.00477732	yes	up
ENSMUSG00000000248	Clec2g	C-type lectin domain family 2, member g [Source:MGI Symbol;Acc:MGI:1918059]	2.569195334	1.361316581	0.000459	0.00477732	yes	up
ENSMUSG00000057003	Myh4	myosin, heavy polypeptide 4, skeletal muscle [Source:MGI Symbol;Acc:MGI:1339713]	26.82963638	4.745755597	0.00046482	0.00482342	yes	up
ENSMUSG00000001506	Col1a1	collagen, type I, alpha 1 [Source:MGI Symbol;Acc:MGI:88467]	2.663938839	1.41356096	0.0004674	0.00484142	yes	up
ENSMUSG00000075394	Hoxc4	homeobox C4 [Source:MGI Symbol;Acc:MGI:96195]	2.066449196	1.047153895	0.00046783	0.00484297	yes	up
ENSMUSG00000075511	1700001L05Rik	RIKEN cDNA 1700001L05 gene [Source:MGI Symbol;Acc:MGI:1916541]	0.466084224	-1.101337414	0.00047141	0.00487716	yes	down
ENSMUSG00000044052	Ccr10	chemokine (C-C motif) receptor 10 [Source:MGI Symbol;Acc:MGI:1096320]	3.157819908	1.658928896	0.00047416	0.00490268	yes	up
ENSMUSG00000073293	Nudt10	nudix (nucleoside diphosphate linked moiety X)-type motif 10 [Source:MGI Symbol;Acc:MGI:2147931]	0.36689335	-1.446567338	0.00047522	0.00491074	yes	down
ENSMUSG00000010122	Slc47a1	solute carrier family 47, member 1 [Source:MGI Symbol;Acc:MGI:1914723]	2.651730474	1.406934145	0.00047689	0.00492203	yes	up
ENSMUSG00000087408	Cers1	ceramide synthase 1 [Source:MGI Symbol;Acc:MGI:2136690]	3.381795977	1.757789625	0.00047905	0.00492966	yes	up
ENSMUSG00000028023	Pitx2	paired-like homeodomain transcription factor 2 [Source:MGI Symbol;Acc:MGI:109340]	3.115713024	1.639562359	0.00047998	0.0049363	yes	up
ENSMUSG00000068887	Lce1j	late cornified envelope 1J [Source:MGI	3.063887583	1.615363364	0.00050061	0.00510595	yes	up
ENSMUSG00000054256	Msi1	musashi RNA-binding protein 1 [Source:MGI Symbol;Acc:MGI:107376]	0.432213827	-1.210182868	0.00050605	0.00515224	yes	down
ENSMUSG00000030577	Cd22	CD22 antigen [Source:MGI Symbol;Acc:MGI:88322]	2.000766339	1.000552691	0.00050738	0.00516277	yes	up
ENSMUSG00000029671	Wnt16	wingless-type MMTV integration site family, member 16 [Source:MGI Symbol;Acc:MGI:2136018]	4.144853174	2.051320997	0.00051825	0.00525484	yes	up
ENSMUSG00000075033	Nxpe3	neurexophilin and PC-esterase domain family, member 3 [Source:MGI	0.46403195	-1.107703952	0.00052086	0.00527516	yes	down
ENSMUSG00000111339		post-GPI attachment to proteins 2	0.031851289	-4.972504436	0.00052241	0.00528773	yes	down
ENSMUSG00000079055	Slc8a3	solute carrier family 8 (sodium/calcium exchanger), member 3 [Source:MGI	2.553181037	1.352295838	0.0005268	0.00532282	yes	up
ENSMUSG00000118633	Gm21104	predicted gene, 21104 [Source:MGI Symbol;Acc:MGI:5434459]	3.214774398	1.684717498	0.00053085	0.00535743	yes	up
ENSMUSG00000047094	Ofcc1	orofacial cleft 1 candidate 1 [Source:MGI Symbol;Acc:MGI:2658851]	2.164282719	1.11388897	0.00053135	0.00535937	yes	up
ENSMUSG00000028167	Bdh2	3-hydroxybutyrate dehydrogenase, type 2 [Source:MGI Symbol;Acc:MGI:1917022]	2.257244473	1.17456268	0.00053998	0.00542776	yes	up
ENSMUSG00000050860	Phospho1	phosphatase, orphan 1 [Source:MGI Symbol;Acc:MGI:2447348]	2.282693574	1.190737207	0.00054299	0.00545137	yes	up
ENSMUSG00000021904	Sema3g	sema domain, immunoglobulin domain (Ig), short basic domain, secreted, (semaphorin) 3G [Source:MGI	2.04952413	1.035288976	0.00055305	0.00552666	yes	up
ENSMUSG00000038526	Car14	carbonic anhydrase 14 [Source:MGI Symbol;Acc:MGI:1344341]	2.479012403	1.30976549	0.00056048	0.00557833	yes	up
ENSMUSG00000026380	Tfcp2l1	transcription factor CP2-like 1 [Source:MGI Symbol;Acc:MGI:2444691]	0.364081815	-1.457665413	0.00057783	0.00573126	yes	down
ENSMUSG00000026117	Zap70	zeta-chain (TCR) associated protein kinase [Source:MGI Symbol;Acc:MGI:99613]	2.108627972	1.076304581	0.00058348	0.00577403	yes	up
ENSMUSG00000005320	Fgfr4	fibroblast growth factor receptor 4 [Source:MGI	3.410828502	1.770122218	0.00058718	0.00580065	yes	up
ENSMUSG00000000320	Alox12	arachidonate 12-lipoxygenase [Source:MGI	0.369219959	-1.43744755	0.00058909	0.00581623	yes	down
ENSMUSG00000086390	1810019D21Rik	RIKEN cDNA 1810019D21 gene [Source:MGI Symbol;Acc:MGI:1917021]	0.482405299	-1.05168234	0.00059204	0.00583871	yes	down
ENSMUSG00000048416	Mlf1	myeloid leukemia factor 1 [Source:MGI	0.480351942	-1.057836276	0.00059314	0.00584622	yes	down
ENSMUSG00000027528	Fabp9	fatty acid binding protein 9, testis [Source:MGI Symbol;Acc:MGI:1194881]	0.038811098	-4.687386937	0.00059713	1	yes	down
ENSMUSG00000042808	Gpx2	glutathione peroxidase 2 [Source:MGI Symbol;Acc:MGI:106609]	0.429476555	-1.219348719	0.00060396	0.00593929	yes	down

ENSMUSG00000079465	Col4a3	collagen, type IV, alpha 3 [Source:MGI	2.888259697	1.530200467	0.00060434	0.00593966	yes	up
ENSMUSG00000037979	Ccdc92	coiled-coil domain containing 92 [Source:MGI Symbol;Acc:MGI:106485]	0.47338462	-1.078915262	0.00060933	0.00597851	yes	down
ENSMUSG00000042845	Wfdc12	WAP four-disulfide core domain 12 [Source:MGI Symbol;Acc:MGI:2183434]	2.683596979	1.424168025	0.00061026	0.0059832	yes	up
ENSMUSG00000034324	Tmem132c	transmembrane protein 132C [Source:MGI Symbol;Acc:MGI:2443061]	2.913274851	1.542641818	0.0006105	0.0059832	yes	up
ENSMUSG00000053024	Cntn2	contactin 2 [Source:MGI Symbol;Acc:MGI:104518]	0.091671441	-3.447383839	0.00061085	0.00598324	yes	down
ENSMUSG00000044749	Abca6	ATP-binding cassette, sub-family A (ABC1), member 6 [Source:MGI Symbol;Acc:MGI:1923434]	2.002207751	1.001591677	0.00061245	0.00599549	yes	up
ENSMUSG00000027761	Aadac	arylacetamide deacetylase [Source:MGI	2.528318392	1.338178154	0.00061788	0.00602472	yes	up
ENSMUSG00000028328	Tmod1	tropomodulin 1 [Source:MGI Symbol;Acc:MGI:98775]	2.160900362	1.111632552	0.00062135	0.00605178	yes	up
ENSMUSG00000029641	Rasl11a	RAS-like, family 11, member A [Source:MGI Symbol;Acc:MGI:1916145]	0.372244102	-1.425679104	0.00063526	0.00615947	yes	down
ENSMUSG00000079049	Serpinb1c	serine (or cysteine) peptidase inhibitor, clade B, member 1c [Source:MGI	6.48868343	2.697925782	0.00064527	0.00623909	yes	up
ENSMUSG00000025141	Myadml2	myeloid-associated differentiation marker-like 2 [Source:MGI Symbol;Acc:MGI:1915765]	2.16053761	1.111390345	0.00064823	0.00625414	yes	up
ENSMUSG00000062991	Nrg1	neuregulin 1 [Source:MGI Symbol;Acc:MGI:96083]	0.337568596	-1.5667474	0.00065281	0.00627685	yes	down
ENSMUSG00000091376	Aadacl2	arylacetamide deacetylase like 2 [Source:MGI Symbol;Acc:MGI:3646333]	2.044245458	1.031568435	0.00065632	0.00630007	yes	up
ENSMUSG00000005465	Il27ra	interleukin 27 receptor, alpha [Source:MGI Symbol;Acc:MGI:1355318]	2.150652706	1.104774573	0.00066385	0.00636174	yes	up
ENSMUSG00000037813	D630003M21Rik	RIKEN cDNA D630003M21 gene [Source:MGI Symbol;Acc:MGI:3606579]	3.118227063	1.640725986	0.00067151	0.00642097	yes	up
ENSMUSG00000046806	Cyren	cell cycle regulator of NHEJ [Source:MGI Symbol;Acc:MGI:1925662]	0.497834524	-1.006261811	0.00067646	0.00646116	yes	down
ENSMUSG00000069270	H2ac6	H2A clustered histone 6 [Source:MGI Symbol;Acc:MGI:2448287]	0.303603996	-1.719737316	0.00067725	0.00646509	yes	down
ENSMUSG00000028289	Epha7	Eph receptor A7 [Source:MGI Symbol;Acc:MGI:95276]	2.668892313	1.416241095	0.00068688	0.00651757	yes	up
ENSMUSG00000076555	Igkv4-57-1	immunoglobulin kappa variable 4-57-1 [Source:MGI Symbol;Acc:MGI:2686264]	3.783182117	1.919600227	0.00068878	0.00651757	yes	up
ENSMUSG00000044211	Gm7887	predicted gene 7887 [Source:MGI Symbol;Acc:MGI:3648251]	3.066337824	1.61651665	0.00068987	0.00652072	yes	up
ENSMUSG00000042010	Acacb	acetyl-Coenzyme A carboxylase beta [Source:MGI Symbol;Acc:MGI:2140940]	2.839025557	1.505395836	0.00069365	0.00653502	yes	up
ENSMUSG00000099269	Calm5	calmodulin 5 [Source:MGI Symbol;Acc:MGI:3511177]	2.524291132	1.335878309	0.00069598	0.00655346	yes	up
ENSMUSG00000073834	Mup11	major urinary protein 11 [Source:MGI Symbol;Acc:MGI:3709617]	2.837377378	1.504558045	0.00069704	0.00655648	yes	up
ENSMUSG00000037872	Ackr1	atypical chemokine receptor 1 (Duffy blood group) [Source:MGI Symbol;Acc:MGI:1097689]	2.091179781	1.064317097	0.00069751	0.00655648	yes	up
ENSMUSG00000043472	Lce3d	late cornified envelope 3D [Source:MGI	2.463051956	1.300447061	0.00069786	0.00655648	yes	up
ENSMUSG00000096074	Ighv1-72	immunoglobulin heavy variable 1-72 [Source:MGI Symbol;Acc:MGI:4439633]	2.252812886	1.171727491	0.00069801	0.00655648	yes	up
ENSMUSG00000032327	Stra6	stimulated by retinoic acid gene 6 [Source:MGI Symbol;Acc:MGI:107742]	0.128899292	-2.955683759	0.0006982	0.00655648	yes	down
ENSMUSG00000027380	Acoxl	acyl-Coenzyme A oxidase-like [Source:MGI Symbol;Acc:MGI:1921371]	3.389750688	1.761179169	0.00069919	0.00656223	yes	up
ENSMUSG00000038663	Fsd2	fibronectin type III and SPRY domain containing 2 [Source:MGI Symbol;Acc:MGI:2444310]	2.017694372	1.01270766	0.00070464	0.00660525	yes	up
ENSMUSG00000020241	Col6a2	collagen, type VI, alpha 2 [Source:MGI	2.543264205	1.346681343	0.00070492	0.00660525	yes	up
ENSMUSG00000094194	Ighv5-16	immunoglobulin heavy variable 5-16 [Source:MGI Symbol;Acc:MGI:4439556]	6.597188331	2.72185129	0.00071373	0.00666611	yes	up
ENSMUSG00000039883	Lrrc17	leucine rich repeat containing 17 [Source:MGI Symbol;Acc:MGI:1921761]	2.536538891	1.342861281	0.00071684	0.00668031	yes	up
ENSMUSG00000074433	Lce3e	late cornified envelope 3E [Source:MGI	2.622111515	1.390729043	0.00071718	0.00668031	yes	up

ENSMUSG00000040724	Kcna2	potassium voltage-gated channel, shaker-related subfamily, member 2 [Source:MGI	2.060746376	1.043166958	0.00072205	0.00671119	yes	up
ENSMUSG00000052133	Sema5b	sema domain, seven thrombospondin repeats (type 1 and type 1-like), transmembrane domain (TM) and short cytoplasmic domain, (semaphorin) 5B [Source:MGI Symbol;Acc:MGI:107555]	2.386327937	1.254792317	0.00072547	0.0067393	yes	up
ENSMUSG00000026442	Nfasc	neurofascin [Source:MGI Symbol;Acc:MGI:104753]	2.720368542	1.443802114	0.00073136	0.00679044	yes	up
ENSMUSG00000021751	Acox2	acyl-Coenzyme A oxidase 2, branched chain [Source:MGI Symbol;Acc:MGI:1934852]	2.04728839	1.033714341	0.00073269	0.00679542	yes	up
ENSMUSG00000060402	Chst8	carbohydrate sulfotransferase 8 [Source:MGI Symbol;Acc:MGI:1916197]	2.976308868	1.573524251	0.00074183	0.00686078	yes	up
ENSMUSG00000068888	Lce1i	late cornified envelope 1I [Source:MGI	2.308513187	1.206963974	0.00076723	0.0070403	yes	up
ENSMUSG00000020926	Adam11	a disintegrin and metallopeptidase domain 11 [Source:MGI Symbol;Acc:MGI:1098667]	2.176597089	1.122074374	0.00077036	0.00705777	yes	up
ENSMUSG00000050520	Cldn8	claudin 8 [Source:MGI Symbol;Acc:MGI:1859286]	0.382085122	-1.388034014	0.0007773	0.00711004	yes	down
ENSMUSG00000034997	Htr2a	5-hydroxytryptamine (serotonin) receptor 2A [Source:MGI Symbol;Acc:MGI:109521]	2.160275462	1.111215285	0.00078665	0.0071804	yes	up
ENSMUSG00000001119	Col6a1	collagen, type VI, alpha 1 [Source:MGI	2.47307071	1.30630349	0.00079177	0.00720943	yes	up
ENSMUSG00000112972	Gm48417	predicted gene, 48417 [Source:MGI Symbol;Acc:MGI:6097910]	0.365880756	-1.450554559	0.00082373	0.00744028	yes	down
ENSMUSG00000113459	Gm30655	predicted gene, 30655 [Source:MGI Symbol;Acc:MGI:5589814]	0.113684705	-3.136889923	0.00082752	0.0074589	yes	down
ENSMUSG00000049001	Ndnf	neuron-derived neurotrophic factor [Source:MGI Symbol;Acc:MGI:1915419]	2.09351309	1.065925939	0.00084443	0.00756006	yes	up
ENSMUSG00000031870	Pgr	progesterone receptor [Source:MGI Symbol;Acc:MGI:97567]	43.65768198	5.448163627	0.00085292	1	yes	up
ENSMUSG00000053228	Ceacam3	carcinoembryonic antigen-related cell adhesion molecule 3 [Source:MGI Symbol;Acc:MGI:3646296]	5.079817811	2.344776755	0.00085345	0.00762506	yes	up
ENSMUSG00000059956	Serpinb12	serine (or cysteine) peptidase inhibitor, clade B (ovalbumin), member 12 [Source:MGI Symbol;Acc:MGI:1919119]	2.874713908	1.523418386	0.00085659	0.00764819	yes	up
ENSMUSG00000013367	Iglon5	IgLON family member 5 [Source:MGI	2.623661386	1.391581536	0.00085737	0.00764819	yes	up
ENSMUSG00000010601	Apol7a	apolipoprotein L 7a [Source:MGI Symbol;Acc:MGI:1923011]	14.16910216	3.824676439	0.00086101	1	yes	up
ENSMUSG00000074109	Mrgprx2	MAS-related GPR, member X2 [Source:MGI Symbol;Acc:MGI:3588270]	2.158407772	1.109967449	0.00087462	0.00777127	yes	up
ENSMUSG00000057609	Lce1a1	late cornified envelope 1A1 [Source:MGI Symbol;Acc:MGI:1914377]	2.037913433	1.02709277	0.00087746	0.00779123	yes	up
ENSMUSG00000041261	Car8	carbonic anhydrase 8 [Source:MGI Symbol;Acc:MGI:88253]	3.236113783	1.694262334	0.00088385	0.00782387	yes	up
ENSMUSG00000059824	Dbp	D site albumin promoter binding protein [Source:MGI	2.639030415	1.400007978	0.00088544	0.00782987	yes	up
ENSMUSG00000028782	Adgrb2	adhesion G protein-coupled receptor B2 [Source:MGI Symbol;Acc:MGI:2451244]	2.401945774	1.264203581	0.00090876	0.00800338	yes	up
ENSMUSG00000093598	A730085K08 Rik	RIKEN cDNA A730085K08 gene [Source:MGI Symbol;Acc:MGI:3605633]	3.190734191	1.673888428	0.00091958	0.00807713	yes	up
ENSMUSG00000082016	Pgam1-ps2	phosphoglycerate mutase 1, pseudogene 2 [Source:MGI Symbol;Acc:MGI:3645709]	2.212007269	1.145356126	0.00091994	0.00807713	yes	up
ENSMUSG00000000386	Mx1	MX dynamin-like GTPase 1 [Source:MGI	2.238081517	1.162262584	0.00093516	0.00818509	yes	up
ENSMUSG00000050069	Grem2	gremlin 2, DAN family BMP antagonist [Source:MGI Symbol;Acc:MGI:1344367]	2.473035418	1.306282902	0.00093743	0.0081914	yes	up
ENSMUSG00000086233	Gm11816	predicted gene 11816 [Source:MGI Symbol;Acc:MGI:3650294]	2.126957633	1.088791297	0.00093966	0.00820034	yes	up
ENSMUSG00000021087	Rtn1	reticulon 1 [Source:MGI Symbol;Acc:MGI:1933947]	2.515140346	1.330638905	0.00094755	0.00824421	yes	up
ENSMUSG00000087614	Gm12514	predicted gene 12514 [Source:MGI Symbol;Acc:MGI:3651364]	10.02960821	3.326193345	0.00094996	1	yes	up
ENSMUSG00000087291	Gm11946	predicted gene 11946 [Source:MGI Symbol;Acc:MGI:3650071]	0.16767428	-2.576266684	0.00095293	0.00827326	yes	down
ENSMUSG00000059230	Defb4	defensin beta 4 [Source:MGI Symbol;Acc:MGI:1927667]	0.273853474	-1.868523915	0.00095328	0.00827326	yes	down
ENSMUSG00000092200	Tnxa	tenascin XA (pseudogene) [Source:MGI Symbol;Acc:MGI:2148489]	2.92361237	1.547752043	0.0009548	0.00828118	yes	up

ENSMUSG00000020067	Mypn	myopalladin [Source:MGI Symbol;Acc:MGI:1916052]	2.176209447	1.121817414	0.00095876	0.00830422	yes	up
ENSMUSG00000037709	Fam13a	family with sequence similarity 13, member A [Source:MGI Symbol;Acc:MGI:1889842]	2.883422129	1.527782061	0.00096515	0.00835109	yes	up
ENSMUSG00000098021	Gm9522	predicted gene 9522 [Source:MGI Symbol;Acc:MGI:3779931]	0.120764531	-3.049731305	0.0009744	0.00841007	yes	down
ENSMUSG00000073555	Gm4951	predicted gene 4951 [Source:MGI Symbol;Acc:MGI:3644953]	2.585663651	1.370534618	0.00098339	0.00846232	yes	up
ENSMUSG00000039601	Rcan2	regulator of calcineurin 2 [Source:MGI	2.251975918	1.1711914	0.00098865	0.00849706	yes	up
ENSMUSG00000099009	Rdh16f1	RDH16 family member 1 [Source:MGI	2.142764695	1.099473431	0.00098923	0.00849706	yes	up
ENSMUSG00000043903	Zfp469	zinc finger protein 469 [Source:MGI Symbol;Acc:MGI:2684868]	2.325610189	1.217609298	0.00099265	0.00852084	yes	up
ENSMUSG00000032368	Zic1	zinc finger protein of the cerebellum 1 [Source:MGI Symbol;Acc:MGI:106683]	2.545746425	1.348088723	0.00099675	0.00854331	yes	up
ENSMUSG00000095200	Ighv1-7	immunoglobulin heavy variable V1-7 [Source:MGI Symbol;Acc:MGI:3704122]	12.54624949	3.649184252	0.00101091	0.00863744	yes	up
ENSMUSG00000105003	Gm40055	predicted gene, 40055 [Source:MGI Symbol;Acc:MGI:5622940]	0.409514269	-1.288014374	0.00101123	0.00863744	yes	down
ENSMUSG00000079173	Zan	zonadhesin [Source:MGI Symbol;Acc:MGI:106656]	0.382864651	-1.385093628	0.00101605	0.00866578	yes	down
ENSMUSG00000106511	Gm43521	predicted gene 43521 [Source:MGI Symbol;Acc:MGI:5663658]	0.013042507	-6.260634958	0.00101606	1	yes	down
ENSMUSG00000091938	Gm2564	predicted gene 2564 [Source:MGI Symbol;Acc:MGI:3708691]	2.38919641	1.256525459	0.00102004	0.00869547	yes	up
ENSMUSG00000031494	Cd209a	CD209a antigen [Source:MGI Symbol;Acc:MGI:2157942]	2.013648946	1.00981219	0.00102501	0.00872928	yes	up
ENSMUSG00000020251	Glt8d2	glycosyltransferase 8 domain containing 2 [Source:MGI Symbol;Acc:MGI:1922032]	2.553054078	1.352224097	0.00103188	0.00877043	yes	up
ENSMUSG00000029134	Plb1	phospholipase B1 [Source:MGI Symbol;Acc:MGI:1922406]	0.386612927	-1.371038216	0.00103325	0.00877432	yes	down
ENSMUSG00000086321	Gm11413	predicted gene 11413 [Source:MGI Symbol;Acc:MGI:3650701]	0.280875969	-1.831994896	0.00104058	0.00882696	yes	down
ENSMUSG00000031962	Cdh15	cadherin 15 [Source:MGI Symbol;Acc:MGI:106672]	3.75693859	1.909557534	0.00104277	0.00883266	yes	up
ENSMUSG00000024331	Dsc2	desmocollin 2 [Source:MGI Symbol;Acc:MGI:103221]	0.442104575	-1.177540433	0.0010543	0.00890848	yes	down
ENSMUSG00000048503	Tlcd5	TLC domain containing 5 [Source:MGI	2.301097881	1.202322353	0.00106258	0.00896962	yes	up
ENSMUSG00000037568	Vash2	vasohibin 2 [Source:MGI Symbol;Acc:MGI:2444826]	2.558082849	1.35506299	0.00106575	0.00899204	yes	up
ENSMUSG00000038204	Asb10	ankyrin repeat and SOCS box-containing 10 [Source:MGI Symbol;Acc:MGI:2152836]	2.564798262	1.358845353	0.00106839	0.0090099	yes	up
ENSMUSG00000027923	Lce1b	late cornified envelope 1B [Source:MGI	2.163357239	1.11327192	0.00108024	0.00909208	yes	up
ENSMUSG00000031831	Dnaaf1	dynein, axonemal assembly factor 1 [Source:MGI Symbol;Acc:MGI:1915520]	0.230727029	-2.115741076	0.00109245	0.00916807	yes	down
ENSMUSG00000107379	Gm43126	predicted gene 43126 [Source:MGI Symbol;Acc:MGI:5663263]	0.2210454	-2.177585382	0.00110433	0.00925428	yes	down
ENSMUSG00000078507	Aadacl3	arylacetamide deacetylase like 3 [Source:MGI Symbol;Acc:MGI:2685281]	2.578982459	1.366801961	0.00110648	0.00926339	yes	up
ENSMUSG00000082815	Gm5678	predicted gene 5678 [Source:MGI Symbol;Acc:MGI:3646098]	7.265693795	2.861100566	0.0011112	0.00929832	yes	up
ENSMUSG00000070323	Mmp27	matrix metallopeptidase 27 [Source:MGI Symbol;Acc:MGI:3039232]	2.256098908	1.173830317	0.00111488	0.00931687	yes	up
ENSMUSG00000090338	Gm17081	predicted gene 17081 [Source:MGI Symbol;Acc:MGI:4937908]	3.383248701	1.758409233	0.00111953	0.00934382	yes	up
ENSMUSG00000056880	Gadl1	glutamate decarboxylase-like 1 [Source:MGI Symbol;Acc:MGI:1920998]	5.311941478	2.409239252	0.00111987	0.00934382	yes	up
ENSMUSG00000039699	Batf2	basic leucine zipper transcription factor, ATF-like 2 [Source:MGI Symbol;Acc:MGI:1921731]	2.332856952	1.222097846	0.00112128	0.009351	yes	up
ENSMUSG00000034656	Cacna1a	calcium channel, voltage-dependent, P/Q type, alpha 1A subunit [Source:MGI Symbol;Acc:MGI:109482]	2.064044422	1.04547402	0.00113156	0.00941859	yes	up
ENSMUSG00000054863	Tafa5	TAFA chemokine like family member 5 [Source:MGI Symbol;Acc:MGI:2146182]	0.483469841	-1.048502199	0.00114268	0.00948347	yes	down
ENSMUSG00000004267	Eno2	enolase 2, gamma neuronal [Source:MGI	2.351972994	1.233871495	0.00114279	0.00948347	yes	up
ENSMUSG00000030708	Dnajb13	DnaJ heat shock protein family (Hsp40) member B13 [Source:MGI Symbol;Acc:MGI:1916637]	0.427982918	-1.22437488	0.00114489	0.00948841	yes	down

ENSMUSG00000002475	Abhd3	abhydrolase domain containing 3 [Source:MGI Symbol;Acc:MGI:2147183]	2.259598631	1.176066532	0.00115631	0.00956933	yes	up
ENSMUSG00000096410	Ighv1-19	immunoglobulin heavy variable V1-19 [Source:MGI Symbol;Acc:MGI:4439779]	2.16658008	1.115419562	0.00116039	0.00958894	yes	up
ENSMUSG00000103243	Lce1d	late cornified envelope 1D [Source:MGI	2.551846908	1.351541781	0.0011622	0.00959511	yes	up
ENSMUSG00000055632	Hmcn2	hemicentin 2 [Source:MGI Symbol;Acc:MGI:2677838]	5.259450081	2.394911962	0.00116896	0.00963706	yes	up
ENSMUSG00000042092	Lce1c	late cornified envelope 1C [Source:MGI	2.286082871	1.192877703	0.00117468	0.00967037	yes	up
ENSMUSG00000046318	Ccbe1	collagen and calcium binding EGF domains 1 [Source:MGI Symbol;Acc:MGI:2445053]	2.12325512	1.086277729	0.00119518	0.00980181	yes	up
ENSMUSG00000087090	Nctc1	non-coding transcript 1 [Source:MGI Symbol;Acc:MGI:1306816]	2.361297705	1.239579944	0.00121189	0.00990799	yes	up
ENSMUSG00000046480	Scn4b	sodium channel, type IV, beta [Source:MGI Symbol;Acc:MGI:2687406]	2.015662395	1.011254021	0.00121214	0.00990799	yes	up
ENSMUSG00000103037	Pcdhgb1	protocadherin gamma subfamily B, 1 [Source:MGI Symbol;Acc:MGI:1935169]	2.163800472	1.113567472	0.00122246	0.00998293	yes	up
ENSMUSG00000027419	Pcsk2	proprotein convertase subtilisin/kexin type 2 [Source:MGI Symbol;Acc:MGI:97512]	0.178026274	-2.489837921	0.00122382	0.00998931	yes	down
ENSMUSG00000031997	Trpc6	transient receptor potential cation channel, subfamily C, member 6 [Source:MGI Symbol;Acc:MGI:109523]	2.301674698	1.202683948	0.00122517	0.00999228	yes	up
ENSMUSG00000049593	Lce1h	late cornified envelope 1H [Source:MGI	2.447790395	1.291480025	0.00122534	0.00999228	yes	up
ENSMUSG00000036560	Lgi4	leucine-rich repeat LGI family, member 4 [Source:MGI Symbol;Acc:MGI:2180197]	2.022122632	1.015870492	0.001229	0.0100174	yes	up
ENSMUSG00000052105	Mtcl1	microtubule crosslinking factor 1 [Source:MGI Symbol;Acc:MGI:1915867]	0.444681561	-1.16915551	0.00124853	0.01015267	yes	down
ENSMUSG00000054200	Ffar4	free fatty acid receptor 4 [Source:MGI Symbol;Acc:MGI:2147577]	2.723388265	1.445402678	0.00126259	0.01025151	yes	up
ENSMUSG00000046182	Gsg1l	GSG1-like [Source:MGI Symbol;Acc:MGI:2685483]	3.488830238	1.8027434	0.00127964	0.01034727	yes	up
ENSMUSG00000045555	Mettl24	methyltransferase like 24 [Source:MGI	2.054684792	1.038917088	0.00129588	0.01044439	yes	up
ENSMUSG00000058966	Tlcd3b	TLC domain containing 3B [Source:MGI Symbol;Acc:MGI:1916202]	3.162573507	1.661099013	0.00131779	0.0105668	yes	up
ENSMUSG00000010086	Rnf112	ring finger protein 112 [Source:MGI Symbol;Acc:MGI:106611]	4.885355332	2.288463499	0.00133008	0.01064558	yes	up
ENSMUSG00000082691	Dynlt1-ps1	dynein light chain Tctex-type 1, pseuodogene 1 [Source:MGI Symbol;Acc:MGI:3642625]	4.54455294	2.18413838	0.00133143	0.01065142	yes	up
ENSMUSG00000020787	P2rx1	purinergic receptor P2X, ligand-gated ion channel, 1 [Source:MGI Symbol;Acc:MGI:1098235]	2.190751983	1.131426165	0.00134279	0.01072447	yes	up
ENSMUSG00000063415	Cyp26b1	cytochrome P450, family 26, subfamily b, polypeptide 1 [Source:MGI	2.030178776	1.021606775	0.00135301	0.01078911	yes	up
ENSMUSG00000025129	Ppp1r27	protein phosphatase 1, regulatory subunit 27 [Source:MGI Symbol;Acc:MGI:1915951]	2.88526776	1.528705211	0.00136274	0.01085674	yes	up
ENSMUSG00000048388	Fam171b	family with sequence similarity 171, member B [Source:MGI Symbol;Acc:MGI:2444579]	2.16702807	1.115717841	0.00138273	0.01099066	yes	up
ENSMUSG00000104701	Gm42555	predicted gene 42555 [Source:MGI Symbol;Acc:MGI:5662692]	0.127239501	-2.97438147	0.00138745	0.01101805	yes	down
ENSMUSG00000060275	Nrg2	neuregulin 2 [Source:MGI Symbol;Acc:MGI:1098246]	0.42947733	-1.219346115	0.00144175	0.0113865	yes	down
ENSMUSG00000118070	Gm50209	predicted gene, 50209 [Source:MGI Symbol;Acc:MGI:6302995]	0.048454006	-4.367240234	0.00145022	1	yes	down
ENSMUSG00000021575	Ahrr	aryl-hydrocarbon receptor repressor [Source:MGI Symbol;Acc:MGI:1333776]	2.841330591	1.5065667	0.00146022	0.01150079	yes	up
ENSMUSG00000031561	Tenm3	teneurin transmembrane protein 3 [Source:MGI Symbol;Acc:MGI:1345183]	2.32936243	1.219935129	0.00146992	0.01156139	yes	up
ENSMUSG00000031872	Bean1	brain expressed, associated with Nedd4, 1 [Source:MGI Symbol;Acc:MGI:1929597]	0.429077081	-1.220691252	0.00149241	0.01171168	yes	down
ENSMUSG00000076940	Iglv2	immunoglobulin lambda variable 2 [Source:MGI	16.05855052	4.005269773	0.00149635	0.01173592	yes	up
ENSMUSG00000042254	Cilp	cartilage intermediate layer protein, nucleotide pyrophosphohydrolase [Source:MGI Symbol;Acc:MGI:2444507]	2.43269811	1.282557298	0.00149774	0.01173592	yes	up

ENSMUSG00000029019	Nppb	natriuretic peptide type B [Source:MGI	0.336479999	-1.571407345	0.00150074	0.01175035	yes	down
ENSMUSG00000001656	Hoxc11	homeobox C11 [Source:MGI Symbol;Acc:MGI:96193]	0.023998298	-5.380924107	0.00151597	1	yes	down
ENSMUSG00000040537	Adam22	a disintegrin and metallopeptidase domain 22 [Source:MGI Symbol;Acc:MGI:1340046]	2.511602305	1.328608041	0.00151841	0.0118726	yes	up
ENSMUSG00000021614	Vcan	versican [Source:MGI Symbol;Acc:MGI:102889]	2.075424104	1.053406175	0.00152057	0.01188409	yes	up
ENSMUSG00000002007	Srpk3	serine/arginine-rich protein specific kinase 3 [Source:MGI Symbol;Acc:MGI:1891338]	3.04053329	1.604324385	0.0015507	0.01209772	yes	up
ENSMUSG00000027577	Chrna4	cholinergic receptor, nicotinic, alpha polypeptide 4 [Source:MGI Symbol;Acc:MGI:87888]	0.271156244	-1.8828037	0.00155171	0.01210008	yes	down
ENSMUSG00000031553	Adam3	a disintegrin and metallopeptidase domain 3 (cyritestin) [Source:MGI Symbol;Acc:MGI:102518]	4.635417962	2.212699428	0.00156122	0.01215235	yes	up
ENSMUSG00000086714	0610009E02Rik	RIKEN cDNA 0610009E02 gene [Source:MGI Symbol;Acc:MGI:3698435]	0.474410495	-1.07579217	0.00156704	0.01218671	yes	down
ENSMUSG00000022863	Btg3	BTG anti-proliferation factor 3 [Source:MGI Symbol;Acc:MGI:109532]	0.498184304	-1.005248527	0.00158466	0.01230516	yes	down
ENSMUSG00000069171	Nr2f1	nuclear receptor subfamily 2, group F, member 1 [Source:MGI Symbol;Acc:MGI:1352451]	2.346063152	1.230241849	0.0015883	0.0123243	yes	up
ENSMUSG00000031382	Asb11	ankyrin repeat and SOCS box-containing 11 [Source:MGI Symbol;Acc:MGI:1916104]	2.756839326	1.463015187	0.00161223	0.01248196	yes	up
ENSMUSG00000009216	Fam163b	family with sequence similarity 163, member B [Source:MGI Symbol;Acc:MGI:1926106]	6.805923424	2.766790919	0.00161674	0.01250571	yes	up
ENSMUSG00000004814	Ccl24	chemokine (C-C motif) ligand 24 [Source:MGI Symbol;Acc:MGI:1928953]	3.924051426	1.972343949	0.0016222	0.01251429	yes	up
ENSMUSG00000060913	Trim55	tripartite motif-containing 55 [Source:MGI Symbol;Acc:MGI:3036269]	3.217461968	1.685923095	0.00163875	0.01260341	yes	up
ENSMUSG00000030351	Tspan11	tetraspanin 11 [Source:MGI Symbol;Acc:MGI:1915748]	2.035646788	1.025487256	0.00164185	0.01261136	yes	up
ENSMUSG00000032315	Cyp1a1	cytochrome P450, family 1, subfamily a, polypeptide 1 [Source:MGI Symbol;Acc:MGI:88588]	2.314031948	1.210408782	0.00166367	0.01274325	yes	up
ENSMUSG00000049382	Krt8	keratin 8 [Source:MGI Symbol;Acc:MGI:96705]	0.37059741	-1.432075298	0.00169578	0.01295478	yes	down
ENSMUSG00000030911	Zp2	zona pellucida glycoprotein 2 [Source:MGI	0.11548321	-3.114244977	0.00171598	0.0130804	yes	down
ENSMUSG00000008658	Rbfox1	RNA binding protein, fox-1 homolog (C. elegans) 1 [Source:MGI Symbol;Acc:MGI:1926224]	2.285051663	1.192226784	0.00174691	0.01328083	yes	up
ENSMUSG00000047642	D930020B18Rik	RIKEN cDNA D930020B18 gene [Source:MGI Symbol;Acc:MGI:2442001]	0.222232267	-2.169859788	0.00177085	0.01345097	yes	down
ENSMUSG00000079343	C1s2	complement component 1, s subcomponent 2 [Source:MGI Symbol;Acc:MGI:3644269]	2.865370795	1.518721844	0.00177407	0.01346953	yes	up
ENSMUSG00000028003	Lrat	lecithin-retinol acyltransferase (phosphatidylcholine-retinol-O-acyltransferase) [Source:MGI Symbol;Acc:MGI:1891259]	0.421384324	-1.24679145	0.00177785	0.01348638	yes	down
ENSMUSG00000061080	Lsamp	limbic system-associated membrane protein [Source:MGI Symbol;Acc:MGI:1261760]	3.172539567	1.665638157	0.00178561	0.01352153	yes	up
ENSMUSG00000063232	Serpina11	serine (or cysteine) peptidase inhibitor, clade A (alpha-1 antiproteinase, antitrypsin), member 11 [Source:MGI	0.256554917	-1.962660419	0.00180048	0.01359246	yes	down
ENSMUSG00000059921	Unc5c	unc-5 netrin receptor C [Source:MGI Symbol;Acc:MGI:1095412]	2.8271833	1.499365423	0.00180359	0.01360997	yes	up
ENSMUSG00000047420	Fam180a	family with sequence similarity 180, member A [Source:MGI Symbol;Acc:MGI:3039626]	3.636694844	1.862627874	0.00182601	0.01372777	yes	up
ENSMUSG00000043456	Zfp536	zinc finger protein 536 [Source:MGI Symbol;Acc:MGI:1926102]	2.89045031	1.531294271	0.00183227	0.01376627	yes	up
ENSMUSG00000038754	Elovl3	elongation of very long chain fatty acids (FEN1/Elo2, SUR4/Elo3, yeast)-like 3 [Source:MGI Symbol;Acc:MGI:1195976]	2.482683258	1.311900213	0.00188096	0.01407114	yes	up
ENSMUSG00000046834	Krt1	keratin 1 [Source:MGI Symbol;Acc:MGI:96698]	2.603002668	1.380176791	0.00188098	0.01407114	yes	up
ENSMUSG00000028654	Mycl	v-myc avian myelocytomatosis viral oncogene lung carcinoma derived [Source:MGI	0.456997776	-1.12974095	0.00189546	0.01414274	yes	down
ENSMUSG00000043366	Olfr78	olfactory receptor 78 [Source:MGI Symbol;Acc:MGI:2157548]	2.350273259	1.232828504	0.00191223	0.01425281	yes	up

ENSMUSG00000036832	Lpar3	lysophosphatidic acid receptor 3 [Source:MGI Symbol;Acc:MGI:1929469]	0.459781347	-1.120980158	0.00192605	0.0143462	yes	down
ENSMUSG00000067158	Col4a4	collagen, type IV, alpha 4 [Source:MGI	2.044633078	1.031841966	0.00192793	0.01435403	yes	up
ENSMUSG00000037440	Vnn1	vanin 1 [Source:MGI Symbol;Acc:MGI:108395]	0.487799864	-1.035638738	0.00193114	0.01435567	yes	down
ENSMUSG00000027070	Lrp2	low density lipoprotein receptor-related protein 2 [Source:MGI Symbol;Acc:MGI:95794]	0.128263011	-2.962822911	0.0019323	0.01435567	yes	down
ENSMUSG00000046743	Fat4	FAT atypical cadherin 4 [Source:MGI Symbol;Acc:MGI:3045256]	2.535723066	1.342397193	0.00193626	0.01437274	yes	up
ENSMUSG00000026548	Slamf9	SLAM family member 9 [Source:MGI Symbol;Acc:MGI:1923692]	2.271907006	1.183903784	0.00194316	0.01441262	yes	up
ENSMUSG00000115678	Gm49212	predicted gene, 49212 [Source:MGI Symbol;Acc:MGI:6118665]	0.220113666	-2.183679376	0.0019433	0.01441262	yes	down
ENSMUSG00000089728	Clec2f	C-type lectin domain family 2, member f [Source:MGI Symbol;Acc:MGI:3522133]	2.204208697	1.140260827	0.00194688	0.01443297	yes	up
ENSMUSG00000032908	Sgpp2	sphingosine-1-phosphate phosphatase 2 [Source:MGI Symbol;Acc:MGI:3589109]	2.219138065	1.149999429	0.00194927	0.01444447	yes	up
ENSMUSG00000025892	Gria4	glutamate receptor, ionotropic, AMPA4 (alpha 4) [Source:MGI Symbol;Acc:MGI:95811]	3.911052472	1.967556892	0.00196701	0.01455099	yes	up
ENSMUSG00000112965	Gm34278	predicted gene, 34278 [Source:MGI Symbol;Acc:MGI:5593437]	0.145964506	-2.776310501	0.00198024	0.01460513	yes	down
ENSMUSG00000100980	Gm29100	predicted gene 29100 [Source:MGI Symbol;Acc:MGI:5579806]	4.539290214	2.182466728	0.00198156	0.01460861	yes	up
ENSMUSG00000034783	Cd207	CD207 antigen [Source:MGI Symbol;Acc:MGI:2180021]	3.739965584	1.903024994	0.00199597	0.01470232	yes	up
ENSMUSG00000094930	Igkv6-25	immunoglobulin kappa chain variable 6-25 [Source:MGI Symbol;Acc:MGI:4439867]	4.192793674	2.067911837	0.00200284	0.01474032	yes	up
ENSMUSG00000054555	Adam12	a disintegrin and metallopeptidase domain 12 (meltrin alpha) [Source:MGI	2.308045944	1.206671943	0.00202359	0.01485988	yes	up
ENSMUSG00000006675	P4htm	prolyl 4-hydroxylase, transmembrane (endoplasmic reticulum) [Source:MGI Symbol;Acc:MGI:1921693]	2.066582658	1.047247069	0.00204562	0.01498503	yes	up
ENSMUSG00000050663	Trhde	TRH-degrading enzyme [Source:MGI Symbol;Acc:MGI:2384311]	2.048634611	1.034662692	0.00206617	0.01507861	yes	up
ENSMUSG00000031673	Cdh11	cadherin 11 [Source:MGI Symbol;Acc:MGI:99217]	2.0079411	1.00571695	0.00208865	0.01521003	yes	up
ENSMUSG00000105245	Gm31305	predicted gene, 31305 [Source:MGI Symbol;Acc:MGI:5590464]	3.104708547	1.634457842	0.00209625	0.01524612	yes	up
ENSMUSG00000031098	Syt8	synaptotagmin VIII [Source:MGI Symbol;Acc:MGI:1859867]	0.468058419	-1.095239489	0.00212294	0.01540779	yes	down
ENSMUSG00000016283	H2-M2	histocompatibility 2, M region locus 2 [Source:MGI	3.135398615	1.64864887	0.00213301	0.01546793	yes	up
ENSMUSG00000018341	Il12rb2	interleukin 12 receptor, beta 2 [Source:MGI Symbol;Acc:MGI:1270861]	3.215304349	1.684955304	0.0021635	0.01565615	yes	up
ENSMUSG00000032845	Alpk2	alpha-kinase 2 [Source:MGI Symbol;Acc:MGI:2449492]	2.098351454	1.069256336	0.00216712	0.01566921	yes	up
ENSMUSG00000029348	Asphd2	aspartate beta-hydroxylase domain containing 2 [Source:MGI Symbol;Acc:MGI:1920148]	2.669042245	1.41632214	0.00216995	0.01568318	yes	up
ENSMUSG00000018620	Mmp20	matrix metallopeptidase 20 (enamelysin) [Source:MGI Symbol;Acc:MGI:1353466]	0.033597663	-4.895495306	0.00217783	1	yes	down
ENSMUSG00000035799	Twist1	twist basic helix-loop-helix transcription factor 1 [Source:MGI Symbol;Acc:MGI:98872]	2.26987346	1.182611873	0.00218362	0.01576749	yes	up
ENSMUSG00000002033	Cd3g	CD3 antigen, gamma polypeptide [Source:MGI	2.020506469	1.014716971	0.00219186	0.01581503	yes	up
ENSMUSG00000027737	Slc7a11	solute carrier family 7 (cationic amino acid transporter, y+ system), member 11 [Source:MGI Symbol;Acc:MGI:1347355]	0.394224404	-1.342911009	0.0021963	0.01582378	yes	down
ENSMUSG00000064294	Aox3	aldehyde oxidase 3 [Source:MGI Symbol;Acc:MGI:1918974]	2.13868999	1.096727374	0.00220561	0.01588113	yes	up
ENSMUSG00000109127	Gm31135	predicted gene, 31135 [Source:MGI Symbol;Acc:MGI:5590294]	0.481775905	-1.053565853	0.00224017	0.01604311	yes	down
ENSMUSG00000043298	Smco3	single-pass membrane protein with coiled-coil domains 3 [Source:MGI Symbol;Acc:MGI:2443451]	2.764808647	1.467179635	0.0022427	0.01605457	yes	up
ENSMUSG00000038085	Cnbd2	cyclic nucleotide binding domain containing 2 [Source:MGI Symbol;Acc:MGI:1918123]	2.688315668	1.426702552	0.00224452	0.01606097	yes	up
ENSMUSG00000035041	Creb3l3	cAMP responsive element binding protein 3-like 3 [Source:MGI Symbol;Acc:MGI:2384786]	2.016886247	1.012129718	0.00224677	0.01607038	yes	up
ENSMUSG00000115925	Gm33432	predicted gene, 33432 [Source:MGI Symbol;Acc:MGI:5592591]	3.023160747	1.596057692	0.00225246	0.01609778	yes	up

ENSMUSG00000010021	Kif19a	kinesin family member 19A [Source:MGI Symbol;Acc:MGI:2447024]	2.257993589	1.17504139	0.0022623	0.01616144	yes	up
ENSMUSG00000031343	Gabra3	gamma-aminobutyric acid (GABA) A receptor, subunit alpha 3 [Source:MGI	2.114595227	1.080381531	0.00226893	0.01620205	yes	up
ENSMUSG00000034459	Ifit1	interferon-induced protein with tetratricopeptide repeats 1 [Source:MGI	2.328876297	1.21963401	0.0022779	0.0162594	yes	up
ENSMUSG00000038242	Aox4	aldehyde oxidase 4 [Source:MGI Symbol;Acc:MGI:1919122]	2.063117925	1.044826286	0.00228628	0.01629907	yes	up
ENSMUSG00000068890	Lce1a2	late cornified envelope 1A2 [Source:MGI Symbol;Acc:MGI:1920972]	2.011197174	1.008054528	0.00230387	0.01639314	yes	up
ENSMUSG00000028262	Clca3a2	chloride channel accessory 3A2 [Source:MGI Symbol;Acc:MGI:1931471]	2.235315484	1.160478462	0.00230422	0.01639314	yes	up
ENSMUSG00000038077	Kcna6	potassium voltage-gated channel, shaker-related, subfamily, member 6 [Source:MGI	2.477607791	1.308947825	0.00235418	0.0166663	yes	up
ENSMUSG00000021624	Cd180	CD180 antigen [Source:MGI Symbol;Acc:MGI:1194924]	2.012333735	1.008869588	0.00235697	0.01667919	yes	up
ENSMUSG00000028457	Atp8b5	ATPase, class I, type 8B, member 5 [Source:MGI Symbol;Acc:MGI:2444287]	2.13536027	1.094479497	0.0023696	0.01675078	yes	up
ENSMUSG00000021708	Rasgrf2	RAS protein-specific guanine nucleotide-releasing factor 2 [Source:MGI Symbol;Acc:MGI:109137]	2.032814271	1.023478409	0.00237513	0.01677337	yes	up
ENSMUSG00000085888	Gm12224	predicted gene 12224 [Source:MGI Symbol;Acc:MGI:3651369]	3.230307906	1.691671686	0.00238715	0.01684452	yes	up
ENSMUSG00000045744	Bricd5	BRICHOS domain containing 5 [Source:MGI Symbol;Acc:MGI:2441766]	0.119526092	-3.064602504	0.00243128	1	yes	down
ENSMUSG00000069763	Tmem100	transmembrane protein 100 [Source:MGI Symbol;Acc:MGI:1915138]	2.288713637	1.194536965	0.00247432	0.01734635	yes	up
ENSMUSG00000056380	Gpr50	G-protein-coupled receptor 50 [Source:MGI Symbol;Acc:MGI:1333877]	0.02572751	-5.280544329	0.0024956	1	yes	down
ENSMUSG00000021260	Hhipl1	hedgehog interacting protein-like 1 [Source:MGI Symbol;Acc:MGI:1919265]	2.497088654	1.320247042	0.00249955	0.01746427	yes	up
ENSMUSG00000086298	Gm11716	predicted gene 11716 [Source:MGI Symbol;Acc:MGI:3649215]	2.204164794	1.140232091	0.00254757	0.017759	yes	up
ENSMUSG00000050447	Lypd6	LY6/PLAUR domain containing 6 [Source:MGI Symbol;Acc:MGI:2443848]	0.440348812	-1.183281321	0.00259659	0.01800775	yes	down
ENSMUSG00000046668	Cxxc5	CXXC finger 5 [Source:MGI Symbol;Acc:MGI:1914643]	2.289175111	1.194827826	0.00259663	0.01800775	yes	up
ENSMUSG00000115754	Gm48972	predicted gene, 48972 [Source:MGI Symbol;Acc:MGI:6118313]	3.793437064	1.923505602	0.00261123	0.01807884	yes	up
ENSMUSG00000066060	Gm12866	predicted gene 12866 [Source:MGI Symbol;Acc:MGI:3649462]	3.177060147	1.667692403	0.00269498	0.01858432	yes	up
ENSMUSG00000059089	Fcgr4	Fc receptor, IgG, low affinity IV [Source:MGI Symbol;Acc:MGI:2179523]	2.194313723	1.133769804	0.00271105	0.0186653	yes	up
ENSMUSG00000066363	Serpina3f	serine (or cysteine) peptidase inhibitor, clade A, member 3F [Source:MGI	3.126913821	1.644739458	0.00271472	0.01868315	yes	up
ENSMUSG00000099411	2310015D24Rik	RIKEN cDNA 2310015D24 gene [Source:MGI Symbol;Acc:MGI:1917350]	4.730606466	2.24202515	0.00271735	0.0186938	yes	up
ENSMUSG00000074228	Gm10645	predicted gene 10645 [Source:MGI Symbol;Acc:MGI:3704313]	4.968954269	2.312942264	0.00272506	0.01873197	yes	up
ENSMUSG00000096459	Ighv9-3	immunoglobulin heavy variable V9-3 [Source:MGI Symbol;Acc:MGI:3642720]	4.510581061	2.173313296	0.00273432	0.01878738	yes	up
ENSMUSG00000033308	Dpyd	dihydropyrimidine dehydrogenase [Source:MGI Symbol;Acc:MGI:2139667]	2.101549186	1.071453223	0.00274859	0.01884875	yes	up
ENSMUSG00000019899	Lama2	laminin, alpha 2 [Source:MGI Symbol;Acc:MGI:99912]	2.134442582	1.093859354	0.00276637	0.01894429	yes	up
ENSMUSG00000036862	Dchs1	dachsous cadherin related 1 [Source:MGI Symbol;Acc:MGI:2685011]	2.154697491	1.107485336	0.00278179	0.01902359	yes	up
ENSMUSG00000106715	Tmem265	transmembrane protein 265 [Source:MGI Symbol;Acc:MGI:5613213]	0.480622755	-1.057023141	0.00282658	0.01926867	yes	down
ENSMUSG00000103409	Lsmem2	leucine-rich single-pass membrane protein 2 [Source:MGI Symbol;Acc:MGI:3612240]	2.706735603	1.43655397	0.00282878	0.01926867	yes	up
ENSMUSG00000059022	Kcp	kielin/chordin-like protein [Source:MGI	2.318727119	1.213333045	0.00285136	0.01939302	yes	up
ENSMUSG00000083878	Gm12034	predicted gene 12034 [Source:MGI Symbol;Acc:MGI:3651409]	0.277184902	-1.851079417	0.0028583	0.01943146	yes	down

ENSMUSG00000047977	Synb	syncytin b [Source:MGI Symbol;Acc:MGI:3045308]	0.227583059	-2.135534926	0.00286373	0.01946073	yes	down
ENSMUSG00000013523	Bcas1	breast carcinoma amplified sequence 1 [Source:MGI Symbol;Acc:MGI:1924210]	0.493264218	-1.019567459	0.00288236	0.01954895	yes	down
ENSMUSG00000017969	Ptgis	prostaglandin I2 (prostacyclin) synthase [Source:MGI Symbol;Acc:MGI:1097156]	4.589680193	2.198393631	0.00288489	0.01955842	yes	up
ENSMUSG00000071691	Gm960	predicted gene 960 [Source:MGI Symbol;Acc:MGI:2685806]	3.749303027	1.906622432	0.00289872	0.01961369	yes	up
ENSMUSG00000024210	Ip6k3	inositol hexaphosphate kinase 3 [Source:MGI Symbol;Acc:MGI:3045325]	2.206454346	1.141729897	0.00290834	0.01966345	yes	up
ENSMUSG00000030433	Sbk2	SH3-binding domain kinase family, member 2 [Source:MGI Symbol;Acc:MGI:2685925]	2.939045962	1.555347921	0.00292103	0.01972609	yes	up
ENSMUSG00000021933	Gucy1b2	guanylate cyclase 1, soluble, beta 2 [Source:MGI Symbol;Acc:MGI:2660873]	0.087736216	-3.510683711	0.00295456	1	yes	down
ENSMUSG00000022887	Masp1	mannan-binding lectin serine peptidase 1 [Source:MGI Symbol;Acc:MGI:88492]	2.205618393	1.141183204	0.00299644	0.02012527	yes	up
ENSMUSG00000111192	Gm47128	predicted gene, 47128 [Source:MGI Symbol;Acc:MGI:6095880]	4.038644994	2.013871336	0.00301283	0.02021324	yes	up
ENSMUSG00000097924	A730020E08Rik	RIKEN cDNA A730020E08 gene [Source:MGI Symbol;Acc:MGI:2444999]	0.393957939	-1.343886485	0.00301305	0.02021324	yes	down
ENSMUSG00000022504	Ciita	class II transactivator [Source:MGI Symbol;Acc:MGI:108445]	2.044622884	1.031834773	0.00304817	0.02040136	yes	up
ENSMUSG00000044576	Garem2	GRB2 associated regulator of MAPK1 subtype 2 [Source:MGI Symbol;Acc:MGI:2685290]	3.091465295	1.62829081	0.00305162	0.02040913	yes	up
ENSMUSG00000094162	Gm2457	predicted gene, 2457 [Source:MGI Symbol;Acc:MGI:3780624]	2.956501829	1.56389117	0.00307442	0.02050764	yes	up
ENSMUSG00000031845	Bco1	beta-carotene oxygenase 1 [Source:MGI Symbol;Acc:MGI:1926923]	0.292820169	-1.771913167	0.00307526	0.02050764	yes	down
ENSMUSG00000023979	Guca1b	guanylate cyclase activator 1B [Source:MGI Symbol;Acc:MGI:1194489]	0.210518723	-2.247979548	0.00307708	0.02050764	yes	down
ENSMUSG00000050272	Dscam	DS cell adhesion molecule [Source:MGI Symbol;Acc:MGI:1196281]	2.80169003	1.48629735	0.00309418	0.02059771	yes	up
ENSMUSG00000030789	Itgax	integrin alpha X [Source:MGI Symbol;Acc:MGI:96609]	2.175784893	1.121535933	0.00310128	0.02062903	yes	up
ENSMUSG00000041608	Entpd3	ectonucleoside triphosphate diphosphohydrolase 3 [Source:MGI Symbol;Acc:MGI:1321386]	0.442571993	-1.176015939	0.00311732	0.02071982	yes	down
ENSMUSG00000037996	Slc24a2	solute carrier family 24 (sodium/potassium/calcium exchanger), member 2 [Source:MGI Symbol;Acc:MGI:1923626]	4.383788698	2.132178261	0.00314538	0.02083223	yes	up
ENSMUSG00000060924	Csmd1	CUB and Sushi multiple domains 1 [Source:MGI Symbol;Acc:MGI:2137383]	2.496452713	1.31987958	0.00319198	0.02106217	yes	up
ENSMUSG00000066191	Anks6	ankyrin repeat and sterile alpha motif domain containing 6 [Source:MGI Symbol;Acc:MGI:1922941]	2.010941796	1.007871325	0.00320019	0.02110834	yes	up
ENSMUSG00000032017	Grik4	glutamate receptor, ionotropic, kainate 4 [Source:MGI	0.457608769	-1.127813396	0.00321365	0.0211809	yes	down
ENSMUSG00000042476	Abcb4	ATP-binding cassette, sub-family B (MDR/TAP), member 4 [Source:MGI Symbol;Acc:MGI:97569]	2.122080897	1.085479655	0.00322781	0.0212401	yes	up
ENSMUSG00000018924	Alox15	arachidonate 15-lipoxygenase [Source:MGI	2.264560349	1.179230987	0.00325106	0.02134612	yes	up
ENSMUSG00000102615	Gm37844	predicted gene, 37844 [Source:MGI Symbol;Acc:MGI:5611072]	3.075813489	1.620968024	0.00328565	0.02154048	yes	up
ENSMUSG00000026414	Tnnt2	troponin T2, cardiac [Source:MGI Symbol;Acc:MGI:104597]	0.345515005	-1.533179729	0.00330686	0.02165572	yes	down
ENSMUSG00000036295	Lrrn3	leucine rich repeat protein 3, neuronal [Source:MGI Symbol;Acc:MGI:106036]	2.195076145	1.134270986	0.00332281	0.02175112	yes	up
ENSMUSG00000092517	Art2a	ADP-ribosyltransferase 2a [Source:MGI Symbol;Acc:MGI:107546]	2.160520792	1.111379115	0.00332656	0.02176344	yes	up
ENSMUSG00000046345	Smco1	single-pass membrane protein with coiled-coil domains 1 [Source:MGI Symbol;Acc:MGI:1916826]	3.88451595	1.957734836	0.00332721	0.02176344	yes	up
ENSMUSG00000082932	Cyp2j8	cytochrome P450, family 2, subfamily j, polypeptide 8 [Source:MGI Symbol;Acc:MGI:2449817]	4.070565652	2.025229288	0.00337057	0.02198882	yes	up
ENSMUSG00000090223	Pcp4	Purkinje cell protein 4 [Source:MGI Symbol;Acc:MGI:97509]	0.40767945	-1.294492859	0.00338569	0.02207916	yes	down
ENSMUSG00000061852	Gm53056	predicted gene 53056 [Source:MGI Symbol;Acc:MGI:6435214]	3.147138392	1.654040621	0.00340008	0.0221479	yes	up

ENSMUSG00000029163	Emilin1	elastin microfibril interfacer 1 [Source:MGI Symbol;Acc:MGI:1926189]	2.179923896	1.124277769	0.00341335	0.02221765	yes	up
ENSMUSG00000039376	Synpo2l	synaptopodin 2-like [Source:MGI Symbol;Acc:MGI:1916010]	2.105687481	1.074291332	0.00343311	0.02232102	yes	up
ENSMUSG00000078490	Cfap74	cilia and flagella associated protein 74 [Source:MGI Symbol;Acc:MGI:1917130]	2.793021482	1.48182667	0.00343643	0.02233424	yes	up
ENSMUSG00000118112	Gm50349	predicted gene, 50349 [Source:MGI Symbol;Acc:MGI:6303230]	0.083659423	-3.579328147	0.003447	1	yes	down
ENSMUSG00000027750	Postn	periostin, osteoblast specific factor [Source:MGI Symbol;Acc:MGI:1926321]	2.044089516	1.031458377	0.00345335	0.02242729	yes	up
ENSMUSG00000026834	Acvr1c	activin A receptor, type IC [Source:MGI Symbol;Acc:MGI:2661081]	2.196869116	1.13544892	0.00351385	0.02271784	yes	up
ENSMUSG00000046442	Ppm1e	protein phosphatase 1E (PP2C domain containing) [Source:MGI Symbol;Acc:MGI:2444096]	2.329017487	1.219721472	0.00352323	0.02276144	yes	up
ENSMUSG00000006269	Atp6v1b1	ATPase, H+ transporting, lysosomal V1 subunit B1 [Source:MGI Symbol;Acc:MGI:103285]	2.654363192	1.408365786	0.00353083	0.02279521	yes	up
ENSMUSG00000021534	1700001L19Rik	RIKEN cDNA 1700001L19 gene [Source:MGI Symbol;Acc:MGI:1916565]	0.123414497	-3.018416228	0.00357401	1	yes	down
ENSMUSG00000050505	Pcdh20	protocadherin 20 [Source:MGI Symbol;Acc:MGI:2443376]	2.70374835	1.43496088	0.00359489	0.02313789	yes	up
ENSMUSG00000095642	Ighv14-3	immunoglobulin heavy variable V14-3 [Source:MGI Symbol;Acc:MGI:4439764]	2.722063686	1.444700821	0.00361904	0.02323284	yes	up
ENSMUSG00000014846	Tppp3	tubulin polymerization-promoting protein family member 3 [Source:MGI	2.238182419	1.162327625	0.00363778	0.02332221	yes	up
ENSMUSG00000076569	Igkv5-39	immunoglobulin kappa variable 5-39 [Source:MGI Symbol;Acc:MGI:2686255]	9.273411584	3.213100188	0.00363971	0.02332221	yes	up
ENSMUSG00000029378	Areg	amphiregulin [Source:MGI Symbol;Acc:MGI:88068]	0.435821018	-1.198192323	0.00365731	0.02341762	yes	down
ENSMUSG00000039257	Vstm2b	V-set and transmembrane domain containing 2B [Source:MGI Symbol;Acc:MGI:1914525]	3.335932142	1.738089943	0.00366631	0.02345787	yes	up
ENSMUSG00000039057	Myo16	myosin XVI [Source:MGI Symbol;Acc:MGI:2685951]	3.763727148	1.912162043	0.0036766	0.02351503	yes	up
ENSMUSG00000045322	Tlr9	toll-like receptor 9 [Source:MGI Symbol;Acc:MGI:1932389]	2.133001036	1.092884666	0.00375208	0.02390932	yes	up
ENSMUSG00000026418	Tnni1	troponin I, skeletal, slow 1 [Source:MGI Symbol;Acc:MGI:105073]	0.372691259	-1.423947113	0.00383719	0.02437082	yes	down
ENSMUSG00000027894	Slc6a17	solute carrier family 6 (neurotransmitter transporter), member 17 [Source:MGI	2.161090365	1.111759399	0.0038974	0.02465564	yes	up
ENSMUSG00000086450	Macrod2os1	mono-ADP ribosylhydrolase 2, opposite strand 1 [Source:MGI Symbol;Acc:MGI:3650192]	0.029969598	-5.060356466	0.00391894	1	yes	down
ENSMUSG00000057706	Mex3b	mex3 RNA binding family member B [Source:MGI Symbol;Acc:MGI:1918252]	2.081540065	1.057651327	0.00393363	0.02481388	yes	up
ENSMUSG00000112014	Gm48435	predicted gene, 48435 [Source:MGI Symbol;Acc:MGI:6097938]	0.027264452	-5.196835027	0.00394491	1	yes	down
ENSMUSG00000073842	Mup7	major urinary protein 7 [Source:MGI Symbol;Acc:MGI:3709615]	2.591114329	1.373572674	0.00395203	0.02491712	yes	up
ENSMUSG00000031637	Lrp2bp	Lrp2 binding protein [Source:MGI Symbol;Acc:MGI:1914870]	3.221887126	1.687905952	0.00395825	0.02494722	yes	up
ENSMUSG00000029189	Sel1l3	sel-1 suppressor of lin-12-like 3 (C. elegans) [Source:MGI Symbol;Acc:MGI:1916941]	2.250803408	1.170440053	0.0039794	0.02503488	yes	up
ENSMUSG00000078952	Lncenc1	long non-coding RNA, embryonic stem cells expressed 1 [Source:MGI Symbol;Acc:MGI:3780541]	0.330452948	-1.597483228	0.00398503	0.02506118	yes	down
ENSMUSG00000047443	Erfe	erythroferrone [Source:MGI Symbol;Acc:MGI:3606476]	0.453964436	-1.139348815	0.00399204	0.02507966	yes	down
ENSMUSG00000041674	BC006965	cDNA sequence BC006965 [Source:MGI Symbol;Acc:MGI:2384955]	0.463843322	-1.108290525	0.00399232	0.02507966	yes	down
ENSMUSG00000075010	AW112010	expressed sequence AW112010 [Source:MGI Symbol;Acc:MGI:2147706]	2.029835004	1.021362462	0.00401132	0.02515334	yes	up
ENSMUSG00000037922	Bank1	B cell scaffold protein with ankyrin repeats 1 [Source:MGI Symbol;Acc:MGI:2442120]	2.643633553	1.402522212	0.00403579	0.02527924	yes	up
ENSMUSG00000085779	Atcayos	ataxia, cerebellar, Cayman type, opposite strand [Source:MGI Symbol;Acc:MGI:1916928]	2.188525104	1.129958933	0.0040629	0.02540306	yes	up
ENSMUSG00000097622	A330033J07Rik	RIKEN cDNA A330033J07 gene [Source:MGI Symbol;Acc:MGI:2444369]	0.090851649	-3.460343483	0.00408764	1	yes	down

ENSMUSG00000076749	Trgc1	T cell receptor gamma, constant 1 [Source:MGI	2.615476238	1.387073663	0.00409681	0.02555038	yes	up
ENSMUSG00000034648	Lrrn1	leucine rich repeat protein 1, neuronal [Source:MGI Symbol;Acc:MGI:106038]	2.397105564	1.261293444	0.00410186	0.0255542	yes	up
ENSMUSG00000057346	Apol9a	apolipoprotein L 9a [Source:MGI Symbol;Acc:MGI:3606001]	2.528952392	1.338539878	0.00411618	0.02563416	yes	up
ENSMUSG00000066058	Cldn19	claudin 19 [Source:MGI Symbol;Acc:MGI:3033992]	2.028629991	1.020505751	0.00412144	0.02565772	yes	up
ENSMUSG00000036330	Slc18a1	solute carrier family 18 (vesicular monoamine), member 1 [Source:MGI Symbol;Acc:MGI:106684]	0.361897507	-1.466346925	0.0041418	0.02575663	yes	down
ENSMUSG00000052544	St6galnac3	ST6 (alpha-N-acetyl-neuraminyl-2,3-beta-galactosyl-1,3)-N-acetylgalactosaminide alpha-2,6-sialyltransferase 3 [Source:MGI Symbol;Acc:MGI:1341828]	2.115684402	1.081124436	0.00414617	0.02577451	yes	up
ENSMUSG00000070687	Htr1d	5-hydroxytryptamine (serotonin) receptor 1D [Source:MGI Symbol;Acc:MGI:96276]	0.347897731	-1.523264823	0.00427586	0.0263606	yes	down
ENSMUSG00000046997	Spsb4	splA/ryanodine receptor domain and SOCS box containing 4 [Source:MGI Symbol;Acc:MGI:2183445]	0.434541502	-1.202434122	0.00434471	0.02665895	yes	down
ENSMUSG00000117901		novel transcript, antisense to Macrod1	0.266692965	-1.906748327	0.0043455	0.02665895	yes	down
ENSMUSG00000032186	Tmod2	tropomodulin 2 [Source:MGI Symbol;Acc:MGI:1355335]	2.020507035	1.014717375	0.0043901	0.02685928	yes	up
ENSMUSG00000094526	Gm21451	predicted gene, 21451 [Source:MGI Symbol;Acc:MGI:5434806]	2.166240846	1.115193653	0.00439305	0.02685928	yes	up
ENSMUSG00000000305	Cdh4	cadherin 4 [Source:MGI Symbol;Acc:MGI:99218]	2.325434939	1.217500577	0.00442752	0.02701835	yes	up
ENSMUSG00000062488	Ifit3b	interferon-induced protein with tetratricopeptide repeats 3B [Source:MGI Symbol;Acc:MGI:3698419]	2.204141285	1.140216703	0.00443646	0.02704424	yes	up
ENSMUSG00000097762	4732463B04Rik	RIKEN cDNA 4732463B04 gene [Source:MGI Symbol;Acc:MGI:3642483]	0.066347509	-3.913813884	0.00445201	1	yes	down
ENSMUSG00000053654	Krt42	keratin 42 [Source:MGI Symbol;Acc:MGI:1915489]	0.435987177	-1.197642391	0.00447099	0.02718763	yes	down
ENSMUSG00000038725	Pkhd1l1	polycystic kidney and hepatic disease 1-like 1 [Source:MGI Symbol;Acc:MGI:2183153]	2.214546871	1.147011532	0.00451716	0.02739135	yes	up
ENSMUSG00000091421	Gm4202	predicted gene 4202 [Source:MGI Symbol;Acc:MGI:3782379]	53.74047891	5.747937273	0.00453201	1	yes	up
ENSMUSG00000031410	Nxf7	nuclear RNA export factor 7 [Source:MGI Symbol;Acc:MGI:2159343]	0.300854706	-1.732861173	0.00457586	0.0276711	yes	down
ENSMUSG00000042453	Reln	reelin [Source:MGI Symbol;Acc:MGI:103022]	2.021272153	1.015263586	0.00458204	0.02768763	yes	up
ENSMUSG00000099032	Tcf24	transcription factor 24 [Source:MGI Symbol;Acc:MGI:3780500]	3.149111458	1.654944821	0.00459926	0.02775379	yes	up
ENSMUSG00000036131	Frmd7	FERM domain containing 7 [Source:MGI Symbol;Acc:MGI:2686379]	0.24561226	-2.025545518	0.00462462	0.02786136	yes	down
ENSMUSG00000040138	Ndp	Norrie disease (pseudoglioma) (human) [Source:MGI Symbol;Acc:MGI:102570]	2.424541045	1.277711677	0.0046253	0.02786136	yes	up
ENSMUSG00000017978	Cadps2	Ca2+-dependent activator protein for secretion 2 [Source:MGI Symbol;Acc:MGI:2443963]	2.312211721	1.209273506	0.00464662	0.0279366	yes	up
ENSMUSG00000019359	Gdpd2	glycerophosphodiester phosphodiesterase domain containing 2 [Source:MGI Symbol;Acc:MGI:1918834]	2.526954662	1.33739978	0.00468541	0.02809618	yes	up
ENSMUSG00000052584	Serp2	stress-associated endoplasmic reticulum protein family member 2 [Source:MGI	3.640845174	1.864273392	0.00470186	0.0281851	yes	up
ENSMUSG00000024353	Mzb1	marginal zone B and B1 cell-specific protein 1 [Source:MGI Symbol;Acc:MGI:1917066]	2.003626013	1.002613247	0.00473277	0.02831144	yes	up
ENSMUSG00000096847	Tmem151b	transmembrane protein 151B [Source:MGI Symbol;Acc:MGI:2685169]	4.107419712	2.038232375	0.00474277	0.02836148	yes	up
ENSMUSG00000027075	Slc43a1	solute carrier family 43, member 1 [Source:MGI Symbol;Acc:MGI:1931352]	2.065122773	1.046227554	0.0047597	0.02845255	yes	up
ENSMUSG00000027925	Sprr2j-ps	small proline-rich protein 2J, pseudogene [Source:MGI Symbol;Acc:MGI:1330345]	4.217480826	2.07638151	0.00477608	0.02852119	yes	up
ENSMUSG00000096006	Gm21596	predicted gene, 21596 [Source:MGI Symbol;Acc:MGI:5434951]	0.463426013	-1.109589066	0.00477847	0.02852563	yes	down
ENSMUSG00000114036	Gm48228	predicted gene, 48228 [Source:MGI Symbol;Acc:MGI:6097631]	0.375099124	-1.414656201	0.00482664	0.02871404	yes	down

ENSMUSG00000096826	Ccl27b	chemokine (C-C motif) ligand 27b [Source:MGI Symbol;Acc:MGI:1891389]	0.319613413	-1.64560014	0.00484404	0.02877912	yes	down
ENSMUSG00000094315	Igkv4-78	immunoglobulin kappa variable 4-78 [Source:MGI Symbol;Acc:MGI:3819775]	10.0235869	3.325326959	0.00486401	0.02885688	yes	up
ENSMUSG00000095197	Ighv1-59	immunoglobulin heavy variable V1-59 [Source:MGI Symbol;Acc:MGI:3644474]	5.32603585	2.41306214	0.00489253	0.0290019	yes	up
ENSMUSG00000025425	St8sia5	ST8 alpha-N-acetyl-neuraminide alpha-2,8-sialyltransferase 5 [Source:MGI	3.370207274	1.752837322	0.00489348	0.0290019	yes	up
ENSMUSG00000038173	Enpp6	ectonucleotide pyrophosphatase/phosphodiesterase 6 [Source:MGI Symbol;Acc:MGI:2445171]	2.300637517	1.202033694	0.00491707	0.02912169	yes	up
ENSMUSG00000095612	Ighv5-4	immunoglobulin heavy variable 5-4 [Source:MGI Symbol;Acc:MGI:4439895]	3.082510218	1.624105677	0.0049315	0.02917722	yes	up
ENSMUSG00000006642	Tcf23	transcription factor 23 [Source:MGI Symbol;Acc:MGI:1934960]	2.239073952	1.162902178	0.0050225	0.02963709	yes	up
ENSMUSG00000101791	2210011K15Rik	RIKEN cDNA 2210011K15 gene [Source:MGI Symbol;Acc:MGI:1919615]	0.120463095	-3.053336868	0.00504286	1	yes	down
ENSMUSG00000109973	Gm45397	predicted gene 45397 [Source:MGI Symbol;Acc:MGI:5791233]	0.408711581	-1.290844973	0.00511021	0.03004047	yes	down
ENSMUSG00000031302	Nlgn3	neuroligin 3 [Source:MGI Symbol;Acc:MGI:2444609]	2.840608759	1.506200141	0.00511139	0.03004047	yes	up
ENSMUSG00000026828	Galnt5	polypeptide N-acetylgalactosaminyltransferase 5 [Source:MGI Symbol;Acc:MGI:2179403]	2.267527676	1.181120159	0.00511216	0.03004047	yes	up
ENSMUSG00000044548	Dact1	dishevelled-binding antagonist of beta-catenin 1 [Source:MGI Symbol;Acc:MGI:1891740]	2.271254421	1.183489323	0.00520073	0.03048836	yes	up
ENSMUSG00000028488	Sh3gl2	SH3-domain GRB2-like 2 [Source:MGI	2.084418281	1.059644813	0.00522854	0.03059953	yes	up
ENSMUSG00000023153	Tmem52	transmembrane protein 52 [Source:MGI	2.361540841	1.239728486	0.00526543	0.03079457	yes	up
ENSMUSG00000023341	Mx2	MX dynamin-like GTPase 2 [Source:MGI	2.00228837	1.001649767	0.00529296	0.03088822	yes	up
ENSMUSG00000094638	Gm21972	predicted gene 21972 [Source:MGI Symbol;Acc:MGI:5439441]	0.019649704	-5.669348588	0.00530003	1	yes	down
ENSMUSG00000095416	Ighv1-12	immunoglobulin heavy variable V1-12 [Source:MGI Symbol;Acc:MGI:3646284]	2.730818228	1.449333287	0.00530294	0.03090939	yes	up
ENSMUSG00000094075	Ighv1-80	immunoglobulin heavy variable 1-80 [Source:MGI Symbol;Acc:MGI:4439738]	2.444217394	1.289372607	0.00535177	0.03112052	yes	up
ENSMUSG00000000544	Gpa33	glycoprotein A33 (transmembrane) [Source:MGI Symbol;Acc:MGI:1891703]	0.262418863	-1.930056668	0.00536643	0.03115341	yes	down
ENSMUSG00000051617	Krt9	keratin 9 [Source:MGI Symbol;Acc:MGI:96696]	3.896559685	1.962200915	0.0053797	0.03120949	yes	up
ENSMUSG00000029636	Wasf3	WASP family, member 3 [Source:MGI	3.702497176	1.888498635	0.00544425	0.0315205	yes	up
ENSMUSG00000032807	Alox12b	arachidonate 12-lipoxygenase, 12R type [Source:MGI Symbol;Acc:MGI:1274782]	2.025002845	1.017923935	0.00552901	0.0319431	yes	up
ENSMUSG00000029320	1700016H13Rik	RIKEN cDNA 1700016H13 gene [Source:MGI Symbol;Acc:MGI:1921468]	0.17865195	-2.484776437	0.00560253	0.03225323	yes	down
ENSMUSG00000103765	Gm37857	predicted gene, 37857 [Source:MGI Symbol;Acc:MGI:5611085]	5.361570118	2.422655551	0.0057128	1	yes	up
ENSMUSG00000026173	Plcd4	phospholipase C, delta 4 [Source:MGI Symbol;Acc:MGI:107469]	3.092639719	1.628838775	0.0057179	0.03278641	yes	up
ENSMUSG00000079457	Gm7609	predicted pseudogene 7609 [Source:MGI Symbol;Acc:MGI:3644536]	2.731064247	1.449463253	0.00572953	0.03283132	yes	up
ENSMUSG00000056222	Spock1	sparc/osteonectin, cwcv and kazal-like domains proteoglycan 1 [Source:MGI	3.150175024	1.655431987	0.00574913	0.0329327	yes	up
ENSMUSG00000116560	Gm2808	predicted gene 2808 [Source:MGI Symbol;Acc:MGI:3780977]	2.562612024	1.357615073	0.00592994	0.03370043	yes	up
ENSMUSG00000026527	Rgs7	regulator of G protein signaling 7 [Source:MGI Symbol;Acc:MGI:1346089]	2.180792904	1.124852773	0.00594791	0.03379146	yes	up
ENSMUSG00000095583	Ighv14-2	immunoglobulin heavy variable 14-2 [Source:MGI Symbol;Acc:MGI:4439607]	3.754207721	1.908508477	0.00602742	0.03415893	yes	up
ENSMUSG00000054134	Umodl1	uromodulin-like 1 [Source:MGI Symbol;Acc:MGI:1929785]	0.253994199	-1.977132548	0.0060284	0.03415893	yes	down
ENSMUSG00000020872	Tac4	tachykinin 4 [Source:MGI Symbol;Acc:MGI:1931130]	2.960980519	1.566074998	0.00603327	0.03417533	yes	up
ENSMUSG00000037705	Tecta	tectorin alpha [Source:MGI Symbol;Acc:MGI:109575]	0.316985336	-1.657511993	0.00606413	0.03427391	yes	down

ENSMUSG00000041301	Cftr	cystic fibrosis transmembrane conductance regulator [Source:MGI Symbol;Acc:MGI:88388]	0.135738885	-2.881094027	0.00606776	1	yes	down
ENSMUSG00000053624	Gykl1	glycerol kinase-like 1 [Source:MGI Symbol;Acc:MGI:891990]	0.059125177	-4.080083587	0.00609213	1	yes	down
ENSMUSG00000073830	Mup14	major urinary protein 14 [Source:MGI Symbol;Acc:MGI:3702005]	2.127070598	1.088867918	0.00610692	0.03443456	yes	up
ENSMUSG00000024593	Megf10	multiple EGF-like-domains 10 [Source:MGI Symbol;Acc:MGI:2685177]	2.710492999	1.43855528	0.00611293	0.03445724	yes	up
ENSMUSG00000091712	Sec14l5	SEC14-like lipid binding 5 [Source:MGI	2.004346776	1.003132134	0.00612394	0.03450284	yes	up
ENSMUSG00000102705	4632432E15Rik	RIKEN cDNA 4632432E15 gene [Source:MGI Symbol;Acc:MGI:1921296]	0.387558467	-1.367514124	0.00612501	0.03450284	yes	down
ENSMUSG00000021792	Prxl2a	peroxiredoxin like 2A [Source:MGI Symbol;Acc:MGI:1917814]	2.04261016	1.030413886	0.00638128	0.03559845	yes	up
ENSMUSG00000040650	Cyp2b23	cytochrome P450, family 2, subfamily b, polypeptide 23 [Source:MGI Symbol;Acc:MGI:3646735]	4.996881291	2.321027945	0.00640613	0.03572552	yes	up
ENSMUSG00000104452	Ighv8-8	immunoglobulin heavy variable 8-8 [Source:MGI Symbol;Acc:MGI:3815333]	10.92519286	3.449586843	0.00642172	0.0357894	yes	up
ENSMUSG00000030450	Oca2	oculocutaneous albinism II [Source:MGI	0.076724978	-3.704159866	0.00642664	0.03580526	yes	down
ENSMUSG00000030621	Me3	malic enzyme 3, NADP(+)-dependent, mitochondrial [Source:MGI	2.010206579	1.007343768	0.00644264	0.0358713	yes	up
ENSMUSG00000049097	Ankrd34a	ankyrin repeat domain 34A [Source:MGI Symbol;Acc:MGI:3617846]	3.73634365	1.901627153	0.00644563	0.03587638	yes	up
ENSMUSG00000095026	Gm3336	predicted gene 3336 [Source:MGI Symbol;Acc:MGI:3781514]	2.865555559	1.518814868	0.00646381	0.03595443	yes	up
ENSMUSG00000098975	Gm27177	predicted gene 27177 [Source:MGI Symbol;Acc:MGI:5521020]	0.499602846	-1.0011464	0.00648478	0.03604788	yes	down
ENSMUSG00000048138	Dmrt2	doublesex and mab-3 related transcription factor 2 [Source:MGI Symbol;Acc:MGI:1330307]	2.603728279	1.380578899	0.00652888	0.03623477	yes	up
ENSMUSG00000004031	Brinp2	bone morphogenic protein/retinoic acid inducible neural-specific 2 [Source:MGI	0.359492155	-1.475967808	0.00663076	0.03668243	yes	down
ENSMUSG00000038192	Cer1	cerberus 1, DAN family BMP antagonist [Source:MGI Symbol;Acc:MGI:1201414]	0.183658283	-2.444904135	0.00663452	0.03669149	yes	down
ENSMUSG00000004110	Cacna1e	calcium channel, voltage-dependent, R type, alpha 1E subunit [Source:MGI Symbol;Acc:MGI:106217]	2.551668889	1.351441134	0.00664759	0.03671328	yes	up
ENSMUSG00000089953	Rnf224	ring finger protein 224 [Source:MGI Symbol;Acc:MGI:2685603]	2.873781376	1.522950312	0.00665667	0.03673171	yes	up
ENSMUSG00000064201	Krt2	keratin 2 [Source:MGI Symbol;Acc:MGI:96699]	0.254627437	-1.973540215	0.00665956	0.03673597	yes	down
ENSMUSG00000095335	Igkv3-5	immunoglobulin kappa chain variable 3-5 [Source:MGI Symbol;Acc:MGI:1330854]	2.274472618	1.185532066	0.00670942	0.0369116	yes	up
ENSMUSG00000118458	Gm10599	predicted pseudogene 10599 [Source:MGI Symbol;Acc:MGI:3710582]	4.434031141	2.148618903	0.00671653	0.03693239	yes	up
ENSMUSG00000084929	Foxo6os	forkhead box O6, opposite strand [Source:MGI Symbol;Acc:MGI:3028036]	7.364245586	2.880537739	0.00672121	1	yes	up
ENSMUSG00000055809	Dnaaf3	dynein, axonemal assembly factor 3 [Source:MGI Symbol;Acc:MGI:3588207]	2.095624038	1.067379916	0.00672228	0.03694748	yes	up
ENSMUSG00000026981	Il1rn	interleukin 1 receptor antagonist [Source:MGI	0.497610271	-1.006911832	0.00672355	0.03694748	yes	down
ENSMUSG00000076548	Igkv4-69	immunoglobulin kappa variable 4-69 [Source:MGI Symbol;Acc:MGI:3648668]	26.91974714	4.750592954	0.00672965	1	yes	up
ENSMUSG00000109877	Gm45609	predicted gene 45609 [Source:MGI Symbol;Acc:MGI:5791445]	2.646947022	1.40432932	0.00674007	0.03701474	yes	up
ENSMUSG00000053166	Cdh22	cadherin 22 [Source:MGI Symbol;Acc:MGI:1341843]	0.228053015	-2.132558852	0.00675279	0.03706983	yes	down
ENSMUSG00000081512	Gm15821	predicted gene 15821 [Source:MGI Symbol;Acc:MGI:3801940]	0.334398858	-1.580358173	0.00675439	0.03706983	yes	down
ENSMUSG00000047495	Dlgap2	DLG associated protein 2 [Source:MGI	5.768128771	2.528103372	0.00678882	0.03719969	yes	up
ENSMUSG00000026784	Pdss1	prenyl (solanesyl) diphosphate synthase, subunit 1 [Source:MGI Symbol;Acc:MGI:1889278]	2.002431817	1.001753119	0.00679914	0.03723264	yes	up
ENSMUSG00000097752	Gm26688	predicted gene, 26688 [Source:MGI Symbol;Acc:MGI:5477182]	0.342277665	-1.54676094	0.00684986	0.03747482	yes	down
ENSMUSG00000076508	Igkv17-127	immunoglobulin kappa variable 17-127 [Source:MGI Symbol;Acc:MGI:3646891]	3.116981633	1.640149654	0.00706358	0.03837514	yes	up

ENSMUSG00000041567	Serpina12	serine (or cysteine) peptidase inhibitor, clade A (alpha-1 antiproteinase, antitrypsin), member 12 [Source:MGI	2.124672076	1.087240191	0.00713029	0.03860583	yes	up
ENSMUSG00000026012	Cd28	CD28 antigen [Source:MGI Symbol;Acc:MGI:88327]	2.265903357	1.18008633	0.00714879	0.03866968	yes	up
ENSMUSG00000033579	Fa2h	fatty acid 2-hydroxylase [Source:MGI Symbol;Acc:MGI:2443327]	4.58646955	2.197384062	0.00717294	0.03877606	yes	up
ENSMUSG00000108195	Gm44415	predicted gene, 44415 [Source:MGI Symbol;Acc:MGI:5690807]	10.48411497	3.390133174	0.00718016	1	yes	up
ENSMUSG00000059974	Ntm	neurotrimin [Source:MGI Symbol;Acc:MGI:2446259]	2.246257351	1.167523225	0.00726445	0.03916053	yes	up
ENSMUSG00000051111	Sv2c	synaptic vesicle glycoprotein 2c [Source:MGI Symbol;Acc:MGI:1922459]	3.207259381	1.681341034	0.00731959	0.03935961	yes	up
ENSMUSG00000074472	Zfp872	zinc finger protein 872 [Source:MGI Symbol;Acc:MGI:3588272]	3.290943322	1.71850118	0.00743461	0.0397678	yes	up
ENSMUSG00000090942	F830016B08Rik	RIKEN cDNA F830016B08 gene [Source:MGI Symbol;Acc:MGI:3588218]	2.17616153	1.121785648	0.00746906	0.03992736	yes	up
ENSMUSG00000094433	Igkv5-43	immunoglobulin kappa chain variable 5-43 [Source:MGI Symbol;Acc:MGI:4943320]	3.019001385	1.594071419	0.0075904	0.04037624	yes	up
ENSMUSG00000050587	Lrrc4c	leucine rich repeat containing 4C [Source:MGI Symbol;Acc:MGI:2442636]	2.784854707	1.47760206	0.00765198	0.04062883	yes	up
ENSMUSG00000117748	Derpc	DERPC proline and glycine rich nuclear protein [Source:MGI Symbol;Acc:MGI:6303050]	0.379151977	-1.399151848	0.00767193	0.04070973	yes	down
ENSMUSG00000033849	B3galt2	UDP-Gal:betaGlcNAc beta 1,3-galactosyltransferase, polypeptide 2 [Source:MGI Symbol;Acc:MGI:1349461]	2.007271415	1.005235705	0.0077137	0.04085613	yes	up
ENSMUSG00000076564	Igkv12-46	immunoglobulin kappa variable 12-46 [Source:MGI Symbol;Acc:MGI:4439773]	2.550133212	1.350572611	0.00771862	0.04085716	yes	up
ENSMUSG00000110696	Gm45706	predicted gene 45706 [Source:MGI Symbol;Acc:MGI:5804821]	0.02099222	-5.574001467	0.00772567	1	yes	down
ENSMUSG00000111291	Gm48604	predicted gene, 48604 [Source:MGI Symbol;Acc:MGI:6098186]	0.37887251	-1.400215629	0.00773408	0.04090142	yes	down
ENSMUSG00000068129	Cst7	cystatin F (leukocystatin) [Source:MGI	2.159847694	1.110929582	0.00779358	0.04110293	yes	up
ENSMUSG00000029121	Crmp1	collapsin response mediator protein 1 [Source:MGI Symbol;Acc:MGI:107793]	2.228159651	1.155852608	0.00781074	0.04114321	yes	up
ENSMUSG00000020096	Tbata	thymus, brain and testes associated [Source:MGI Symbol;Acc:MGI:1923820]	5.709921684	2.513470958	0.00795733	1	yes	up
ENSMUSG00000104371	Gm37513	predicted gene, 37513 [Source:MGI Symbol;Acc:MGI:5610741]	0.177128869	-2.497128731	0.00795826	1	yes	down
ENSMUSG00000102660	Gm38378	predicted gene, 38378 [Source:MGI Symbol;Acc:MGI:5611606]	0.177128869	-2.497128731	0.00795826	1	yes	down
ENSMUSG00000043165	Lor	loricrin [Source:MGI Symbol;Acc:MGI:96816]	2.26731736	1.180986341	0.00821021	0.04290754	yes	up
ENSMUSG00000015890	Amdhd1	amidohydrolase domain containing 1 [Source:MGI Symbol;Acc:MGI:1919011]	0.323592603	-1.627749465	0.00824901	0.04303014	yes	down
ENSMUSG00000032503	Arpp21	cyclic AMP-regulated phosphoprotein, 21 [Source:MGI Symbol;Acc:MGI:107562]	2.914230395	1.543114939	0.00825607	0.04303014	yes	up
ENSMUSG00000078606	Gvin2	GTPase, very large interferon inducible, family member 2 [Source:MGI	2.036013071	1.025746823	0.00829352	0.04313996	yes	up
ENSMUSG00000033544	Angptl1	angiopoietin-like 1 [Source:MGI Symbol;Acc:MGI:1919963]	4.216380799	2.076005169	0.00841113	0.04355528	yes	up
ENSMUSG00000047040	Prr15l	proline rich 15-like [Source:MGI Symbol;Acc:MGI:2387599]	0.127394666	-2.972623221	0.00847052	1	yes	down
ENSMUSG00000072769	Gm10419	predicted gene 10419 [Source:MGI Symbol;Acc:MGI:3642823]	2.949130517	1.560289672	0.00847935	0.04381084	yes	up
ENSMUSG00000005131	4930550C14Rik	RIKEN cDNA 4930550C14 gene [Source:MGI Symbol;Acc:MGI:1922561]	2.758912972	1.464099949	0.0085311	0.04403873	yes	up
ENSMUSG00000026589	Sec16b	SEC16 homolog B (S. cerevisiae) [Source:MGI Symbol;Acc:MGI:2148802]	2.068418298	1.048527973	0.00855518	0.04413765	yes	up
ENSMUSG00000104358	Gm37127	predicted gene, 37127 [Source:MGI Symbol;Acc:MGI:5610355]	0.245643803	-2.025360253	0.00855537	0.04413765	yes	down
ENSMUSG00000047976	Kcna1	potassium voltage-gated channel, shaker-related subfamily, member 1 [Source:MGI	2.619543614	1.389315482	0.00860054	0.04429526	yes	up
ENSMUSG00000091956	C2cd4b	C2 calcium-dependent domain containing 4B [Source:MGI Symbol;Acc:MGI:1922947]	0.31755269	-1.654932103	0.00863073	0.0443748	yes	down
ENSMUSG00000022842	Ece2	endothelin converting enzyme 2 [Source:MGI Symbol;Acc:MGI:1101356]	2.012317564	1.008857995	0.00863256	0.0443748	yes	up

ENSMUSG00000078302	Foxd1	forkhead box D1 [Source:MGI Symbol;Acc:MGI:1347463]	2.728676821	1.448201535	0.00867228	0.04448107	yes	up
ENSMUSG00000085996	A830012C17Rik	RIKEN cDNA A830012C17 gene [Source:MGI Symbol;Acc:MGI:2441830]	18.79352257	4.232163599	0.00868992	1	yes	up
ENSMUSG00000079339	Ifit1bl1	interferon induced protein with tetratricpeptide repeats 1B like 1 [Source:MGI Symbol;Acc:MGI:3650685]	2.305275259	1.204939024	0.00871417	0.04460827	yes	up
ENSMUSG00000084897	Gm14226	predicted gene 14226 [Source:MGI Symbol;Acc:MGI:3649244]	0.284085009	-1.81560539	0.00871626	0.04460827	yes	down
ENSMUSG00000090722	Gm8378	predicted gene 8378 [Source:MGI Symbol;Acc:MGI:3646777]	0.427404147	-1.226327188	0.0087564	0.04480042	yes	down
ENSMUSG00000038984	Tspyl5	testis-specific protein, Y-encoded-like 5 [Source:MGI Symbol;Acc:MGI:2442458]	2.034491364	1.024668156	0.00876495	0.04483089	yes	up
ENSMUSG00000026166	Ccl20	chemokine (C-C motif) ligand 20 [Source:MGI Symbol;Acc:MGI:1329031]	0.412882623	-1.276196395	0.00887963	0.04523281	yes	down
ENSMUSG00000076501	Igkv2-137	immunoglobulin kappa chain variable 2-137 [Source:MGI Symbol;Acc:MGI:4439879]	13.23193337	3.72595197	0.00893929	0.04548039	yes	up
ENSMUSG00000025175	Fn3k	fructosamine 3 kinase [Source:MGI Symbol;Acc:MGI:1926834]	2.326185984	1.217966449	0.00896754	0.0455437	yes	up
ENSMUSG00000035448	Ccr3	chemokine (C-C motif) receptor 3 [Source:MGI Symbol;Acc:MGI:104616]	2.17244516	1.119319759	0.00904271	0.04578871	yes	up
ENSMUSG00000102439	Flg	filaggrin [Source:MGI Symbol;Acc:MGI:95553]	2.098753679	1.069532854	0.00907578	0.04591807	yes	up
ENSMUSG00000094198	Ighv1-50	immunoglobulin heavy variable 1-50 [Source:MGI Symbol;Acc:MGI:4439753]	2.093643404	1.066015739	0.00916507	0.0463021	yes	up
ENSMUSG00000085584	Rtl9	retrotransposon Gag like 9 [Source:MGI Symbol;Acc:MGI:2685231]	3.65577667	1.87017794	0.00925552	0.04658206	yes	up
ENSMUSG00000035045	Zc3h12b	zinc finger CCCH-type containing 12B [Source:MGI Symbol;Acc:MGI:2442133]	2.270669096	1.183117478	0.00927526	0.04665831	yes	up
ENSMUSG00000037579	Kcnh3	potassium voltage-gated channel, subfamily H (eag-related), member 3 [Source:MGI Symbol;Acc:MGI:1341723]	3.060554126	1.613792883	0.00928201	0.04667463	yes	up
ENSMUSG00000100599	1700120C14Rik	RIKEN cDNA 1700120C14 gene [Source:MGI Symbol;Acc:MGI:1920850]	2.352689346	1.234310837	0.00936284	0.0469718	yes	up
ENSMUSG00000063388	BC023105	cDNA sequence BC023105 [Source:MGI Symbol;Acc:MGI:2384767]	3.00867724	1.589129348	0.00940253	0.04710258	yes	up
ENSMUSG00000074141	Il4i1	interleukin 4 induced 1 [Source:MGI Symbol;Acc:MGI:109552]	2.404923307	1.265990887	0.00962993	0.0480191	yes	up
ENSMUSG00000037418	Best1	bestrophin 1 [Source:MGI Symbol;Acc:MGI:1346332]	2.717201734	1.44212168	0.00966407	0.04814768	yes	up
ENSMUSG00000107611	Gm44131	predicted gene, 44131 [Source:MGI Symbol;Acc:MGI:5690523]	0.048313179	-4.371439394	0.00967351	1	yes	down
ENSMUSG00000056895	H2bu2	H2B.U histone 2 [Source:MGI Symbol;Acc:MGI:1925553]	0.440033781	-1.184313813	0.00969006	0.04822158	yes	down
ENSMUSG00000071113	Mboat4	membrane bound O-acyltransferase domain containing 4 [Source:MGI Symbol;Acc:MGI:2685017]	0.275109662	-1.861921287	0.00971349	0.04826871	yes	down
ENSMUSG00000096490	Igkv10-94	immunoglobulin kappa variable 10-94 [Source:MGI Symbol;Acc:MGI:3646140]	3.094074699	1.629508028	0.0097379	0.04836221	yes	up
ENSMUSG00000074505	Fat3	FAT atypical cadherin 3 [Source:MGI Symbol;Acc:MGI:2444314]	3.558603455	1.831311178	0.0098069	0.04856536	yes	up
ENSMUSG00000117123	Gm49890	predicted gene, 49890 [Source:MGI Symbol;Acc:MGI:6270578]	0.475256464	-1.073221845	0.00983405	0.04865799	yes	down
ENSMUSG00000094796	BC147527	cDNA sequence BC147527 [Source:MGI Symbol;Acc:MGI:4840510]	2.037685216	1.0269312	0.00991716	0.04899909	yes	up
ENSMUSG00000025497	Cdhr5	cadherin-related family member 5 [Source:MGI Symbol;Acc:MGI:1919290]	0.149020158	-2.746420592	0.01000762	1	yes	down
ENSMUSG00000102278	Gm37145	predicted gene, 37145 [Source:MGI Symbol;Acc:MGI:5610373]	0.28036037	-1.834645663	0.0100291	0.04938283	yes	down
ENSMUSG00000028593	Aadacl4fm1	AADACL4 family member 1 [Source:MGI Symbol;Acc:MGI:2685880]	9.426611087	3.236739208	0.01008934	1	yes	up
ENSMUSG00000085337	Gm15964	predicted gene 15964 [Source:MGI Symbol;Acc:MGI:3802003]	0.491935931	-1.023457661	0.0101602	0.04988628	yes	down
ENSMUSG00000070637	Srarp	steroid receptor associated and regulated protein [Source:MGI Symbol;Acc:MGI:2685540]	5.081490656	2.345251774	0.01018645	0.04999727	yes	up
ENSMUSG00000050463	Krt78	keratin 78 [Source:MGI Symbol;Acc:MGI:1917529]	2.395968486	1.260608932	0.01018859	0.04999727	yes	up
ENSMUSG00000118607	Gm7592	predicted gene 7592 [Source:MGI Symbol;Acc:MGI:3644077]	2.114099186	1.080043064	0.01021609	0.0500443	yes	up

ENSMUSG00000100075	1700018L02Rik	RIKEN cDNA 1700018L02 gene [Source:MGI Symbol;Acc:MGI:1914579]	2.689293073	1.427226986	0.01021729	0.0500443	yes	up
ENSMUSG00000070577	Gm572	predicted gene 572 [Source:MGI Symbol;Acc:MGI:2685418]	3.130214582	1.64626156	0.01028215	0.05027634	yes	up
ENSMUSG00000033200	Tpsg1	tryptase gamma 1 [Source:MGI Symbol;Acc:MGI:1349391]	2.158719413	1.110175736	0.01028618	0.05027634	yes	up
ENSMUSG00000115317	Gm32618	predicted gene, 32618 [Source:MGI Symbol;Acc:MGI:5591777]	2.935973578	1.553838985	0.01035893	0.05056211	yes	up
ENSMUSG00000040323	Gm15429	predicted pseudogene 15429 [Source:MGI Symbol;Acc:MGI:3642423]	2.310397079	1.208140824	0.01044642	0.05077249	yes	up
ENSMUSG00000019577	Pdk4	pyruvate dehydrogenase kinase, isoenzyme 4 [Source:MGI Symbol;Acc:MGI:1351481]	2.047275499	1.033705257	0.01046539	0.05082182	yes	up
ENSMUSG00000007594	Hapln4	hyaluronan and proteoglycan link protein 4 [Source:MGI Symbol;Acc:MGI:2679531]	3.593350974	1.845329855	0.01051045	0.05099765	yes	up
ENSMUSG00000029282	Amtn	amelotin [Source:MGI Symbol;Acc:MGI:1918671]	0.307742093	-1.700206305	0.01055115	0.0511234	yes	down
ENSMUSG00000049608	Gpr55	G protein-coupled receptor 55 [Source:MGI Symbol;Acc:MGI:2685064]	2.098283174	1.06920939	0.01063776	0.05139898	yes	up
ENSMUSG00000050511	Oprd1	opioid receptor, delta 1 [Source:MGI Symbol;Acc:MGI:97438]	0.277592448	-1.848959774	0.01068801	0.05158411	yes	down
ENSMUSG00000096674	Mup15	major urinary protein 15 [Source:MGI Symbol;Acc:MGI:3780235]	2.68690085	1.425943085	0.01070299	0.05162761	yes	up
ENSMUSG00000085604	Dhx58os	DEAH (Asp-Glu-Ala-His) box polypeptide 58, opposite strand [Source:MGI Symbol;Acc:MGI:3705104]	2.968519519	1.569743599	0.01076964	0.0518756	yes	up
ENSMUSG00000043531	Sorcs1	sortilin-related VPS10 domain containing receptor 1 [Source:MGI Symbol;Acc:MGI:1929666]	2.144242269	1.100467919	0.01077821	0.0518756	yes	up
ENSMUSG00000028186	Uox	urate oxidase [Source:MGI Symbol;Acc:MGI:98907]	0.433576561	-1.205641327	0.0107838	0.05188707	yes	down
ENSMUSG00000067813	Xkr9	X-linked Kx blood group related 9 [Source:MGI Symbol;Acc:MGI:2686466]	12.16789971	3.605008262	0.0108585	1	yes	up
ENSMUSG00000042031	Lce3b	late cornified envelope 3B [Source:MGI	2.281983915	1.190288622	0.01086468	0.05216367	yes	up
ENSMUSG00000082902	Ccl19-ps1	chemokine (C-C motif) ligand 19, pseudogene 1 [Source:MGI Symbol;Acc:MGI:1891387]	2.440161748	1.286976781	0.01089253	0.05223581	yes	up
ENSMUSG00000066154	Mup3	major urinary protein 3 [Source:MGI Symbol;Acc:MGI:97235]	3.099512408	1.632041279	0.01091646	0.05233602	yes	up
ENSMUSG00000060691	Krtap19-1	keratin associated protein 19-1 [Source:MGI Symbol;Acc:MGI:2157755]	0.045445597	-4.459715676	0.01094317	0.052435	yes	down
ENSMUSG00000045114	Prrt2	proline-rich transmembrane protein 2 [Source:MGI Symbol;Acc:MGI:1916267]	11.10317292	3.472900105	0.01118226	1	yes	up
ENSMUSG00000030303	Far2	fatty acyl CoA reductase 2 [Source:MGI Symbol;Acc:MGI:2687035]	3.850610207	1.945087088	0.01126996	0.05359973	yes	up
ENSMUSG00000089840	Gm4491	predicted gene 4491 [Source:MGI Symbol;Acc:MGI:3782676]	0.049170785	-4.34605481	0.0113672	1	yes	down
ENSMUSG00000035486	Plk5	polo like kinase 5 [Source:MGI Symbol;Acc:MGI:3026984]	3.302943528	1.723752303	0.01146677	0.05435632	yes	up
ENSMUSG00000032591	Mst1	macrophage stimulating 1 (hepatocyte growth factor-like) [Source:MGI Symbol;Acc:MGI:96080]	2.108823262	1.07643819	0.01150456	0.05450555	yes	up
ENSMUSG00000073399	Trim40	tripartite motif-containing 40 [Source:MGI Symbol;Acc:MGI:2684881]	0.109629051	-3.189297939	0.01153926	0.05461614	yes	down
ENSMUSG00000048643	Krtap19-9a	keratin associated protein 19-9A [Source:MGI Symbol;Acc:MGI:3704466]	0.116727336	-3.098785636	0.01156597	0.05470656	yes	down
ENSMUSG00000053168	9030619P08Rik	RIKEN cDNA 9030619P08 gene [Source:MGI Symbol;Acc:MGI:3612405]	6.09038875	2.606534318	0.01160861	1	yes	up
ENSMUSG00000020805	Slc13a5	solute carrier family 13 (sodium-dependent citrate transporter), member 5 [Source:MGI Symbol;Acc:MGI:3037150]	0.108707289	-3.201479414	0.01163319	1	yes	down
ENSMUSG00000118221	Gm50194	predicted gene, 50194 [Source:MGI Symbol;Acc:MGI:6302970]	3.309769996	1.726730964	0.01185042	0.05579249	yes	up
ENSMUSG00000021986	Amer2	APC membrane recruitment 2 [Source:MGI Symbol;Acc:MGI:1919375]	0.372761886	-1.423673738	0.01187726	0.05587324	yes	down
ENSMUSG00000094322	Ighv9-4	immunoglobulin heavy variable 9-4 [Source:MGI Symbol;Acc:MGI:3646379]	9.859736613	3.301549108	0.01192252	0.05604036	yes	up
ENSMUSG00000055546	Timd4	T cell immunoglobulin and mucin domain containing 4 [Source:MGI Symbol;Acc:MGI:2445125]	3.014566536	1.591950572	0.01195023	0.05615537	yes	up

ENSMUSG00000032036	Kirrel3	kirre like nephrin family adhesion molecule 3 [Source:MGI Symbol;Acc:MGI:1914953]	2.791478716	1.481029556	0.01195873	0.05618005	yes	up
ENSMUSG00000046487	Mospd4	motile sperm domain containing 4 [Source:MGI Symbol;Acc:MGI:1919326]	0.052035133	-4.264370155	0.01207203	1	yes	down
ENSMUSG00000118167	Gm50216	predicted gene, 50216 [Source:MGI Symbol;Acc:MGI:6303008]	0.444028524	-1.171275737	0.01226563	0.05721753	yes	down
ENSMUSG00000053693	Mast1	microtubule associated serine/threonine kinase 1 [Source:MGI	0.418643985	-1.256204198	0.01228976	0.05731462	yes	down
ENSMUSG00000045672	Col27a1	collagen, type XXVII, alpha 1 [Source:MGI Symbol;Acc:MGI:2672118]	2.055272095	1.039329403	0.01236178	0.05758346	yes	up
ENSMUSG00000027513	Pck1	phosphoenolpyruvate carboxykinase 1, cytosolic [Source:MGI Symbol;Acc:MGI:97501]	2.205270493	1.140955624	0.01241677	0.05776806	yes	up
ENSMUSG00000109143	Gm10046	predicted gene 10046 [Source:MGI Symbol;Acc:MGI:3797981]	0.286448204	-1.803653801	0.01241706	0.05776806	yes	down
ENSMUSG00000019767	Ccdc170	coiled-coil domain containing 170 [Source:MGI Symbol;Acc:MGI:2685067]	2.334622297	1.223189165	0.01246006	0.05790578	yes	up
ENSMUSG00000070609	Aadacl4	arylacetamide deacetylase like 4 [Source:MGI Symbol;Acc:MGI:3650257]	3.388514307	1.760652863	0.01247677	0.05796786	yes	up
ENSMUSG00000004988	Fxyd4	FXYD domain-containing ion transport regulator 4 [Source:MGI Symbol;Acc:MGI:1889005]	0.38791676	-1.366180985	0.01253872	0.05819094	yes	down
ENSMUSG00000085412	Halr1	Hoxa adjacent long noncoding RNA 1 [Source:MGI Symbol;Acc:MGI:3705267]	2.374415724	1.247572551	0.01263704	0.05853941	yes	up
ENSMUSG00000030638	Sh3gl3	SH3-domain GRB2-like 3 [Source:MGI	2.609518906	1.383783854	0.01267799	0.05866623	yes	up
ENSMUSG00000026764	Kif5c	kinesin family member 5C [Source:MGI	2.210748544	1.144534939	0.01268412	0.0586789	yes	up
ENSMUSG00000039252	Lgi2	leucine-rich repeat LGI family, member 2 [Source:MGI Symbol;Acc:MGI:2180196]	2.163775831	1.113551042	0.01277103	0.05889185	yes	up
ENSMUSG00000068407	Rnase12	ribonuclease, RNase A family, 12 (non-active) [Source:MGI Symbol;Acc:MGI:3528588]	0.309172579	-1.693515727	0.01281014	0.05900923	yes	down
ENSMUSG00000112041	9530020I12Rik	RIKEN cDNA 9530020I12 gene [Source:MGI Symbol;Acc:MGI:1924692]	18.20082183	4.18593169	0.01287596	1	yes	up
ENSMUSG00000087410	2310065F04Rik	RIKEN cDNA 2310065F04 gene [Source:MGI Symbol;Acc:MGI:1921434]	15.0159409	3.908422972	0.01295537	1	yes	up
ENSMUSG00000076576	Igkv6-32	immunoglobulin kappa variable 6-32 [Source:MGI Symbol;Acc:MGI:3641634]	2.366853819	1.242970606	0.01310584	0.06008321	yes	up
ENSMUSG00000081723	Gm15931	predicted gene 15931 [Source:MGI Symbol;Acc:MGI:3805553]	2.160231328	1.111185811	0.01315062	0.06019271	yes	up
ENSMUSG00000071341	Egr4	early growth response 4 [Source:MGI Symbol;Acc:MGI:99252]	0.143406523	-2.801817449	0.01317024	1	yes	down
ENSMUSG00000028524	Sgip1	SH3-domain GRB2-like (endophilin) interacting protein 1 [Source:MGI Symbol;Acc:MGI:1920344]	2.048228937	1.034376979	0.01319225	0.06036728	yes	up
ENSMUSG00000076591	Igkv8-16	immunoglobulin kappa variable 8-16 [Source:MGI Symbol;Acc:MGI:1330843]	0.03320562	-4.912428745	0.01320834	1	yes	down
ENSMUSG00000066101	Gm10153	predicted gene 10153 [Source:MGI Symbol;Acc:MGI:3642359]	0.024877022	-5.329042382	0.01321964	1	yes	down
ENSMUSG00000022025	Cnmd	chondromodulin [Source:MGI Symbol;Acc:MGI:1341171]	4.661896996	2.220917129	0.01335406	0.06091426	yes	up
ENSMUSG00000074589	4930449A18Rik	RIKEN cDNA 4930449A18 gene [Source:MGI Symbol;Acc:MGI:1921922]	4.593907439	2.199721789	0.01341552	0.06109986	yes	up
ENSMUSG00000095213	Gm9944	predicted gene 9944 [Source:MGI Symbol;Acc:MGI:3642412]	2.493512907	1.318179671	0.01345439	0.06125515	yes	up
ENSMUSG00000036123	Slc9a3	solute carrier family 9 (sodium/hydrogen exchanger), member 3 [Source:MGI Symbol;Acc:MGI:105064]	5.084376672	2.346070915	0.01350328	1	yes	up
ENSMUSG00000069307	H2bc23	H2B clustered histone 23 [Source:MGI	4.98055997	2.316307955	0.01357435	0.06170742	yes	up
ENSMUSG00000085184	4933439K11Rik	RIKEN cDNA 4933439K11 gene [Source:MGI Symbol;Acc:MGI:1918569]	0.449575597	-1.153364368	0.01360355	0.06175896	yes	down
ENSMUSG00000025427	Rnf165	ring finger protein 165 [Source:MGI Symbol;Acc:MGI:2444521]	2.166913054	1.115641267	0.01361782	0.06178093	yes	up
ENSMUSG00000078686	Mup9	major urinary protein 9 [Source:MGI Symbol;Acc:MGI:3782918]	2.066413202	1.047128766	0.01361911	0.06178093	yes	up
ENSMUSG00000022306	Zfpm2	zinc finger protein, multitype 2 [Source:MGI Symbol;Acc:MGI:1334444]	2.039848584	1.028462066	0.0136351	0.06180742	yes	up

ENSMUSG00000050395	Tnfsf15	tumor necrosis factor (ligand) superfamily, member 15 [Source:MGI Symbol;Acc:MGI:2180140]	0.363143387	-1.461388785	0.01368646	0.06197263	yes	down
ENSMUSG00000118180	Gm50223	predicted gene, 50223 [Source:MGI Symbol;Acc:MGI:6303020]	0.462796538	-1.111550023	0.01372769	0.06209429	yes	down
ENSMUSG00000089837	Npcd	neuronal pentraxin chromo domain [Source:MGI Symbol;Acc:MGI:3845555]	39.9241005	5.319187999	0.01373908	1	yes	up
ENSMUSG00000025789	St8sia2	ST8 alpha-N-acetyl-neuraminide alpha-2,8-sialyltransferase 2 [Source:MGI	2.283719289	1.191385328	0.01377137	0.06224299	yes	up
ENSMUSG00000109783	Gm45338	predicted gene 45338 [Source:MGI Symbol;Acc:MGI:5791174]	2.084021234	1.059369977	0.01386938	0.06260412	yes	up
ENSMUSG00000021221	Dpf3	D4, zinc and double PHD fingers, family 3 [Source:MGI Symbol;Acc:MGI:1917377]	2.243447488	1.165717415	0.01401522	0.06313056	yes	up
ENSMUSG00000076563	Igkv5-48	immunoglobulin kappa variable 5-48 [Source:MGI Symbol;Acc:MGI:3642817]	2.197347334	1.135762934	0.01407581	0.06333747	yes	up
ENSMUSG00000034028	Cd226	CD226 antigen [Source:MGI Symbol;Acc:MGI:3039602]	2.491247359	1.316868276	0.0141802	0.06369117	yes	up
ENSMUSG00000111737	Gm47248	predicted gene, 47248 [Source:MGI Symbol;Acc:MGI:6096073]	6.208738383	2.634300142	0.01433086	1	yes	up
ENSMUSG00000093894	Ighv1-53	immunoglobulin heavy variable 1-53 [Source:MGI Symbol;Acc:MGI:3576502]	2.152559219	1.106052928	0.01434453	0.06429563	yes	up
ENSMUSG00000098934	Gvin-ps4	GTPase, very large interferon inducible, pseudogene 4 [Source:MGI Symbol;Acc:MGI:5011038]	2.676492242	1.420343471	0.01441336	0.06447519	yes	up
ENSMUSG00000047904	Sstr2	somatostatin receptor 2 [Source:MGI Symbol;Acc:MGI:98328]	0.38102135	-1.392056256	0.0144769	0.06460421	yes	down
ENSMUSG00000024485	Slc4a9	solute carrier family 4, sodium bicarbonate cotransporter, member 9 [Source:MGI Symbol;Acc:MGI:2443384]	0.467342738	-1.097447118	0.01456811	0.06492163	yes	down
ENSMUSG00000031340	Gabre	gamma-aminobutyric acid (GABA) A receptor, subunit epsilon [Source:MGI	0.49432747	-1.016461015	0.01458066	0.06494986	yes	down
ENSMUSG00000087273	Gm13203	predicted gene 13203 [Source:MGI Symbol;Acc:MGI:3651445]	0.416472589	-1.263706551	0.01461959	0.06509564	yes	down
ENSMUSG00000100985	Gm10640	predicted gene 10640 [Source:MGI Symbol;Acc:MGI:3648835]	0.409483629	-1.288122319	0.01462236	0.06509564	yes	down
ENSMUSG00000071005	Ccl19	chemokine (C-C motif) ligand 19 [Source:MGI Symbol;Acc:MGI:1346316]	2.358438153	1.237831768	0.01462468	0.06509564	yes	up
ENSMUSG00000024347	Psd2	pleckstrin and Sec7 domain containing 2 [Source:MGI Symbol;Acc:MGI:1921252]	3.689711172	1.883507887	0.01472028	0.06542012	yes	up
ENSMUSG00000056271	Lman1l	lectin, mannose-binding 1 like [Source:MGI Symbol;Acc:MGI:2667537]	0.209653466	-2.253921411	0.01473914	1	yes	down
ENSMUSG00000038304	Cd160	CD160 antigen [Source:MGI Symbol;Acc:MGI:1860383]	3.598826423	1.847526521	0.01474088	0.06546118	yes	up
ENSMUSG00000037727	Avp	arginine vasopressin [Source:MGI Symbol;Acc:MGI:88121]	0.205908126	-2.279927329	0.01477027	0.06555879	yes	down
ENSMUSG00000038540	Tmc3	transmembrane channel-like gene family 3 [Source:MGI Symbol;Acc:MGI:2669033]	0.383064994	-1.384338901	0.01484082	0.06578077	yes	down
ENSMUSG00000107760	Gm44401	predicted gene, 44401 [Source:MGI Symbol;Acc:MGI:5690793]	0.296684349	-1.752999272	0.01507883	0.06675618	yes	down
ENSMUSG00000044296	Zfp879	zinc finger protein 879 [Source:MGI Symbol;Acc:MGI:3053099]	0.33611495	-1.572973381	0.01528271	0.06740382	yes	down
ENSMUSG00000096719	Mrgpra2b	MAS-related GPR, member A2B [Source:MGI Symbol;Acc:MGI:3033098]	0.237895476	-2.071600257	0.01532164	0.06751987	yes	down
ENSMUSG00000047104	Pbp2	phosphatidylethanolamine binding protein 2 [Source:MGI Symbol;Acc:MGI:1923650]	0.297638228	-1.748368262	0.01533712	0.06753644	yes	down
ENSMUSG00000075324	Fign	fidgetin [Source:MGI Symbol;Acc:MGI:1890647]	2.056394468	1.040117036	0.01542856	0.0678527	yes	up
ENSMUSG00000060560	Ces4a	carboxylesterase 4A [Source:MGI Symbol;Acc:MGI:2384581]	3.684646086	1.881526054	0.0154464	0.06791389	yes	up
ENSMUSG00000050640	Tmem150c	transmembrane protein 150C [Source:MGI Symbol;Acc:MGI:3041258]	2.07814276	1.055294765	0.01546603	0.06794833	yes	up
ENSMUSG00000037953	A4gnt	alpha-1,4-N-acetylglucosaminyltransferase [Source:MGI Symbol;Acc:MGI:2143261]	2.94178379	1.556691218	0.01549157	0.06804325	yes	up
ENSMUSG00000106917	Gm7832	predicted gene 7832 [Source:MGI Symbol;Acc:MGI:3779767]	16.93558787	4.081986161	0.01559502	1	yes	up
ENSMUSG00000076535	Igkv1-88	immunoglobulin kappa chain variable 1-88 [Source:MGI Symbol;Acc:MGI:4439828]	0.172010862	-2.539428426	0.01564164	0.06856303	yes	down

ENSMUSG00000062309	Rpp25	ribonuclease P/MRP 25 subunit [Source:MGI Symbol;Acc:MGI:2143151]	0.293824485	-1.766973473	0.01571168	0.06878288	yes	down
ENSMUSG00000050534	Htr5b	5-hydroxytryptamine (serotonin) receptor 5B [Source:MGI Symbol;Acc:MGI:96284]	0.16586743	-2.591897469	0.01589421	1	yes	down
ENSMUSG00000027869	Hsd3b6	hydroxy-delta-5-steroid dehydrogenase, 3 beta- and steroid delta-isomerase 6 [Source:MGI Symbol;Acc:MGI:109598]	4.143300845	2.050780578	0.01591838	0.06935408	yes	up
ENSMUSG00000103217	Gm38287	predicted gene, 38287 [Source:MGI Symbol;Acc:MGI:5611515]	0.203365519	-2.297853009	0.01595194	0.06944425	yes	down
ENSMUSG00000010492	Uckl1os	uridine-cytidine kinase 1-like 1, opposite strand [Source:MGI Symbol;Acc:MGI:3801877]	2.245372074	1.16695453	0.01595916	0.06944425	yes	up
ENSMUSG00000081752	Sms-ps	spermine synthase, pseudogene [Source:MGI Symbol;Acc:MGI:3705601]	2.081101694	1.057347465	0.01599742	0.06957571	yes	up
ENSMUSG00000026885	Ttll11	tubulin tyrosine ligase-like family, member 11 [Source:MGI Symbol;Acc:MGI:1921660]	2.090951629	1.064159688	0.01602594	0.06965079	yes	up
ENSMUSG00000044703	Phf11a	PHD finger protein 11A [Source:MGI Symbol;Acc:MGI:1918441]	2.808216881	1.489654361	0.01603083	0.0696509	yes	up
ENSMUSG00000107872	Gm44511	predicted gene 44511 [Source:MGI Symbol;Acc:MGI:5753087]	2.712857714	1.439813382	0.01605544	0.06973018	yes	up
ENSMUSG00000117499	Gm50010	predicted gene, 50010 [Source:MGI Symbol;Acc:MGI:6275297]	0.484678471	-1.044900095	0.01605714	0.06973018	yes	down
ENSMUSG00000055833	1700034H15Rik	RIKEN cDNA 1700034H15 gene [Source:MGI Symbol;Acc:MGI:1921515]	0.36707573	-1.445850365	0.01611086	0.06990623	yes	down
ENSMUSG00000018868	Pnpla5	patatin-like phospholipase domain containing 5 [Source:MGI Symbol;Acc:MGI:1923022]	4.053690089	2.019235797	0.01611385	0.06990623	yes	up
ENSMUSG00000105867	Gm42517	predicted gene 42517 [Source:MGI Symbol;Acc:MGI:5662654]	2.795576673	1.483145914	0.01615114	0.07001528	yes	up
ENSMUSG00000067768	Xlr4b	X-linked lymphocyte-regulated 4B [Source:MGI Symbol;Acc:MGI:1350975]	3.185993344	1.671743253	0.01621238	0.07021032	yes	up
ENSMUSG00000059991	Nptx2	neuronal pentraxin 2 [Source:MGI Symbol;Acc:MGI:1858209]	2.482572036	1.31183558	0.01622752	0.07025831	yes	up
ENSMUSG00000024366	Gfra3	glial cell line derived neurotrophic factor family receptor alpha 3 [Source:MGI	2.11164453	1.078366994	0.01626557	0.07040542	yes	up
ENSMUSG00000113415	Gm3234	predicted gene 3234 [Source:MGI Symbol;Acc:MGI:3781412]	5.113685875	2.354363541	0.01632476	1	yes	up
ENSMUSG00000096452	Ighv1-77	immunoglobulin heavy variable 1-77 [Source:MGI Symbol;Acc:MGI:4439670]	2.914776635	1.543385331	0.01640639	0.07083757	yes	up
ENSMUSG00000096805	Ighv9-1	immunoglobulin heavy variable 9-1 [Source:MGI Symbol;Acc:MGI:4439911]	11.16884994	3.481408733	0.01643712	1	yes	up
ENSMUSG00000095127	Ighv1-82	immunoglobulin heavy variable 1-82 [Source:MGI Symbol;Acc:MGI:4439671]	2.202507679	1.139147049	0.01648026	0.0710273	yes	up
ENSMUSG00000028197	Col24a1	collagen, type XXIV, alpha 1 [Source:MGI Symbol;Acc:MGI:1918605]	2.348033664	1.231453093	0.01663343	0.0714609	yes	up
ENSMUSG00000027048	Abcb11	ATP-binding cassette, sub-family B (MDR/TAP), member 11 [Source:MGI	0.323437607	-1.62844066	0.01665218	0.07150592	yes	down
ENSMUSG00000109097	Gm29683	predicted gene, 29683 [Source:MGI Symbol;Acc:MGI:5588842]	23.70878268	4.567349685	0.01687915	1	yes	up
ENSMUSG00000043460	Elfn2	leucine rich repeat and fibronectin type III, extracellular 2 [Source:MGI Symbol;Acc:MGI:3608416]	13.80643097	3.787268519	0.01694618	1	yes	up
ENSMUSG00000031610	Scrg1	scrapie responsive gene 1 [Source:MGI	0.481190423	-1.055320165	0.01705973	0.07285784	yes	down
ENSMUSG00000095565	Ighv2-9-1	immunoglobulin heavy variable 2-9-1 [Source:MGI Symbol;Acc:MGI:4439519]	2.773588125	1.471753565	0.01715973	0.07307414	yes	up
ENSMUSG00000058975	Kcnc1	potassium voltage gated channel, Shaw-related subfamily, member 1 [Source:MGI	2.206195256	1.14156048	0.01716109	0.07307414	yes	up
ENSMUSG00000084819	Gm11967	predicted gene 11967 [Source:MGI Symbol;Acc:MGI:3650297]	17.33053584	4.115244357	0.01725407	1	yes	up
ENSMUSG00000025726	Slc28a1	solute carrier family 28 (sodium-coupled nucleoside transporter), member 1 [Source:MGI Symbol;Acc:MGI:3605073]	5.286032791	2.402185374	0.01734469	0.07363824	yes	up
ENSMUSG00000108950	9130015G15Rik	RIKEN cDNA 9130015G15 gene [Source:MGI Symbol;Acc:MGI:1921817]	0.042057975	-4.571476802	0.01749039	1	yes	down
ENSMUSG00000090527	Aadacl2fm2	AADACL2 family member 2 [Source:MGI Symbol;Acc:MGI:3779495]	3.911450753	1.967703801	0.01753653	0.07430673	yes	up

ENSMUSG00000038298	Pdzk1	PDZ domain containing 1 [Source:MGI	4.276694624	2.096496196	0.01763466	0.07464937	yes	up
ENSMUSG00000096833	Igkv4-55	immunoglobulin kappa variable 4-55 [Source:MGI Symbol;Acc:MGI:2686370]	2.788087667	1.479275925	0.01780056	0.07522271	yes	up
ENSMUSG00000026904	Slc4a10	solute carrier family 4, sodium bicarbonate cotransporter-like, member 10 [Source:MGI Symbol;Acc:MGI:2150150]	16.2634236	4.023559085	0.0178134	1	yes	up
ENSMUSG00000099474	1700097N02Rik	RIKEN cDNA 1700097N02 gene [Source:MGI Symbol;Acc:MGI:1914772]	0.335792626	-1.574357545	0.01793966	0.07562564	yes	down
ENSMUSG00000094525	Trbv12-2	T cell receptor beta, variable 12-2 [Source:MGI	3.016706503	1.592974343	0.01813412	0.07614941	yes	up
ENSMUSG00000034438	Gbp8	guanylate-binding protein 8 [Source:MGI Symbol;Acc:MGI:1923324]	2.152792261	1.10620911	0.01813438	0.07614941	yes	up
ENSMUSG00000106501	Gm42711	predicted gene 42711 [Source:MGI Symbol;Acc:MGI:5662848]	36.13391253	5.175281571	0.01825307	1	yes	up
ENSMUSG00000086746	Gm15222	predicted gene 15222 [Source:MGI Symbol;Acc:MGI:3705297]	0.288445451	-1.793629586	0.01827776	0.07654697	yes	down
ENSMUSG00000104213	Ighd	immunoglobulin heavy constant delta [Source:MGI	2.36813669	1.243752357	0.01830159	0.07662822	yes	up
ENSMUSG00000031189	Aff2	AF4/FMR2 family, member 2 [Source:MGI Symbol;Acc:MGI:1202294]	2.934263721	1.552998541	0.01833641	0.07671583	yes	up
ENSMUSG00000035085	1700020L24Rik	RIKEN cDNA 1700020L24 gene [Source:MGI Symbol;Acc:MGI:1913580]	2.0102912	1.007404498	0.01840941	0.07687485	yes	up
ENSMUSG00000029477	Morn3	MORN repeat containing 3 [Source:MGI Symbol;Acc:MGI:1922140]	0.043136924	-4.534932885	0.01847437	1	yes	down
ENSMUSG00000106517	Gm42634	predicted gene 42634 [Source:MGI Symbol;Acc:MGI:5662771]	0.155075023	-2.688961758	0.01852344	0.07723908	yes	down
ENSMUSG00000086040	Wipf3	WAS/WASL interacting protein family, member 3 [Source:MGI Symbol;Acc:MGI:3044681]	2.014770663	1.010615629	0.01868501	0.07774405	yes	up
ENSMUSG00000021250	Fos	FBJ osteosarcoma oncogene [Source:MGI	2.009522951	1.006853055	0.01871245	0.07782074	yes	up
ENSMUSG00000067597	Dgat2l6	diacylglycerol O-acyltransferase 2-like 6 [Source:MGI Symbol;Acc:MGI:3045268]	3.508466017	1.810840388	0.0187578	0.07791562	yes	up
ENSMUSG00000005364	Il5ra	interleukin 5 receptor, alpha [Source:MGI	2.586158559	1.370810731	0.01897321	0.07858382	yes	up
ENSMUSG00000024105	Themis3	thymocyte selection associated family member 3 [Source:MGI Symbol;Acc:MGI:1921806]	0.15605893	-2.67983718	0.01899508	1	yes	down
ENSMUSG00000026697	Myoc	myocilin [Source:MGI Symbol;Acc:MGI:1202864]	4.633572554	2.212124962	0.01907997	0.07891257	yes	up
ENSMUSG00000002032	Tmem25	transmembrane protein 25 [Source:MGI	2.689591371	1.427387001	0.01936292	0.07960668	yes	up
ENSMUSG00000104252	Pcdha4	protocadherin alpha 4 [Source:MGI Symbol;Acc:MGI:1298406]	8.899960638	3.153798955	0.01938204	1	yes	up
ENSMUSG00000014609	Chrne	cholinergic receptor, nicotinic, epsilon polypeptide [Source:MGI Symbol;Acc:MGI:87894]	3.638976611	1.863532778	0.0194966	1	yes	up
ENSMUSG00000026424	Gpr37l1	G protein-coupled receptor 37-like 1 [Source:MGI Symbol;Acc:MGI:1928503]	2.154423271	1.107301719	0.01951933	0.08005934	yes	up
ENSMUSG00000021922	Itih4	inter alpha-trypsin inhibitor, heavy chain 4 [Source:MGI Symbol;Acc:MGI:109536]	4.733053141	2.24277112	0.01952083	1	yes	up
ENSMUSG00000075571	Defb30	defensin beta 30 [Source:MGI Symbol;Acc:MGI:1920920]	0.041416564	-4.593648329	0.01958923	1	yes	down
ENSMUSG00000031994	Adamts8	a disintegrin-like and metallopeptidase (reprolysin type) with thrombospondin type 1 motif, 8 [Source:MGI	2.086072417	1.060789241	0.01960242	0.08026025	yes	up
ENSMUSG00000049122	Frmd3	FERM domain containing 3 [Source:MGI Symbol;Acc:MGI:2442466]	3.100990132	1.632728935	0.01963928	0.08036069	yes	up
ENSMUSG00000025433	Crisp3	cysteine-rich secretory protein 3 [Source:MGI Symbol;Acc:MGI:102552]	0.049872942	-4.325598872	0.01964835	1	yes	down
ENSMUSG00000066553	Gm6969	predicted pseudogene 6969 [Source:MGI Symbol;Acc:MGI:3645320]	2.135484083	1.094563145	0.01991881	0.081197	yes	up
ENSMUSG00000071265	1700086L19Rik	RIKEN cDNA 1700086L19 gene [Source:MGI Symbol;Acc:MGI:1921534]	15.6881251	3.97160104	0.01993321	1	yes	up
ENSMUSG00000111044	Gm47106	predicted gene, 47106 [Source:MGI Symbol;Acc:MGI:6095842]	0.041229985	-4.600162255	0.02003273	1	yes	down
ENSMUSG00000095700	Ighv10-3	immunoglobulin heavy variable V10-3 [Source:MGI Symbol;Acc:MGI:3648785]	4.238938589	2.083703066	0.02004605	0.08160426	yes	up

ENSMUSG00000009356	Lpo	lactoperoxidase [Source:MGI Symbol;Acc:MGI:1923363]	2.803562851	1.487261413	0.02011383	0.08181313	yes	up
ENSMUSG00000054423	Cadps	Ca2+-dependent secretion activator [Source:MGI Symbol;Acc:MGI:1350922]	4.25421216	2.088891983	0.02012853	0.08182999	yes	up
ENSMUSG00000045915	Ccdc42	coiled-coil domain containing 42 [Source:MGI Symbol;Acc:MGI:3045254]	3.139056506	1.650330999	0.02018537	0.08194074	yes	up
ENSMUSG00000076514	Igkv17-121	immunoglobulin kappa variable 17-121 [Source:MGI Symbol;Acc:MGI:3647671]	2.178691165	1.123461705	0.02031503	0.08234506	yes	up
ENSMUSG00000083994	Gm16465	predicted gene 16465 [Source:MGI Symbol;Acc:MGI:3646326]	0.095556891	-3.387496276	0.02046124	1	yes	down
ENSMUSG00000059852	Kcng2	potassium voltage-gated channel, subfamily G, member 2 [Source:MGI Symbol;Acc:MGI:3694646]	0.475294568	-1.073106181	0.02065096	0.08308443	yes	down
ENSMUSG00000117970	Gm4402	predicted gene 4402 [Source:MGI Symbol;Acc:MGI:3782587]	3.100060382	1.632296316	0.02085758	0.08367574	yes	up
ENSMUSG00000040441	Slc26a10	solute carrier family 26, member 10 [Source:MGI Symbol;Acc:MGI:2143920]	2.062429263	1.044344638	0.02099185	0.08414261	yes	up
ENSMUSG00000020723	Cacng4	calcium channel, voltage-dependent, gamma subunit 4 [Source:MGI Symbol;Acc:MGI:1859167]	0.272153846	-1.877505669	0.02103641	0.08422357	yes	down
ENSMUSG00000102700	Gm38312	predicted gene, 38312 [Source:MGI Symbol;Acc:MGI:5611540]	0.295006161	-1.761183009	0.02105425	0.08427549	yes	down
ENSMUSG00000042540	Acot5	acyl-CoA thioesterase 5 [Source:MGI Symbol;Acc:MGI:2384969]	3.366508035	1.751252908	0.02108479	0.08431967	yes	up
ENSMUSG00000096862	Gm13301	predicted gene 13301 [Source:MGI Symbol;Acc:MGI:3707230]	2.185483305	1.127952357	0.02147007	0.08560297	yes	up
ENSMUSG00000044867	Gimap1os	GTPase, IMAP family member 1, opposite strand [Source:MGI Symbol;Acc:MGI:3781523]	5.69434696	2.5095304	0.02168309	1	yes	up
ENSMUSG00000085517	Gm12963	predicted gene 12963 [Source:MGI Symbol;Acc:MGI:3651569]	2.414540694	1.271748778	0.02185167	0.08672959	yes	up
ENSMUSG00000019761	Krt10	keratin 10 [Source:MGI Symbol;Acc:MGI:96685]	2.691237454	1.428269689	0.02200903	0.08720882	yes	up
ENSMUSG00000097413	A830052D11Rik	RIKEN cDNA A830052D11 gene [Source:MGI Symbol;Acc:MGI:3028074]	4.960970175	2.310622284	0.02207041	1	yes	up
ENSMUSG00000022518	4930562C15Rik	RIKEN cDNA 4930562C15 gene [Source:MGI Symbol;Acc:MGI:1926059]	0.150735671	-2.729907225	0.02208302	1	yes	down
ENSMUSG00000053046	Brsk2	BR serine/threonine kinase 2 [Source:MGI Symbol;Acc:MGI:1923020]	7.449605809	2.897164088	0.02212025	1	yes	up
ENSMUSG00000061356	Nuggc	nuclear GTPase, germinal center associated [Source:MGI Symbol;Acc:MGI:2685446]	2.546767285	1.348667137	0.02214729	0.08757603	yes	up
ENSMUSG00000076596	Igkv3-10	immunoglobulin kappa variable 3-10 [Source:MGI Symbol;Acc:MGI:1330821]	3.628743756	1.859470184	0.02219654	0.08769052	yes	up
ENSMUSG00000027840	Wnt2b	wingless-type MMTV integration site family, member 2B [Source:MGI Symbol;Acc:MGI:1261834]	2.482210346	1.311625377	0.02223339	0.087796	yes	up
ENSMUSG00000024653	Scgb1a1	secretoglobin, family 1A, member 1 (uteroglobin) [Source:MGI Symbol;Acc:MGI:98919]	0.237513266	-2.073920002	0.02231654	0.08808409	yes	down
ENSMUSG00000045215	Asxl3	ASXL transcriptional regulator 3 [Source:MGI Symbol;Acc:MGI:2685175]	2.702495319	1.434292119	0.02245316	0.08848193	yes	up
ENSMUSG00000033419	Snap91	synaptosomal-associated protein 91 [Source:MGI Symbol;Acc:MGI:109132]	2.148894398	1.103594587	0.02248525	0.08856801	yes	up
ENSMUSG00000003411	Rab3b	RAB3B, member RAS oncogene family [Source:MGI Symbol;Acc:MGI:1917158]	0.456059098	-1.132707309	0.02272637	0.08935491	yes	down
ENSMUSG00000084349	Rpl3-ps1	ribosomal protein L3, pseudogene 1 [Source:MGI Symbol;Acc:MGI:3644217]	23.46888119	4.552677162	0.0228628	1	yes	up
ENSMUSG00000079157	Fam155a	family with sequence similarity 155, member A [Source:MGI Symbol;Acc:MGI:2142765]	6.486564721	2.69745463	0.02291772	1	yes	up
ENSMUSG00000095204	Ighv1-52	immunoglobulin heavy variable 1-52 [Source:MGI Symbol;Acc:MGI:4439752]	2.984751503	1.577610824	0.0230367	0.09016491	yes	up
ENSMUSG00000046561	Arsj	arylsulfatase J [Source:MGI Symbol;Acc:MGI:2443513]	2.099798092	1.070250611	0.02304999	0.09019653	yes	up
ENSMUSG00000049100	Pcdh10	protocadherin 10 [Source:MGI Symbol;Acc:MGI:1338042]	2.651179643	1.40663443	0.02305843	0.09020914	yes	up
ENSMUSG00000038347	Tcte2	t-complex-associated testis expressed 2 [Source:MGI	0.420746406	-1.248977148	0.02323327	0.09069406	yes	down
ENSMUSG00000020651	Slc26a4	solute carrier family 26, member 4 [Source:MGI Symbol;Acc:MGI:1346029]	0.098227754	-3.347725484	0.02331798	1	yes	down

ENSMUSG00000114784	Gm47754	predicted gene, 47754 [Source:MGI Symbol;Acc:MGI:6096900]	3.095750577	1.630289239	0.02337417	0.09115556	yes	up
ENSMUSG00000099923	1700105P06Rik	RIKEN cDNA 1700105P06 gene [Source:MGI Symbol;Acc:MGI:1915479]	3.585906986	1.842338067	0.02353971	0.09164262	yes	up
ENSMUSG00000095285	Ighv5-9	immunoglobulin heavy variable 5-9 [Source:MGI Symbol;Acc:MGI:4439873]	4.994227415	2.320261517	0.02355148	0.09166097	yes	up
ENSMUSG00000030680	Pagr1a	PAXIP1 associated glutamate rich protein 1A [Source:MGI Symbol;Acc:MGI:1914528]	0.246545341	-2.020075104	0.02359588	0.09179244	yes	down
ENSMUSG00000055093	Rps27a-ps3	ribosomal protein S27A, pseudogene 3 [Source:MGI Symbol;Acc:MGI:3645406]	17.27170146	4.110338307	0.02392002	1	yes	up
ENSMUSG00000029601	Iqcd	IQ motif containing D [Source:MGI Symbol;Acc:MGI:1922982]	4.869808885	2.283865155	0.02398197	1	yes	up
ENSMUSG00000040966	Slc22a2	solute carrier family 22 (organic cation transporter), member 2 [Source:MGI	0.269092043	-1.893828366	0.02419299	1	yes	down
ENSMUSG00000040759	Cmtm5	CKLF-like MARVEL transmembrane domain containing 5 [Source:MGI Symbol;Acc:MGI:2447164]	2.971523202	1.571202646	0.02423473	0.09377128	yes	up
ENSMUSG00000020990	Cdkl1	cyclin-dependent kinase-like 1 (CDC2-related kinase) [Source:MGI Symbol;Acc:MGI:1918341]	2.772793677	1.47134027	0.02430782	0.09394892	yes	up
ENSMUSG00000036834	Plch1	phospholipase C, eta 1 [Source:MGI Symbol;Acc:MGI:2683547]	0.347855249	-1.523441005	0.02432379	0.09398965	yes	down
ENSMUSG00000042041	2010003K11Rik	RIKEN cDNA 2010003K11 gene [Source:MGI Symbol;Acc:MGI:1917111]	2.172648654	1.119454891	0.02498288	0.09606408	yes	up
ENSMUSG00000108236	0610033M10Rik	RIKEN cDNA 0610033M10 gene [Source:MGI Symbol;Acc:MGI:1921343]	0.164186886	-2.606589196	0.02500179	1	yes	down
ENSMUSG00000071398	2410004P03Rik	RIKEN cDNA 2410004P03 gene [Source:MGI Symbol;Acc:MGI:1920917]	3.405946936	1.768055958	0.02507008	0.09631945	yes	up
ENSMUSG00000005045	Chd5	chromodomain helicase DNA binding protein 5 [Source:MGI Symbol;Acc:MGI:3036258]	2.225485222	1.154119921	0.02507398	0.09631945	yes	up
ENSMUSG00000009580	Odam	odontogenic, ameloblast asssociated [Source:MGI Symbol;Acc:MGI:1916842]	0.332495313	-1.58859409	0.02530266	0.09692831	yes	down
ENSMUSG00000024731	Ms4a10	membrane-spanning 4-domains, subfamily A, member 10 [Source:MGI	0.382403481	-1.38683244	0.02530793	0.09692831	yes	down
ENSMUSG00000054598	9130230L23Rik	RIKEN cDNA 9130230L23 gene [Source:MGI Symbol;Acc:MGI:3041166]	2.863193299	1.517625072	0.02533725	0.09697446	yes	up
ENSMUSG00000094347	Olfr784	olfactory receptor 784 [Source:MGI Symbol;Acc:MGI:3030618]	0.481179228	-1.05535373	0.02541398	0.09724587	yes	down
ENSMUSG00000029123	Stk32b	serine/threonine kinase 32B [Source:MGI Symbol;Acc:MGI:1927552]	2.606878608	1.382323404	0.02549699	0.09741258	yes	up
ENSMUSG00000049685	Cyp2g1	cytochrome P450, family 2, subfamily g, polypeptide 1 [Source:MGI Symbol;Acc:MGI:109612]	4.662837363	2.22120811	0.02575113	0.09801532	yes	up
ENSMUSG00000113259	Gm35190	predicted gene, 35190 [Source:MGI Symbol;Acc:MGI:5594349]	0.225270805	-2.150267741	0.02599938	1	yes	down
ENSMUSG00000025202	Scd3	stearoyl-coenzyme A desaturase 3 [Source:MGI Symbol;Acc:MGI:1353437]	3.444848874	1.784440693	0.026105	0.09910039	yes	up
ENSMUSG00000062296	Trank1	tetratricopeptide repeat and ankyrin repeat containing 1 [Source:MGI Symbol;Acc:MGI:1341834]	2.283447816	1.19121382	0.02611327	0.09911005	yes	up
ENSMUSG00000033520	Idi2	isopentenyl-diphosphate delta isomerase 2 [Source:MGI Symbol;Acc:MGI:2444315]	0.250024273	-1.999859935	0.02619238	0.09926044	yes	down
ENSMUSG00000109675	Nxpe1-ps	neurexophilin and PC-esterase domain family, member 1, pseudogene [Source:MGI Symbol;Acc:MGI:3646632]	2.161077518	1.111750823	0.02625854	0.09944309	yes	up
ENSMUSG00000037341	Slc9a7	solute carrier family 9 (sodium/hydrogen exchanger), member 7 [Source:MGI Symbol;Acc:MGI:2444530]	2.219334227	1.150126951	0.02631548	0.0995639	yes	up
ENSMUSG00000036815	Dpp10	dipeptidylpeptidase 10 [Source:MGI Symbol;Acc:MGI:2442409]	3.095621275	1.63022898	0.02635051	0.09961233	yes	up
ENSMUSG00000087341	0610040F04Rik	RIKEN cDNA 0610040F04 gene [Source:MGI Symbol;Acc:MGI:1922644]	0.105400762	-3.2460428	0.02644409	1	yes	down
ENSMUSG00000033213	AA467197	expressed sequence AA467197 [Source:MGI Symbol;Acc:MGI:3034182]	0.415778628	-1.266112491	0.02664199	0.10047705	yes	down
ENSMUSG00000046999	1110032F04Rik	RIKEN cDNA 1110032F04 gene [Source:MGI Symbol;Acc:MGI:1915975]	5.52265279	2.465361428	0.02664616	1	yes	up

ENSMUSG00000000889	Dbh	dopamine beta hydroxylase [Source:MGI	22.22135548	4.473874917	0.0266583	1	yes	up
ENSMUSG00000105376	Gm36535	predicted gene, 36535 [Source:MGI Symbol;Acc:MGI:5595694]	3.839629812	1.940967224	0.02690189	1	yes	up
ENSMUSG00000037169	Mycn	v-myc avian myelocytomatosis viral related oncogene, neuroblastoma derived [Source:MGI Symbol;Acc:MGI:97357]	0.469989494	-1.089299586	0.02693809	0.10119196	yes	down
ENSMUSG00000107060	Gm38901	predicted gene, 38901 [Source:MGI Symbol;Acc:MGI:5621786]	3.93158398	1.975110671	0.02694428	0.10119196	yes	up
ENSMUSG00000076666	Ighv14-4	immunoglobulin heavy variable 14-4 [Source:MGI Symbol;Acc:MGI:4439765]	9.89310742	3.306423742	0.02695089	0.10119196	yes	up
ENSMUSG00000046240	Hepacam	hepatocyte cell adhesion molecule [Source:MGI Symbol;Acc:MGI:1920177]	4.08386972	2.029936843	0.02705094	1	yes	up
ENSMUSG00000095630	Igkv6-23	immunoglobulin kappa variable 6-23 [Source:MGI Symbol;Acc:MGI:3711980]	3.615793561	1.854312311	0.02718015	0.10188431	yes	up
ENSMUSG00000073408	Mucl3	mucin like 3 [Source:MGI Symbol;Acc:MGI:2685476]	0.099391013	-3.330740777	0.02719624	1	yes	down
ENSMUSG00000085543	Gm13568	predicted gene 13568 [Source:MGI Symbol;Acc:MGI:3702049]	0.491267691	-1.025418733	0.02739191	0.10251026	yes	down
ENSMUSG00000027261	Hao1	hydroxyacid oxidase 1, liver [Source:MGI	0.147173435	-2.764410805	0.02739496	1	yes	down
ENSMUSG00000046215	Rprml	reprimo-like [Source:MGI Symbol;Acc:MGI:2144486]	0.364483537	-1.456074444	0.02759549	0.10312814	yes	down
ENSMUSG00000054626	Xlr	X-linked lymphocyte-regulated [Source:MGI	2.718965948	1.443058083	0.02761998	0.10317508	yes	up
ENSMUSG00000017453	Pipox	pipecolic acid oxidase [Source:MGI Symbol;Acc:MGI:1197006]	3.87467217	1.954074251	0.02778612	1	yes	up
ENSMUSG00000111977	Gm47163	predicted gene, 47163 [Source:MGI Symbol;Acc:MGI:6095939]	2.00688294	1.004956468	0.02784761	0.1038543	yes	up
ENSMUSG00000076586	Igkv8-21	immunoglobulin kappa variable 8-21 [Source:MGI Symbol;Acc:MGI:1330840]	2.003884604	1.002799432	0.02795814	0.10407844	yes	up
ENSMUSG00000026807	Ak8	adenylate kinase 8 [Source:MGI Symbol;Acc:MGI:1916120]	2.25563024	1.17353059	0.02803584	0.10430031	yes	up
ENSMUSG00000117485	Gm19696	predicted gene, 19696 [Source:MGI Symbol;Acc:MGI:5011881]	0.190404276	-2.392862217	0.02807186	1	yes	down
ENSMUSG00000051726	Kcnf1	potassium voltage-gated channel, subfamily F, member 1 [Source:MGI Symbol;Acc:MGI:2687399]	0.402774377	-1.311956187	0.02817728	0.10460687	yes	down
ENSMUSG00000097373	Gm26877	predicted gene, 26877 [Source:MGI Symbol;Acc:MGI:5477371]	0.251476746	-1.991503092	0.02841975	0.10516799	yes	down
ENSMUSG00000076556	Igkv4-57	immunoglobulin kappa variable 4-57 [Source:MGI Symbol;Acc:MGI:2685035]	3.470943264	1.795327783	0.02842115	0.10516799	yes	up
ENSMUSG00000031727	Pmfbp1	polyamine modulated factor 1 binding protein 1 [Source:MGI Symbol;Acc:MGI:1930136]	7.604781326	2.926906764	0.02853976	1	yes	up
ENSMUSG00000040328	Olfr56	olfactory receptor 56 [Source:MGI Symbol;Acc:MGI:1333785]	2.308416054	1.20690327	0.02857656	0.10560224	yes	up
ENSMUSG00000048126	Col6a3	collagen, type VI, alpha 3 [Source:MGI	2.582617476	1.368833975	0.0286827	0.10584131	yes	up
ENSMUSG00000044951	Mylk4	myosin light chain kinase family, member 4 [Source:MGI Symbol;Acc:MGI:3643758]	4.498509328	2.169447014	0.02871757	0.10588442	yes	up
ENSMUSG00000005089	Slc1a2	solute carrier family 1 (glial high affinity glutamate transporter), member 2 [Source:MGI Symbol;Acc:MGI:101931]	3.831471398	1.937898536	0.02879142	0.106084	yes	up
ENSMUSG00000118157	Gm9902	predicted gene 9902 [Source:MGI Symbol;Acc:MGI:3642122]	0.463404421	-1.109656287	0.02882451	0.10618327	yes	down
ENSMUSG00000040680	Kremen2	kringle containing transmembrane protein 2 [Source:MGI Symbol;Acc:MGI:1920266]	0.469497103	-1.090811838	0.0290121	0.10664706	yes	down
ENSMUSG00000107742	9530085L11Rik	RIKEN cDNA 9530085L11 gene [Source:MGI Symbol;Acc:MGI:2443889]	0.426071213	-1.230833514	0.02905382	0.10677774	yes	down
ENSMUSG00000049493	Pls1	plastin 1 (I-isoform) [Source:MGI Symbol;Acc:MGI:104809]	2.673903164	1.418947219	0.02921382	0.10718346	yes	up
ENSMUSG00000108470	Gm4598	predicted gene 4598 [Source:MGI Symbol;Acc:MGI:3782781]	2.919906134	1.545921992	0.02939555	0.10766744	yes	up
ENSMUSG00000073008	Gpr174	G protein-coupled receptor 174 [Source:MGI Symbol;Acc:MGI:2685222]	2.411280558	1.269799522	0.02940364	0.10767426	yes	up
ENSMUSG00000084946	Dlx1as	distal-less homeobox 1, antisense [Source:MGI Symbol;Acc:MGI:1195983]	0.140381614	-2.832574103	0.0295707	1	yes	down
ENSMUSG00000015665	Awat1	acyl-CoA wax alcohol acyltransferase 1 [Source:MGI Symbol;Acc:MGI:3588200]	3.674959174	1.877728223	0.02963186	0.10823485	yes	up

ENSMUSG00000033316	Galnt9	polypeptide N-acetylgalactosaminyltransferase 9 [Source:MGI Symbol;Acc:MGI:2677965]	2.368647756	1.24406367	0.02975496	0.10852398	yes	up
ENSMUSG00000076436	Oxct2a	3-oxoacid CoA transferase 2A [Source:MGI Symbol;Acc:MGI:1891061]	0.04728707	-4.402410446	0.02989572	1	yes	down
ENSMUSG00000097093	C330013E15Rik	RIKEN cDNA C330013E15 gene [Source:MGI Symbol;Acc:MGI:1925695]	2.731309262	1.449592677	0.02993861	0.10889516	yes	up
ENSMUSG00000022847	Thpo	thrombopoietin [Source:MGI Symbol;Acc:MGI:101875]	3.274763132	1.711390559	0.03007415	1	yes	up
ENSMUSG00000021587	Pcsk1	proprotein convertase subtilisin/kexin type 1 [Source:MGI Symbol;Acc:MGI:97511]	0.440081589	-1.184157078	0.03008064	0.10918205	yes	down
ENSMUSG00000032890	Rims3	regulating synaptic membrane exocytosis 3 [Source:MGI Symbol;Acc:MGI:2443331]	2.035101586	1.025100811	0.03054923	0.11030389	yes	up
ENSMUSG00000115422	4930452G13Rik	RIKEN cDNA 4930452G13 gene [Source:MGI Symbol;Acc:MGI:1921239]	0.131281233	-2.929267402	0.03060344	1	yes	down
ENSMUSG00000105285	Gm43238	predicted gene 43238 [Source:MGI Symbol;Acc:MGI:5663375]	30.13688338	4.913458323	0.03063053	1	yes	up
ENSMUSG00000042529	Kcnj12	potassium inwardly-rectifying channel, subfamily J, member 12 [Source:MGI	2.084415858	1.059643136	0.03073824	0.11087057	yes	up
ENSMUSG00000000791	Il12rb1	interleukin 12 receptor, beta 1 [Source:MGI Symbol;Acc:MGI:104579]	2.205728901	1.141255485	0.03076845	0.11095639	yes	up
ENSMUSG00000113386	Gm47357	predicted gene, 47357 [Source:MGI Symbol;Acc:MGI:6096262]	0.345616116	-1.532757604	0.03078436	0.11099061	yes	down
ENSMUSG00000026831	1700007K13Rik	RIKEN cDNA 1700007K13 gene [Source:MGI Symbol;Acc:MGI:1916577]	0.490590366	-1.027409191	0.03079627	0.11101042	yes	down
ENSMUSG00000085636	Gm11769	predicted gene 11769 [Source:MGI Symbol;Acc:MGI:3702101]	2.708567691	1.437530146	0.03083267	0.11109529	yes	up
ENSMUSG00000092368	A930015D03Rik	RIKEN cDNA A930015D03 gene [Source:MGI Symbol;Acc:MGI:1925060]	2.605077168	1.381326109	0.03087848	0.11119262	yes	up
ENSMUSG00000024502	Jakmip2	janus kinase and microtubule interacting protein 2 [Source:MGI Symbol;Acc:MGI:1923467]	0.317892633	-1.653388511	0.03101018	0.11147676	yes	down
ENSMUSG00000109279	Gm45220	predicted gene 45220 [Source:MGI Symbol;Acc:MGI:5753796]	2.773539016	1.47172802	0.03118374	0.11191707	yes	up
ENSMUSG00000072573	Gm10369	predicted gene 10369 [Source:MGI Symbol;Acc:MGI:3642774]	6.744260658	2.753660296	0.03136636	1	yes	up
ENSMUSG00000045326	Fndc7	fibronectin type III domain containing 7 [Source:MGI Symbol;Acc:MGI:2443535]	3.803369863	1.927278242	0.03159593	1	yes	up
ENSMUSG00000045052	Prlhr	prolactin releasing hormone receptor [Source:MGI Symbol;Acc:MGI:2135956]	0.050441719	-4.309238751	0.03166969	1	yes	down
ENSMUSG00000093637	Gm20636	predicted gene 20636 [Source:MGI Symbol;Acc:MGI:5313083]	0.049696459	-4.330713132	0.03169083	1	yes	down
ENSMUSG00000073010	Gm5127	predicted gene 5127 [Source:MGI Symbol;Acc:MGI:3648285]	6.399433436	2.677944184	0.03180984	1	yes	up
ENSMUSG00000045613	Chrm2	cholinergic receptor, muscarinic 2, cardiac [Source:MGI Symbol;Acc:MGI:88397]	4.202149701	2.071127559	0.03198371	1	yes	up
ENSMUSG00000109561	Ankrd31	ankyrin repeat domain 31 [Source:MGI	21.79598782	4.445990685	0.03212813	1	yes	up
ENSMUSG00000078722	Gm12394	predicted gene 12394 [Source:MGI Symbol;Acc:MGI:3649790]	2.476607501	1.308365245	0.03216364	0.11413074	yes	up
ENSMUSG00000038916	Soga3	SOGA family member 3 [Source:MGI Symbol;Acc:MGI:1914662]	3.311635892	1.72754406	0.03222576	0.11428571	yes	up
ENSMUSG00000013415	Igf2bp1	insulin-like growth factor 2 mRNA binding protein 1 [Source:MGI Symbol;Acc:MGI:1890357]	0.276653408	-1.853848399	0.03223714	1	yes	down
ENSMUSG00000034833	Tespa1	thymocyte expressed, positive selection associated 1 [Source:MGI Symbol;Acc:MGI:1914846]	2.019678593	1.014125724	0.03241628	0.11476812	yes	up
ENSMUSG00000103527	Gm37261	predicted gene, 37261 [Source:MGI Symbol;Acc:MGI:5610489]	3.035620975	1.601991668	0.03302909	0.1162947	yes	up
ENSMUSG00000023885	Thbs2	thrombospondin 2 [Source:MGI Symbol;Acc:MGI:98738]	2.594954657	1.37570933	0.03307711	0.11644005	yes	up
ENSMUSG00000113794	Gm36607	predicted gene, 36607 [Source:MGI Symbol;Acc:MGI:5595766]	0.170914673	-2.548651833	0.03327874	1	yes	down
ENSMUSG00000118433	Gm50471	predicted gene, 50471 [Source:MGI Symbol;Acc:MGI:6324741]	9.39058956	3.231215736	0.03358641	1	yes	up
ENSMUSG00000041596	Nlrp5-ps	NLR family, pyrin domain containing 5, pseudogene [Source:MGI Symbol;Acc:MGI:5010941]	3.640393079	1.864094237	0.03362652	1	yes	up
ENSMUSG00000044303	Cdkn2a	cyclin dependent kinase inhibitor 2A [Source:MGI Symbol;Acc:MGI:104738]	2.070487925	1.049970789	0.0336504	0.11778661	yes	up

ENSMUSG00000055775	Myh8	myosin, heavy polypeptide 8, skeletal muscle, perinatal [Source:MGI Symbol;Acc:MGI:1339712]	2.198219038	1.136335148	0.0336841	0.11788072	yes	up
ENSMUSG00000034117	Ptgdr2	prostaglandin D2 receptor 2 [Source:MGI Symbol;Acc:MGI:1330275]	2.803140621	1.48704412	0.03377876	0.11814025	yes	up
ENSMUSG00000076522	Igkv16-104	immunoglobulin kappa variable 16-104 [Source:MGI Symbol;Acc:MGI:2685913]	2.409135025	1.268515255	0.03408782	0.11893245	yes	up
ENSMUSG00000026416	Il20	interleukin 20 [Source:MGI Symbol;Acc:MGI:1890473]	6.714337541	2.747245066	0.03413844	1	yes	up
ENSMUSG00000081778	Gm6325	predicted gene 6325 [Source:MGI Symbol;Acc:MGI:3644961]	0.096858432	-3.36797854	0.03422867	1	yes	down
ENSMUSG00000059657	Stfa2l1	stefin A2 like 1 [Source:MGI Symbol;Acc:MGI:3524944]	0.422974435	-1.241357628	0.03424367	0.11933542	yes	down
ENSMUSG00000026100	Mstn	myostatin [Source:MGI Symbol;Acc:MGI:95691]	2.193203721	1.133039826	0.03444019	0.119751	yes	up
ENSMUSG00000070858	Gm1673	predicted gene 1673 [Source:MGI Symbol;Acc:MGI:2686519]	2.059566561	1.042340752	0.0344856	0.1198125	yes	up
ENSMUSG00000063632	Sox11	SRY (sex determining region Y)-box 11 [Source:MGI	2.006490216	1.004674121	0.03451648	0.11989569	yes	up
ENSMUSG00000107230	Gm19265	predicted gene, 19265 [Source:MGI Symbol;Acc:MGI:5011450]	0.413212863	-1.275042931	0.03462386	0.12017211	yes	down
ENSMUSG00000039099	Wdr93	WD repeat domain 93 [Source:MGI Symbol;Acc:MGI:3646885]	2.611265124	1.384748943	0.03475484	0.12050576	yes	up
ENSMUSG00000075605	Slurp2	secreted Ly6/Plaur domain containing 2 [Source:MGI Symbol;Acc:MGI:1916712]	2.24754396	1.168349334	0.03478476	0.1205853	yes	up
ENSMUSG00000070337	Gpr179	G protein-coupled receptor 179 [Source:MGI Symbol;Acc:MGI:2443409]	0.396303713	-1.335321612	0.03479721	0.12060428	yes	down
ENSMUSG00000049555	Tmie	transmembrane inner ear [Source:MGI	2.205714279	1.141245921	0.03486864	0.12075503	yes	up
ENSMUSG00000034115	Scn11a	sodium channel, voltage-gated, type XI, alpha [Source:MGI Symbol;Acc:MGI:1345149]	4.926088984	2.300442689	0.03503547	1	yes	up
ENSMUSG00000073805	Insyn2a	inhibitory synaptic factor 2A [Source:MGI Symbol;Acc:MGI:3605068]	0.286429257	-1.80374923	0.03510135	1	yes	down
ENSMUSG00000026188	Tmem169	transmembrane protein 169 [Source:MGI Symbol;Acc:MGI:2442781]	5.494218271	2.457914226	0.03512729	1	yes	up
ENSMUSG00000107666	4933406J09Rik	RIKEN cDNA 4933406J09 gene [Source:MGI Symbol;Acc:MGI:1921314]	0.483111848	-1.04957086	0.03516689	0.12149585	yes	down
ENSMUSG00000027895	Kcnc4	potassium voltage gated channel, Shaw-related subfamily, member 4 [Source:MGI	2.297059425	1.19978818	0.03534144	0.12193384	yes	up
ENSMUSG00000042262	Ccr8	chemokine (C-C motif) receptor 8 [Source:MGI Symbol;Acc:MGI:1201402]	7.753818564	2.954906977	0.03544171	1	yes	up
ENSMUSG00000024155	Meiob	meiosis specific with OB domains [Source:MGI Symbol;Acc:MGI:1922428]	5.23238088	2.387467563	0.03550207	1	yes	up
ENSMUSG00000096461	Igkv14-130	immunoglobulin kappa variable 14-130 [Source:MGI Symbol;Acc:MGI:3645770]	10.91197291	3.447840063	0.03579151	1	yes	up
ENSMUSG00000052854	Nrk	Nik related kinase [Source:MGI Symbol;Acc:MGI:1351326]	2.183347259	1.126541608	0.03601832	0.12364667	yes	up
ENSMUSG00000035681	Kcnc2	potassium voltage gated channel, Shaw-related subfamily, member 2 [Source:MGI	2.576905506	1.365639635	0.03603969	0.12369547	yes	up
ENSMUSG00000094940	Ighv1-84	immunoglobulin heavy variable 1-84 [Source:MGI Symbol;Acc:MGI:3644235]	4.817351213	2.268240109	0.03610115	1	yes	up
ENSMUSG00000086763	Plxna4os1	plexin A4, opposite strand 1 [Source:MGI Symbol;Acc:MGI:2442851]	4.446079827	2.152533851	0.03612134	1	yes	up
ENSMUSG00000104648	Gm42570	predicted gene 42570 [Source:MGI Symbol;Acc:MGI:5662707]	0.364046963	-1.45780352	0.03615086	0.12400319	yes	down
ENSMUSG00000111147	Gm33699	predicted gene, 33699 [Source:MGI Symbol;Acc:MGI:5592858]	2.918578067	1.545265658	0.0362303	0.12417713	yes	up
ENSMUSG00000045532	C1ql1	complement component 1, q subcomponent-like 1 [Source:MGI Symbol;Acc:MGI:1344400]	2.935020093	1.55337038	0.03632711	1	yes	up
ENSMUSG00000094051	Ighv1-36	immunoglobulin heavy variable 1-36 [Source:MGI Symbol;Acc:MGI:4439639]	0.070655122	-3.823062034	0.03643132	1	yes	down
ENSMUSG00000112963	Gm6093	predicted gene 6093 [Source:MGI Symbol;Acc:MGI:3648840]	2.591647139	1.373869304	0.03646978	0.12487414	yes	up
ENSMUSG00000024565	Sall3	spalt like transcription factor 3 [Source:MGI Symbol;Acc:MGI:109295]	0.051833406	-4.269973994	0.03662734	1	yes	down
ENSMUSG00002076072	5S_rRNA	5S ribosomal RNA [Source:RFAM;Acc:RF00001]	3.763278785	1.911990169	0.0367704	1	yes	up

ENSMUSG00000071478	H2ac7	H2A clustered histone 7 [Source:MGI Symbol;Acc:MGI:2448289]	0.424245466	-1.237028853	0.03679892	0.12564864	yes	down
ENSMUSG00000013936	Myl2	myosin, light polypeptide 2, regulatory, cardiac, slow [Source:MGI Symbol;Acc:MGI:97272]	4.785082193	2.258543706	0.03698077	0.12612421	yes	up
ENSMUSG00000028354	Fmn2	formin 2 [Source:MGI Symbol;Acc:MGI:1859252]	2.157374272	1.109276484	0.03704099	0.12622998	yes	up
ENSMUSG00000085286	Ube4bos3	ubiquitination factor E4B, opposite strand 3 [Source:MGI Symbol;Acc:MGI:1921560]	0.240023269	-2.058753818	0.03716159	1	yes	down
ENSMUSG00000108064	Gm44423	predicted gene, 44423 [Source:MGI Symbol;Acc:MGI:5690815]	2.7223279	1.444840847	0.03718519	0.12667145	yes	up
ENSMUSG00000059654	Reg1	regenerating islet-derived 1 [Source:MGI	2.060358799	1.042895596	0.03725529	0.12673542	yes	up
ENSMUSG00000077668	Gm26143	predicted gene, 26143 [Source:MGI Symbol;Acc:MGI:5455920]	0.078500898	-3.671147034	0.03764435	1	yes	down
ENSMUSG00000032179	Bmp5	bone morphogenetic protein 5 [Source:MGI	2.214214586	1.146795045	0.03774269	0.12799653	yes	up
ENSMUSG00000048385	Scrt1	scratch family zinc finger 1 [Source:MGI Symbol;Acc:MGI:2176606]	3.472747551	1.796077539	0.03780538	1	yes	up
ENSMUSG00000056073	Grik2	glutamate receptor, ionotropic, kainate 2 (beta 2) [Source:MGI Symbol;Acc:MGI:95815]	2.052393531	1.037307383	0.0378775	0.12831988	yes	up
ENSMUSG00000025333	Gpr143	G protein-coupled receptor 143 [Source:MGI Symbol;Acc:MGI:107193]	0.204930423	-2.286793922	0.03791021	0.12835715	yes	down
ENSMUSG00000097281	Gm26685	predicted gene, 26685 [Source:MGI Symbol;Acc:MGI:5477179]	0.079053688	-3.661023416	0.03809924	1	yes	down
ENSMUSG00000030200	Bcl2l14	BCL2-like 14 (apoptosis facilitator) [Source:MGI Symbol;Acc:MGI:1914063]	0.447815802	-1.159022657	0.0381234	0.12897795	yes	down
ENSMUSG00000045318	Adra2c	adrenergic receptor, alpha 2c [Source:MGI	2.656005018	1.409257872	0.03838622	0.129696	yes	up
ENSMUSG00000042498	Radx	RPA1 related single stranded DNA binding protein, X-linked [Source:MGI	4.428075759	2.146679905	0.038815	1	yes	up
ENSMUSG00000034774	Dsg1c	desmoglein 1 gamma [Source:MGI Symbol;Acc:MGI:2664358]	2.75042476	1.459654438	0.03896588	0.13090598	yes	up
ENSMUSG00000086480	Gm15287	predicted gene 15287 [Source:MGI Symbol;Acc:MGI:3705156]	0.371069632	-1.430238159	0.03905039	0.13113891	yes	down
ENSMUSG00000028584	Lrrc38	leucine rich repeat containing 38 [Source:MGI Symbol;Acc:MGI:2442845]	2.465164657	1.301684013	0.03910392	0.13124219	yes	up
ENSMUSG00000041293	Adgrf1	adhesion G protein-coupled receptor F1 [Source:MGI Symbol;Acc:MGI:1924846]	0.30259815	-1.724524926	0.03913856	1	yes	down
ENSMUSG00000027345	4921508D12Rik	RIKEN cDNA 4921508D12 gene [Source:MGI Symbol;Acc:MGI:1918095]	4.16290665	2.057591208	0.03938557	1	yes	up
ENSMUSG00000113757	Gm47507	predicted gene, 47507 [Source:MGI Symbol;Acc:MGI:6096493]	0.374445032	-1.417174145	0.03958429	0.13251999	yes	down
ENSMUSG00000097636	Mirt1	myocardial infarction associated transcript 1 [Source:MGI Symbol;Acc:MGI:1922001]	0.479488426	-1.060432103	0.03974655	0.13293449	yes	down
ENSMUSG00000024578	Il17b	interleukin 17B [Source:MGI Symbol;Acc:MGI:1928397]	0.39660362	-1.33423025	0.04002413	0.13355281	yes	down
ENSMUSG00000078672	Mup20	major urinary protein 20 [Source:MGI Symbol;Acc:MGI:3651981]	5.189204131	2.375513289	0.04022369	0.13414103	yes	up
ENSMUSG00000021335	Slc17a1	solute carrier family 17 (sodium phosphate), member 1 [Source:MGI Symbol;Acc:MGI:103209]	0.111046788	-3.170760428	0.04029886	1	yes	down
ENSMUSG00000032087	Dscaml1	DS cell adhesion molecule like 1 [Source:MGI Symbol;Acc:MGI:2150309]	0.34049994	-1.55427355	0.04034479	0.13446708	yes	down
ENSMUSG00000096879	Tdpoz8	TD and POZ domain containing 8 [Source:MGI Symbol;Acc:MGI:3645677]	0.129378433	-2.95033095	0.04060516	1	yes	down
ENSMUSG00000086822	5330413P13Rik	RIKEN cDNA 5330413P13 gene [Source:MGI Symbol;Acc:MGI:3041161]	0.074124119	-3.753913144	0.04086972	1	yes	down
ENSMUSG00000040963	Asgr2	asialoglycoprotein receptor 2 [Source:MGI	2.096886188	1.068248559	0.04097693	0.13580512	yes	up
ENSMUSG00000079492	Gm11127	predicted gene 11127 [Source:MGI Symbol;Acc:MGI:3779381]	2.157503472	1.109362881	0.04103698	0.13583289	yes	up
ENSMUSG00000116617	Gm49767	predicted gene, 49767 [Source:MGI Symbol;Acc:MGI:6215277]	0.470675838	-1.087194302	0.04103744	0.13583289	yes	down
ENSMUSG00000086144	Gm11379	predicted gene 11379 [Source:MGI Symbol;Acc:MGI:3650783]	12.86319155	3.685176737	0.0410726	1	yes	up
ENSMUSG00000099338	2810030D12Rik	RIKEN cDNA 2810030D12 gene [Source:MGI Symbol;Acc:MGI:1925585]	0.266325912	-1.908735293	0.04110754	0.13593481	yes	down
ENSMUSG00000026679	Enkur	enkurin, TRPC channel interacting protein [Source:MGI Symbol;Acc:MGI:1918483]	3.512332928	1.812429602	0.04135515	1	yes	up

ENSMUSG00000112704	Gm46329	predicted gene, 46329 [Source:MGI Symbol;Acc:MGI:5825966]	19.90323897	4.314931323	0.0413687	1	yes	up
ENSMUSG00000032346	Ooep	oocyte expressed protein [Source:MGI	3.262108648	1.705804833	0.04142018	0.13657678	yes	up
ENSMUSG00000097624	Gm5091	predicted gene 5091 [Source:MGI Symbol;Acc:MGI:3779456]	18.40686949	4.202172379	0.04149421	1	yes	up
ENSMUSG00000115546	Gm49077	predicted gene, 49077 [Source:MGI Symbol;Acc:MGI:6118462]	18.40686949	4.202172379	0.04149421	1	yes	up
ENSMUSG00000044689	Gm13749	predicted gene 13749 [Source:MGI Symbol;Acc:MGI:3649721]	18.39827861	4.201498885	0.04153514	1	yes	up
ENSMUSG00000108120	9930120I10Rik	RIKEN cDNA 9930120I10 gene [Source:MGI Symbol;Acc:MGI:1924996]	18.39827861	4.201498885	0.04153514	1	yes	up
ENSMUSG00000089781	Gm15756	predicted gene 15756 [Source:MGI Symbol;Acc:MGI:3783199]	2.67846913	1.421408669	0.04153889	0.13686379	yes	up
ENSMUSG00000020542	Myocd	myocardin [Source:MGI Symbol;Acc:MGI:2137495]	2.162843549	1.11292931	0.0415688	0.13691015	yes	up
ENSMUSG00000035186	Ubd	ubiquitin D [Source:MGI Symbol;Acc:MGI:1344410]	2.273374112	1.184835117	0.04186364	0.13767142	yes	up
ENSMUSG00000074758	Gm5535	predicted gene 5535 [Source:MGI Symbol;Acc:MGI:3645019]	3.33468173	1.737549074	0.04197389	1	yes	up
ENSMUSG00000048905	Bnip5	BCL2 interacting protein 5 [Source:MGI Symbol;Acc:MGI:1925441]	2.822926019	1.49719132	0.04203963	0.13809255	yes	up
ENSMUSG00000103711	Tstd1	thiosulfate sulfurtransferase (rhodanese)-like domain containing 1 [Source:MGI Symbol;Acc:MGI:3648482]	0.082795695	-3.594300427	0.04208039	1	yes	down
ENSMUSG00000026532	Spta1	spectrin alpha, erythrocytic 1 [Source:MGI	2.283089157	1.190987199	0.04222353	0.13845985	yes	up
ENSMUSG00000059213	Ddn	dendrin [Source:MGI Symbol;Acc:MGI:108101]	2.796661287	1.483705535	0.04227956	0.13858662	yes	up
ENSMUSG00000031220	Awat2	acyl-CoA wax alcohol acyltransferase 2 [Source:MGI Symbol;Acc:MGI:3045345]	2.941532606	1.556568028	0.04237657	0.13885635	yes	up
ENSMUSG00000044309	Apol7c	apolipoprotein L 7c [Source:MGI Symbol;Acc:MGI:1920912]	2.193357921	1.133141256	0.04239324	0.13888466	yes	up
ENSMUSG00000024786	Majin	membrane anchored junction protein [Source:MGI Symbol;Acc:MGI:1923913]	18.21554667	4.187098387	0.04241021	1	yes	up
ENSMUSG00000083907	Plk-ps1	polo like kinase, pseudogene 1 [Source:MGI Symbol;Acc:MGI:103247]	0.057724018	-4.11468447	0.04252702	1	yes	down
ENSMUSG00000113599	9530014B07Rik	RIKEN cDNA 9530014B07 gene [Source:MGI Symbol;Acc:MGI:1924648]	0.213375382	-2.228534356	0.04257158	1	yes	down
ENSMUSG00000045288	Ush1g	USH1 protein network component sans [Source:MGI Symbol;Acc:MGI:2450757]	0.263485986	-1.924201861	0.04289927	1	yes	down
ENSMUSG00000048528	Nkx1-2	NK1 homeobox 2 [Source:MGI Symbol;Acc:MGI:104806]	0.099989132	-3.32208489	0.04294724	1	yes	down
ENSMUSG00000021579	Lrrc14b	leucine rich repeat containing 14B [Source:MGI Symbol;Acc:MGI:2145269]	2.493655593	1.318262224	0.04308274	0.14053103	yes	up
ENSMUSG00000030147	Clec4b1	C-type lectin domain family 4, member b1 [Source:MGI Symbol;Acc:MGI:1917060]	2.110889686	1.077851186	0.04314044	0.14066614	yes	up
ENSMUSG00000026610	Esrrg	estrogen-related receptor gamma [Source:MGI Symbol;Acc:MGI:1347056]	2.226584923	1.154832638	0.04351701	0.14160024	yes	up
ENSMUSG00000107201	5930420M18Rik	RIKEN cDNA 5930420M18 gene [Source:MGI Symbol;Acc:MGI:2441703]	0.395861805	-1.336931219	0.0436442	0.14177394	yes	down
ENSMUSG00000041798	Gck	glucokinase [Source:MGI Symbol;Acc:MGI:1270854]	2.104167452	1.073249521	0.04382457	0.14216624	yes	up
ENSMUSG00000095348	Gm3892	predicted gene 3892 [Source:MGI Symbol;Acc:MGI:3782065]	3.013064333	1.591231477	0.04384409	0.14216624	yes	up
ENSMUSG00000094695	Gm21953	predicted gene, 21953 [Source:MGI Symbol;Acc:MGI:5439404]	18.60058553	4.217276132	0.04385612	1	yes	up
ENSMUSG00000060530	A930017M01Rik	RIKEN cDNA A930017M01 gene [Source:MGI Symbol;Acc:MGI:2685151]	4.177042969	2.062481984	0.04402237	1	yes	up
ENSMUSG00000097239	Gm27029	predicted gene, 27029 [Source:MGI Symbol;Acc:MGI:5504144]	0.39470462	-1.341154689	0.04432661	0.14336728	yes	down
ENSMUSG00000047344	Lancl3	LanC lantibiotic synthetase component C-like 3 (bacterial) [Source:MGI	3.268355386	1.708564864	0.04434202	0.14336728	yes	up
ENSMUSG00000057751	Megf6	multiple EGF-like-domains 6 [Source:MGI Symbol;Acc:MGI:1919351]	2.435820459	1.284407798	0.0444042	0.14348781	yes	up
ENSMUSG00000049107	Ntf3	neurotrophin 3 [Source:MGI Symbol;Acc:MGI:97380]	2.741782409	1.455114082	0.04443634	0.14356483	yes	up
ENSMUSG00000006204	Cdcp3	CUB domain containing protein 3 [Source:MGI Symbol;Acc:MGI:1918645]	2.383741907	1.25322804	0.04446114	0.14359993	yes	up

ENSMUSG00000021057	Akap5	A kinase (PRKA) anchor protein 5 [Source:MGI Symbol;Acc:MGI:2685104]	0.34841818	-1.52110819	0.04458028	0.14381487	yes	down
ENSMUSG00000026686	Lmx1a	LIM homeobox transcription factor 1 alpha [Source:MGI Symbol;Acc:MGI:1888519]	2.445201262	1.289953217	0.04479184	0.14428197	yes	up
ENSMUSG00000117919	Gm41717	predicted gene, 41717 [Source:MGI Symbol;Acc:MGI:5624602]	18.39031778	4.200874505	0.04489858	1	yes	up
ENSMUSG00000113701	B230303A05 Rik	RIKEN cDNA B230303A05 gene [Source:MGI Symbol;Acc:MGI:3646895]	0.17206113	-2.539006876	0.0451584	0.14506869	yes	down
ENSMUSG00000116207	Nnt	nicotinamide nucleotide transhydrogenase [Source:MGI Symbol;Acc:MGI:109279]	0.00439526	-7.829835756	0.04516713	0.14506869	yes	down
ENSMUSG00000062391	4932435O22 Rik	RIKEN cDNA 4932435O22 gene [Source:MGI Symbol;Acc:MGI:2442791]	6.591204269	2.720542082	0.04524301	1	yes	up
ENSMUSG00000059406	Tmprss9	transmembrane protease, serine 9 [Source:MGI Symbol;Acc:MGI:3612246]	0.055286805	-4.176920988	0.04544629	1	yes	down
ENSMUSG00000021966	Prss52	protease, serine 52 [Source:MGI Symbol;Acc:MGI:1920632]	0.055286805	-4.176920988	0.04544629	1	yes	down
ENSMUSG00000105971	Gm43805	predicted gene 43805 [Source:MGI Symbol;Acc:MGI:5663942]	0.055286805	-4.176920988	0.04544629	1	yes	down
ENSMUSG00000044499	Hs3st5	heparan sulfate (glucosamine) 3-O-sulfotransferase 5 [Source:MGI Symbol;Acc:MGI:2441996]	3.136199851	1.649017497	0.0454664	1	yes	up
ENSMUSG00000117902	Gm41715	predicted gene, 41715 [Source:MGI Symbol;Acc:MGI:5624600]	0.067497622	-3.889019508	0.04562083	1	yes	down
ENSMUSG00000021680	Crhbp	corticotropin releasing hormone binding protein [Source:MGI Symbol;Acc:MGI:88497]	0.114854941	-3.122115171	0.04579868	1	yes	down
ENSMUSG00000073879	Gm5859	predicted pseudogene 5859 [Source:MGI Symbol;Acc:MGI:3704096]	2.273213247	1.184733028	0.0458126	0.14642416	yes	up
ENSMUSG00000082419	Gm11425	predicted gene 11425 [Source:MGI Symbol;Acc:MGI:3650957]	26.65079558	4.736106696	0.04599941	1	yes	up
ENSMUSG00000086754	Gm16098	predicted gene 16098 [Source:MGI Symbol;Acc:MGI:3801762]	0.038751543	-4.689602448	0.0465147	1	yes	down
ENSMUSG00000111008	Gm31562	predicted gene, 31562 [Source:MGI Symbol;Acc:MGI:5590721]	0.051534285	-4.27832364	0.04671948	1	yes	down
ENSMUSG00000103053	Gm38271	predicted gene, 38271 [Source:MGI Symbol;Acc:MGI:5611499]	0.051534285	-4.27832364	0.04671948	1	yes	down
ENSMUSG00000045331	2310079G19 Rik	RIKEN cDNA 2310079G19 gene [Source:MGI Symbol;Acc:MGI:1916949]	0.353755334	-1.499176195	0.04677953	0.14841838	yes	down
ENSMUSG00000102224	4930447F24Rik	RIKEN cDNA 4930447F24 gene [Source:MGI Symbol;Acc:MGI:1924123]	3.671565551	1.876395358	0.04696486	1	yes	up
ENSMUSG00000006522	Itih3	inter-alpha trypsin inhibitor, heavy chain 3 [Source:MGI Symbol;Acc:MGI:96620]	2.662609939	1.412841095	0.047102	0.14913909	yes	up
ENSMUSG00000078651	Aoc2	amine oxidase, copper containing 2 (retina-specific) [Source:MGI Symbol;Acc:MGI:2668431]	0.484397693	-1.0457361	0.04739829	0.14966676	yes	down
ENSMUSG00000073375	Lrrc30	leucine rich repeat containing 30 [Source:MGI Symbol;Acc:MGI:2685172]	2.443883066	1.289175258	0.0476533	0.15016956	yes	up
ENSMUSG00000104684	5430427N15 Rik	RIKEN cDNA 5430427N15 gene [Source:MGI Symbol;Acc:MGI:2441699]	0.482638061	-1.050986402	0.0478496	0.15062344	yes	down
ENSMUSG00000100465	Gm29264	predicted gene 29264 [Source:MGI Symbol;Acc:MGI:5579970]	12.87160697	3.686120274	0.04793477	1	yes	up
ENSMUSG00000010797	Wnt2	wingless-type MMTV integration site family, member 2 [Source:MGI Symbol;Acc:MGI:98954]	2.178257443	1.123174473	0.0479348	0.15078181	yes	up
ENSMUSG00000051860	Samd7	sterile alpha motif domain containing 7 [Source:MGI Symbol;Acc:MGI:1923203]	0.109065041	-3.196739355	0.04847393	1	yes	down
ENSMUSG00000105161	Gm42595	predicted gene 42595 [Source:MGI Symbol;Acc:MGI:5662732]	0.25039653	-1.997713526	0.04848408	0.15187402	yes	down
ENSMUSG00000056296	Synpr	synaptoporin [Source:MGI Symbol;Acc:MGI:1919253]	2.476000923	1.308011853	0.04868773	0.15220859	yes	up
ENSMUSG00000046748	Tmem45a2	transmembrane protein 45A2 [Source:MGI Symbol;Acc:MGI:1916707]	9.119935376	3.189023602	0.04878657	1	yes	up
ENSMUSG00000108617	Gm31749	predicted gene, 31749 [Source:MGI Symbol;Acc:MGI:5590908]	0.056579368	-4.143580137	0.04894518	1	yes	down
ENSMUSG00000046719	Nxph3	neurexophilin 3 [Source:MGI Symbol;Acc:MGI:1336188]	2.038433521	1.027460907	0.04904215	0.15301221	yes	up
ENSMUSG00000116940	Gm7450	predicted gene 7450 [Source:MGI Symbol;Acc:MGI:3647897]	0.122269298	-3.031865911	0.04915697	1	yes	down
ENSMUSG00000111055	D030034A15 Rik	RIKEN cDNA D030034A15 gene [Source:MGI Symbol;Acc:MGI:2442288]	2.884929314	1.528535971	0.04917226	1	yes	up

ENSMUSG00000059040	Eno1b	enolase 1B, retrotransposed [Source:MGI Symbol;Acc:MGI:3648653]	0.286920091	-1.8012791	0.04941009	0.15396567	yes	down
ENSMUSG00000108782	Gm32772	predicted gene, 32772 [Source:MGI Symbol;Acc:MGI:5591931]	5.308808895	2.408388209	0.04944475	1	yes	up
ENSMUSG00000070304	Scn2b	sodium channel, voltage-gated, type II, beta [Source:MGI Symbol;Acc:MGI:106921]	2.240575379	1.163869263	0.04965612	0.15456516	yes	up
ENSMUSG00000003545	Fosb	FBJ osteosarcoma oncogene B [Source:MGI	4.08034242	2.028690227	0.0497293	0.15470939	yes	up
ENSMUSG00000079103	Tgm7	transglutaminase 7 [Source:MGI Symbol;Acc:MGI:2151164]	0.262054622	-1.932060542	0.04974526	1	yes	down
ENSMUSG00000001095	Slc13a2	solute carrier family 13 (sodium-dependent dicarboxylate transporter), member 2 [Source:MGI Symbol;Acc:MGI:1276558]	0.323796918	-1.626838842	0.04977099	1	yes	down
ENSMUSG00000019856	Fam184a	family with sequence similarity 184, member A [Source:MGI Symbol;Acc:MGI:1923156]	2.079445282	1.056198723	0.04985498	0.15498884	yes	up
ENSMUSG00000078487	Ankrd65	ankyrin repeat domain 65 [Source:MGI	0.112752493	-3.148768769	0.04993339	1	yes	down
ENSMUSG00002075453			41.78780738	5.385010157	0.04995372	1	yes	up
ENSMUSG00002074955	Snord3b4	small nucleolar RNA, C/D box 3B4 [Source:NCBI gene (formerly Entrezgene);Acc:19861]	41.78780738	5.385010157	0.04995372	1	yes	up
