# Supplementary figures and images for: WFDC12-overexpressing contributes to the development of atopic dermatitis via accelerating ALOX12/15 metabolism and PAF accumulation
Source: Cell Death Dis. 2023 Mar 8;14(3):185. doi: 10.1038/s41419-023-05686-3 (PMC9992393; doi:10.1038/s41419-023-05686-3)

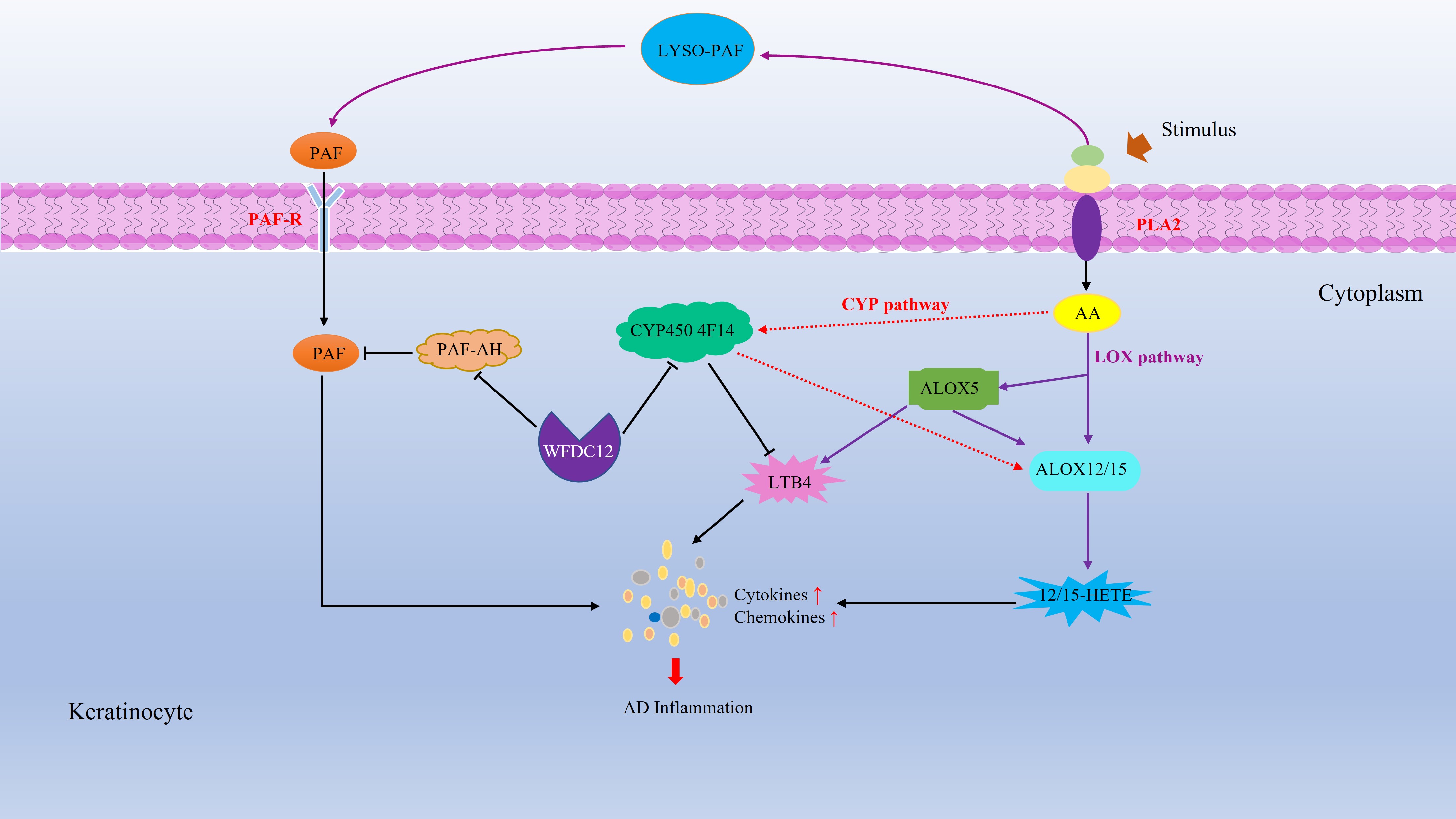

Supplement: Supplementary file 4 — Supplementary Figure S7 [file 41419_2023_5686_MOESM4_ESM.jpg]
